# Pillar[*n*]arene-based phosphine ligands: synthesis, coordination chemistry and application in selective Au(i) catalysis

**DOI:** 10.1039/d6qo00815a

**Published:** 2026-07-20

**Authors:** Antoine Konter, Michele Buccio, Louis Vidal, Céline Besnard, Clément Mazet

**Affiliations:** a Department of Organic Chemistry, University of Geneva 30 quai Ernest Ansermet 1211 Geneva Switzerland clement.mazet@unige.ch; b Laboratory of Crystallography, University of Geneva 24 quai Ernest Ansermet 1211 Geneva Switzerland

## Abstract

We report a divergent and modular synthetic strategy for the functionalization of pillar[*n*]arenes, enabling the synthesis of a comprehensive library of 17 mono- and bisphosphine ligands. By leveraging a Pd-catalyzed phosphinylation of A1/A2-ditriflate-pillararenes, we demonstrate that precise stoichiometric control permits the isolation of bifunctional phosphine-triflate intermediates. These scaffolds serve as versatile pivotal intermediates for the construction of rim-differentiated bisphosphines and hybrid aryl-phosphine derivatives *via* sequential Pd-catalyzed cross-couplings. Coordination studies with gold(i) precursors, supported by X-ray crystallography, reveal a certain degree of conformational plasticity inherent to the macrocyclic backbone. The catalytic utility of these macromolecular ligands was validated in the gold(i)-catalyzed cycloisomerization of 1,6-enynes, where several derivatives exhibited excellent levels of regioselectivity.

## Abstract 2

A divergent Pd-catalyzed phosphinylation strategy giving access to 17 pillar[*n*]arene-based mono- and bisphosphine ligands, whose gold(i) complexes deliver high regioselectivity in 1,6-enyne cycloisomerization, is reported.

## Graphical abstract

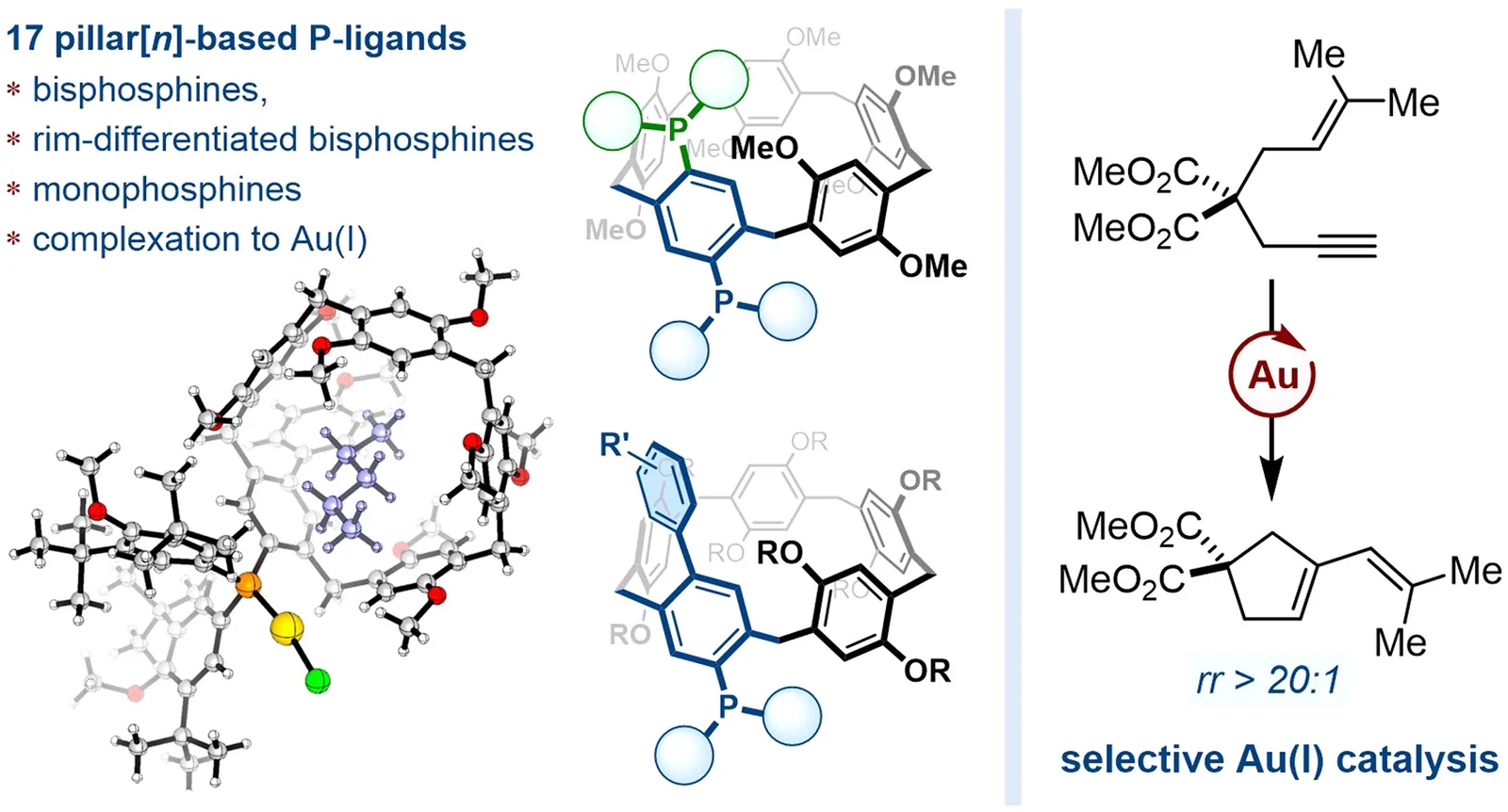

## Introduction

**Fig. 1:**
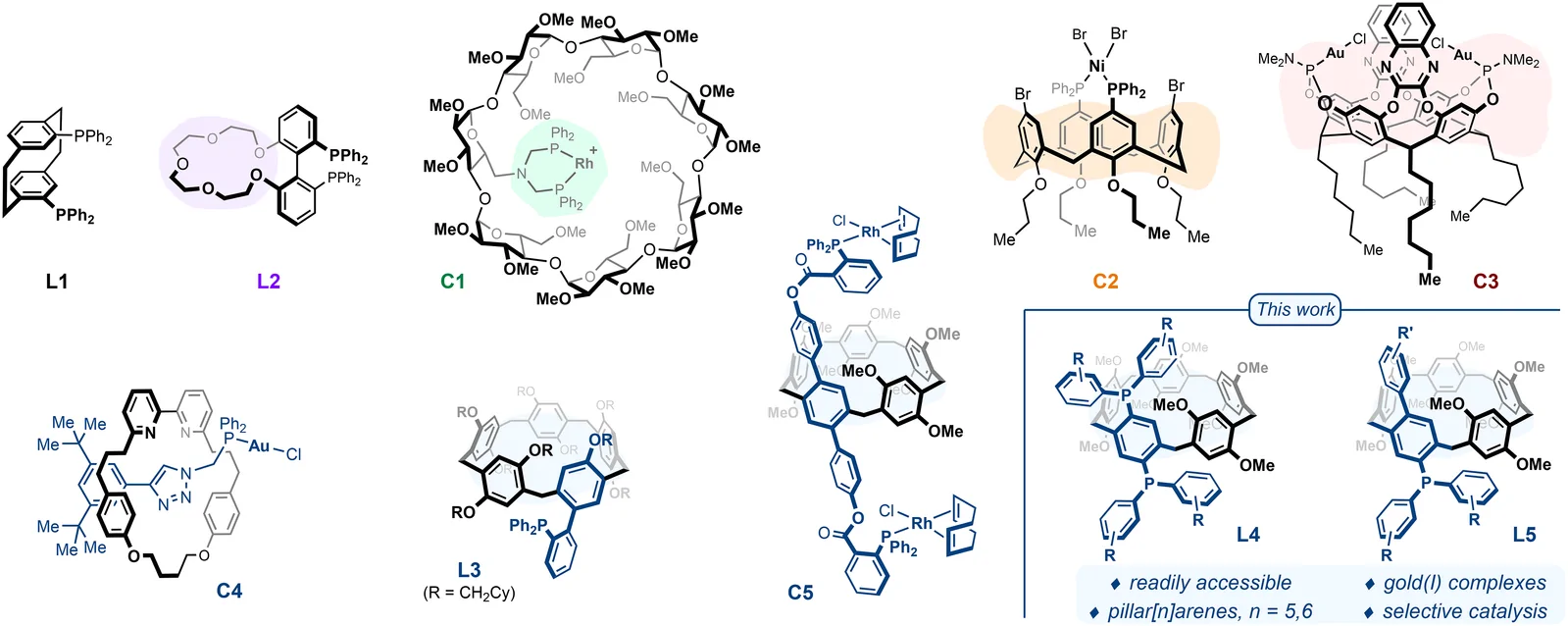
Non-exhaustive representative selection of monophosphine and bisphosphine ligands (L1–L3) as well as transition metal complexes thereof (C1–C5) incorporating macrocyclic scaffolds. This work: bisphosphine and monophosphine ligands based on pillar[*n*]arenes (L4–L5).

Macrocycles are cyclic molecules containing a ring of at least twelve atoms. Whether naturally occurring or synthetic, macrocycles find applications (i) in drug discovery where they occupy a unique region of the chemical space between small-molecule drugs and protein therapeutics;^1–3^ (ii) in material science as building elements for the design of rotaxanes, catenanes, molecular machines, sensors (*etc*.);^4–7^ (iii) in catalysis, where they have been proposed to mimic enzymes by providing a confined and preorganized environment, although the extent to which such host–guest effects operate is strongly system-dependent.^8–13^ More specifically, within this domain, numerous phosphine-based ligands have been elaborated on macrocyclic frameworks for applications in (selective) transition metal catalysis. A non-exhaustive but representative selection of such systems is depicted in Fig. 1.^14–20^ Since it was first reported in 1997, PhanePhos (L1), a planar-chiral *C*_2_-symmetric bisphosphine built on a [2.2]paracyclophane scaffold, has established itself as a so-called privileged ligand for its ability to catalyze mechanistically unrelated reactions with high levels of efficiency and selectivity.^14,21^ Phosphine ligands incorporating a crown-ether framework such as L2 have been found to undergo cation-triggered conformational changes that influence their bite angle and consequently catalytic performances.^17^ In the late 1990s, the Reetz group reported a bisphosphine–Rh complex covalently attached to a permethylated *β*-cyclodextrin (C1) for application in the catalytic hydroformylation of olefins in biphasic media. The system displayed improved regioselectivity for linear aldehydes compared to conventional catalysts, a result attributed to the substrate being encapsulated in the hydrophobic cavity of the macrocyclic support.^15^ Well-defined nickel complexes of phosphorus-containing calix[4]arenes (*e.g.*C2) have shown high levels of activity in the oligomerization of ethylene with often excellent C_6_ selectivity.^16^ The Iwasawa group reported a dinuclear gold(i) complex supported by a resorcin[4]arene-based bisphosphine ligand, where the two metal centers face each other (C3). When methyl 2-acetyloct-7-ynoate was subjected to Lewis acid catalysis, the expected Conia-ene product was not formed. Instead, as a result of the reaction occurring inside the cavity and involving both metal centers, intermolecular dimerization of the terminal alkyne yielded a conjugated enyne.^19^ The Goldup group reported a rotaxane in which one end of the axle is terminated by a (diphenylphosphine)AuCl moiety (C4). Notably, catalytic activity in the Au-catalyzed Ohe–Uemura intermolecular cyclopropanation of styrene was only achieved using zinc or copper additives to prevent inference of the bipyridyl core of the macrocyclic unit with the reactive cationic metal center. Moreover, higher levels of diastereoselectivity were obtained with the stimuli-responsive catalyst compared to the unthreaded native gold complex.^18^ While the development of P-based ligands bound or interlocked to macrocycles – such as crown-ethers, calixarenes, resorcinarenes and *β*-cyclodextrin – has been particularly fertile and has often led to unusual catalytic systems, there is, to the best of our knowledge, only a single example of a phosphine ligand that has been built on pillar[5]arenes for application in transition metal catalysis.^22^ Nagata, Ogoshi, Suginome and coworkers reported the synthesis and resolution of the enantiomers of a planar chiral pillar[5]arene with cyclohexylmethoxy side chains bearing a pendant diarylphosphine unit reminiscent of Buchwald-type ligands (L3).^20,23^ This approach was subsequently validated in the Pd-catalyzed enantioselective hydrosilylation of styrene, even though the level of enantioinduction measured remained modest.

As part of a study aimed at accessing configurationally stable bis-arylated pillar[5]arene derivatives by Dynamic Kinetic Resolution (DKR), our group described access to a bisphosphine ligand and the corresponding dinuclear rhodium(i) complex (C5).^24^ Preliminary attempts to apply this system to transition metal-based catalytic reactions were unsuccessful, leading us to revise our initial strategy. We report herein the syntheses of mono- and bisphosphine ligands (L4–L5) where the P-atom is directly connected to one of the π-panels of the macrocyclic structure by Pd-catalyzed phosphinylation reactions. The study of the coordination chemistry of these ligands as well as their application in a gold-catalyzed selective cycloisomerization reaction is also presented.

## Results and discussion

### Reaction development

The catalytic formation of P–C(sp^2^) bond is a difficult reaction, which finds numerous precedents in the literature.^25–27^ Nevertheless, unlike the situation observed in many Pd-catalyzed cross-coupling reactions (*e.g.*, Suzuki–Miyaura), there is currently no robust and general protocol that would apply to any type of electrophile or nucleophile.^28,29^ For example, some catalytic systems use free phosphines, while others employ phosphine oxides or BH_3_-protected phosphines. Similarly, the electrophilic partner can be an aryl halide or a pseudo-aryl halide, but these are rarely interchangeable, resulting in very specific reaction conditions overall. To tackle this challenge, we began our investigations by subjecting the A1/A2-ditriflate-pillar[5]arene (1) to a series of protocols that have been reported in the literature.^30–32^ Unfortunately, independently of the nature of the phosphine precursor, most of them gave no or little conversion to any bis-phosphinylated cross-coupling product (see SI for details). In two independent studies, Buchwald and Clarke showed that 1,10-bis-(di-*iso*-propylphosphino)-ferrocene (dippf) was effective in the coupling of aryl bromides at elevated temperature.^33,34^ Using the borane-protected phosphine 2a·BH_3_ and a slightly modified version of these protocols (5 mol% Pd(OAc)_2_/reactive site, Et_3_N, DMF : THF (1 : 1), 160 °C; Table 1, entry 1), the product of double cross-coupling (3a) was generated in 58% conversion together with 14% of the inseparable detriflated monophosphine 4a. Of important note, the use of an excess of base enabled us to generate directly the fully deprotected bisphosphine ligand, which was stable enough to be purified *via* silica gel chromatography using a CH_2_Cl_2_ : *n*-pentane : Et_2_O eluent mixture (1 : 1.5 : 1). Whereas the use of DBU and DMAP led to no or poor reactivity (entries 2 and 3), we found that DABCO gave the bis-phosphinylated product 3a in 83% conversion without traces of 4a (entry 4). A similar result was obtained with a reduced stoichiometry (entry 5) but a slightly diminished conversion was obtained when the experiment was conducted in DMF (entry 6). Finally, we found that when the non-protected phosphine precursor 2a was used, the stoichiometry in base could be substantially decreased without compromising the efficiency of the catalytic process and 3a was isolated in 75% yield as a white solid (entry 7). Of note, a nearly identical result was obtained employing Barton's base (entry 8).

**Table 1:** Reaction optimization^a^

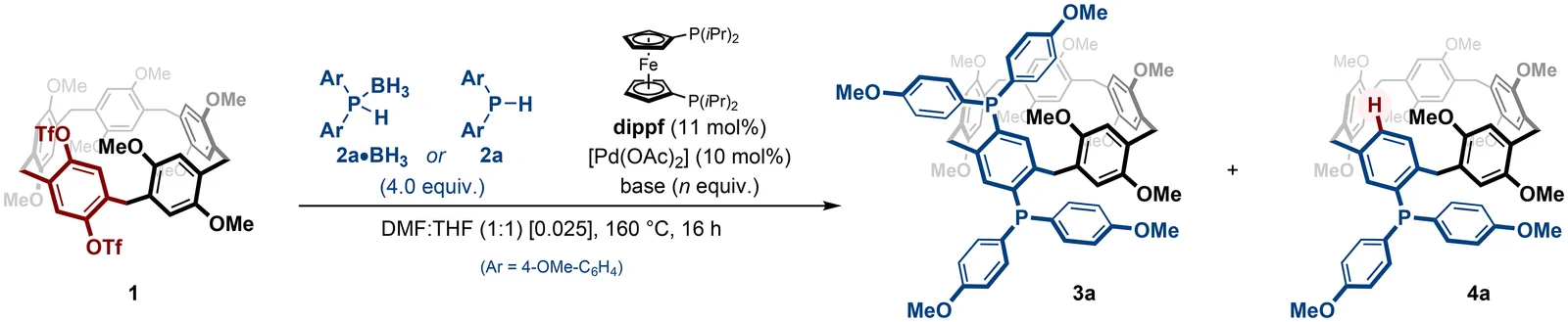				
Entry	Phosphine	Base (equiv.)	Conv. 3a b (%)	Conv. 4a b (%)
1	2a.BH_3_	Et_3_N (12.0)	58	14
2	2a.BH_3_	DBU (12.0)	<1	<1
3	2a.BH_3_	DMAP (12.0)	23	9
4	2a.BH_3_	DABCO (12.0)	83	<1
5	2a.BH_3_	DABCO (8.0)	89	<1
6^c^	2a.BH_3_	DABCO (12.0)	73	<1
7	2a	DABCO (4.0)	90 (75)^d^	<1
8	2a	BTMG (4.0)^e^	88	<1

a Reactions performed on a 0.05 mmol scale. Throughout this study, concentration is that of the limiting reagent (1).

b Conversion determined by ^1^H NMR spectroscopy using an internal standard.

c Reaction performed in DMF.

d Yield after purification.

e BTMG: Barton's base (2-*tert*-butyl-1,1,3,3-tetramethylguanidine).

**Fig. 2:**
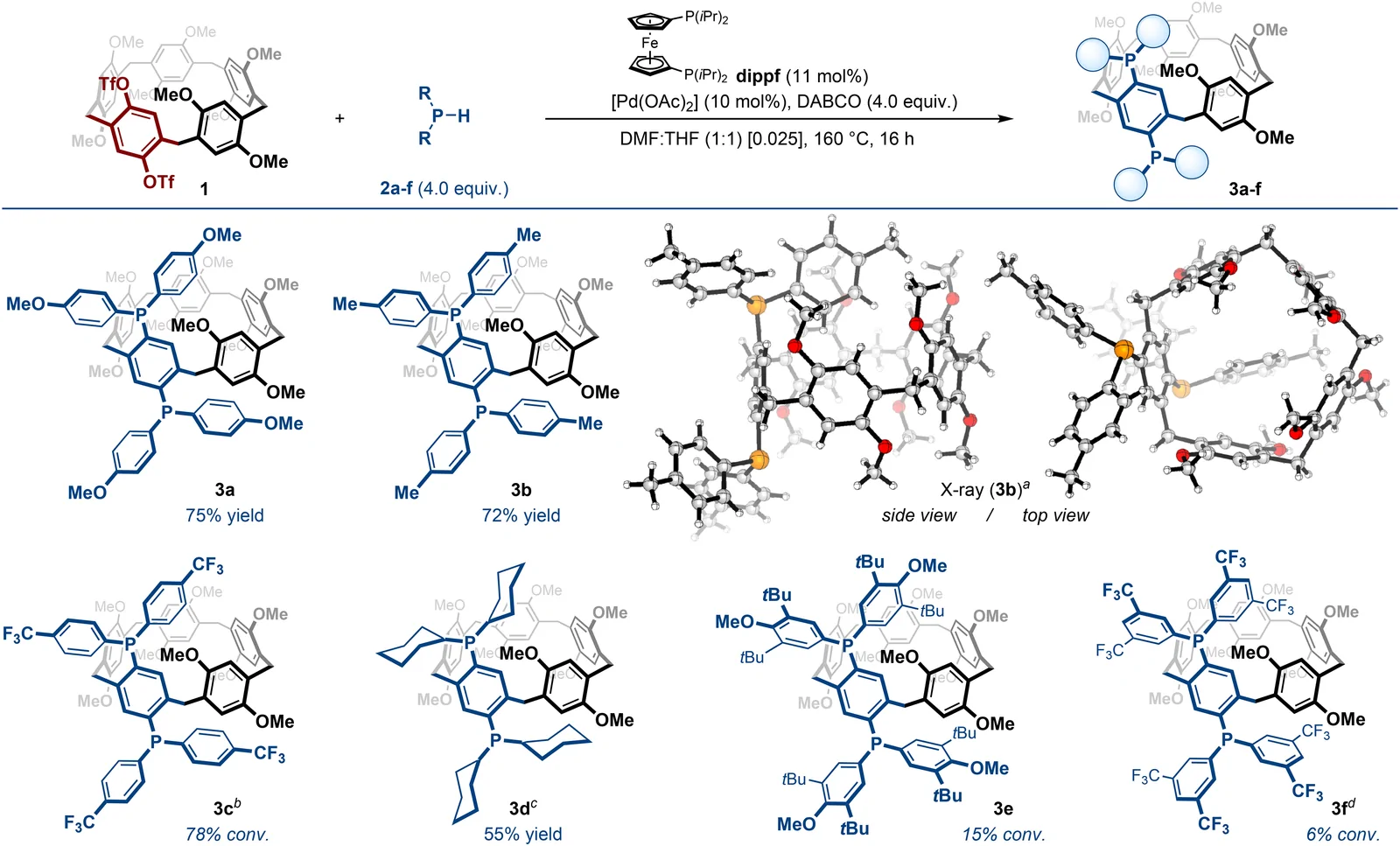
Scope of the Pd-catalyzed diphosphinylation of A1/A2-ditriflate-pillar[5]arene 1. ^*a*^ Color code: C (grey), O (red), P (orange), H (light grey). The molecule of CH_2_Cl_2_ located inside the annulus of the macrocycle has been omitted for clarity. ^*b*^ Along with *ca*. 12% of the corresponding A1/A2-phosphine-triflate-pillar[5]arene. ^*c*^ Using 8 equiv. of DABCO and 4 equiv. of 2e·BH_3_. ^*d*^ Contains *ca.* 10–15% of the corresponding A1/A2-phosphine-triflate-pillar[5]arene.

The generality of the optimized reaction conditions was evaluated across a range of diverse phosphines (2a–f) on scales ranging from 25 mg to 0.5 g of 1 (Fig. 2). Starting from di-*p*-tolylphosphine 2b, 3b was isolated in a similar yield after purification (72%). Crystals of sufficient quality for an X-ray diffraction analysis were grown by layering of a dichloromethane solution with *n*-hexane. Noticeably, the solid-state structure of 3b shows that while the lone pair of one phosphorus atom is pointing above the cavity of the macrocyclic structure, the second lone pair is oriented outward, suggesting free rotation around the newly formed P–C bonds is possible.

The use of an electron-poor diarylphosphine such as 2c proved to be compatible with the catalytic method. Unfortunately, despite an excellent conversion (78%), the lipophilic nature of the bisphosphine generated (3c) precluded its separation from the monophosphine byproduct (5c). Satisfactorily, using Barton's base instead of DABCO, the bis(dicyclohexyl)phosphine **3**d was isolated in 55% yield starting from dicyclohexylphosphine 2d. As anticipated, this electron-rich ligand was found to be sensitive toward oxidation if exposed to a standard laboratory atmosphere over prolonged periods of time. The diarylphosphine precursors 2e and 2f with large *meta* substituents led to only poor conversion of the targeted products 3e and 3f. In the latter case, the reaction mixture contained *ca.* 10–15% of the (non-detriflated) monophosphinylation product (*vide infra*).

**Fig. 3:**
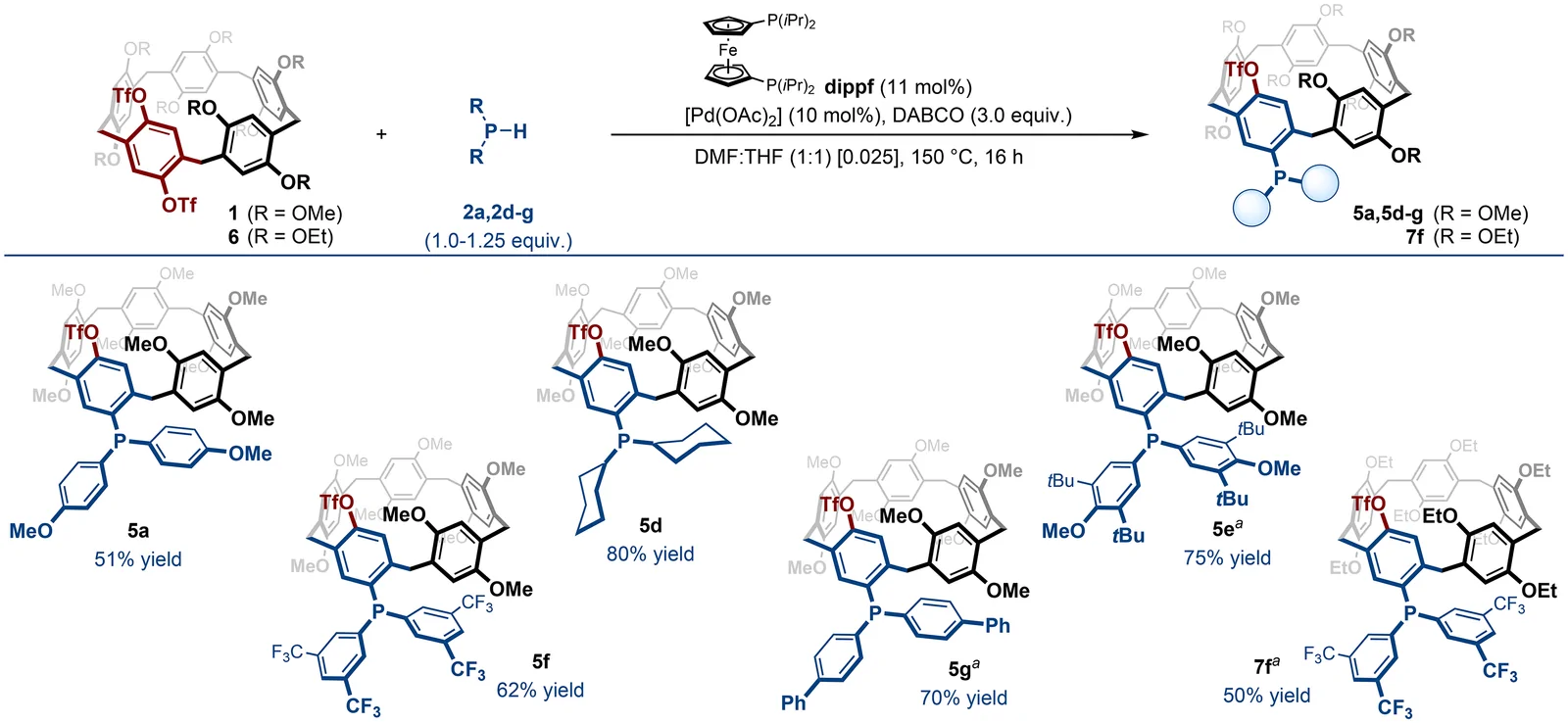
Pd-catalyzed monophosphinylation of A1/A2-ditriflate-pillar[5]arenes 1 and 6. ^*a*^ Isolated as the corresponding phosphine oxides (see SI).

**Fig. 4:**
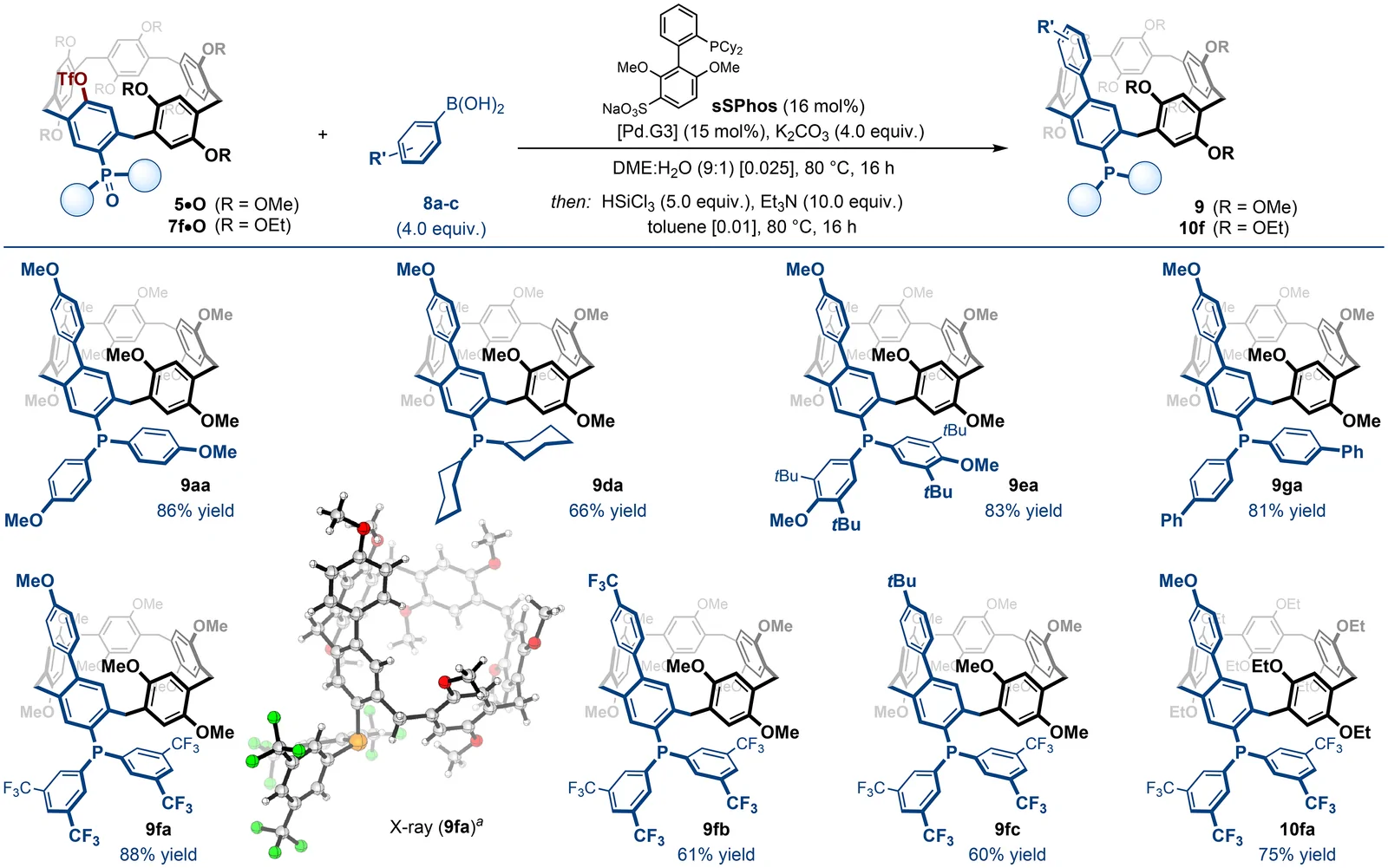
Pd-catalyzed Suzuki–Miyaura cross-coupling of A1/A2-phosphinoxide-triflate-pillar[5]arenes 5·O and 7·O. ^*a*^ Color code: C (grey), O (red), P (orange), F (light green), H (light grey).The molecule of CH_2_Cl_2_ located inside the annulus of the macrocycle has been omitted for clarity.

**Fig. 5:**
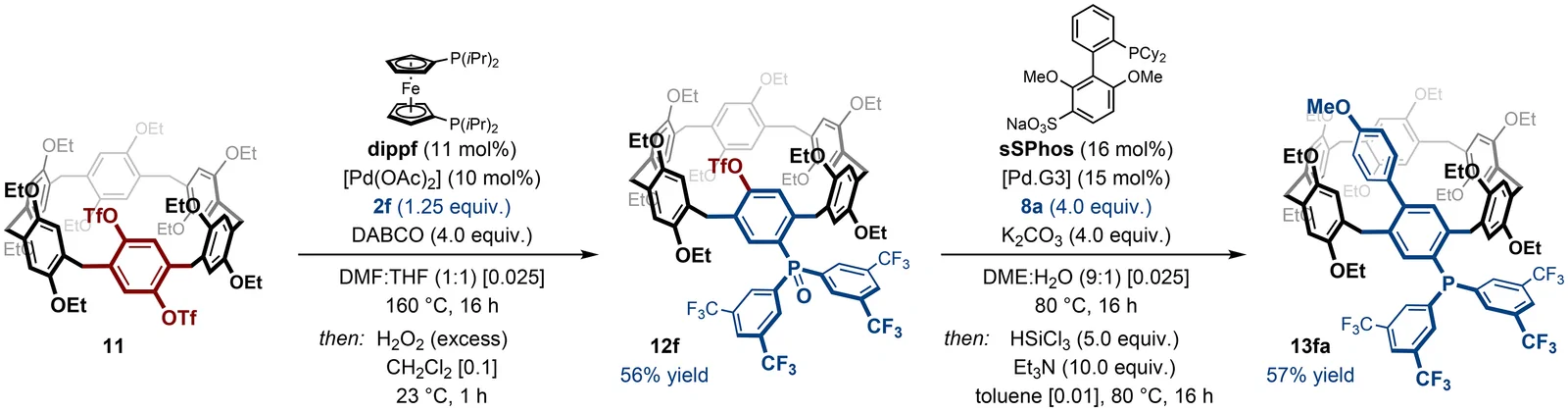
Sequence of Pd-catalyzed phosphinylation/oxidation/Pd-catalyzed Suzuki–Miyaura cross-coupling/reduction of A1/A2-ditriflate-pillar[6]arene 11.

**Fig. 6:**
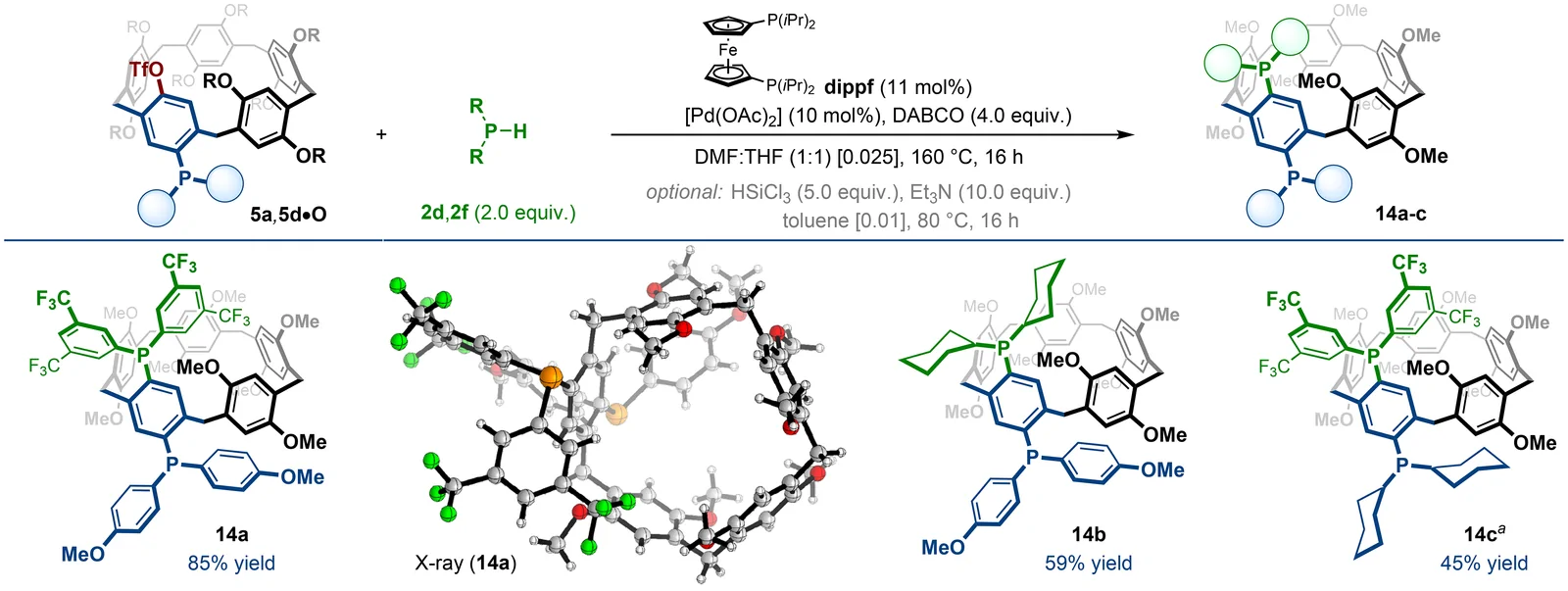
Pd-catalyzed phosphinylation of A1/A2-phosphine-triflate-pillar[5]arenes. ^*a*^ Using 5d·O. Yield over two steps after reduction of the bisphosphine mono-oxide (see SI). Color code: C (grey), O (red), P (orange), F (light green), H (light grey).

This observation prompted us to deliberately try to synthesize the products of monophosphinylation to access rim-differentiated derivatives subsequently. By reducing the stoichiometry in phosphine and base and by conducting the catalytic reaction at 150 °C, several A1/A2-phosphine-triflate-pillar[5]arenes (5a, 5d–f) were isolated in a range of satisfactory yields (51–80%), including when using phosphine precursors that did not provide access to the analogous diphosphinylated products (Fig. 3). Next, emphasis was placed on identifying conditions suitable for converting the products of the Pd-catalyzed monophosphinylation into A1/A2-aryl-phosphine-pillar[5]arene derivatives by a Pd-catalyzed Suzuki–Miyaura cross-coupling reaction. We anticipated that the free monophosphines 5a, 5d–g and 7 – or their borane-protected analogues that would likely be deprotected under the necessary basic conditions – may poison the palladium catalyst. Consequently, they were all converted quantitatively into the corresponding phosphine oxides by simple treatment with an excess of hydrogen peroxide (see SI). Subjecting these compounds to the conditions developed in our previous study for the dynamic kinetic resolution of A1/A2-ditriflate-pillar[5]arenes *via* a Pd-catalyzed Suzuki–Miyaura cross-coupling, followed by a high-yielding reduction enabled us to rapidly generate a small collection of eight macrocycle-based monophosphine ligands (Fig. 4). These compounds were obtained as white solids after purification by silica gel column chromatography. The lone-pair of the P atom in the solid-state structure of 9fa is essentially aligned with the C–C bond of one methylene bridge, while the macrocyclic core retains a regular pillar-shape where the π-panels are all aligned. The synthetic sequence consisting of a Pd-catalyzed phosphinylation/oxidation/Pd-catalyzed Suzuki–Miyaura cross-coupling/reduction was applied to the literature-known A1/A2-ditriflate-pillar[6]arene (11) to afford the corresponding A1/A2-aryl-phosphine-pillar[6]arene 13fa in 32% yield over 4 steps (Fig. 5).^35^ We next established that A1/A2-phosphine-triflate-pillar[5]arenes can be engaged in a second Pd-catalyzed phosphinylation reaction to prepare rim-differentiated bisphosphine ligands in moderate to good yields (Fig. 6). A small but representative selection of structures was chosen to demonstrate that it is possible to synthesize derivatives combining an electron-rich diaryl phosphine moiety with an electron-poor diaryl phosphine unit (14a) as well as other variants combining diaryl phosphine moieties with a dialkyl phosphine fragment (14b–c). Remarkably, with this sequential approach we could also prepare bisphosphine 3f in 80% yield – a derivative that was not accessible *via* the direct diphosphinylation of 1 (*vide supra*) (see SI). The solid-state structure of 14a shows that the *P*-aryl substituents are projected away from the macrocyclic core and the lone pairs of the P atoms are oriented in opposite direction and are located primarily above the cavity.

### Organometallic synthesis

**Fig. 7:**
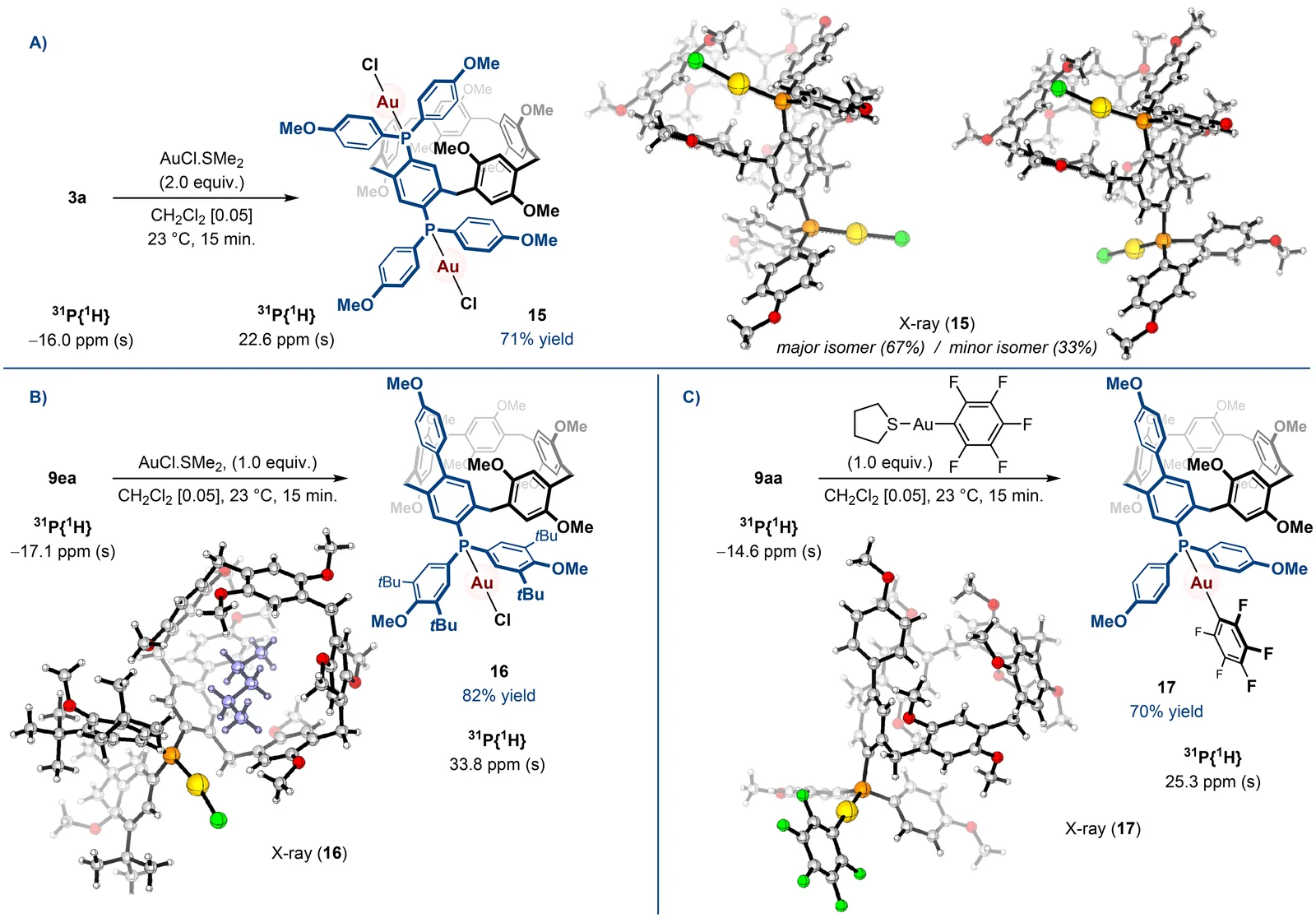
(A–C) Coordination chemistry of pillar[5]arene-based mono- and bis-phosphine ligands to Au(i). Color code: C (grey), O (red), P (orange), Au (yellow), Cl (green), F (light green), H (light grey). The encapsulated pentane molecule in 16 is shown in silver color.

In order to obtain a preliminary understanding of the coordination chemistry of both mono- and bisphosphine-based pillararene ligands, a series of gold(i) complexes was prepared following literature protocols.^36^ The reaction between bisphosphine ligand 3a and 2.0 equivalents of [(Me_2_S)AuCl] led to the formation of the corresponding dinuclear gold(i) complex 15 in high yield (Fig. 7A). X-ray diffraction analysis revealed the presence of two isomeric structures in the solid-state in a 67/33 ratio. In the major species, the vertices defined by the P–Au–Cl axes are oriented in opposite directions in a nearly perfect linear arrangement (〈Au–P–P–Au〉 = 177.74°). In the minor isomer, these are pointing above the cavity (〈Au–P–P–Au〉 = 48.28°). Noticeably, in both structures, the macrocyclic pillararene skeleton is highly distorted and does not exhibit the regular pentagonal shape found in the related free bisphosphine ligands 3b, 9fa and 14a. Observation of a single and sharp resonance at 22.6 ppm by ^31^P NMR spectroscopy at ambient temperature suggests that complex 15 is highly dynamic in solution with (at least partial) rotation of the π-panels through the annulus of the macrocycle and rotation around the P–C bond formed by phosphinylation. It is worth emphasizing that preorganization in macrocyclic ligands does not necessarily imply a rigid, lock-and-key pocket. The pillararene backbone of our monodentate P-ligands retains an appreciable degree of conformational plasticity, as evidenced both by the two solid-state isomers of 15 and by its dynamic behavior in solution. Rather than a rigid cavity, the scaffold is best regarded as a defined but adaptable platform that projects a controlled steric and electronic environment onto the metal center. This picture is fully consistent with the catalytic results, in which the regioselectivity tracks the nature of the *P*-substituents of the mononuclear active species and not the distorted dinuclear motif observed for 15. Synthesis of an analogous mononuclear gold(i) species (16) was achieved starting from 9ea using 1.0 equivalent of [(Me_2_S)AuCl] (Fig. 7B). Crystals of suitable quality for X-ray analysis were grown by slow diffusion of *n*-pentane through a saturated CH_2_Cl_2_ solution of 16. The P–Au and Au–Cl interatomic distances are in line with those measured for similar triarylphosphine complexes (2.2718(11) Å and 2.262(2) Å respectively) and the nearly linear geometry around the metal center is characterized by a 〈P–Au–Cl〉 angle of 170.98(7)°.^36–38^ In solution, the downfield shift of the singlet detected by ^31^P{^1^H} from −17.1 ppm for 9ea to 33.8 ppm is also indicative of effective binding to the metal center. The macrocyclic core structure retains a regular pentagonal pillar shape will all five π-panels aligned and accommodates one molecule of *n*-pentane. The reaction of 9aa with a stoichiometric amount of [(tetrahydrothiophen)Au(C_6_F_5_)] in dichloromethane led to the formation of a mononuclear gold(i) complex (17) as evidenced from solution and solid-state analyses (Fig. 7C). The X-ray diffraction study shows that, while the four 1,4-dimethoxyaryl *p*-units are essentially aligned, the A1/A2-disubstituted aromatic ring is tilted, with the *p*-methoxy aryl substituent oriented toward the center of the macrocycle and the sterically bulky diarylphosphine moiety outward. Moreover, the electron-deficient pentafluoroarene coordinated to the gold atom is pointing away from the electron-rich cavity of the pillar[5]arene, which is likely too narrow to favor any interaction.

### Selective Au(i) catalysis

The gold-catalyzed intramolecular cycloisomerization of 1,6-enyne 18 was elected as a benchmark reaction to evaluate the potential of the mono- and bisphosphine-based pillararene ligands in selective catalysis.^39,40^ A catalyst loading of 5 mol% in [Au] was employed throughout, in line with established protocols for this benchmark cycloisomerization, so as to allow a direct comparison of the pillararene-based ligands with the reference catalyst [(Ph_3_P)AuCl] (22) under strictly identical conditions. A representative selection of the results obtained is disclosed in Table 2. All reactions were run with a catalyst loading of 5 mol% in [Au] and were calibrated using [(Ph_3_P)AuCl] (22) as reference (94% conv., rr_19__/__20_ = 1 : 8.0; entry 1). We obtained comparable outcomes using an *in situ* protocol, in which the ligand and metal source are premixed, or an *ex situ* protocol employing isolated, well-defined gold complexes. Similarly, no significant difference was observed in the performances achieved when using AgSbF_6_ or NaBAr_F_ as halide scavengers. Nonetheless, we found the latter to be more practical and reliable, as it does not require conducting the experiments in the dark, thereby avoiding any reproducibility issues (see SI for details). The bisphosphine-based pillararene ligands 3a and 14a gave results comparable to 22, with preferential formation of the exocyclic product 20 (entries 1–3). In the monophosphine series, the *P*-substituents were found to exert a strong influence on the reaction outcome. While 9aa (P_Ar_ = 4-OMe-C_6_H_4_) and 9ga (P_Ar_ = 4-Ph-C_6_H_4_) led to regioisomeric ratio in the range of those measured with the other ligands (entries 4 and 7), the dicyclohexyl derivative 9da exhibited a markedly improved selectivity (rr_19__/__20_ = 1 : 16.1, entry 5). By contrast, a greater amount of the endocyclic product 20 was generated with 9ea (P_Ar_ = 3,5-di-*tert*-butyl-4-methoxyphenyl) (rr_19__/__20_ = 1 : 4.1, entry 6). The highest level of selectivity was obtained with 9fa (P_Ar_ = 3,5-(CF_3_)_2_–C_6_H_3_) (rr_19__/__20_ = 1 : 23.1, entry 8). Variation of the aryl moiety introduced by Pd-catalyzed Suzuki–Miyaura cross-coupling (9fb) or use of the analogous pillar[6]arene ligand (13fa) led to lower regioselectivity (entries 9 and 10). Although a detailed mechanistic picture awaits a dedicated computational study, the data in Table 2 already point to a coherent qualitative rationale. Within the monophosphine series, the *exo*/*endo* (19/20) selectivity correlates with the steric and electronic environment projected onto the linear, two-coordinate Au(i) center by the *P*-substituents rather than with the size of the macrocyclic cavity: the electron-poor, sterically compact bis[3,5-(CF_3_)_2_C_6_H_3_] ligand 9fa affords the highest selectivity (rr_19__/__20_ = 1 : 23.1), whereas the bulky, electron-rich 3,5-di-*tert*-butyl-4-methoxyphenyl ligand 9ea erodes it (rr_19__/__20_ = 1 : 4.1). This is consistent with the generally accepted mechanism in which a more electrophilic, less encumbered gold center favors the anti-Markovnikov 5-*exo-dig* pathway leading to 20. The observation that the regular pentagonal pillar shape is retained in the mononuclear complexes (*e.g.*16, 17) further supports the view that the pillararene backbone acts as a defined but conformationally adaptable ligand platform rather than as a rigid, substrate-encapsulating pocket in these transformations. The propensity of pillararenes to accommodate small molecules within their cavity, prompted us to investigate whether the nature of the solvent could influence regioselectivity in the Au-catalyzed cycloisomerization of 18 by means of host–guest interactions.^41,42^ Using 1,2-dichloroethane – the templating solvent employed in the synthesis of the native pillar[5]arenes – instead of CD_2_Cl_2_, led to lower regioselectivities with both 22 and 9fa (compare entries 1, 8, 11 and 12). Similar trends were observed when 1,4-dibromobutane was employed (compare entries 1, 8, 10 and 13–15) and with chlorocyclohexane, which has been shown to accommodate within the cavity of pillar[6]arenes but not that of pillar[5]arene (compare entries 1, 8, 10 and 16–18). Two observations argue against an operative host–guest interaction as the origin of these variations. First, the cavity-free reference catalyst 22 shows the same qualitative solvent dependence as the pillararene-based catalysts, indicating that the trend is not a specific property of the macrocyclic cavity. Second, chlorocyclohexane, which is reported to be accommodated within pillar[6]arene but not within pillar[5]arene, does not induce the pillar[5]/pillar[6]-dependent divergence in regioselectivity that a size-selective encapsulation would be expected to produce (compare the pillar[5]arene ligand 9fa with the pillar[6]arene ligand 13fa). Taken together, these results indicate that the observed variations in regioselectivity are governed primarily by an overall solvent effect rather than by a host–guest interaction between the macrocycle and the haloalkane.

**Table 2:** Au(i)-catalyzed intramolecular cycloisomerization of 1,6-enyne 18 a

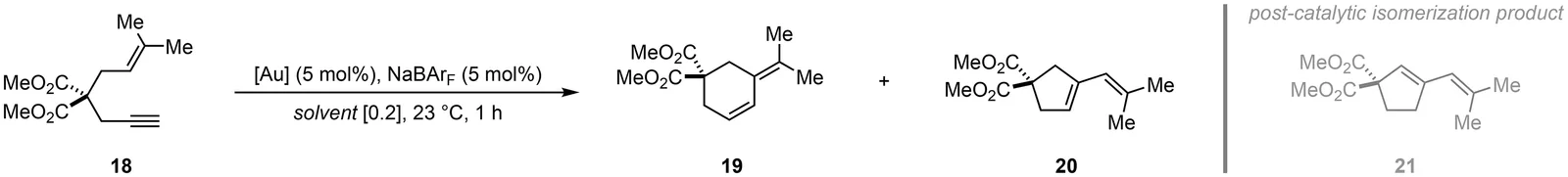									
Entry	[Au]	Solvent	Conv.^b^ (%)	rr_19__/__20_ b	Entry	[Au]	Solvent	Conv.^b^ (%)	rr_19__/__20_^b^
1	22	CD_2_Cl_2_	94	1 : 8	10	13fa	CD_2_Cl_2_	98	1 : 10.6
2	3a	CD_2_Cl_2_	97	1 : 10.8	11	22	ClCH_2_CH_2_Cl	96	1 : 6.3
3	14a	CD_2_Cl_2_	95	1 : 9.4	12	9fa	ClCH_2_CH_2_Cl	95	1 : 9.9
4	9aa	CD_2_Cl_2_	>99	1 : 7.0	13	22	Br(CH_2_)_4_Br	>99	1 : 3.8
5	9da	CD_2_Cl_2_	94	1 : 16.1	14	9fa	Br(CH_2_)_4_Br	>99	1 : 10.7^d^
6	9ea	CD_2_Cl_2_	97	1 : 4.1^c^	15	13fa	Br(CH_2_)_4_Br	>99	1 : 5.6^f^
7	9ga	CD_2_Cl_2_	95	1 : 10	16	22	C_6_H_11_Cl	97	1 : 4.6
8	9fa	CD_2_Cl_2_	98	1 : 23.1^d^	17	9fa	C_6_H_11_Cl	73	1 : 7.8
9	9fb	CD_2_Cl_2_	73	1 : 13.7^e^	18	13fa	C_6_H_11_Cl	95	1 : 6.3
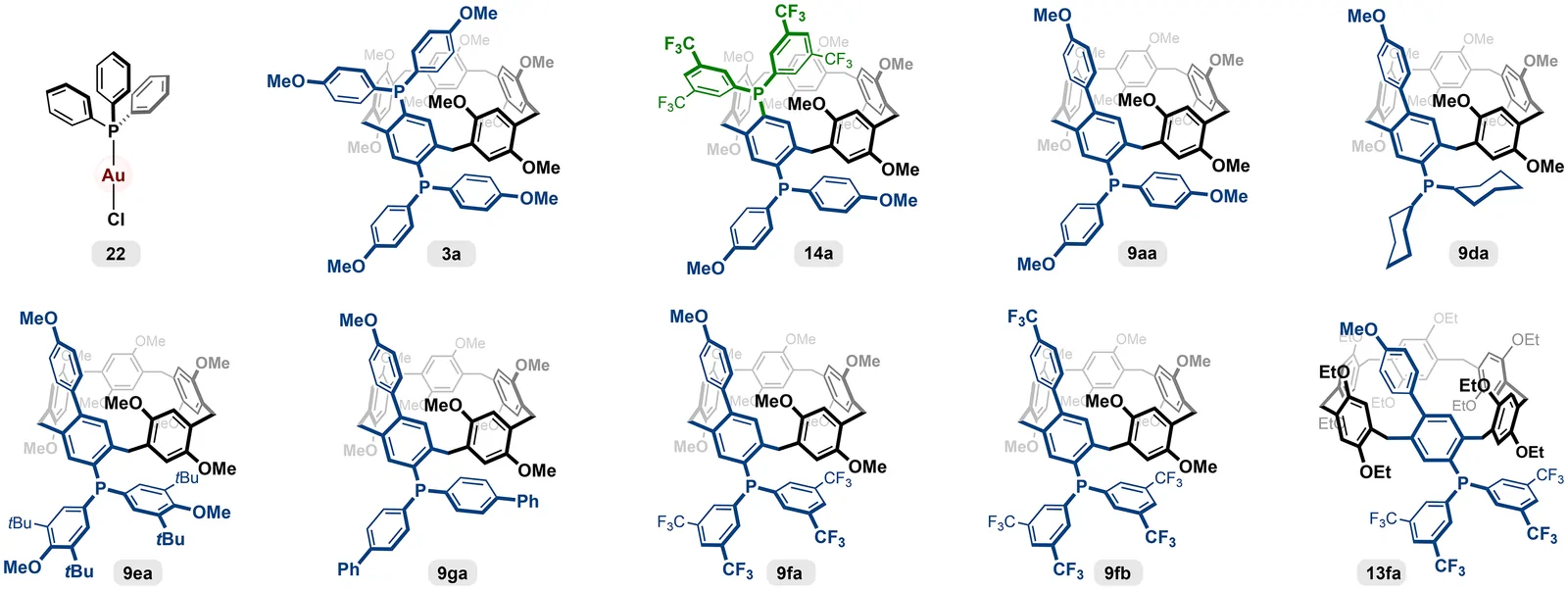									

a Reactions performed on a 0.1 mmol scale. Reactions performed indifferently *in situ* using the appropriate ligand and [(Me_2_S)AuCl] or *ex situ* (see SI).

b Determined by ^1^H NMR spectroscopy using an internal standard.

c Contains 20% of 21.

d Contains 4% of 21.

e Using AgSbF_6_ instead of NaBAr_F_.

f Contains 48% of 21.

## Conclusions

In conclusion, we have developed a Pd-catalyzed phosphinylation of A1/A2-ditriflate-pillar[*n*]arene which provides access to a series of pillararene-based bisphosphine ligands. Adjustment of the stoichiometry of the reagents enabled the isolation of A1/A2-phosphine-triflate-pillar[5]arenes that can be engaged subsequently in (i) a distinct Pd-catalyzed phosphinylation reaction to obtain rim-differentiated bisphosphine ligands, or in (ii) a Pd-catalyzed Suzuki–Miyaura cross-coupling to afford A1/A2-aryl-phosphine-pillar[5]arene derivatives. The latter synthetic sequence was successfully applied to the literature-known A1/A2-ditriflate-pillar[6]arene precursor. The modularity of our synthetic plan led to the preparation of a collection of 17 mono- and bisphosphine ligands based on the pillarene scaffold. Study of the coordination chemistry of some of these derivatives using gold(i) precursors revealed that the macrocyclic structure maintains a certain degree of flexibility both in solution and in the solid state. The potential of the macromolecular phosphine derivatives to serve as effective ligands in catalysis was evaluated in benchmark gold-catalyzed intramolecular cycloisomerization of 1,6-enynes. We found that several structures achieved selectivity comparable to, or even superior to, those of a reference system. The modularity and ready accessibility of this ligand family, combined with the confining and readily functionalizable macrocyclic scaffold, make its immobilization on solid supports and the associated questions of catalyst recovery and recyclability attractive directions for future work. The origin of the high levels of selectivity obtained remains unclear and is the focus of ongoing studies in our laboratories.

## Author contributions

The manuscript was written through the contributions of all authors, and all authors have given approval to the final version.

## Conflicts of interest

There are no conflicts to declare.

## Note added

While this manuscript was being finalized, Xi, Ni, Liu and coworkers reported a Pd-catalyzed enantioselective Hirao-type C–P coupling of pillar[5]arene triflates with secondary phosphine oxides, giving direct access to inherently chiral phosphinopillarenes (*J. Am. Chem. Soc.*, 2026,  **148**, 24551–24562. https://doi.org/10.1021/jacs.6c09608).^43^ The two studies were conducted independently and concurrently. That work relies on a dynamic kinetic asymmetric transformation to access enantioenriched pillar[5]arene-based phosphine oxides, which are reduced to the corresponding P-ligands and applied in asymmetric catalysis. The present study relies on Pd-catalyzed phosphinylation of A1/A2-ditriflate-pillar[*n*]arenes to build a modular library of 17 racemic mono- and bisphosphine ligands on both pillar[5]- and pillar[6]arene scaffolds, and demonstrates their utility in selective Au(i) catalysis The simultaneous appearance of these contributions confirms the timeliness of pillar[*n*]arene-based phosphine ligands as an emerging research area.

## Supplementary Material

QO-013-D6QO00815A-s001

QO-013-D6QO00815A-s002

## Data Availability

The data supporting this article have been included as part of the supplementary information (SI). Supplementary information: experimental procedures, characterization of all new compounds, spectroscopic data and X-ray crystallographic data. See DOI: https://doi.org/10.1039/d6qo00815a. All structures disclosed in this study have been generated using CylView.^44^ CCDC 2553580–2553583 (9fa, 3b, 14a, 17, 15, 16) contain the supplementary crystallographic data for this paper.^45*a*–*f*^
