# Supplementary material for: Pillar[n]arene-based phosphine ligands: synthesis, coordination chemistry and application in selective Au(i) catalysis
Source: Org Chem Front. 2026 Jul 20. Online ahead of print. doi: 10.1039/d6qo00815a (PMC13403166; doi:10.1039/d6qo00815a)
Supplement: QO-OLF-D6QO00815A-s001 [file QO-OLF-D6QO00815A-s001.pdf]

Supplementary Information (SI) for Organic Chemistry Frontiers.
This journal is © the Partner Organisations 2026

Pillar[*n*]arene-based phosphine ligands: synthesis, coordination chemistry and application in selective Au(I) catalysis

Antoine Konter,[†a] Michele Buccio,[†a] Louis Vidal,[a] Céline Besnard[b] and Clément Mazet*[a]

[a] Department of Organic Chemistry, University of Geneva, 30 quai Ernest Ansermet, 1211 Geneva, Switzerland.

[b] Laboratory of Crystallography, University of Geneva, 24 quai Ernest Ansermet, 1211 Geneva, Switzerland.

[†] denotes equal contribution

clement.mazet@unige.ch

Contents

1. General information 3

2. Syntheses of secondary phosphine precursors 4

2.1 Secondary phosphines used in this study 4

2.2 Synthesis of bis(4-methoxyphenyl)phosphine borane (2a•BH_3) 5

2.3 Synthesis of bis(3,5-di-*tert*-butyl-4-methoxyphenyl)phosphine borane (2e•BH_3) 6

3. General Procedure for reaction optimization 9

4. Syntheses of A1/A2-diphosphine pillar[5]arenes 12

4.1 General Procedure I (GP-I) for Pd-catalyzed bis-phosphinylation 12

5. Syntheses of A1-phosphine-oxide-A2-triflate-pillar[5]arenes 18

5.1 General Procedure II (GP-II) for mono-phosphinylation 18

5.2 General Procedure III (GP-III) for oxidation 19

6. Syntheses of A1-phosphine-A2-aryl-pillar[5]arenes 29

6.1 General Procedure IV (GP-IV) for Suzuki-Miyaura cross-coupling 29

6.2 General Procedure V (GP-V) for phosphine oxide reduction 30

7. Synthesis of A1-(bis(3,5-bis(trifluoromethyl)phenyl)phosphine)-A2-(trifluoromethyl)phenyl-ethoxy-pillar[6]arene (13fa) 39

8. Syntheses of rim-differentiated A1/A2-di-phosphine-pillar[5]arenes 43

8.1 General Procedure VI (GP-VI) for sequential phosphinylation 43

10. Syntheses of gold complexes 49

11. Au(I)-catalyzed cycloisomerization of 1,6-enyne 61

11.1 General procedure 61

12. X-ray analyses 65

13. References 83

14. NMR spectra for new compounds 85

1. General information

Unless otherwise noted, all reactions were carried out under an inert atmosphere of nitrogen using either a two-manifold vacuum/inert gas lines or a M. Braun glovebox. Solvents were dried over activated alumina columns and further degassed by three successive "freeze-pump-thaw" cycles. Commercial reagents were purchased from ABCR, Fluka, Acros, Fluorochem or Strem and used without purification unless otherwise noted. Liquid reagents were transferred with stainless steel syringes or cannula. Thin layer chromatography (TLC) was performed on plates of silica precoated with 0.25 mm Kieselgel 60 F_{254} from Merck. Flash chromatography was performed using silica gel Kieselgel 60M (230-400 mesh) from Macherey-Nagel.

NMR spectra were acquired at the University of Geneva NMR platform (https://www.unige.ch/sciences/chiorg/nmr/) using a 500 MHz Avance III Bruker NMR spectrometer equipped with a helium-cooled cryogenic 5-mm DCH ^{13}C-^{1}H/D Bruker probe, a 400 MHz Avance III HD NanoBay spectrometer equipped with a N_2 prodigy cryogenic 5 mm CPP BB(F)-H-D probe or a 300 MHz Avance III, HD NanoBay spectrometer, equipped with a 5 mm PA BBO, BB(F)-H-D probe. ^{1}H NMR spectra were referenced to $CDCl_3$ (7.26 ppm) and $^{13}C\{^{1}H\}$ NMR spectra were referenced to $CDCl_3$ (77.16 ppm). $^{19}F\{^{1}H\}$ NMR chemical shifts are reported in ppm with absolute reference relative to ^{1}H. Microwave reactions were performed in a Biotage Initiator SW apparatus. HRMS data were obtained on a Xevo G2 Tof spectrometer (Ionization mode: ESI positive polarity; Mobile phases: MeOH 100 µL/min). Mass spectrum is calibrated using the MS lockspray system (LeuEnk calibration solution). Infrared spectra were obtained on a Perkin–Elmer 1650 FT-IR spectrometer using neat samples on a diamond ATR Golden Gate sampler. Melting points were recorded on a Büchi SMP-20 melting point apparatus using open glass capillaries.

Starting materials **1**, **6**, **11**, [(tetrahydrothiophen)Au(C_6F_5)], **18** and **22** were synthesized according to the literature.[1–7] The palladium precursor [(Pd.G_3)] was synthesized according to the literature.[7]

2. Syntheses of secondary phosphine precursors

2.1 Secondary phosphines used in this study

The secondary phosphines **2a**, **2f** and **2g** were synthesized according to the literature.[8] The spectroscopic data were in accordance with those reported in the literature.[8-9] The secondary phosphines **2b-d** were purchased from Strem and Fluorochem and used without further purification.

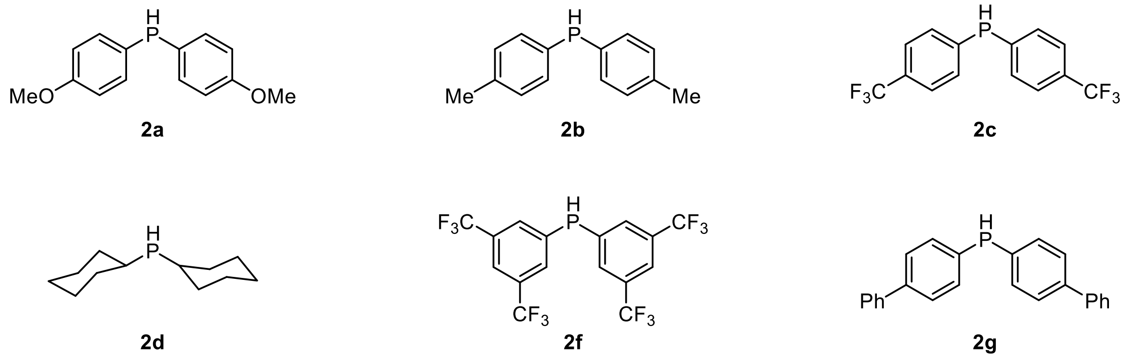

Figure S1. Secondary phosphines used in this study

2.2 Synthesis of bis(4-methoxyphenyl)phosphine borane (2a•BH_3)

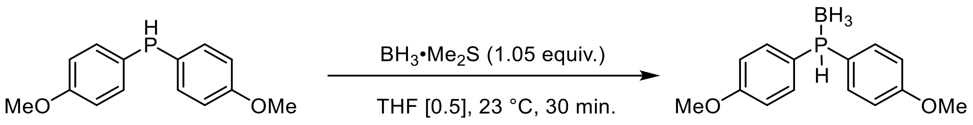

2a **2a•BH_3**

Figure S2. Synthesis of secondary phosphine borane **2a•BH_3**

In an oven dried Schlenk tube, **2a** (650 mg, 2.6 mmol, 1.0 equiv.) was dissolved in THF (5.3 mL). Next, BH_3•Me_2S (250 µL, 2.8 mmol, 1.05 equiv.) was added in one portion and the mixture was stirred at 23 °C. After 30 min., the reaction mixture was diluted with H_2O (20 mL). The aqueous phase was extracted with CH_2Cl_2 (3 × 30 mL) and the combined organic phases were dried over Na_2SO_4, filtered and concentrated under reduced pressure. The residue was purified by flash column chromatography over silica gel (eluent: *n*-pentane/Et_2O = 95:5) to afford **2a•BH_3** as a white solid (687 mg, 85% yield). The spectroscopic data were in accordance with those reported in the literature.[10]

2.3 Synthesis of bis(3,5-di-*tert*-butyl-4-methoxyphenyl)phosphine borane (2e•BH_3)

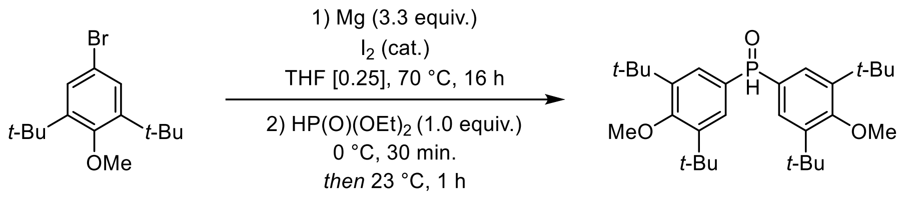

Figure S3. Synthesis of secondary phosphine oxide **2e•O**

Step 1: In an oven-dried three-necked round bottom flask equipped with an addition funnel, stirring bar and a condenser, Mg turnings (0.96 g, 38 mmol, 3.3 equiv.) were suspended in dry THF (10 mL), next a crystal of iodine was added, yielding an orange mixture. The suspension was heated to 70 °C until the mixture turned pale yellow. The addition funnel was loaded with a solution of 5-bromo-1,3-di-*tert*-butyl-2-methoxybenzene in dry THF (38 mL, 1.0 M, 3.3 equiv.). Next, 1 mL of the solution was added in one portion and the rest dropwise over 1 h. After addition was complete, the mixture was stirred at 70 °C. After 16 h, the flask was cooled to 0 °C with an ice/water bath. The addition funnel was loaded with a diethyl phosphite solution in dry THF (11.6 mL, 1.0 M, 1.0 equiv.), which was next added dropwise to the mixture over 30 min., after which the ice/water bath was removed. After 1 h, ^{31}P NMR analysis showed full consumption of diethyl phosphite. The flask was again cooled to 0 °C and after 15 min., an aqueous HCl solution (2.0 M, 40 mL) was loaded in the addition funnel and added dropwise. Once the addition was complete, the mixture was diluted with H_2O (50 mL) and extracted with EtOAc (3 × 50 mL). The combined organic phases were washed with a saturated aqueous NaCl solution (150 mL), dried over Na_2SO_4, filtered and concentrated under reduced pressure. The residue was purified by flash column chromatography over silica gel (*n*-pentane/EtOAc = 7:3) yielding **2e•O** as a white solid (5.1 g, 84% yield). The NMR data were in accordance with those reported in the literature.[11]

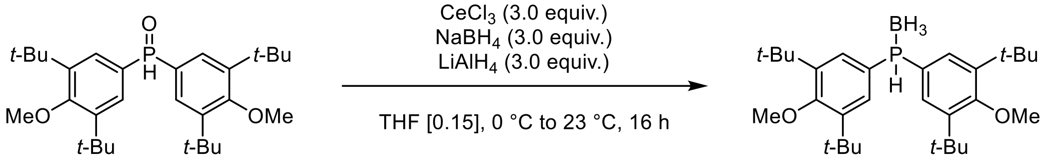

Figure S4. Synthesis of secondary phosphine borane **2e•BH$_3$**

Step 2: Following a modified literature procedure, in a three-necked flask equipped with a stirring bar, activated $CeCl_3$ (4.1 g, 17.0 mmol, 3.0 equiv.) was suspended in dry THF (37 mL).[12-13] After stirring vigorously for 15 min., the flask was cooled to 0 °C with an ice/water bath. After 30 min., $NaBH_4$ (630 mg, 16.6 mmol, 3.0 equiv.) was added in one portion and the resulting mixture stirred for 30 min.. Next, **2e•O** (2.7 g, 5.6 mmol, 1.0 equiv.) was added. After 30 min., $LiAlH_4$ (0.63 g, 17.0 mmol, 3.0 equiv.) was added in 10 portions over 1 h. The ice/water bath was removed and the mixture warmed to 23 °C. After 16 h, the reaction mixture was diluted with CH_2Cl_2 (50 mL), poured over a mixture of crushed ice and 37% HCl (4 mL) and stirred until no bubbling was observed. Next, the mixture was filtered through a pad of Celite, and the aqueous layer was extracted with CH_2Cl_2 (3 × 50 mL). The combined organic phases were dried over Na_2SO_4, filtered and concentrated under reduced pressure. The residue was purified by flash column chromatography over silica gel (*n*-pentane/Et_2O = 95:5) yielding **2e•BH$_3$** as a white solid (1.8 g, 65% yield).

Bis(3,5-di-*tert*-butyl-4-methoxyphenyl)phosphine borane (2e•BH$_3$)

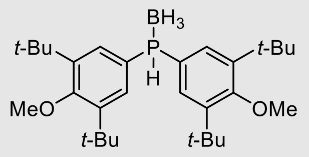

2e•BH3

TLC: 0.4, *n*-pentane/Et$_2$O = 95:5

^{1}H NMR (400 MHz, CDCl$_3$) δ (ppm) = 7.53 (d, $^2J_{P\text{-}H}$ = 12.2 Hz, 4H), 6.27 (dq, $^1J_{P\text{-}H}$ = 375.4 Hz, $J_{H\text{-}H}$ = 6.8 Hz, 1H), 3.72 (s, 6H), 1.42 (s, 36H). *The BH$_3$ signal was not observed due to the quadrupolar moment of ^{11}B.*

^{13}C{^{1}H} NMR (101 MHz, CDCl$_3$) δ (ppm) = 162.6 (d, $^4J_{C\text{-}P}$ = 2.8 Hz) (2 × C), 144.8 (d, $^3J_{C\text{-}P}$= 10.4 Hz) (2 × C), 131.6 (d, $^2J_{C\text{-}P}$ = 11 Hz) (4 × CH) , 119.5 (d, $^1J_{C\text{-}P}$ = 60.2 Hz) (2 × C), 64.5 (2 × CH$_3$), 36.0 (4 × C), 31.9 (12 × CH$_3$).

^{31}P{^{1}H} NMR (162 MHz, CDCl$_3$) δ (ppm) = –1.36 (bs).

HRMS (ESI$^+$) *m/z*: calculated for $C_{30}H_{48}O_2P$ [M-BH$_3$+H]$^+$: 471.3392; found: 471.3397.

IR (neat) ν (cm^{-1}): 2961, 2378, 1408, 1225, 1147, 1116, 1060.

Melting point: 141-144 °C.

3. General Procedure for reaction optimization

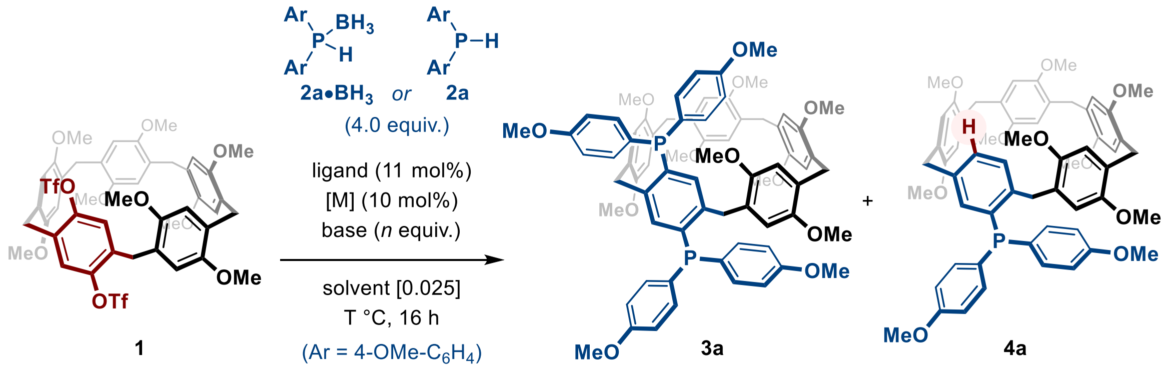

In a glovebox, in an oven-dried J-Young flask, the appropriate metal precursor noted [M] (5 μmol, 10 mol%) and the appropriate ligand (5.5 μmol,11 mol%) were dissolved in the selected solvent (1 mL). After 10 min., **1** (0.025 mmol, 1.0 equiv.), **2a** or **2a•BH$_3$** (0.1 mmol, 4.0 equiv.) and the selected base (0.1 mmol, 4.0 equiv.) were added in sequence. The J-Young flask was closed, taken out of the glovebox and placed in a preheated oil bath and stirred at the desired temperature. After 16 h, the reaction mixture was cooled to 23 °C and diluted with a saturated aqueous NaCl solution (10 mL). The aqueous layer was extracted with EtOAc (3 × 10 mL), and the combined organic phases were washed with an aqueous LiCl solution (4 × 15 mL, 5% w/w), dried over Na_2SO_4, filtered and concentrated under reduced pressure. Triethyl phosphate and fluorobenzene were added as internal standard before NMR analyses.

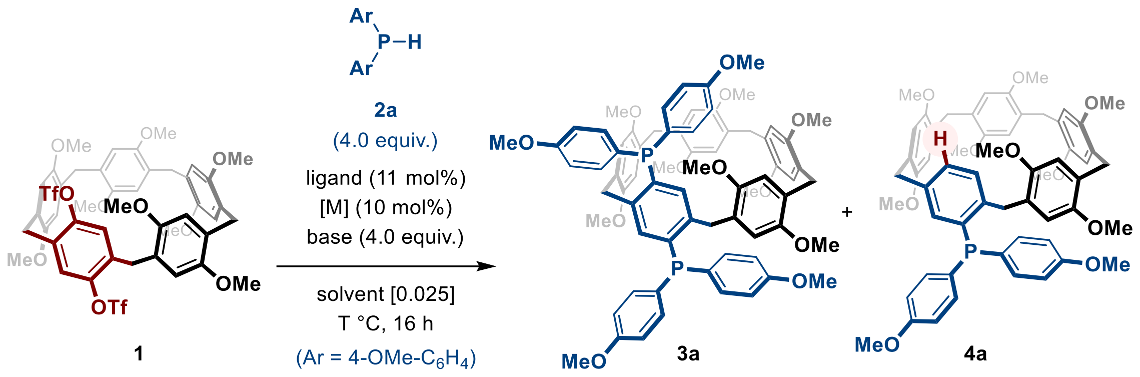

entry	[M]	ligand	base	solvent	T (°C)	**3a** (%)[a]	**4a** (%)[a]
1	$(Ph_3P)_4Pd$	**L1**	DMAP	1,4-dioxane	80	<1	<1
2	$Pd_2(dba)_3$	**L2**	CsF	THF	80	<1	<1
3	$(Ph_3P)_2PdCl_2$	-	$(i\text{Pr})_2NEt$	DMF	120	<1	<1
4	(IPr)Pd(allyl)Cl	-	K_2CO_3	THF	80	<1	<1
5	$Pd(OAc)_2$	**L3**	DABCO	THF	80	<1	<1
6	$(dppe)NiCl_2$	-	DABCO	DMF	100	<1	<1
7[b]	$Pd(OAc)_2$	**L4**	DABCO	DMF:THF (1:1)	140[c]	20	<1
8[b]	$Pd(OAc)_2$	**L4**	DABCO	DMF:THF (1:1)	150[c]	30	<1
9[b]	$Pd(OAc)_2$	**L4**	Et_3N	DMF:THF (1:1)	150[c]	50	<1

Table S1. Reactions conditions: **1** (0.025 mmol), **2a** (0.1 mmol), [M] (10 mol%), ligand (11 mol%) in 1 mL of solvent. [a] Determined by ^1H NMR analysis of the crude reaction mixture using an internal standard. [b] 1 h. [c] Microwave heating, normal absorption level.

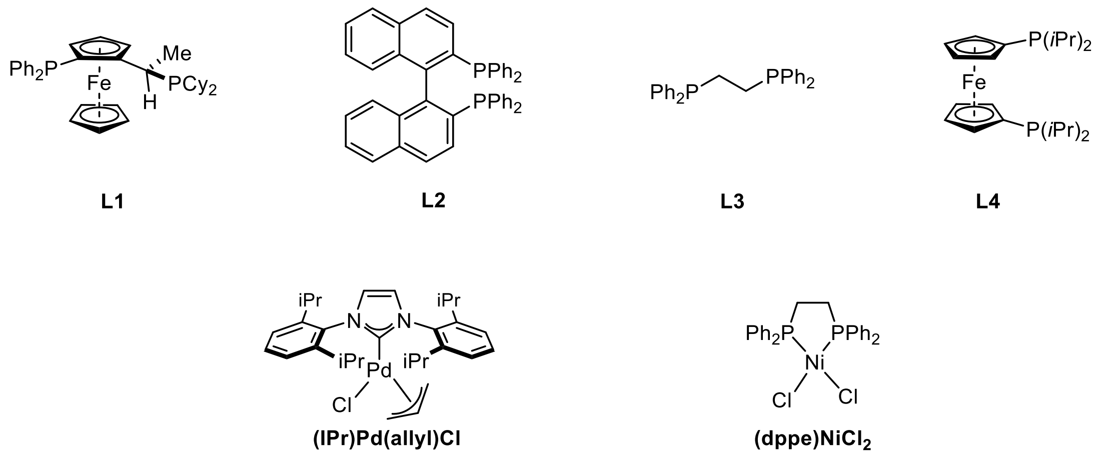

Figure S5. Ligands and precatalysts evaluated

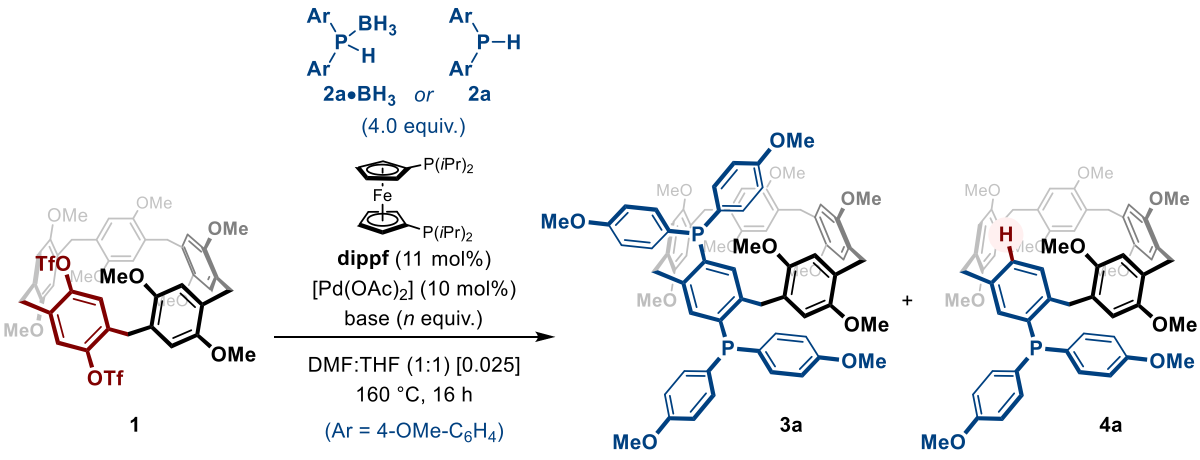

entry	phosphine	base (*n* equiv.)	**3a** (%)[a]	**4a** (%)[a]
1	**2a•BH₃**	Et_3N (8.0)	56	14
2	**2a•BH₃**	Et_3N (12.0)	59	14
3	**2a•BH₃**	DBU (12.0)	<1	<1
4	**2a•BH₃**	DMAP (12.0)	23	9
5	**2a•BH₃**	DABCO (12.0)	83	<1
6	**2a•BH₃**	DABCO (8.0)	89	<1
7	**2a•BH₃**	DABCO (20.0)	81	<1
8[b]	**2a•BH₃**	DABCO (12.0)	73	<1
9	**2a**	DABCO (4.0)	90	<1
10	**2a**	BTMG (4.0)[c]	88	<1

Table S2. Reaction conditions: **1** (0.05 mmol), **2a•BH₃** or **2a** (0.2 mmol), [Pd(OAc)₂] (10 mol%), dippf (11 mol%). [a] Determined by ^{1}H NMR analysis of the crude reaction mixture using an internal standard. [b] Reaction performed in DMF. [c] BTMG: Barton's base (2-*tert*-butyl-1,1,3,3-tetramethylguanidine).

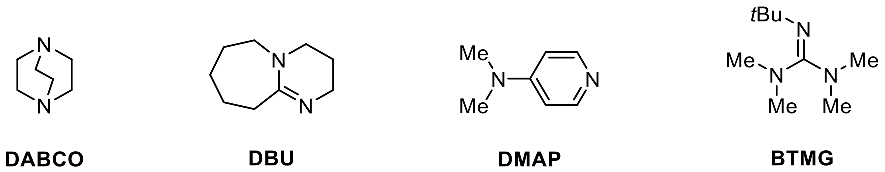

Figure S6. Bases screened

4. Syntheses of A1/A2-diphosphine pillar[5]arenes

4.1 General Procedure I (GP-I) for Pd-catalyzed bis-phosphinylation

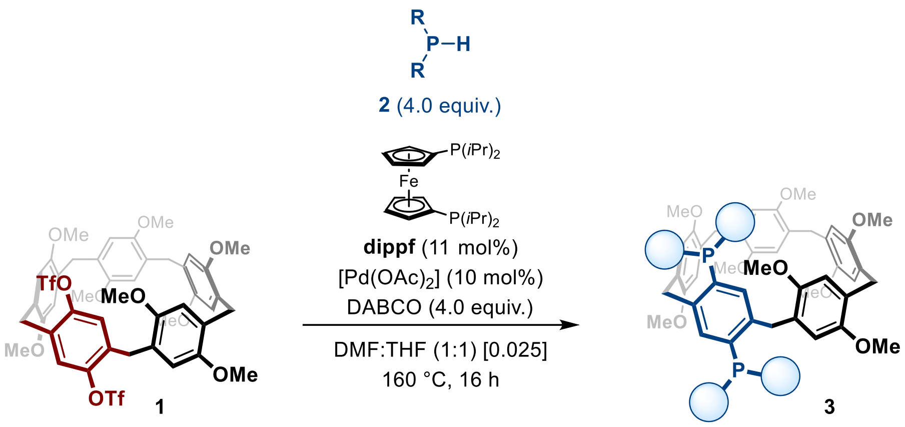

In a glovebox, in an oven-dried J-Young flask, $[Pd(OAc)_2]$ (2.3 mg, 10 µmol, 10 mol%), dippf (4.8 mg, 11 µmol, 11 mol%), **1** (100 mg, 0.1 mmol, 1.0 equiv.) and the appropriate secondary phosphine **2** (0.41 mmol, 4.0 equiv.) were dissolved in a 1:1 mixture of DMF:THF (4 mL), next DABCO (46 mg, 0.41 mmol, 4.0 equiv.) was added. The J-Young flask was closed, taken out of the glovebox and placed in a preheated oil bath and stirred at 160 °C. After 16 h, the reaction mixture was cooled to 23 °C and diluted with saturated aqueous NaCl solution (20 mL). The aqueous layer was extracted with EtOAc (3 × 15 mL) and the combined organic phases were washed with an aqueous LiCl solution (4 × 20 mL, 5% w/w), dried over Na_2SO_4, filtered and concentrated under reduced pressure. The residue was purified by flash column chromatography over silica gel using *n*-pentane/CH_2Cl_2/Et_2O as eluent to afford A1/A2-diphosphine-pillar[5]arene **3**.

A1/A2-(bis(4-methoxyphenyl)phosphine)-pillar[5]arene (3a)

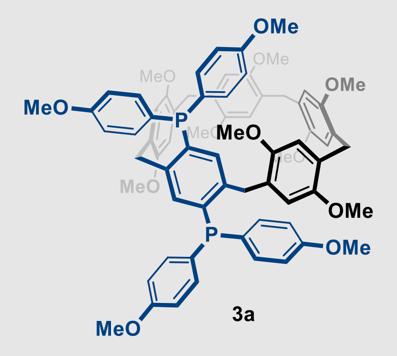

Synthesized following General Procedure I using **1** (0.51 mmol, 1.0 equiv.) and **2a** (2.0 mmol, 4.0 equiv.). Purification by flash column chromatography over silica gel (eluent: *n*-pentane/CH_2Cl_2/Et_2O = 5:2:1) afforded **3a** as a white solid. (448 mg, 75% yield).

TLC: 0.35, *n*-pentane/CH_2Cl_2/Et_2O = 5:2:1

^{1}H NMR (400 MHz, $CDCl_3$) δ (ppm) = 7.23 (dd, $J_{H\text{-}H}$ = 8.7 Hz, $J_{P\text{-}H}$ = 7.1 Hz, 4H), 7.02 (t, $J_{P\text{-}H}$ = 4.9 Hz, 2H), 6.98 (dd, $J_{H\text{-}H}$ = 8.7 Hz, $J_{P\text{-}H}$ = 7.1 Hz, 4H), 6.88 (dd, $J_{H\text{-}H}$ = 8.7 Hz, $J_{P\text{-}H}$ = 0.8 Hz, 4H), 6.86 (s, 4H), 6.77 (s, 2H), 6.73 (dd, $J_{H\text{-}H}$ = 8.7 Hz, $J_{P\text{-}H}$ = 0.8 Hz, 4H), 6.59 (s, 2H), 4.16 (dd, $J_{H\text{-}H}$ = 13.6 Hz, $J_{P\text{-}H}$ = 3.5 Hz, 2H), 3.85 (s, 2H), 3.83 (s, 12H), 3.79 (s, 2H), 3.76 (s, 2H), 3.72 (s, 6H), 3.65 (s, 2H), 3.63 (s, 6H), 3.35 (s, 6H), 3.25 (s, 6H).

^{13}C{^{1}H} NMR (101 MHz, $CDCl_3$) δ (ppm) = 160.2 (C), 159.9 (C), 151.1 (C), 151.1 (C), 150.7 (C), 150.6 (C), 143.9 (C), 143.6 (C), 136.4 (C), 136.2 (C), 135.7 (CH), 135.6 (CH), 135.6 (CH), 135.5 (CH), 135.1 (CH), 134.9 (CH) 129.6 (C), 129.5 (C), 128.6 (C), 128.4 (C), 128.4 (C), 128.4 (C), 128.4 (C), 128.3 (C), 114.5 (CH), 114.3 (CH), 114.2 (CH), 114,2 (CH), 114,1 (CH), 114.1 (CH), 113.6 (CH), 56.7 (CH_3), 55.9 (CH_3), 55.5 (CH_3), 55.4 (CH_3), 55.3 (CH_3), 55.2 (CH_3), 33.5 (CH_2), 33.4 (CH_2), 31.7 (CH_2), 30.2 (CH_2), 30.1 (CH_2), 22.8 (CH_2).

^{31}P{^{1}H} NMR (162 MHz, $CDCl_3$) δ (ppm) = –16.0.

HRMS (ESI$^+$) *m/z*: calculated for $C_{71}H_{73}O_{12}P_2$ $[M+H]^+$: 1179.4573; found: 1179.4579.

IR (neat) ν (cm^{-1}): 2929, 2830, 1592, 1497, 1209, 1175, 1045.

Melting point: 98-100 °C.

A1/A2-(bis(4-methylphenyl)phosphine)-pillar[5]arene (3b)

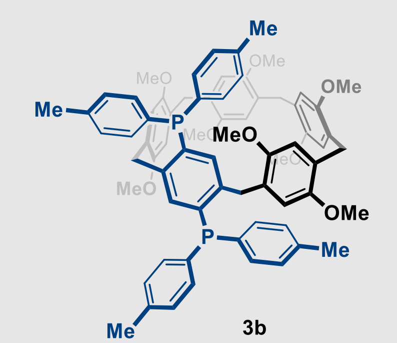

3b

Synthesized following General Procedure I using **1** (0.08 mmol, 1.0 equiv.) and **2b** (0.32 mmol, 4.0 equiv.). Purification by flash column chromatography over silica gel (eluent: *n*-pentane/CH_2Cl_2/Et_2O = 3:1:1) afforded **3b** as a white solid. (65 mg, 72% yield).

Crystals suitable for X-ray diffraction were grown by diffusion of hexane in a concentrated CH_2Cl_2 solution.

TLC: 0.35, *n*-pentane/CH_2Cl_2/Et_2O = 3:1:1

^{1}H NMR (400 MHz, $CDCl_3$) δ (ppm) = 7.22 – 7.12 (m, 8H), 7.04 (t, $J_{P\text{-}H}$ = 4.8 Hz, 2H), 7.01 – 6.93 (m, 8H), 6.86 (s, 4H), 6.79 (s, 2H), 6.59 (s, 2H), 4.19 (dd, $J_{H\text{-}H}$ = 13.6 Hz, $J_{P\text{-}H}$ = 3.4 Hz, 2H), 3.89 – 3.72 (m, 6H), 3.70 (s, 6H), 3.68 (s, 2H), 3.65 (s, 6H), 3.34 (s, 6H), 3.22 (s, 6H), 2.38 (s, 6H), 2.36 (s, 6H).

^{13}C{^{1}H} NMR (101 MHz, $CDCl_3$) δ (ppm) = 151.2 (C), 151.1 (C), 150.7 (C), 150.5 (C), 144.2 (C), 143.9 (C), 138.5 (C), 137.8 (C), 136.1 (C), 135.9 (C), 135.9 (CH), 135.8 (CH), 135.1 (C), 135.0 (C), 134.5 (CH), 134.3 (CH), 134.0 (C), 133.9 (C), 133.8 (CH), 133.6 (CH), 129.4 (CH), 129.3 (CH), 129.2 (CH), 129.1 (CH), 128.6 (C), 128.5 (C), 128.2 (C), 114.8 (CH), 114.2, (CH) 114.1 (CH), 114.0 (CH), 113.6 (CH), 56.5 (CH_3), 56.0 (CH_3), 55.4 (CH_3), 55.4 (CH_3), 33.5 (CH_2), 33.3 (CH_2), 30.3 (CH_2), 30.1 (CH_2), 21.5 (CH_3), 21.4 (CH_3).

^{31}P{^{1}H} NMR (162 MHz, $CDCl_3$) δ (ppm) = –14.49.

HRMS (ESI$^+$) *m/z*: calculated for $C_{71}H_{73}O_8P_2$ $[M+H]^+$: 1115.5; found: 1115.9.

IR (neat) ν (cm^{-1}): 2931, 2827, 1497, 1397, 1209, 1045.

Melting point: 100-103 °C.

A1/A2-di-cyclohexyl-phosphine-pillar[5]arene (3d)

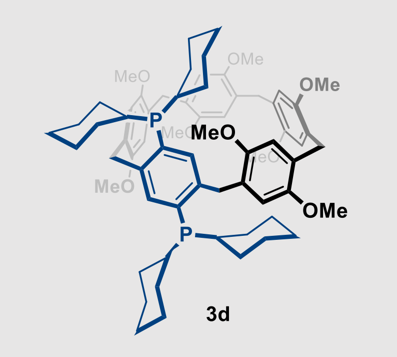

Synthesized following General Procedure I using **1** (0.53 mmol, 1.0 equiv.) and **2d** (2.2 mmol, 4.0 equiv.) and BTMG (Barton's base) (2.2 mmol, 4.0 equiv.) instead of DABCO. Purification by flash column chromatography over silica gel (*n*-pentane/CH_2Cl_2/Et_2O = 20:1:1) afforded **3d** as a white solid. (318 mg, 55% yield).

TLC: 0.4, *n*-pentane/CH_2Cl_2/Et_2O = 20:1:1

^{1}H NMR (400 MHz, $CDCl_3$) δ (ppm) = 7.38 – 7.32 (dd, $J_{P\text{-}H}$ = 4.6, $J_{H\text{-}H}$ = 3.5 Hz, 2H), 6.82 (s, 2H), 6.81 (s, 2H), 6.74 (s, 2H), 6.65 (s, 2H), 4.63 (dd, $J_{H\text{-}H}$ = 13.5 Hz, $J_{P\text{-}H}$ = 5.1 Hz, 2H), 3.86 – 3.77 (m, 6H), 3.72 (s, 2H), 3.67 (s, 6H), 3.66 (s, 6H), 3.62 (s, 6H), 3.44 (s, 6H), 2.06 – 1.54 (m, 16H), 1.46 – 1.08 (m, 18H), 1.07 – 0.62 (m, 10H).

^{13}C{^{1}H} NMR (101 MHz, $CDCl_3$) δ (ppm) = 150.9 (C), 150.8 (C), 150.7 (C), 150.6 (C), 145.1 (C), 144.8 (C), 134.5 (CH), 134.5 (CH), 134.5 (CH), 133.3 (C), 133.1 (C), 129.3 (C), 128.5 (C), 128.4 (C), 128.2 (C), 114.9 (CH), 114.8 (CH), 113.9 (CH), 113.8 (CH), 56.01 (CH_3), 55.9 (CH_3), 55.9 (CH_3), 55.8 (CH_3), 36.0 (CH), 35.9 (CH), 33.8 (CH_2), 33.6 (CH_2), 31.9 (CH), 31.8 (CH), 31.3 (CH_2), 31.1 (CH_2), 30.3 (CH_2), 30.2 (CH_2), 30.0 (CH_2), 30.0 (CH_2), 29.8 (CH_2), 27.8 (CH_2), 27.7 (CH_2), 27.4 (CH_2), 27.4 (CH_2), 27.2 (CH_2), 27.1 (CH_2), 26.9 (CH_2), 26.7 (CH_2), 26.6 (CH_2).

^{31}P{^{1}H} NMR (162 MHz, $CDCl_3$) δ (ppm) = –14.01.

HRMS (ESI$^+$) *m/z*: calculated for $C_{67}H_{89}O_8P_2$ $[M+H]^+$: 1083.6033; found: 1083.6034.

IR (neat) ν (cm^{-1}): 2919, 2846, 1500, 1446, 1397, 1208, 1175, 1045.

Melting point: 126-128 °C.

Attempted synthesis of A1/A2-(bis(4-trifluoromethyl)phenyl)phosphine-pillar[5]arene (3c)

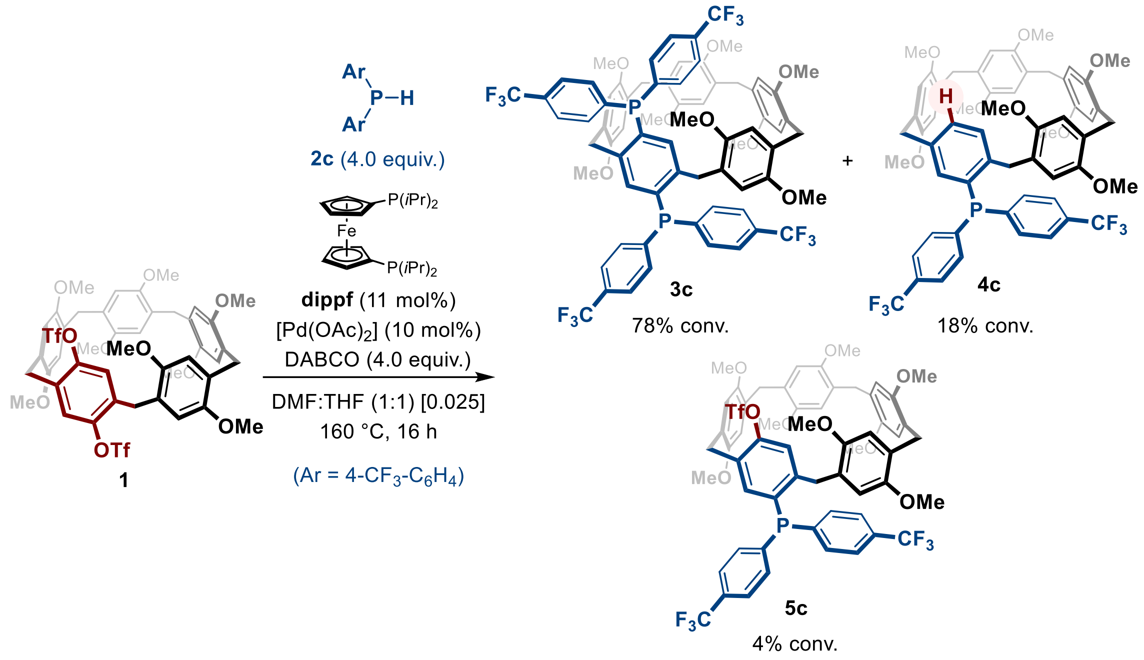

Figure S7. Attempted synthesis of **3c**

Synthesized following General Procedure I, using **1** (0.1 mmol, 1.0 equiv.) and **2c** (0.4 mmol, 4.0 equiv.). Purification by flash column chromatography over silica gel (eluent: *n*-pentane/CH_2Cl_2 = 97:3) afforded **3c** and **5c** (3.7:1) as an inseparable mixture.

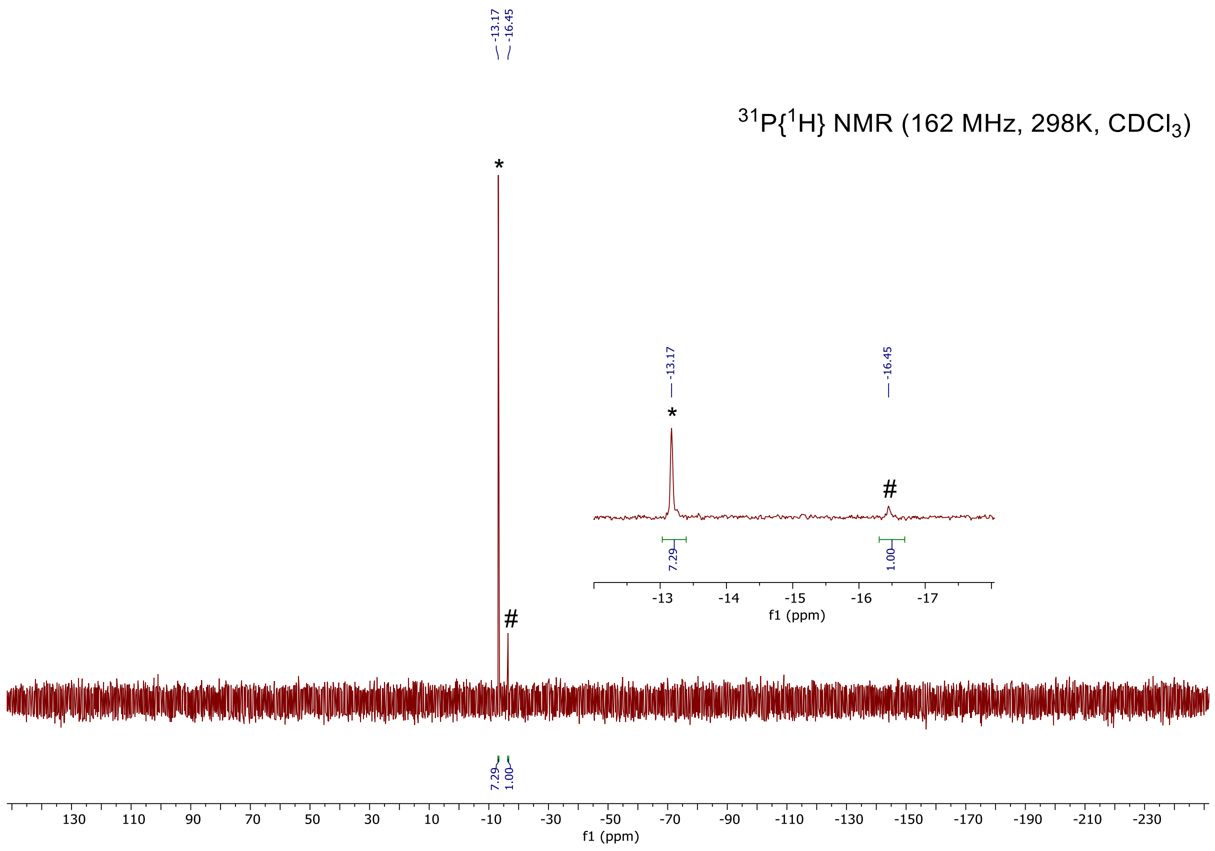

Figure S8. $^{31}P\{^1H\}$ NMR (162 MHz, $CDCl_3$) of the mixture of **3c** (*) and **5c** (#)

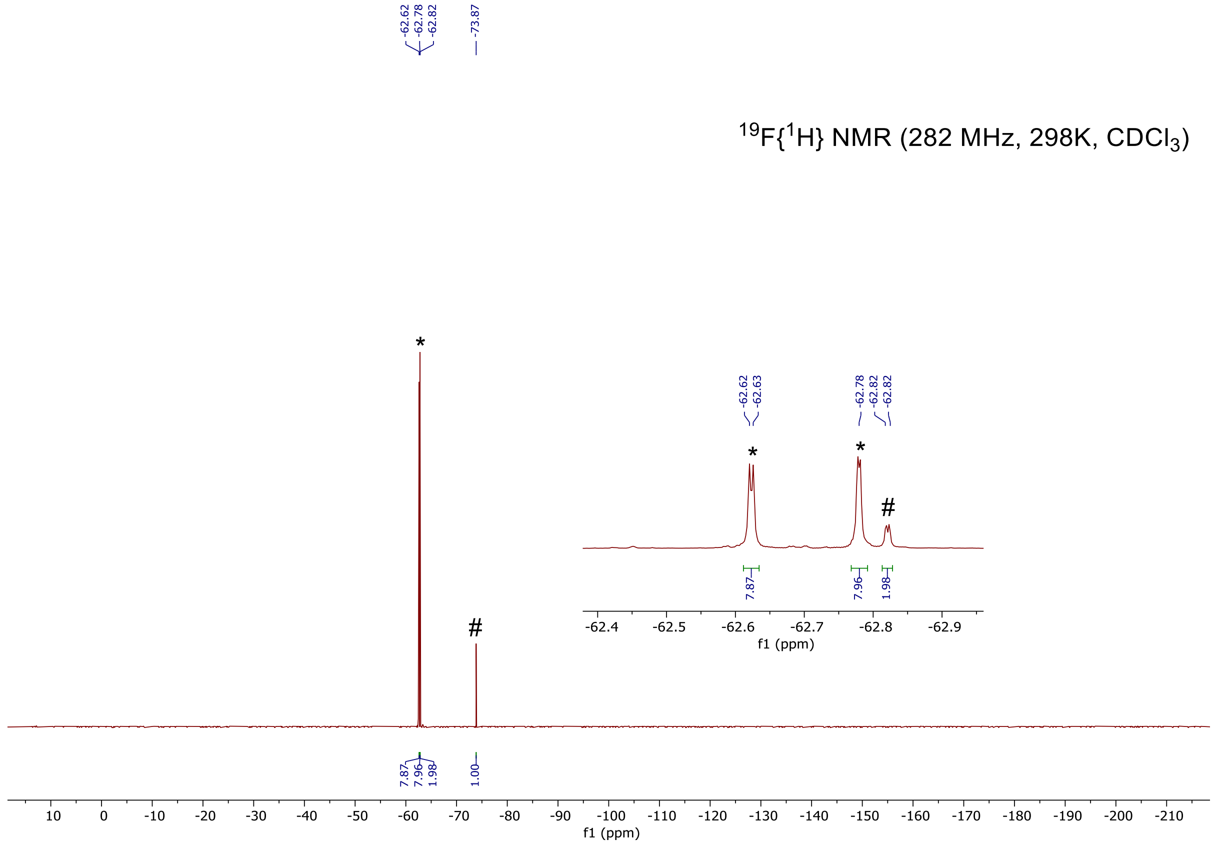

Figure S9. $^{19}F\{^1H\}$ NMR (282 MHz, $CDCl_3$) of the mixture of **3c** (*) and **5c** (#)

5. Syntheses of A1-phosphine-oxide-A2-triflate-pillar[5]arenes

5.1 General Procedure II (GP-II) for mono-phosphinylation

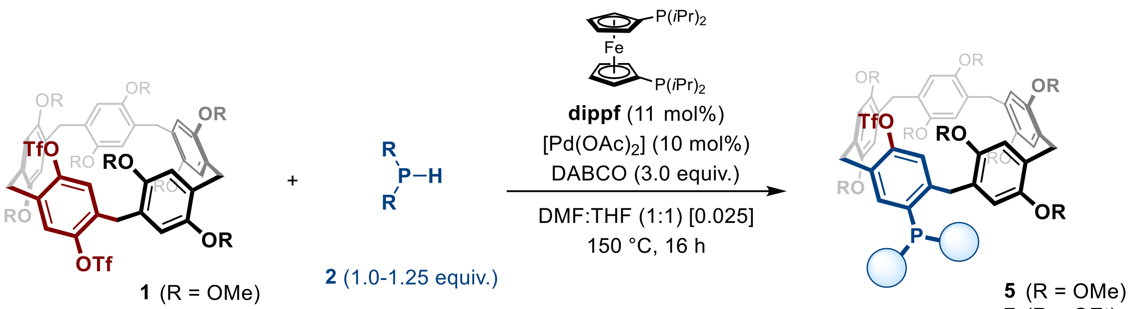

1 (R = OMe)
6 (R = OEt)

2 (1.0-1.25 equiv.)

5 (R = OMe)
7 (R = OEt)

In a glovebox, in a J-Young flask, [Pd(OAc)$_2$] (2.3 mg, 10 μmol, 10 mol%), dippf (4.8 mg, μmol, 11 mol%), the desired A1/A2-ditriflate-pillar[5]arene **1** or **6** (0.1 mmol, 1.0 equiv.) and the appropriate secondary phosphine **2** (0.1-0.12 mmol, 1.0-1.25 equiv.) were dissolved in a 1:1 mixture of DMF:THF (4 mL). Next, DABCO (34 mg, 0.3 mmol, 3.0 equiv.) was added and the J-Young flask was closed, taken out of the glovebox, placed in a preheated oil bath and stirred at 150 °C. After 16 h, the reaction mixture was cooled to 23 °C and diluted with a saturated aqueous NaCl solution (30 mL). The aqueous layer was extracted with EtOAc (3 × 20 mL) and the combined organic phases were washed with an aqueous LiCl solution (5 × 20 mL, 5% w/w), dried over Na_2SO_4, filtered and concentrated under reduced pressure. The residue was purified by flash column chromatography over silica gel using *n*-pentane/CH_2Cl_2/Et_2O as eluent to afford A1-phosphine-A2-triflate-pillar[5]arene **5** or **7**.

5.2 General Procedure III (GP-III) for oxidation

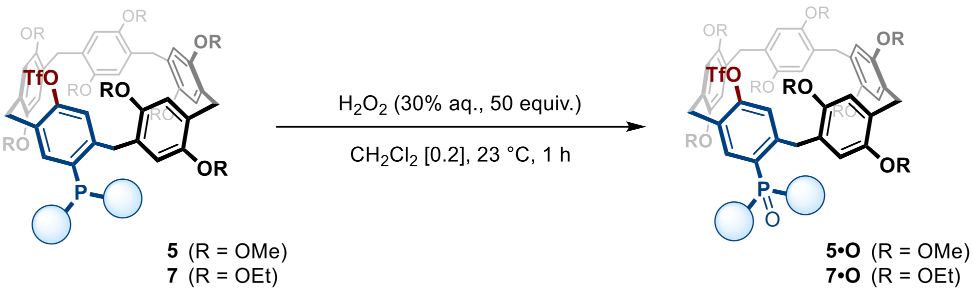

The desired A1-phosphine-A2-triflate-pillar[5]arene **5** or **7** (0.05 mmol, 1.0 equiv.) was dissolved in CH_2Cl_2 (0.3 mL), and H_2O_2 (0.25 mL, 30% aq., 50 equiv.) was added. After stirring for 1 h, ^{31}P NMR analysis indicated full conversion to the corresponding phosphine oxide. The reaction mixture was diluted with H_2O (10 mL), and the aqueous layer was extracted with CH_2Cl_2 (3 × 15 mL). The combined organic phases were dried over Na_2SO_4, filtered and concentrated under reduced pressure to afford the corresponding A1-phosphine-oxide-A2-triflate-pillar[5]arene **5•O** or **7•O**.

A1-(bis(4-methoxyphenyl)phosphine)-A2-triflate-pillar[5]arene (5a)

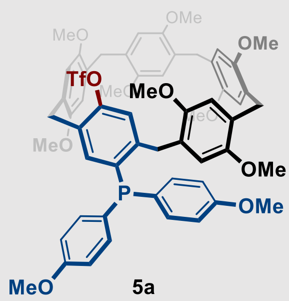

5a

Synthesized following General Procedure II using **1** (0.71 mmol, 1.0 equiv.) and **2a** (0.88 mmol, 1.0 equiv.). Purification by flash column chromatography over silica gel (eluent: *n*-pentane/CH_2Cl_2/Et_2O = 6:1:1) afforded **5a** as a white solid. (392 mg, 51% yield).

TLC: 0.3, *n*-pentane/CH_2Cl_2/Et_2O = 6:1:1

^{1}H NMR (400 MHz, $CDCl_3$) δ (ppm) = 7.21 (d, $J_{P\text{-}H}$ = 3.8 Hz, 1H), 7.16 (dd, $J_{H\text{-}H}$ = 8.7, $J_{P\text{-}H}$ = 7.3 Hz, 4H), 6.93 (d, $J_{P\text{-}H}$ = 3.9 Hz, 1H), 6.87 (d, $J_{H\text{-}H}$ = 8.1 Hz, 4H), 6.83 (s, 1H), 6.82 (s, 1H), 6.79 (s, 1H), 6.78 (s, 2H), 6.71 (s, 1H), 6.62 (s, 2H), 4.07 (s, 2H), 3.82 (s, 6H), 3.81 – 3.75 (m, 6H), 3.67 (s, 15H), 3.66 (s, 2H), 3.65 (s, 3H), 3.47 (s, 3H), 3.34 (s, 3H).

^{13}C{^{1}H} NMR (101 MHz, $CDCl_3$) δ (ppm) = 160.4 (C), 151.2 (C), 151.0 (C), 150.9 (C), 150.9 (C), 150.7 (C), 150.6 (C), 150.2 (C), 149.6 (C), 147.0 (C), 146.8 (C), 137.5 (CH), 135.7 (bs, CH), 135.6 (C), 135.5 (bs, CH), 135.4 (C), 130.4 (C), 129.4 (C), 128.7 (C), 128.4 (C), 128.4 (C), 128.4 (C), 127.9 (C), 127.9 (C), 127.3 (C), 124.9 (C), 122.1 (CH), 122.1 (CH), 121.9 ($J_{C\text{-}F}$ = 321.0 Hz) (C), 114.6 (CH), 114.5 (CH), 114.4 (CH), 114.3 (CH), 114.2 (CH), 114.1 (CH), 113.8 (CH), 113.1 (CH), 56.2 (CH_3), 55.9 (CH_3), 55.8 (CH_3), 55.7 (CH_3), 55.7 (CH_3), 55.5 (CH_3), 55.4 (CH_3), 55.3 (CH_3), 55.2 (CH_3), 32.8 (CH_2), 32.6 (CH_2), 32.2 (CH_2), 30.6 (CH_2), 29.5 (CH_2), 29.4 (CH_2).

^{31}P{^{1}H} NMR (162 MHz, $CDCl_3$) δ (ppm) = –17.89.

^{19}F{^{1}H} NMR (282 MHz, $CDCl_3$) δ (ppm) = –73.80.

HRMS (ESI$^+$) *m/z*: calculated for $C_{58}H_{59}F_3O_{13}PS$ $[M+H]^+$: 1083.3360; found: 1083.3335.

IR (neat) ν (cm^{-1}): 2935, 2830, 1501, 1400, 1208, 1175, 1043.

Melting point: 112-115 °C.

A1-(bis(4-methoxyphenyl)phosphine-oxide)-A2-triflate-pillar[5]arene (5a•O)

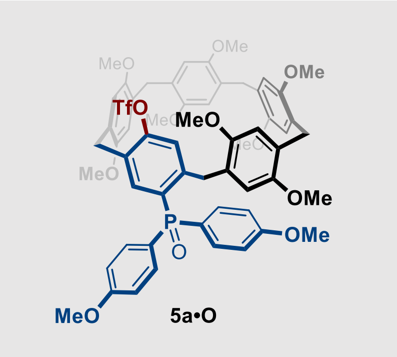

Synthesized following General Procedure III using **5a** (0.19 mmol, 1.0 equiv.). **5a•O** was obtained as a white solid. (205 mg, quantitative).

^{1}H NMR (400 MHz, $CDCl_3$) δ (ppm) = 7.56 (dd, $J_{P\text{-}H}$ = 11.5, $J_{H\text{-}H}$ = 8.8 Hz, 4H), 7.40 (d, $J_{P\text{-}H}$ = 3.2 Hz, 1H), 7.10 (d, $J_{P\text{-}H}$ = 13.8 Hz, 1H), 7.03 (s, 1H), 6.99 (dd, $J_{H\text{-}H}$ = 8.8, $J_{P\text{-}H}$ = 2.2 Hz, 4H), 6.92 (s, 1H), 6.89 (s, 1H), 6.83 (d, $J_{P\text{-}H}$ = 1.2 Hz, 2H), 6.80 (s, 1H), 6.78 (s, 1H), 6.62 (s, 1H), 4.17 (bs, 2H), 3.86 (s, 6H), 3.81 (d, $J_{H\text{-}H}$ = 5.2 Hz, 4H), 3.75 (m, 8H), 3.73 (s, 3H), 3.71 (s, 3H), 3.70 (s, 3H), 3.68 (s, 2H), 3.65 (s, 6H), 3.41 (s, 3H).

^{13}C{^{1}H} NMR (101 MHz, $CDCl_3$) δ (ppm) = 162.4 (C), 162.4 (C), 151.5 (C), 151.5 (C), 151.2 (C), 150.9 (C), 150.9 (C), 150.8 (C), 150.6 (C), 150.4 (C), 150.3 (C), 149.6 (C), 148.8 (C), 148.7 (C), 137.7 (CH), 137.6 (CH), 133.9 (CH), 133.8 (CH), 130.7 (C), 129.8 (C), 129.7 (C), 129.4 (C), 129.3 (C), 128.2 (C), 128.2 (C), 128.1 (C), 127.6 (C), 126.8 (C), 125.3 (C), 124.2 (C), 123.8 (C), 123.7 (bs, CH), 118.5 (q, $J_{C\text{-}F}$ = 320.8 Hz) (C), 114.5 (CH), 114.4 (CH), 114.2 (CH), 114.1 (CH), 114.1 (CH), 114.0 (CH), 114.0 (CH), 113.4 (CH), 112.9 (CH), 56.1 (CH_3), 55.6 (CH_3), 55.6 (CH_3), 55.5 (CH_3), 55.5 (CH_3), 55.4 (CH_3), 55.3 (CH_3), 55.0 (CH_3), 32.7 (CH_2), 32.1 (CH_2), 32.1 (CH_2), 31.1 (CH_2), 29.1 (CH_2), 28.8 (CH_2).

^{31}P{^{1}H} NMR (162 MHz, $CDCl_3$) δ (ppm) = 30.31.

^{19}F{^{1}H} NMR (282 MHz, $CDCl_3$) δ (ppm) = –73.79.

HRMS (ESI$^+$) *m/z*: calculated for $C_{58}H_{59}F_3O_{14}PS$ $[M+H]^+$: 1099.3311; found: 1099.3318.

IR (neat) ν (cm^{-1}): 2936, 2829, 1500, 1399, 1208, 1174, 1043.

Melting point: 135-137 °C.

A1-(dicyclohexylphosphine)-A2-triflate-pillar[5]arene (5d)

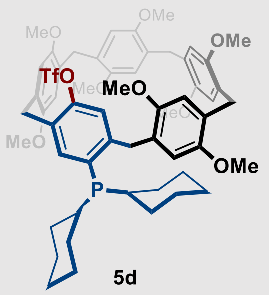

5d

Synthesized following General Procedure II using **1** (0.61 mmol, 1.0 equiv.) and **2d** (0.75 mmol, 1.25 equiv.). Purification by flash column chromatography over silica gel (eluent: *n*-pentane/CH_2Cl_2/Et_2O = 10:1:1) afforded **5d** as a white solid. (503 mg, 80% yield).

TLC: 0.3, *n*-pentane/CH_2Cl_2/Et_2O = 10:1:1

^{1}H NMR (400 MHz, $CDCl_3$) δ (ppm) = 7.39 (d, $J_{P\text{-}H}$ = 1.8 Hz, 1H), 7.14 (d, $J_{P\text{-}H}$ = 3.3 Hz, 1H), 6.84 (s, 1H), 6.82 (s, 1H), 6.80 (s, 1H), 6.77 (s, 2H), 6.75 (s, 1H), 6.74 (s, 1H), 6.69 (s, 1H), 3.88 – 3.72 (m, 10H), 3.70 (s, 3H), 3.66 (s, 6H), 3.66 (s, 3H), 3.65 (s, 6H), 3.64 (s, 3H), 3.61 (s, 3H), 1.99 – 1.57 (m, 10H), 1.52 – 1.07 (m, 10H), 1.01 – 0.82 (m, 2H).

^{13}C{^{1}H} NMR (101 MHz, $CDCl_3$) δ (ppm) = 151.1 (C), 151.0 (C), 150.9 (C), 150.8 (C), 150.7 (C), 150.6 (C), 150.3 (C), 149.7 (C), 149.4 (C), 149.4 (C), 136.3 (CH), 136.2 (CH), 132.9 (C), 132.7 (C), 129.6 (C), 129.6 (C), 128.8 (C), 128.4 (C), 128.3 (C), 128.2 (C), 128.0 (C), 125.1 (C), 122.2 (CH), 122.2 (CH), 118.7 (q, $J_{C\text{-}F}$ = 320.5 Hz) (C), 114.4 (CH), 114.4 (CH), 114.2 (CH), 114.2 (CH), 114.1 (CH), 114.0 (CH), 113.6 (CH), 113.5 (CH), 56.0 (CH_3), 55.8 (CH_3), 55.8 (CH_3), 55.8 (CH_3), 55.8 (CH_3), 55.7 (CH_3), 55.6 (CH_3), 55.4 (CH_3), 32.8 (CH_2), 32.5 (CH_2), 32.2 (CH_2), 30.5 (CH_2), 30.4 (CH_2), 29.7 (CH_2), 29.4 (CH_2), 27.4 (CH_2), 27.2 (CH_2), 27.1 (CH_2), 26.5 (CH_2).

The CH of the cyclohexyl substituents was not observed.

^{31}P{^{1}H} NMR (162 MHz, $CDCl_3$) δ (ppm) = –14.91.

^{19}F{^{1}H} NMR (282 MHz, $CDCl_3$) δ (ppm) = –73.97.

HRMS (ESI$^+$) *m/z*: calculated for $C_{56}H_{67}F_3O_{11}PS$ $[M+H]^+$: 1035.4089; found: 1035.4105.

IR (neat) ν (cm^{-1}): 2926, 2848, 1502, 1399, 1206, 1175, 1044.

Melting point: 135-137 °C.

A1-(dicyclohexylphosphine-oxide)-A2-triflate-pillar[5]arene (5d•O)

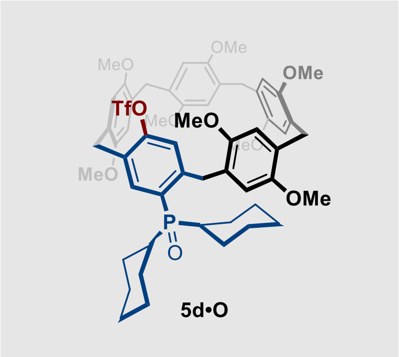

Synthesized following General Procedure III using **5d** (0.19 mmol, 1.0 equiv.). **5d•O** was obtained as a white solid. (200 mg, quantitative).

^{1}H NMR (400 MHz, $CDCl_3$) δ (ppm) = 7.35 (s, 1H), 7.32 (d, $J_{P\text{-}H}$ = 11.8 Hz, 1H), 7.11 (s, 1H), 6.85 (s, 2H), 6.84 (s, 1H), 6.82 (s, 1H), 6.79 (s, 2H), 6.71 (s, 1H), 4.50 (bs, 2H), 3.86 (s, 3H), 3.82 (s, 3H), 3.81 – 3.76 (m, 4H), 3.74 (s, 3H), 3.71 (s, 5H), 3.70 (s, 4H), 3.68 (s, 4H), 3.66 (s, 3H), 3.62 (s, 3H), 2.08 (s, 4H), 1.93 – 1.83 (m, 2H), 1.81 – 1.65 (m, 5H), 1.54 (s, 2H), 1.39 – 1.14 (m, 9H).

^{13}C{^{1}H} NMR (101 MHz, $CDCl_3$) δ (ppm) = 150.9 (C), 150.9 (C), 150.8 (C), 150.8 (C), 150.7 (C), 150.6 (C), 150.5 (C), 150.5 (C), 150.1 (C), 134.6 (CH), 134.5 (CH), 133.8 (C), 129.9 (C), 129.7 (C), 129.7 (C), 129.7 (C), 128.4 (C), 128.3 (C), 128.3 (C), 128.2 (C), 128.0 (C), 127.9 (C), 127.5 (C), 126.8 (C), 126.0 (C), 124.5 (CH), 124.4 (CH), 124.3 (C), 124.1 (C), 118.5 (q, $J_{C\text{-}F}$ = 320.7 Hz) (C), 114.7 (CH), 114.6 (CH), 114.2 (CH), 114.1 (CH), 114.0 (CH), 113.9 (CH), 113.9 (CH), 113.6 (CH), 113.5 (CH), 55.8 (CH_3), 55.7 (CH_3), 55.6 (CH_3), 55.6 (CH_3), 55.6 (CH_3), 55.5 (CH_3), 55.2 (CH_3), 36.9 (CH), 36.3 (CH), 32.3 (CH_2), 31.0 (CH_2), 30.3 (CH_2), 29.7 (CH_2), 29.5 (CH_2), 29.3 (CH_2), 29.1 (CH_2), 26.6 (CH_2), 26.5 (CH_2), 26.5 (CH_2), 26.4 (CH_2), 25.8 (CH_2), 25.6 (CH_2), 25.0 (CH_2), 25.0 (CH_2).

^{31}P{^{1}H} NMR (162 MHz, $CDCl_3$) δ (ppm) = 49.23.

^{19}F{^{1}H} NMR (282 MHz, $CDCl_3$) δ (ppm) = –73.88.

HRMS (ESI$^+$) *m/z*: calculated for $C_{56}H_{67}F_3O_{12}PS$ $[M+H]^+$: 1051.4038; found: 1051.4037.

IR (neat) ν (cm^{-1}): 2930, 2852, 1502, 1400, 1207, 1170, 1040.

Melting point: 129-131 °C.

A1-(bis((3,5-dimethoxy-4-*tert*-butyl)phenyl)phosphine-oxide)-A2-triflate-pillar[5]arene (5e•O)

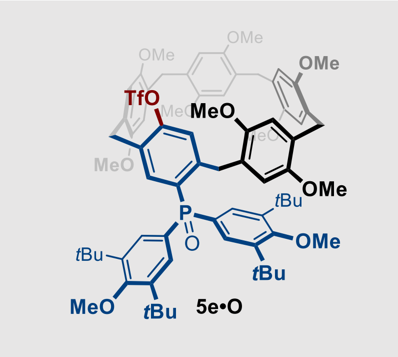

Synthesized following General Procedures II and III using **1** (1.52 mmol, 1.0 equiv.) and **2e•BH₃** (1.52 mmol, 1.00 equiv.). Purification by flash column chromatography over silica gel (eluent: *n*-pentane/CH_2Cl_2/Et_2O = 5:1:1) afforded **5e•O** as a white solid. (1.50 g, 75% yield over 2 steps).

TLC: 0.35, *n*-pentane/CH_2Cl_2/Et_2O = 5:1:1

^{1}H NMR (400 MHz, $CDCl_3$) δ (ppm) = 7.47 (d, $J_{P\text{-}H}$ = 12.5 Hz, 4H), 7.43 (d, $J_{P\text{-}H}$ = 3.2 Hz, 1H), 7.30 (s, 1H), 7.23 (s, 1H), 6.92 (s, 1H), 6.83 (s, 1H), 6.82 (s, 1H), 6.81 (s, 1H), 6.77 (s, 1H), 6.73 (s, 1H), 6.58 (s, 1H), 4.20 (bs, 2H), 3.78 (s, 2H), 3.77 (s, 2H), 3.75 (s, 5H), 3.71 (s, 12H), 3.70 (s, 5H), 3.68 (s, 3H), 3.68 (s, 3H), 3.66 (s, 3H), 3.17 (s, 3H), 1.37 (s, 36H).

^{13}C{^{1}H} NMR (101 MHz, $CDCl_3$) δ (ppm) = 162.7 (C), 162.7 (C), 151.2 (C), 151.1 (C), 151.0 (C), 150.9 (C), 150.8 (C), 150.7 (C), 150.5 (C), 149.8 (C), 148.9 (C), 148.8 (C), 144.2 (C), 144.1 (C), 137.6 (CH), 137.5 (CH), 131.0 (CH), 131.0 (C), 130.9 (CH), 130.0 (C), 129.9 (C), 129.6 (C), 129.5 (C), 128.4 (C), 128.4 (C), 128.3 (C), 128.2 (C), 127.8 (C), 127.1 (C), 124.2 (C), 123.9 (CH), 123.8 (CH), 118.7 (q, $J_{C\text{-}F}$ = 320.9 Hz) (C), 114.8 (CH), 114.5 (CH), 114.2 (CH), 114.0 (CH), 114.0 (CH), 113.7 (CH), 113.6 (CH), 64.6 (CH_3), 56.1 (CH_3), 55.9 (CH_3), 55.8 (CH_3), 55.8 (CH_3), 55.8 (CH_3), 55.8 (CH_3), 55.4 (CH_3), 55.3 (CH_3), 36.1 (C), 32.4 (CH_2), 32.0 (CH_3), 31.0 (CH_2), 29.3 (CH_2), 29.0 (CH_2).

^{31}P{^{1}H} NMR (162 MHz, $CDCl_3$) δ (ppm) = 30.70.

^{19}F{^{1}H} NMR (282 MHz, $CDCl_3$) δ (ppm) = –73.56.

HRMS (ESI$^+$) *m/z*: calculated for $C_{74}H_{91}F_3O_{14}PS$ $[M+H]^+$: 1323.5815; found: 1323.5854.

IR (neat) ν (cm^{-1}): 2952, 2829, 1503, 1400, 1208, 1141, 1044, 1000.

Melting point: 135-140 °C.

A1-(bis(3,5-bis(trifluoromethyl)phenyl)phosphine)-A2-triflate-pillar[5]arene (5f)

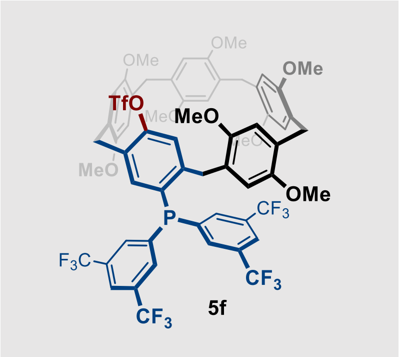

Synthesized following General Procedure II using **1** (1.52 mmol, 1.0 equiv.) and **2f** (0.75 mmol, 1.25 equiv.). Purification by flash column chromatography over silica gel (eluent: *n*-pentane/CH_2Cl_2/Et_2O = 20:1:1) afforded **5f** as a white solid. (1.22 g, 62% yield).

TLC: 0.4, *n*-pentane/CH_2Cl_2/Et_2O = 20:1:1

^{1}H NMR (400 MHz, $CDCl_3$) δ (ppm) = 7.96 (s, 2H), 7.66 (d, $J_{P\text{-}H}$ = 6.6 Hz, 4H), 7.48 (d, $J_{P\text{-}H}$ = 4.5 Hz, 1H), 6.88 (s, 1H), 6.86 (d, $J_{P\text{-}H}$ = 4.2 Hz, 1H), 6.84 (d, $J_{P\text{-}H}$ = 1.2 Hz, 2H), 6.81 (s, 1H), 6.80 (s, 1H), 6.74 (s, 1H), 6.65 (s, 1H), 6.63 (s, 1H), 4.17 – 4.12 (m, 2H), 3.83 (s, 2H), 3.79 (s, 4H), 3.75 (s, 3H), 3.74 (s, 6H), 3.73 (s, 3H), 3.69 (s, 5H), 3.59 (s, 6H), 3.19 (s, 3H).

^{13}C{^{1}H} NMR (101 MHz, $CDCl_3$) δ (ppm) = 151.1 (C), 151.0 (C), 151.0 (C), 150.9 (C), 150.8 (C), 150.7 (C), 150.7 (C), 150.2 (C), 148.5 (C), 148.2 (C), 139.4 (C), 139.2 (C), 137.5 (CH), 133.7 (CH, bs), 133.5 (CH, bs), 132.6 (q, $J_{C\text{-}F}$ = 33.4 Hz) (C), 132.6 (q, $J_{C\text{-}F}$ = 33.4 Hz) (C), 132.1 (C), 129.9 (C), 129.6 (C), 129.2 (C), 129.1 (C), 128.7 (C), 128.4 (C), 128.1 (C), 127.5 (C), 126.1 (C), 124.4 (C), 124.3 (C), 123.7 (bs, CH), 123.2 (CH), 123.2 (CH), 123.1 (q, $J_{C\text{-}F}$ = 273.5 Hz) (C), 118.7 (q, $J_{C\text{-}F}$ = 320.5 Hz) (C), 114.6 (CH), 114.3 (CH), 114.1 (CH), 114.0 (CH), 113.8 (CH), 113.6 (CH), 113.2 (CH), 113.1 (CH), 55.8 (CH_3), 55.8 (CH_3), 55.8 (CH_3), 55.7 (CH_3), 55.6 (CH_3), 55.6 (CH_3), 55.4 (CH_3), 55.3 (CH_3), 33.5 (CH_2), 33.3 (CH_2), 32.0 (CH_2), 30.3 (CH_2), 29.3 (CH_2), 29.1 (CH_2).

^{31}P{^{1}H} NMR (162 MHz, $CDCl_3$) δ (ppm) = –13.89.

^{19}F{^{1}H} NMR (282 MHz, $CDCl_3$) δ (ppm) = –62.98, –73.59.

HRMS (ESI$^+$) *m/z*: calculated for $C_{60}H_{54}F_{15}NO_{11}PS$ $[M+NH_4]^+$: 1312.2916; found: 1312.282.

IR (neat) ν (cm^{-1}): 2944, 1502, 1401, 1352, 1277, 1134, 1095, 1046.

Melting point: 112-116 °C.

A1-(bis(3,5-bis(trifluoromethyl)phenyl)phosphine-oxide)-A2-triflate-pillar[5]arene (5f•O)

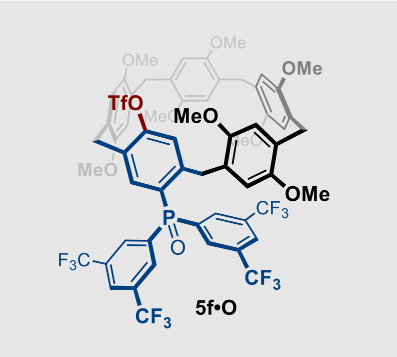

Synthesized following General Procedure III using **5f** (0.94 mmol, 1.0 equiv.). **5f•O** was obtained as a white solid. (1.31 g, quantitative).

^{1}H NMR (400 MHz, $CDCl_3$) δ (ppm) = 8.15 (d, $J_{P\text{-}H}$ = 8.5 Hz, 4H), 8.11 (s, 2H), 7.62 (d, $J_{P\text{-}H}$ = 3.5 Hz, 1H), 7.05 (s, 1H), 7.02 (d, $J_{P\text{-}H}$ = 14.8 Hz, 1H), 6.92 (s, 1H), 6.85 – 6.83 (m, 2H), 6.82 – 6.79 (m, 2H), 6.78 (s, 1H), 6.67 (s, 1H), 4.04 (bs, 2H), 3.83 (s, 2H), 3.81 (s, 2H), 3.79 (s, 3H), 3.77 (s, 3H), 3.76 (s, 2H), 3.75 (s, 3H), 3.74 (s, 3H), 3.74 (s, 3H), 3.71 (s, 2H), 3.65 (s, 3H), 3.58 (s, 3H), 3.30 (s, 3H).

^{13}C{^{1}H} NMR (101 MHz, $CDCl_3$) δ (ppm) = 152.44 (C), 152.40 (C), 151.2 (C), 151.0 (C), 150.7 (C), 150.6 (C), 150.5 (C), 150.3 (C), 149.5 (C), 149.5 (C), 149.4 (C), 137.1 (CH), 136.9 (CH), 136.1 (C), 135.1 (C), 132.8 (q, $J_{C\text{-}F}$ = 34.2 Hz) (C), 132.8 (q, $J_{C\text{-}F}$ = 34.2 Hz) (C), 131.7 (CH), 131.7 (CH), 130.8 (C), 130.7 (C), 130.2 (C), 129.0 (C), 128.6 (C), 128.5 (C), 127.9 (C), 127.1 (C), 126.6 (CH), 126.5 (CH), 126.5 (CH), 126.5 (CH), 126.4 (CH), 125.9 (C), 125.1 (C), 124.8 (C), 124.8 (CH), 124.6 (CH), 123.2 (C), 122.6 (q, $J_{C\text{-}F}$ = 272.5 Hz) (C), 118.5 (C), 118.5 (q, $J_{C\text{-}F}$ = 320.5 Hz) (C), 114.2 (CH), 114.1 (CH), 114.0 (CH), 113.9 (CH), 113.7 (CH), 113.6 (CH_3), 55.8 (CH_3), 55.7 (CH_3), 55.6 (CH_3), 55.6 (CH_3), 55.5 (CH_3), 55.5 (CH_3), 55.1 (CH_3), 55.1 (CH_3), 33.1 (CH_2), 33.0 (CH_2), 32.6 (CH_2), 30.9 (CH_2), 28.9 (CH_2), 28.7 (CH_2).

^{31}P{^{1}H} NMR (162 MHz, $CDCl_3$) δ (ppm) = 25.91.

^{19}F{^{1}H} NMR (282 MHz, $CDCl_3$) δ (ppm) = –62.89, –73.54.

HRMS (ESI$^+$) *m/z*: calculated for $C_{60}H_{54}F_{15}NO_{12}PS$ $[M+NH_4]^+$: 1328.2860; found: 1328.2899.

IR (neat) ν (cm^{-1}): 2933, 2831, 1595, 1500, 1210, 1175, 1115, 1043.

Melting point: 181-183 °C.

A1-(di([1,1'-biphenyl]-4-yl)phosphine-oxide)-A2-triflate-pillar[5]arene (5g•O)

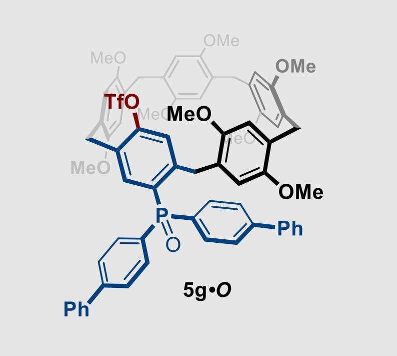

Synthesized following General Procedures II and III using **1** (0.25 mmol, 1.0 equiv.) and **2g** (0.25 mmol, 1.00 equiv.). Purification by flash column chromatography over silica gel (eluent: *n*-pentane/CH_2Cl_2/Et_2O = 10:1:1) afforded **5g•O** as a white solid. (211 mg, 70% yield over 2 steps).

TLC: 0.4, *n*-pentane/CH_2Cl_2/Et_2O = 10:1:1

^{1}H NMR (400 MHz, $CDCl_3$) δ (ppm) = 7.81 – 7.69 (m, 8H), 7.69 – 7.60 (m, 4H), 7.54 – 7.46 (m, 5H), 7.45 – 7.39 (m, 2H), 7.23 (d, $J_{P\text{-}H}$ = 13.9 Hz, 1H), 7.11 (s, 1H), 6.93 (s, 1H), 6.90 (s, 1H), 6.82 (s, 2H), 6.81 (s, 1H), 6.77 (s, 1H), 6.62 (s, 1H), 4.24 (s, 2H), 3.80 (d, $J_{H\text{-}H}$ = 3.4 Hz, 4H), 3.75 (s, 8H), 3.72 – 3.67 (m, 11H), 3.64 (s, 6H), 3.37 (s, 3H).

^{13}C{^{1}H} NMR (101 MHz, $CDCl_3$) δ (ppm) = 151.7 (C), 151.2 (C), 150.9 (C), 150.9 (C), 150.9 (C), 150.6 (C), 150.4 (C), 150.3 (C), 149.6 (C), 149.1 (C), 149.0 (C), 144.7 (C), 139.8 (C), 137.7 (CH), 132.6 (CH), 129.8 (C), 129.8 (C), 129.6 (C), 129.5 (C), 129.1 (CH), 128.7 (C), 128.3 (CH), 128.2 (C), 128.2 (C), 128.2 (C), 127.6 (C), 127.4 (CH), 127.3 (CH), 126.7 (C), 123.9 (CH), 123.7 (C), 118.5 (q, $J_{C\text{-}F}$ = 320.6 Hz) (C), 114.5 (CH), 114.4 (CH), 114.2 (CH), 114.1 (CH), 114.0 (CH), 114.0 (CH), 113.4 (CH), 112.9 (CH), 56.1 (CH_3), 55.6 (CH_3), 55.6 (CH_3), 55.5 (CH_3), 55.3 (CH_3), 55.0 (CH_3), 32.7 (CH_2), 32.2 (CH_2), 31.1 (CH_2), 29.0 (CH_2), 28.9 (CH_2).

^{31}P{^{1}H} NMR (162 MHz, $CDCl_3$) δ (ppm) = 30.4.

^{19}F{^{1}H} NMR (282 MHz, $CDCl_3$) δ (ppm) = –73.79.

HRMS (ESI$^+$) *m/z*: calculated for $C_{68}H_{63}F_3O_{12}PS$ $[M+H]^+$: 1191.3730; found: 1191.3701.

IR (neat) ν (cm^{-1}): 2935, 2829, 1502, 1400, 1208, 1176, 1043.

Melting point: 146-150 °C.

A1-(bis(3,5-bis(trifluoromethyl)phenyl)phosphine-oxide)-A2-triflate-ethoxy-pillar[5]arene (7f•O)

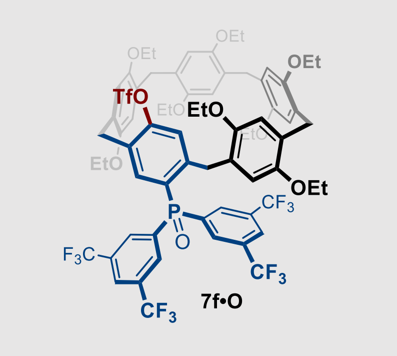

Synthesized following General Procedures II and III using **6** (0.51 mmol, 1.0 equiv.) and **2f** (0.25 mmol, 1.00 equiv.). Purification by flash column chromatography over silica gel (eluent: *n*-pentane/CH_2Cl_2/Et_2O = 20:1:1) afforded **7f•O** as a white solid. (361 mg, 50% yield over two steps).

TLC: 0.4, *n*-pentane/CH_2Cl_2/Et_2O = 20:1:1

^{1}H NMR (400 MHz, $CDCl_3$) δ (ppm) = 8.29 – 8.02 (m, 6H), 7.60 (d, $J_{P\text{-}H}$ = 3.7 Hz, 1H), 7.17 (d, $J_{P\text{-}H}$ = 14.6 Hz, 1H), 6.99 (s, 1H), 6.87 (s, 1H), 6.83 (s, 1H), 6.83 (s, 1H), 6.79 (s, 1H), 6.78 (s, 1H), 6.77 (s, 1H), 6.69 (s, 1H), 4.04 – 3.83 (m, 15H), 3.81 (d, $J_{H\text{-}H}$ = 7.5 Hz, 4H), 3.75 (d, $J_{H\text{-}H}$ = 8.9 Hz, 4H), 3.60 (q, $J_{H\text{-}H}$ = 6.9 Hz, 2H), 1.47 – 1.29 (m, 18H), 1.21 (t, $J_{H\text{-}H}$ = 7.0 Hz, 3H), 0.77 (t, $J_{H\text{-}H}$ = 6.9 Hz, 3H).

^{13}C{^{1}H} NMR (101 MHz, $CDCl_3$) δ (ppm) = 152.6 (C), 152.5 (C), 150.5 (C), 150.3 (C), 150.0 (C), 149.9 (C), 149.8 (C), 149.8 (C), 149.8 (C), 149.7 (C), 149.6 (C), 149.5 (C), 148.8 (C), 137.0 (CH), 136.9 (CH), 133.2 (C), 133.0 (C), 132.9 (q, $J_{C\text{-}F}$ = 34.2 Hz) (C), 132.9 (q, $J_{C\text{-}F}$ = 34.2 Hz) (C), 132.2–131.5 (m) (CH), 131.4 (C), 131.3 (C), 130.5 (C), 129.6 (C), 129.0 (C), 128.9 (C), 128.2 (C), 127.5 (C), 126.6 (CH), 126.0 (C), 125.4 (C), 125.4 (CH), 125.2 (CH), 122.7 (q, $J_{C\text{-}F}$ = 273.5 Hz) (C), 122.7 (q, $J_{C\text{-}F}$ = 273.5 Hz) (C), 118.6 (q, $J_{C\text{-}F}$ = 321.0 Hz) (C), 115.8 (CH), 115.3 (CH), 115.2 (CH), 114.9 (CH), 114.8 (CH), 114.8 (CH), 114.6 (CH), 114.6 (CH), 63.9 (CH_2), 63.9 (CH_2), 63.9 (CH_2), 63.8 (CH_2), 63.8 (CH_2), 63.7 (CH_2), 63.6 (CH_2), 63.4 (CH_2), 33.3 (CH_2), 33.2 (CH_2), 33.0 (CH_2), 31.0 (CH_2), 29.1, 29.0 (CH_2), 15.4 (CH_3), 15.4 (CH_3), 15.3 (CH_3), 15.2 (CH_3), 15.2 (CH_3), 14.7 (CH_3).

^{31}P{^{1}H} NMR (162 MHz, $CDCl_3$) δ (ppm) = –24.70.

^{19}F{^{1}H} NMR (282 MHz, $CDCl_3$) δ (ppm) = –62.96, –73.48.

HRMS (ESI$^+$) *m/z*: calculated for $C_{68}H_{70}F_{15}NO_{12}PS$ $[M+NH_4]^+$: 1440.4111; found: 1440.4087.

IR (neat) ν (cm^{-1}): 2979, 2900, 1503, 1360, 1278, 1204, 1133, 1048.

Melting point: 177-178 °C.

6. Syntheses of A1-phosphine-A2-aryl-pillar[5]arenes

6.1 General Procedure IV (GP-IV) for Suzuki-Miyaura cross-coupling

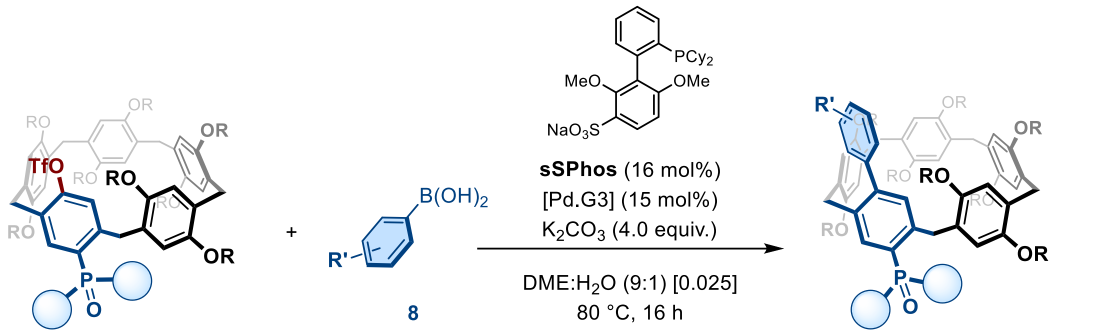

8
(4.0 equiv.)

5•O (R = OMe)
7•O (R = OEt)

9•O (R = OMe)
10•O (R = OEt)

In a glovebox, in an oven dried J-Young flask [Pd.G$_3$] (5.3 mg, 15 µmol, 15.0 mol%), sSPhos (8.2 mg, 16 µmol, 16.0 mol%) were dissolved in 1,2-dimethoxyethane (3.5 mL). The mixture was stirred at 23 °C. After 10 min., the desired A1-phosphine-oxide-A2-triflate-pillar[5]arene **5•O or 7•O** (0.1 mmol, 1.0 equiv.), the appropriate boronic acid **8** (0.4 mmol, 4.0 equiv.) and K_2CO_3 (55.3 mg, 0.4 mmol, 4.0 equiv.) were added in sequence. The J-Young flask was sealed, taken out of the glove box and attached to a two-manifold Schlenk line. Degassed H_2O was added (0.4 mL). Next, the J-Young flask was placed in a preheated oil bath and stirred at 80 °C. After 16 h, the reaction mixture was cooled to 23 °C, diluted with H_2O (20 mL) and extracted with CH_2Cl_2 (3 $\times$ 15 mL). The combined organic phases were washed with a saturated aqueous NaCl solution (50 mL), dried over Na_2SO_4, filtered and concentrated under reduced pressure. The phosphine oxide obtained was pure enough to be engaged in the next step without further purification.

6.2 General Procedure V (GP-V) for phosphine oxide reduction

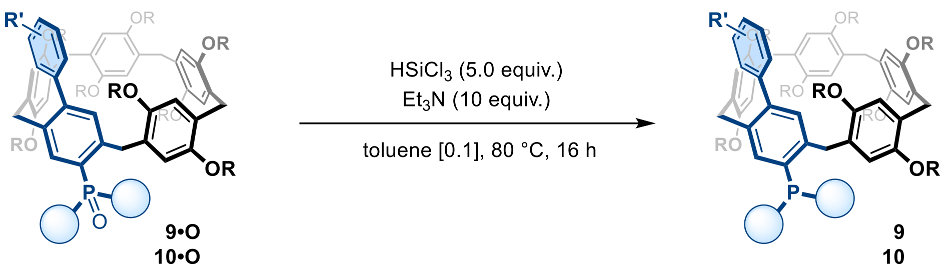

In an oven dried J-Young flask, the desired A1-phosphine-oxide-A2-aryl-pillar[5]arene **9•O** or **10•O** (0.1 mmol, 1.0 equiv.) was dissolved in toluene (10 mL), and $HSiCl_3$ (50 µL, 0.5 mmol, 5.0 equiv.) and Et_3N (139 µL, mmol, 10 equiv.) were added in sequence. The J-Young flask was placed in a preheated oil bath and stirred at 80 °C. After 16 h, ^{31}P NMR analysis indicated full consumption of the starting material. The J-Young flask was cooled to 0 °C with an ice/water bath. After 10 min., an aqueous NaOH solution (4.0 M) was added dropwise (*ca.* 10 mL). The mixture was diluted with H_2O (20 mL) and extracted with CH_2Cl_2 (3 × 20 mL). The combined organic phases were dried over Na_2SO_4, filtered and concentrated under reduced pressure. The residue was purified by flash column chromatography over silica gel using *n*-pentane/CH_2Cl_2/Et_2O as eluent to afford A1-phosphine-A2-aryl-pillar[5]arene **9** or **10**.

A1-(bis(4-methoxyphenyl)phosphine)-A2-(4-(methoxy)phenyl)-pillar[5]arene (9aa)

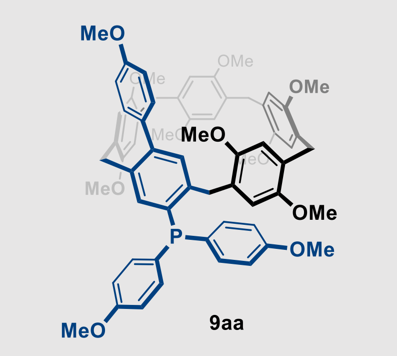

Synthesized following General Procedures IV and V, using **5a•O** (0.5 mmol, 1.0 equiv.) and **8a** (2.0 mmol, 4.0 equiv.). Purification by flash column chromatography over silica gel (eluent: *n*-pentane/CH_2Cl_2/Et_2O = 8:1:1) afforded **9aa** as a white solid. (458 mg, 86% yield over two steps).

TLC: 0.3, *n*-pentane/CH_2Cl_2/Et_2O = 8:1:1

^{1}H NMR (400 MHz, $CDCl_3$) δ (ppm) = 7.30 (dd, $J_{H\text{-}H}$ = 8.6 Hz, $J_{P\text{-}H}$ = 7.2 Hz, 2H), 7.26 – 7.22 (dd, $J_{H\text{-}H}$ = 8.6 Hz, $J_{P\text{-}H}$ = 7.5 Hz, 2H, 2H), 6.94 – 6.90 (m, 3H), 6.87 (d, $J_{H\text{-}H}$ = 8.0 Hz, 2H), 6.83 (s, 1H), 6.80 (s, 1H), 6.73 (d, $J_{H\text{-}H}$ = 3.9 Hz, 2H), 6.68 (s, 1H), 6.67 (s, 1H), 6.66 (s, 1H), 6.64 – 6.56 (m, 4H), 6.52 (s, 1H), 5.53 (s, 1H), 4.30 (dd, $J_{H\text{-}H}$ = 14.5 Hz, $J_{P\text{-}H}$ = 4.8 Hz, 1H), 3.97 – 3.84 (m, 4H), 3.84 (s, 3H), 3.82 (s, 3H), 3.82 (s, 3H), 3.81 – 3.75 (m, 3H), 3.73 (s, 3H), 3.72 – 3.66 (m, 2H), 3.65 (s, 3H), 3.61 (s, 3H), 3.56 (s, 3H), 3.50 (s, 3H), 3.47 (s, 3H), 3.27 (s, 3H), 3.23 (s, 3H).

^{13}C{^{1}H} NMR (101 MHz, $CDCl_3$) δ (ppm) = 160.2 (C), 160.1 (C), 158.2 (C), 151.3 (C), 151.2 (C), 151.1 (C), 151.1 (C), 151.0 (C), 150.9 (C), 150.6 (C), 150.3 (C), 144.0 (C), 143.8 (C), 142.1 (C), 136.6 (C), 136.1 (CH), 135.9 (CH), 135.7 (CH), 135.2 (CH), 135.0 (CH), 134.9 (C), 133.6 (C), 133.4 (C), 131.7 (CH), 131.6 (CH), 130.1 (CH), 129.1 (C), 129.1 (C), 129.0 (C), 128.7 (C), 128.7 (C), 128.6 (C), 128.5 (C), 128.5, (C) 128.4 (C), 128.1 (C), 127.8 (C), 115.0 (CH), 114.5 (CH), 114.5 (CH), 114.4 (CH), 114.4 (CH), 114.3 (CH), 114.2, (CH) 114.1 (CH), 114.0 (CH), 113.9 (CH), 113.8 (CH), 113.4 (CH), 113.3 (CH), 56.3 (CH_3), 56.2 (CH_3), 56.0 (CH_3), 56.0 (CH_3), 55.9 (CH_3), 55.5 (CH_3), 55.3 (CH_3), 55.2 (CH_3), 34.2 (CH_2), 33.3 (CH_2), 33.1 (CH_2), 30.6 (CH_2), 30.3 (CH_2), 29.6 (CH_2).

^{31}P{^{1}H} NMR (162 MHz, $CDCl_3$) δ (ppm) = –17.12.

HRMS (ESI$^+$) *m/z*: calculated for $C_{64}H_{66}O_{11}P$ $[M+H]^+$: 1041.4338; found: 1041.4322.

IR (neat) ν (cm^{-1}): 2931, 2830, 1496, 1462, 1398, 1209, 1175, 1044.

Melting point: 132-135 °C.

A1-(dicyclohexylphosphine)-A2-(4-(methoxy)phenyl)-pillar[5]arene (9da)

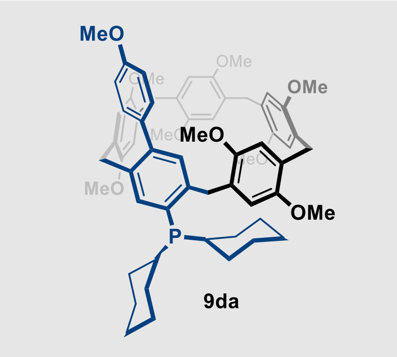

9da

Synthesized following General Procedures IV and V, using **5d•O** (0.1 mmol, 1.0 equiv.) and **8a** (0.4 mmol, 4.0 equiv.). Purification by flash column chromatography over silica gel (eluent: *n*-pentane/CH_2Cl_2/Et_2O = 10:1:1) afforded **9da** as a white solid. (123 mg, 66% yield over two steps).

TLC: 0.4, *n*-pentane/CH_2Cl_2/Et_2O = 10:1:1

^{1}H NMR (400 MHz, $CDCl_3$) δ (ppm) = 7.34 (d, $J_{P\text{-}H}$ = 2.8 Hz, 1H), 6.86 (s, 1H), 6.75 (s, 1H), 6.74 (s, 1H), 6.66 – 6.62 (m, 3H), 6.59 (s, 1H), 6.57 (s, 4H), 6.53 (s, 1H), 5.68 (s, 1H), 4.70 (dd, $J_{H\text{-}H}$ = 14.5, $J_{P\text{-}H}$ = 6.2 Hz, 1H), 4.01 (d, $J_{H\text{-}H}$ = 14.1 Hz, 1H), 3.89 – 3.72 (m, 10H), 3.69 (s, 3H), 3.67 – 3.65 (m, 1H), 3.64 (s, 3H), 3.59 (s, 3H), 3.57 (s, 3H), 3.46 (s, 3H), 3.44 (s, 3H), 3.39 (s, 3H), 3.22 (s, 3H), 2.11 – 1.91 (m, 3H), 1.87 – 1.52i (m, 9H), 1.39 – 1.33 (m, 2H), 1.30 – 0.93 (m, 8H).

^{13}C{^{1}H} NMR (101 MHz, $CDCl_3$) δ (ppm) = 158.2 (C), 151.3 (C), 151.2 (C), 151.1 (C), 151.1 (C), 151.0 (C), 150.9 (C), 150.6 (C), 150.6 (C), 146.7 (C), 146.5 (C), 141.8 (C), 136.0 (C), 135.0 (C), 134.7 (CH), 134.6 (CH), 131.9 (CH), 131.9 (CH), 131.0 (CH), 130.9 (C), 130.0 (C), 129.5 (C), 129.2 (CH), 128.9 (C), 128.8 (C), 128.7 (C), 128.5 (C), 128.4 (C), 128.4 (C), 128.3 (CH), 128.1 (C), 115.2 (CH), 115.2 (CH), 115.0 (CH), 114.4 (CH), 114.3 (CH), 113.9 (CH), 113.8 (CH), 113.7 (CH), 113.6 (CH), 113.2 (CH), 56.4 (CH_3), 56.2 (CH_3), 56.1 (CH_3), 56.0 (CH_3), 55.9 (CH_3), 55.9 (CH_3), 55.7 (CH_3), 55.4 (CH_3), 55.3 (CH_3), 35.2 (CH_3), 35.1 (CH_3), 33.8 (CH_2), 33.7 (CH_2), 33.5 (CH_2), 32.8 (CH_3), 32.7 (CH_3), 31.0 (CH_2), 30.8 (CH_2), 30.4 (CH_2), 30.3 (CH_2), 30.2 (CH_2), 30.1 (CH_2), 29.9 (CH_2), 28.5 (CH_2), 28.5 (CH_2), 27.7 (CH_2), 27.6 (CH_2), 27.4 (CH_2), 27.4 (CH_2), 27.2 (CH_2), 27.1 (CH_2), 27.0 (CH_2), 26.8 (CH_2), 26.6 (CH_2).

^{31}P{^{1}H} NMR (162 MHz, $CDCl_3$) δ (ppm) = –15.33.

HRMS (ESI$^+$) *m/z*: calculated for $C_{62}H_{73}O_9P$ $[M+H]^+$: 993.5066; found: 993.5116.

IR (neat) ν (cm^{-1}): 2932, 2847, 1501, 1463, 1398, 1208, 1173, 1044.

Melting point: 172-173 °C.

A1-(bis((3,5-dimethoxy-4-*tert*-butyl)phenyl)phosphine)-A2-(4-(methoxy)phenyl)-pillar[5]arene (9ea)

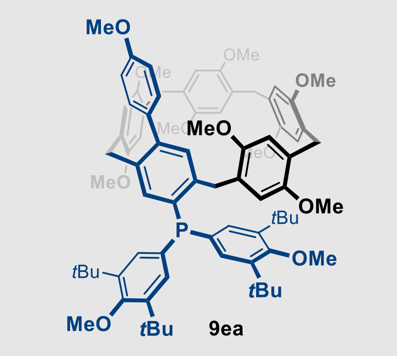

Synthesized following General Procedures IV and V, using **5e•O** (0.23 mmol, 1.0 equiv.) and **8a** (0.91 mmol, 4.0 equiv.). Purification by flash column chromatography over silica gel (eluent: *n*-pentane/CH_2Cl_2/Et_2O = 9:1:1) afforded **9ea** as a white solid. (237 mg, 83% yield over two steps).

TLC: 0.5, *n*-pentane/CH_2Cl_2/Et_2O = 9:1:1

^{1}H NMR (400 MHz, $CDCl_3$) δ (ppm) = 7.24 (d, $J_{P\text{-}H}$ = 8.1 Hz, 2H), 7.08 (d, $J_{P\text{-}H}$ = 7.6 Hz, 2H), 6.90 – 6.86 (m, 2H), 6.82 (s, 1H), 6.79 (s, 1H), 6.73 (s, 2H), 6.62 (s, 1H), 6.61 (d, $J_{P\text{-}H}$ = 4.8 Hz, 1H), 6.55 – 6.37 (m, 5H), 5.46 (s, 1H), 4.37 (dd, $J_{H\text{-}H}$ = 14.5, $J_{P\text{-}H}$ = 5.2 Hz, 1H), 3.91 (d, J = 13.5 Hz, 1H), 3.87 – 3.81 (m, 4 H), 3.80 (s, 3H), 3.76 – 3.75 (m, 2H), 3.73 (s, 3H), 3.71 (s, 3H), 3.68 (s, 3H), 3.65 (s, 4H), 3.65 (s, 3H), 3.59 (s, 3H), 3.54 (s, 3H), 3.53 (s, 3H), 3.46 (d, $J_{\text{H-H}}$ = 13.7 Hz, 1H), 3.23 (s, 3H), 2.99 (s, 3H), 1.37 (s, 18H), 1.35 (s, 18H).

^{13}C{^{1}H} NMR (101 MHz, $CDCl_3$) δ (ppm) = 160.2 (C), 160.0 (C), 158.1 (C), 151.3 (C), 151.2 (C), 151.1 (C), 151.0 (C), 150.99 (C), 150.8 (C), 150.7 (C), 150.5 (C), 143.9 (C), 143.6 (C), 143.4 (C), 143.4 (C), 143.3 (C), 143.2 (C), 142.0 (C), 136.3 (C), 135.4 (CH), 135.2 (CH), 134.0 (CH), 133.7 (CH), 133.6 (C), 133.5 (C), 131.9 (CH), 131.8 (CH), 131.5 (CH), 131.3 (CH), 131.0 (C), 130.9 (C), 129.9 (CH), 128.8 (C), 128.8 (C), 128.6 (C), 128.4 (C), 128.1 (C), 114.9 (CH), 114.6 (CH), 114.5 (CH), 114.5 (CH), 114.0 (CH), 113.5 (CH), 113.3 (CH), 113.2 (CH), 64.4 (CH_3), 64.3 (CH_3), 56.3 (CH_3), 56.2 (CH_3), 56.1 (CH_3), 56.0 (CH_3), 55.9 (CH_3), 55.7 (CH_3), 55.6 (CH_3), 55.3 (CH_3), 55.2 (CH_3), 35.9 (C), 35.9 (C), 34.5 (CH_2), 33.1 (CH_2), 32.9 (CH_2), 32.1 (CH_3), 30.5 (CH_2), 30.2 (CH_2), 29.6 (CH_2).

^{31}P{^{1}H} NMR (162 MHz, $CDCl_3$) δ (ppm) = 14.68.

HRMS (ESI$^+$) *m/z*: calculated for $C_{80}H_{98}O_{11}P$ $[M+H]^+$: 1265.6842; found: 1265.6830.

IR (neat) ν (cm^{-1}): 2948, 2827, 1502, 1395, 1464, 1208, 1176, 1049.

Melting point: 186-188 °C.

A1-(di([1,1'-biphenyl]-4-yl)phosphine)-A2-(4-(methoxy)phenyl)-pillar[5]arene (9ga)

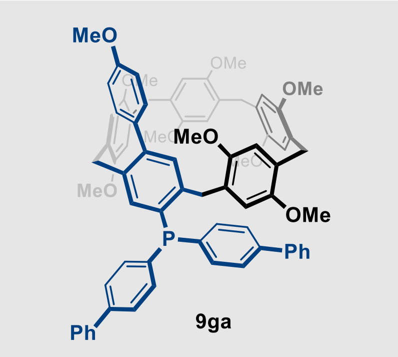

Synthesized following General Procedures IV and V, using **5g•O** (0.34 mmol, 1.0 equiv.) and **8a** (1.34 mmol, 4.0 equiv.). Purification by flash column chromatography over silica gel (eluent: *n*-pentane/CH_2Cl_2/Et_2O = 8:1:1) afforded **9ga** as a white solid. (308 mg, 81% yield over two steps).

TLC: 0.5, *n*-pentane/CH_2Cl_2/Et_2O = 8:1:1

^{1}H NMR (400 MHz, $CDCl_3$) δ (ppm) = 7.71 – 7.57 (m, 6H), 7.57 – 7.32 (m, 12H), 7.10 (d, $J_{P\text{-}H}$ = 4.6 Hz, 1H), 6.90 (d, $J_{P\text{-}H}$ = 4.9 Hz, 1H), 6.81 (s, 1H), 6.78 (s, 1H), 6.73 (d, $J_{H\text{-}H}$ = 9.3 Hz, 5H), 6.65 (d, $J_{H\text{-}H}$ = 9.0 Hz, 3H), 6.50 (s, 1H), 5.53 (s, 1H), 4.44 (dd, $J_{H\text{-}H}$ = 14.2 Hz, $J_{P\text{-}H}$ = 5.3 Hz, 1H), 4.01 – 3.84 (m, 4H), 3.83 (s, 3H), 3.81 – 3.74 (m, 3H), 3.71 (s, 3H), 3.70 – 3.62 (m, 3H), 3.58 (s, 3H), 3.58 (s, 3H), 3.56 (s, 3H), 3.54 (s, 3H), 3.42 (s, 3H), 3.26 (s, 3H), 3.20 (s, 3H).

^{13}C{^{1}H} NMR (101 MHz, $CDCl_3$) δ (ppm) = 158.3 (C), 151.3 (C), 151.3 (C), 151.2 (C), 151.1 (C), 151.0 (C), 150.9 (C), 150.6 (C), 150.3 (C), 142.5 (C), 141.4 (C), 141.3 (C), 140.8 (C), 140.8 (C), 136.9 (C), 136.5 (CH), 136.4 (CH), 135.1 (CH), 134.9 (CH), 134.2 (CH), 134.0 (CH), 132.1 (CH), 132.0 (CH), 130.3 (CH), 129.0 (C), 129.0 (CH), 128.8 (CH), 128.6 (C), 128.55 (C), 128.4 (C), 128.2 (C), 127.7 (CH), 127.6 (C), 127.62 (CH), 127.3 (CH), 127.2 (CH), 127.1 (CH), 114.9 (CH), 114.9 (CH), 114.5 (CH), 114.3 (CH), 114.28 (CH), 114.2 (CH), 114.0 (CH), 113.6 (CH), 113.6 (CH), 113.4 (CH), 113.4 (CH), 56.3 (CH_3), 56.1 (CH_3), 56.1 (CH_3), 55.9 (CH_3), 55.7 (CH_3), 55.7 (CH_3), 55.4 (CH_3), 55.3 (CH_3), 55.2 (CH_3), 33.9 (CH_2), 33.9 (CH_2), 33.5 (CH_2), 33.3 (CH_2), 30.1 (CH_2), 29.9 (CH_2), 29.7 (CH_2).

^{31}P{^{1}H} NMR (162 MHz, $CDCl_3$) δ (ppm) = 16.15.

HRMS (ESI$^+$) *m/z*: calculated for $C_{74}H_{70}O_9P$ [M+H]$^+$: 1133.4753; found: 1133.4701.

IR (neat) ν (cm^{-1}): 2930, 2826, 1501, 1398, 1208, 1173, 1042.

Melting point: 182-183 °C.

A1-(bis(3,5-bis(trifluoromethyl)phenyl)phosphine)-A2-(4-(methoxy)phenyl)-pillar[5]arene (9fa)

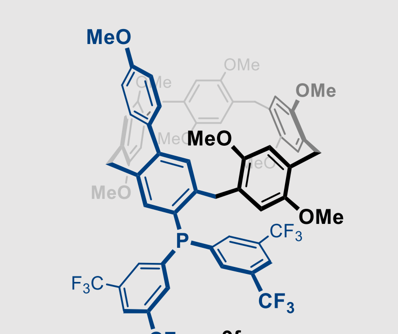

9fa

Synthesized following General Procedures IV and V, using **5f•O** (0.38 mmol, 1.0 equiv.) and **8a** (3.1 mmol, 4.0 equiv.). Purification by flash column chromatography over silica gel (eluent: *n*-pentane/CH_2Cl_2/Et_2O = 20:1:1) afforded **9fa** as a white solid. (445 mg, 88% yield over two steps).

TLC: 0.5, *n*-pentane/CH_2Cl_2/Et_2O = 20:1:1

^{1}H NMR (400 MHz, $CDCl_3$) δ (ppm) = 7.97 (s, 1H), 7.89 (s, 1H), 7.78 (d, J = 6.8 Hz, 2H), 7.59 (d, J = 5.9 Hz, 2H), 7.33 – 7.27 (m, 2H), 7.05 (d, J = 8.1 Hz, 2H), 6.92 (s, 1H), 6.91 (s, 1H), 6.84 (m, 3H), 6.82 (s, 2H), 6.80 (s, 1H), 6.60 (s, 1H), 5.51 (s, 1H), 4.04 – 3.93 (m, 2H), 3.86 (s, 4H), 3.86 (s, 3H), 3.84 – 3.80 (m, 4H), 3.78 (s, 6H), 3.72 (s, 3H), 3.69 (s, 1H), 3.65 (s, 3H), 3.62 (s, 5H), 3.35 (s, 3H), 3.01 (s, 3H).

^{13}C{^{1}H} NMR (101 MHz, $CDCl_3$) δ (ppm) = 158.8 (C), 150.7 (C), 150.7 (C), 150.7 (C), 150.6 (C), 150.6 (C), 150.4 (C), 150.3 (C), 149.9 (C), 144.3 (C), 144.1 (C), 143.6 (C), 140.4 (C), 140.2 (C), 139.7 (C), 139.5 (C), 138.7 (C), 135.3 (CH), 135.2 (CH), 135.1 (CH), 134.3 (C), 133.3 (CH), 133.3 (CH), 132.8 (C), 132.6 (C), 132.4 (C), 132.4 (C), 132.4 (C), 132.3 (C), 132.1 (C), 132.0 (C), 132.0 (C), 132.0 (C), 131.8 (C), 131.7 (CH), 131.6 (CH), 130.8 (CH), 129.6 (C), 128.6 (C), 128.5 (C), 128.3 (C), 128.3 (C), 127.8 (C), 127.4 (C), 127.3 (C), 127.2 (C), 127.3, 123.5 (CH), 123.2 (CH), 123.2 (q, $J_{C\text{-}F}$ = 273.4 Hz) (C), 123.2 (q, $J_{C\text{-}F}$ = 273.4 Hz) (C), 114.1 (CH), 113.8 (CH), 113.7 (CH), 113.6 (CH), 113.3 (CH), 112.9 (CH), 112.0 (CH), 55.8 (CH_3), 55.7 (CH_3), 55.7 (CH_3), 55.5 (CH_3), 55.5 (CH_3), 55.3 (CH_3), 55.2 (CH_3), 55.1 (CH_3), 55.1 (CH_3), 55.1 (CH_3), 33.8 (CH_2), 33.6 (CH_2), 32.2 (CH_2), 29.8 (CH_2), 29.4 (CH_2), 29.2 (CH_2).

^{31}P{^{1}H} NMR (162 MHz, $CDCl_3$) δ (ppm) = –9.71.

^{19}F{^{1}H} NMR (282 MHz, $CDCl_3$) δ (ppm) = –62.84, –62.95.

HRMS (ESI$^+$) *m/z*: calculated for $C_{66}H_{61}F_{12}NO_9P$ [M+NH$_4$]$^+$: 1270.3888; found: 1270.3894.

IR (neat) ν (cm^{-1}): 2936, 2829, 1502, 1352, 1276, 1211, 1094, 1044.

Melting point: 234-236 °C.

A1-(bis(3,5-bis(trifluoromethyl)phenyl)phosphine)-A2-(4-(trifluoromethyl)phenyl)-pillar[5]arene (9fb)

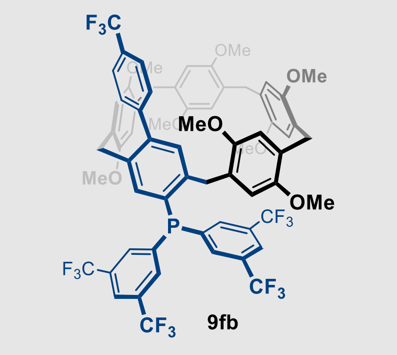

Synthesized following General Procedures IV and V, using **5f•O** (0.23 mmol, 1.0 equiv.) and **8b** (0.92 mmol, 4.0 equiv.). Purification by flash column chromatography over silica gel (eluent: *n*-pentane/CH_2Cl_2/Et_2O = 20:1:1) afforded **9fb** as a white solid. (179 mg, 61% yield over two steps).

TLC: 0.5, *n*-pentane/CH_2Cl_2/Et_2O = 20:1:1

^{1}H NMR (400 MHz, $CDCl_3$) δ (ppm) = 7.97 (s, 1H), 7.94 (s, 1H), 7.83 (d, $J_{P\text{-}H}$ = 6.6 Hz, 2H), 7.74 (d, $J_{P\text{-}H}$ = 6.2 Hz, 2H), 7.09 (d, $J_{H\text{-}H}$ = 7.9 Hz, 2H), 6.85 (s, 1H), 6.78 (d, $J_{P\text{-}H}$ = 5.1 Hz, 1H), 6.76 (s, 1H), 6.74 (s, 1H), 6.62 (s, 1H), 6.60 (s, 1H), 6.59 (s, 1H), 6.53 (s, 1H), 6.50 (d, J = 7.9 Hz, 2H), 6.46 (d, $J_{P\text{-}H}$ = 5.1 Hz, 1H), 5.35 (s, 1H), 4.19 (dd, $J_{H\text{-}H}$ = 15.0, $J_{P\text{-}H}$ = 4.2 Hz, 1H), 3.98 – 3.82 (m, 4H), 3.79 (s, 3H), 3.78 – 3.75 (m, 2H), 3.72 (s, 3H), 3.66 (s, 2H), 3.63 (s, 3H), 3.60 (s, 3H), 3.46 (d, $J_{H\text{-}H}$ = 14.1 Hz, 1H), 3.39 (s, 3H), 3.35 (s, 3H), 3.23 (s, 3H), 3.11 (s, 3H).

^{13}C{^{1}H} NMR (101 MHz, $CDCl_3$) δ (ppm) = 151.4 (C), 151.3 (C), 151.3 (C), 151.1 (C), 151.0 (C), 150.9 (C), 150.6 (C), 150.5 (C), 145.5 (C), 145.1 (C), 144.8 (C), 143.3 (C), 140.0 (C), 139.9 (C), 139.8 (C), 139.7 (C), 138.2 (C), 135.6 (CH), 134.1 (CH), 133.9 (CH), 133.4 (CH), 133.2 (C), 132.4 (q, $J_{C\text{-}F}$ = 33.2 Hz) (C), 132.4 (q, $J_{C\text{-}F}$ = 33.2 Hz) (C), 131.6 (CH), 131.5 (CH), 129.9 (C), 128.9 (C), 128.9 (CH), 128.8 (CH), 128.6 (C), 128.6 (C), 128.5 (C), 128.4 (C), 128.3 (C), 126.5 (C), 126.4 (C), 126.4 (C), 124.9 (CH), 123.6 (CH), 124.4 (q, $J_{C\text{-}F}$ = 272.0 Hz) (C), 123.2 (q, $J_{C\text{-}F}$ = 273.0 Hz) (C), 114.6 (CH), 114.6 (CH), 114.3 (CH), 114.2 (CH), 113.9 (CH), 113.9 (CH), 113.4 (CH), 113.2 (CH), 56.2 (CH_3), 56.1 (CH_3), 56.04 (CH_3), 55.8 (CH_3), 55.7 (CH_3), 55.5 (CH_3), 55.4 (CH_3), 55.2 (CH_3), 34.8 (CH_2), 34.2 (CH_2), 34.0 (CH_2), 30.5 (CH_2), 29.3 (CH_2).

^{31}P{^{1}H} NMR (162 MHz, $CDCl_3$) δ (ppm) = –11.90.

^{19}F{^{1}H} NMR (282 MHz, $CDCl_3$) δ (ppm) = –62.20, –62.85, –62.95.

HRMS (ESI$^+$) *m/z*: calculated for $C_{66}H_{58}F_{15}NO_8P$ $[M+NH_4]^+$: 1308.3656; found: 1308.3630.

IR (neat) ν (cm^{-1}): 2940, 2832, 1502, 1279, 1213, 1135, 1121, 1044.

Melting point: 167-169 °C.

A1-(bis(3,5-bis(trifluoromethyl)phenyl)phosphine)-A2-(4-(*tert*-butyl)phenyl)-pillar[5]arene (9fc)

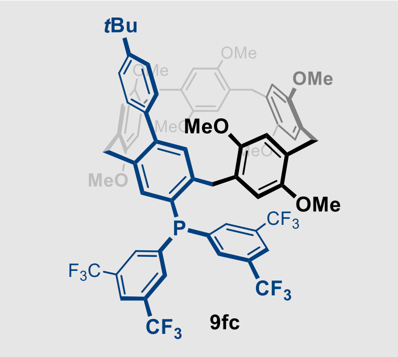

Synthesized following General Procedures IV and V, using **5f•O** (0.15 mmol, 1.0 equiv.) and **8c** (0.61 mmol, 4.0 equiv.). Purification by flash column chromatography over silica gel (eluent: *n*-pentane/CH_2Cl_2/Et_2O = 20:1:1) afforded **9fc** as a white solid. (119 mg, 60% yield over two steps).

TLC: 0.5, *n*-pentane/CH_2Cl_2/Et_2O = 20:1:1

^{1}H NMR (400 MHz, $CDCl_3$) δ (ppm) = 7.94 (s, 1H), 7.90 (s, 1H), 7.75 (d, $J_{P\text{-}H}$ = 6.7 Hz, 2H), 7.66 (d, $J_{P\text{-}H}$ = 6.0 Hz, 2H), 7.34 (d, $J_{H\text{-}H}$ = 8.4 Hz, 2H), 7.24 (d, $J_{P\text{-}H}$ = 5.6 Hz, 1H), 7.00 (d, $J_{H\text{-}H}$ = 8.2 Hz, 2H), 6.87 (s, 1H), 6.84 (s, 1H), 6.83 (d, $J_{P\text{-}H}$ = 5.3 Hz, 1H), 6.79 (s, 1H), 6.75 (s, 1H), 6.74 (s, 1H), 5.45 (s, 1H), 4.16 (dd, $J_{H\text{-}H}$ = 14.2 Hz, $J_{P\text{-}H}$ = 4.4 Hz, 1H), 3.96 (dd, $J_{H\text{-}H}$ = 13.7 Hz, $J_{P\text{-}H}$ = 4.0 Hz, 2H), 3.92 – 3.80 (m, 3H), 3.74 (s, 4H), 3.73 (s, 3H), 3.71 (m, 1H), 3.67 (s, 4H), 3.66 (s, 4H), 3.64 (s, 3H), 3.35 (s, 3H), 3.23 (s, 3H), 3.09 (s, 3H), 1.37 (s, 9H).

^{13}C{^{1}H} NMR (101 MHz, $CDCl_3$) δ (ppm) = 151.4 (C), 151.1 (C), 151.1 (C), 150.9 (C), 150.8 (C), 150.5 (C), 150.0 (C), 145.1 (C), 144.8 (C), 144.0 (C), 140.8 (C), 140.6 (C), 140.1 (C), 139.9 (C), 139.0 (C), 137.8 (C), 137.8 (C), 135.9 (CH), 134.7 (CH), 134.5 (bs, CH), 133.4 (bs, CH), 133.4 (CH), 132.9 (bs, CH), 132.8 (bs, CH), 132.4 (C), 132.3 (C), 132.3 (C), 132.3 (C), 132.0 (C), 132.0 (C), 132.0 (C), 131.9 (C), 131.7 (C), 131.7 (C), 131.7 (C), 131.6 (C), 129.2 (CH), 129.1 (C), 128.9 (C), 128.6 (C), 128.2 (C), 127.9 (C), 127.5 (C), 127.4 (C), 127.36 (C), 127.1 (C), 125.3 (CH), 123.3 (CH), 123.2 (q, $J_{C\text{-}F}$ = 273.0 Hz) (C), 123.2 (q, $J_{C\text{-}F}$ = 273.0 Hz) (C), 123.1 (bs, CH), 123.0 (bs, CH)114.6 (CH), 114.5 (CH), 114.4 (CH), 114.3 (CH), 114.2 (CH), 113.8 (CH), 113.6 (CH), 113.5 (CH), 113.0 (CH), 56.1 (CH_3), 56.0 (CH_3), 55.8 (CH_3), 55.7 (CH_3), 55.6 (CH_3), 55.5 (CH_3), 55.4 (CH_3), 34.7 (C), 34.1 (CH_2), 33.9 (CH_2), 32.9 (CH_2), 31.5 (CH_3), 30.3 (CH_2), 29.6 (CH_2), 29.5 (CH_2).

^{31}P{^{1}H} NMR (162 MHz, $CDCl_3$) δ (ppm) = –11.44.

^{19}F{^{1}H} NMR (282 MHz, $CDCl_3$) δ (ppm) = –62.84, –62.96.

HRMS (ESI$^+$) *m/z*: calculated for $C_{69}H_{64}F_{12}O_8P$ [M+H]$^+$: 1279.4147; found: 1279.4119.

IR (neat) ν (cm^{-1}): 2945, 2828, 1502, 1276, 1210, 1172, 1131, 1043.

Melting point: 196-198 °C.

A1-(bis(3,5-bis(trifluoromethyl)phenyl)phosphine)-A2-(4-(methoxy)phenyl)-ethoxy-pillar[5]arene (10fa)

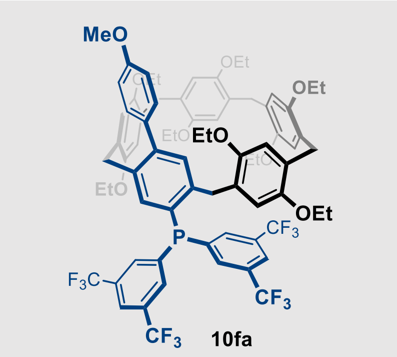

Synthesized following General Procedures IV and V, using **7f•O** (0.14 mmol, 1.0 equiv.) and **8a** (0.56 mmol, 4.0 equiv.). Purification by flash column chromatography over silica gel (eluent: *n*-pentane/CH_2Cl_2/Et_2O = 30:1:1) afforded **10fa** as a white solid. (146 mg, 75% yield over 2 steps).

TLC: 0.55, *n*-pentane/CH_2Cl_2/Et_2O = 30:1:1

^{1}H NMR (400 MHz, $CDCl_3$) δ (ppm) = 7.94 (s, 1H), 7.89 (s, 1H), 7.83 (d, $J_{P\text{-}H}$ = 6.6 Hz, 2H), 7.70 (d, $J_{P\text{-}H}$ = 6.2 Hz, 2H), 6.91 – 6.83 (m, 3H), 6.81 (s, 1H), 6.80 (s, 1H), 6.73 (s, 1H), 6.66 (d, $J_{H\text{-}H}$ = 8.9 Hz, 2H), 6.62 (d, $J_{H\text{-}H}$ = 8.8 Hz, 3H), 6.56 (s, 1H), 6.53 (s, 1H), 5.44 (s, 1H), 4.24 (dd, $J_{H\text{-}H}$ = 14.4 Hz, $J_{P\text{-}H}$ = 5.3 Hz, 1H), 4.04 – 3.85 (m, 10H), 3.84 (s, 3H), 3.83 – 3.63 (m, 8H), 3.63 – 3.54 (m, 2H), 3.50 – 3.35 (m, 5H), 1.44 (t, $J_{H\text{-}H}$ = 7.0 Hz, 3H), 1.40 (t, $J_{H\text{-}H}$ = 7.0 Hz, 3H), 1.35 (t, $J_{H\text{-}H}$ = 7.0 Hz, 3H), 1.20 (d, $J_{H\text{-}H}$ = 7.0 Hz, 3H), 1.17 (d, $J_{H\text{-}H}$ = 7.0 Hz, 3H), 1.14 (d, $J_{H\text{-}H}$ = 7.0 Hz, 3H), 0.85 (t, $J_{H\text{-}H}$ = 7.0 Hz, 3H), 0.77 (t, $J_{H\text{-}H}$ = 6.9 Hz, 3H).

^{13}C{^{1}H} NMR (101 MHz, $CDCl_3$) δ (ppm) = 158.6 (C), 150.2 (C), 150.1 (C), 150.1 (C), 149.8 (C), 149.7 (C), 149.6 (C), 149.6 (C), 145.6 (C), 145.4 (C), 144.3 (C), 141.2 (C), 141.1 (C), 140.1 (C), 139.9 (C), 138.4 (C), 138.4 (C), 135.6 (CH), 134.6 (CH), 134.6 (CH), 134.4 (CH), 134.4 (CH), 134.2 (C), 133.3 – 132.6 (m) (CH), 132.5 (C), 132.4 (C), 132.3 (C), 132.1 (C), 132.1 (C), 132.0 (C), 131.7, 130.0 (CH), 129.8 (C), 129.2 (C), 129.1 (C), 129.0 (C), 128.7 (C), 128.5 (C), 127.3 (C), 127.0 (C), 123.4 (CH), 123.2 (q, $J_{C\text{-}F}$ = 273.0 Hz) (C), 123.2 (q, $J_{C\text{-}F}$ = 273.0 Hz) (C), 123.1 (CH), 115.7 (CH), 115.4 (CH), 115.3 (CH), 115.1 (CH), 114.7 (CH), 114.7 (CH), 114.1 (CH), 113.3 (CH), 64.2 (CH_2), 64.2 (CH_2), 64.2 (CH_2), 64.0 (CH_2), 63.9 (CH_2), 63.8 (CH_2), 63.5 (CH_2), 63.3 (CH_2), 55.3 (CH_3), 34.2 (CH_2), 34.1 (CH_2), 33.9 (CH_2), 30.5 (CH_2), 29.7 (CH_2), 29.5 (CH_2), 15.4 (CH_3), 15.4 (CH_3), 15.3 (CH_3), 15.1 (CH_3), 15.0 (CH_3), 14.9 (CH_3), 14.7 (CH_3), 14.5 (CH_3).

^{31}P{^{1}H} NMR (162 MHz, $CDCl_3$) δ (ppm) = –11.38.

^{19}F{^{1}H} NMR ($CDCl_3$, 282 MHz) δ (ppm) = –62.91, –62.94.

HRMS (ESI$^+$) *m/z*: calculated for $C_{74}H_{77}F_{12}NO_9P$ $[M+NH_4]^+$: 1382.5139; found: 1382.5209.

IR (neat) ν (cm^{-1}): 2977, 2931, 1503, 1277, 1205, 1175, 1125, 1048.

Melting point: 151-152 °C.

7. Synthesis of A1-(bis(3,5-bis(trifluoromethyl)phenyl)phosphine)-A2-(trifluoromethyl)phenyl-ethoxy-pillar[6]arene (13fa)

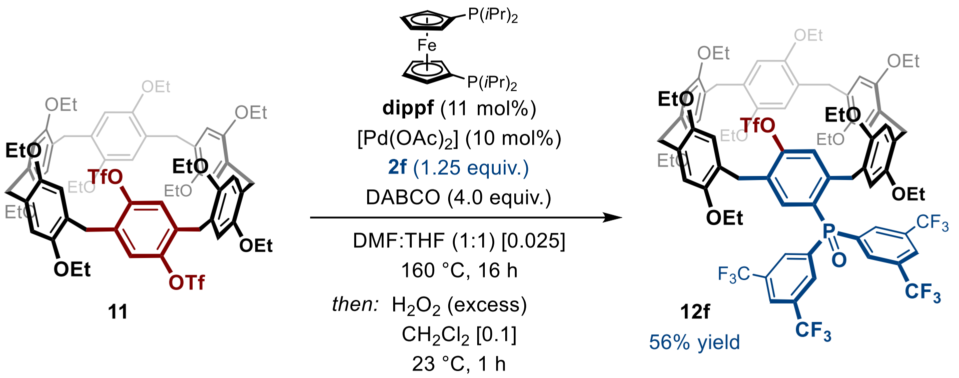

Figure S10. Synthesis of A1-Phosphine-oxide-A2-triflate-pillar[6]arene **12f**

Step 1: In a glovebox, in an oven dried J-Young flask, [Pd(OAc)$_2$] (8.8 mg, 39 μmol, 10 mol%), dippf (18.4 mg, 43 μmol, 11 mol%), A1/A2-di-triflate-pillar[6]arene **11** (250 mg, 0.2 mmol, 1.0 equiv.) and **2f** (118 mg, 0.25 mmol, 1.25 equiv.) were dissolved in a 1:1 mixture of DMF:THF (8 mL). The reaction mixture was stirred vigorously for 5 min., next DABCO (66 mg, 0.59 mmol, 3.0 equiv.) was added. The J-Young flask was closed, taken out of the glovebox and placed in a preheated oil bath and stirred at 150 °C. After 16 h, the reaction mixture was cooled to 23 °C, diluted with CH_2Cl_2 (30 mL) and washed with an aqueous LiCl solution (4 × 30 mL, 5% w/w). The combined organic phases were washed with a saturated aqueous NaCl solution (50 mL), dried over Na_2SO_4, filtered and concentrated under reduced pressure. The oxidation was conducted next. The residue was dissolved in CH_2Cl_2 (2 mL) and H_2O_2 (1 mL, 30% aq., 50 equiv.) was added. The resulting mixture was stirred vigorously. After 1 h, ^{31}P NMR analysis indicated full conversion to the corresponding oxide. The aqueous layer was extracted with CH_2Cl_2 (3 × 20 mL) and the combined organic phases were dried over Na_2SO_4, filtered and concentrated under reduced pressure. The residue was purified by flash column chromatography over silica gel (eluent: CH_2Cl_2/Et_2O = 98:2) to afford **12f** as a white solid (171 mg, 56% yield).

A1-(bis(3,5-bis(trifluoromethyl)phenyl)phosphine-oxide)-A2-triflate-ethoxy-pillar[6]arene (12f)

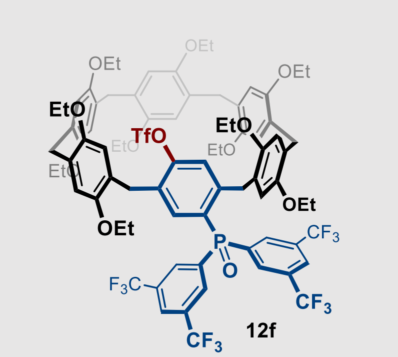

TLC: 0.4, CH_2Cl_2/Et_2O = 95:5

^{1}H NMR (400 MHz, $CDCl_3$) δ (ppm) = 8.14 (t, $J_{P\text{-}H}$ = 10.6 Hz, 4H), 8.12 (s, 2H), 7.24 (d, $J_{P\text{-}H}$ = 14.8 Hz, 1H), 7.01 (d, $J_{P\text{-}H}$ = 3.7 Hz, 1H), 6.82 (s, 1H), 6.77 (d, J = 2.8 Hz, 2H), 6.74 (d, J = 3.6 Hz, 2H), 6.71 (d, J = 4.7 Hz, 2H), 6.67 (d, J = 3.7 Hz, 2H), 6.48 (s, 1H), 4.04 – 4.01 (m, 2H), 3.94 – 3.86 (m, 9H), 3.86 – 3.82 (m, 7H), 3.80 (s, 2H), 3.79 – 3.74 (m, 7H), 3.74 – 3.65 (m, 5H), 1.41 – 1.38 (m, 3H), 1.38 – 1.37 (m, 2H), 1.37 – 1.36 (m, 4H), 1.34 (s, 6H), 1.33 – 1.32 (m, 3H), 1.31 – 1.30 (m, 2H), 1.29 – 1.27 (m, 3H), 1.03 (t, J = 6.9 Hz, 3H), 0.89 (t, J = 7.0 Hz, 4H).

^{13}C{^{1}H} NMR (101 MHz, $CDCl_3$) δ (ppm) = 151.4 (C), 151.4 (C), 149.8 (C), 149.7 (C), 149.6 (C), 149.4 (C), 149.4 (C), 149.3 (C), 149.3 (C), 148.9 (C), 148.8 (C), 147.1 (C), 147.0 (C), 136.6 (CH), 136.4 (CH), 134.8 (C), 133.8 (C), 132.4 (C), 132.3 (C), 132.0 (C), 132.0 (C), 131.8 (q, $J_{C\text{-}F}$ = 34.2), 131.8 (q, $J_{C\text{-}F}$ = 34.2), 131.7 (C), 131.6 (C), 131.4 (C), 131.2 (C), 130.7 (CH, bs), 130.6 (C), 130.5 (C), 128.7 (C), 128.0 (C), 127.0 (C), 127.0 (C), 126.7 (C), 126.7 (C), 126.0 (C), 126.0 (C), 125.5 (CH, bs), 125.0 (C), 123.9 (C), 123.4 (CH), 123.3 (CH), 121.8 (C), 120.7 (q, $J_{C\text{-}F}$ = 320.5 Hz) (C), 121.5 (q, J = 272.4 Hz), 114.7 (CH), 114.4 (CH), 114.4 (CH), 114.2 (CH), 114.2 (CH), 114.0 (CH), 113.8 (CH), 113.7 (CH), 113.5 (CH), 113.2 (CH), 63.0 (CH_2), 63.0 (CH_2), 62.9 (CH_2), 62.8 (CH_2), 62.8 (CH_2), 62.6 (CH_2), 62.5 (CH_2), 62.5 (CH_2), 62.3 (CH_2), 33.6 (CH_2), 33.6 (CH_2), 32.5 (CH_2), 30.9 (CH_2), 30.4 (CH_2), 30.1 (CH_2), 28.7 (CH_2), 21.7 (CH_3), 14.2 (CH_3), 14.2 (CH_3), 14.2 (CH_3), 14.1 (CH_3), 14.1 (CH_3), 14.0 (CH_3), 13.8 (CH_3), 13.4 (CH_3).

^{31}P{^{1}H} NMR (162 MHz, $CDCl_3$) δ (ppm) = 25.94.

^{19}F{^{1}H} NMR ($CDCl_3$, 282 MHz) δ (ppm) = –63.93, –73.53.

HRMS (ESI$^+$) *m/z*: calculated for $C_{79}H_{81}F_{15}O_{14}PS$ $[M+H]^+$: 1601.4845; found: 1601.4801.

IR (neat) ν (cm^{-1}): 2978, 2927, 1503, 1411, 1278, 1206, 1133, 1049.

Melting point: 89-93°C.

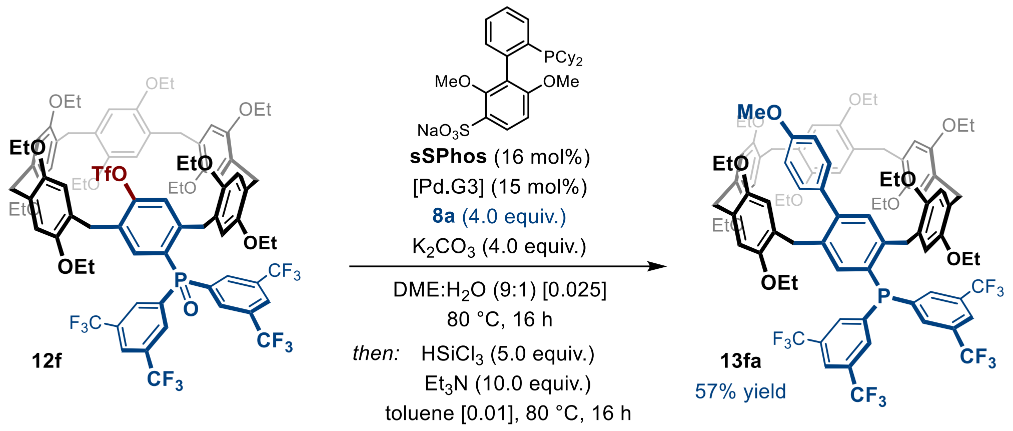

Figure S11. Synthesis of A1-Phosphine-oxide-A2-aryl-pillar[6]arene **13fa**

Step 2: In a glovebox, in an oven dried J-Young flask [Pd.G$_3$] (4.7 mg, 6.7 µmol, 15 mol%), sSPhos (7.2 mg, 14 µmol, 16 mol%), **12f** (140 mg, 87 µmol, 1.0 equiv.), **8a** (53 mg, 0.35 mmol, 4.0 equiv.) were dissolved in 1,2-dimethoxyethane (3.5 mL). The reaction mixture was stirred vigorously. After 5 min., K_2CO_3 (48 mg, 0.35 mmol, 4.0 equiv.) was added. The J-Young flask was closed, taken out of the glovebox and attached to a two manifold Schlenk line. Degassed H_2O (0.4 mL) was added. Next, the J-Young flask placed in a preheated oil bath and stirred at 80 °C. After 16 h, the reaction mixture was cooled to 23 °C, diluted with H_2O (40 mL) and extracted with CH_2Cl_2 (3 × 20 mL). The combined organic phases were washed with saturated aqueous NaCl solution (50 mL), dried over Na_2SO_4, filtered and concentrated under reduced pressure. The reduction was conducted next. The residue was dissolved in toluene (1 mL) and transferred into a J-Young flask. $HSiCl_3$ (32 µL, 0.32 mmol, 5.0 equiv.) and Et_3N (58 µL, 0.64 mmol, 10 equiv.) were added in sequence. The J-Young flask was next placed in a pre-heated oil bath and stirred at 80 °C. After 16 h, ^{31}P NMR analysis indicated full consumption of the starting material. The J-Young flask was cooled to room temperature and then placed in an ice/water bath. After 10 min., an aqueous NaOH solution (4.0 M) in H_2O was added dropwise (*ca.* 15 mL). The mixture was diluted with H_2O (50 mL) and extracted with CH_2Cl_2 (3 × 20 mL). The combined organic phases were dried over Na_2SO_4, filtered and concentrated under reduced pressure. The residue was purified by flash column chromatography over silica gel (eluent: *n*-pentane/CH_2Cl_2/Et_2O = 12:1:1) to afford **13fa** as a white solid (77 mg, 57% yield).

A1-(bis(3,5-bis(trifluoromethyl)phenyl)phosphine)-A2-triflate-ethoxy-pillar[6]arene (13fa)

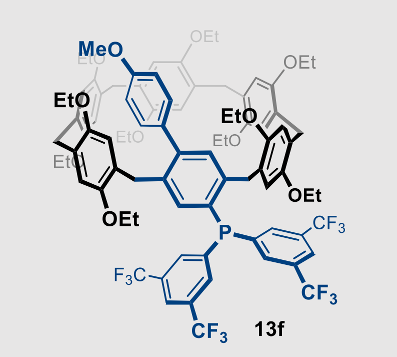

TLC: 0.5, *n*-pentane/CH_2Cl_2/Et_2O = 12:1:1

^{1}H NMR (400 MHz, $CDCl_3$) δ (ppm) = 7.69 (s, 1H), 7.55 (s, 1H), 7.47 (d, $J_{P\text{-}H}$ = 6.2 Hz, 2H), 7.41 (d, $J_{P\text{-}H}$ = 6.2 Hz, 2H), 6.83 (d, $J_{P\text{-}H}$ = 5.5 Hz, 1H), 6.69 (d, $J_{P\text{-}H}$ = 8.5 Hz, 2H), 6.65 (d, $J_{P\text{-}H}$ = 4.9 Hz, 1H), 6.63 (s, 1H), 6.61 (s, 1H), 6.59 (s, 1H), 6.58 (s, 1H), 6.47 (s, 1H), 6.45 (d, $J_{H\text{-}H}$ = 8.7 Hz, 2H), 6.43 (s, 1H), 6.36 (s, 1H), 6.35 (s, 1H), 6.32 (s, 1H), 5.39 (s, 1H), 4.20 (dd, $J_{H\text{-}H}$ = 14.8, 5.2 Hz, 1H), 3.76 – 3.64 (m, 9H), 3.64 – 3.56 (m, 11H), 3.55 (s, 4H), 3.48 (s, 4H), 3.40 – 3.31 (m, 2H), 3.31 – 3.25 (m, 1H), 3.25 – 3.17 (m, 3H), 1.20 – 1.10 (m, 14H), 1.06 – 0.99 (m, 10H), 0.93 (d, $J_{H\text{-}H}$ = 6.8 Hz, 3H), 0.63 (d, $J_{H\text{-}H}$ = 6.8 Hz, 3H).

^{13}C{^{1}H} NMR (101 MHz, $CDCl_3$) δ (ppm) = 158.7 (C), 150.7 (C), 150.6 (C), 150.6 (C), 150.6 (C), 150.5 (C), 150.4 (C), 150.4 (C), 150.4 (C), 150.0 (C), 144.9 (C), 144.6 (C), 144.4 (C), 141.3 (C), 141.1 (C), 139.9 (C), 139.7 (C), 137.9 (C), 136.2 (CH), 133.9 (C), 133.8 (CH, bs), 133.6 (CH, bs), 133.2 (CH, bs), 133.0 (CH, bs), 132.9 (CH), 132.8 (CH), 132.3 (C), 132.3 (C), 132.2 (C), 132.1 (C), 132.0 (C), 131.9 (C), 131.9 (C), 131.8 (C), 130.4 CH), 129.1 (C), 128.2 (C), 128.1 (C), 128.1 (C), 128.0 (C), 128.0 (C), 127.9 (C), 127.8 (C), 127.7 (C), 127.0 (C), 126.1 (C), 123.7 (q, $J_{C\text{-}F}$ = 274.5) (C), 123.7 (q, $J_{C\text{-}F}$ = 274.5) (C), 123.0 (CH, bs), 116.0 (CH), 115.5 (CH), 115.5 (CH), 115.4 (CH), 115.3 (CH), 115.1 (CH), 114.5 (CH), 114.5 (CH), 113.6 (CH), 113.2 (CH), 64.3 (CH_2), 64.1 (CH_2), 64.1 (CH_2), 64.1 (CH_2), 64.0 (CH_2), 63.9 (CH_2), 63.9 (CH_2), 63.8 (CH_2), 63.4 (CH_2), 34.9 (CH_2), 34.7 (CH_2), 34.4 (CH_2), 31.3 (CH_2), 31.3 (CH_2), 30.9 (CH_2), 30.8 (CH_2), 29.9 (CH_2), 15.4 (CH_3), 15.4 (CH_3), 15.3 (CH_3), 15.30 (CH_3), 15.1 (CH_3), 15.07 (CH_3), 14.8 (CH_3).

^{31}P{^{1}H} NMR (162 MHz, $CDCl_3$) δ (ppm) = –11.80.

^{19}F{^{1}H} NMR ($CDCl_3$, 282 MHz) δ (ppm) = –62.89, –63.05.

HRMS (ESI^+) *m/z*: calculated for $C_{85}H_{91}F_{12}NO_{11}P$ $[M+NH_4]^+$: 1560.6138; found: 1560.6198.

IR (neat) ν (cm^{-1}): 2977, 2902, 1611, 1503, 1437, 1409, 1390.

Melting point: 96-99°C.

8. Syntheses of rim-differentiated A1/A2-di-phosphine-pillar[5]arenes

8.1 General Procedure VI (GP-VI) for sequential phosphinylation

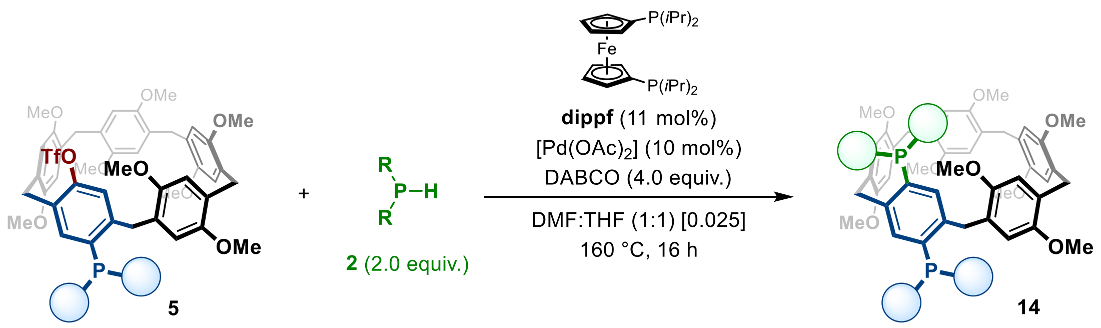

In a glovebox, in an oven-dried J-Young flask, [Pd(OAc)$_2$] (2.3 mg, 10 μmol, 10 mol%), dippf (4.8 mg, 11 μmol, 11 mol%), the appropriate A1-phosphine-A2-triflate-pillar[5]arene **5** (0.1 mmol, 1.0 equiv.) and the secondary phosphine of choice **2** (0.2 mmol, 2.0 equiv.) were dissolved in a 1:1 mixture of DMF:THF (4 mL). Next, DABCO (45.5 mg, 0.4 mmol, 4.0 equiv.) was added. The J-Young flask was closed, taken out of the glovebox, placed in a preheated oil bath and stirred at 160 °C. After 16 h, the reaction mixture was cooled to 23 °C and diluted with saturated aqueous NaCl solution (30 mL). The aqueous layer was extracted with EtOAc (3 × 20 mL). The combined organic phases were washed with an aqueous LiCl solution (4 × 20 mL, 5% w/w), dried over Na_2SO_4, filtered and concentrated under reduced pressure. The residue was purified by flash column chromatography over silica gel using *n*-pentane/CH_2Cl_2/Et_2O as eluent to afford rim-differentiated A1/A2-di-phosphine-pillar[5]arene **14**.

A1-(bis(4-methoxyphenyl)phosphine)-A2-(bis(3,5-bis(trifluoromethyl)phenyl)-phosphine)-pillar[5]arene (14a)

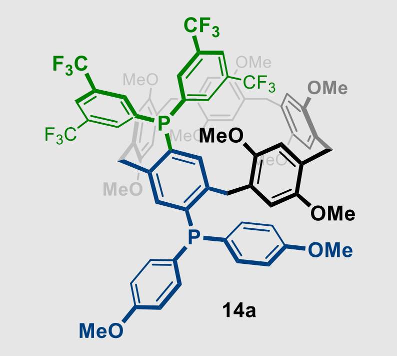

Synthesized following General Procedure VI, using **5a** (0.07 mmol, 1.0 equiv.) and **2f** (0.14 mmol, 2.0 equiv.). Purification by flash column chromatography over silica gel (eluent: *n*-pentane/CH_2Cl_2/Et_2O = 10:1:1) afforded **14a** as a light orange solid. (87 mg, 85% yield). Single crystals suitable for X-ray analysis were obtained by slow evaporation of a mixture of CH_2Cl_2: *n*-hexane (1: 2).

TLC: 0.35, *n*-pentane/CH_2Cl_2/Et_2O = 10:1:1

^{1}H NMR (400 MHz, $CDCl_3$) δ (ppm) = 7.96 (s, 1H), 7.90 (s, 1H), 7.68 (d, $J_{P\text{-}H}$ = 6.7 Hz, 2H), 7.58 (d, $J_{P\text{-}H}$ = 5.9 Hz, 2H), 7.18 (dd, $J_{H\text{-}H}$ = 8.6 Hz, $J_{P\text{-}H}$ = 7.2 Hz, 2H), 7.15 – 7.11 (m, 2H), 7.01 – 6.96 (m, 3H), 6.92 (dd, $J_{H\text{-}H}$ = 7.9 Hz, $J_{P\text{-}H}$ = 0.8 Hz, 2H), 6.90 (s, 1H), 6.86 (s, 1H), 6.84 (s, 1H), 6.84 (s, 1H), 6.82 (d, $J_{P\text{-}H}$ = 4.9 Hz, 1H), 6.78 (dd, $J_{H\text{-}H}$ = 7.7 Hz, $J_{P\text{-}H}$ = 0.8 Hz, 2H), 6.58 (s, 1H), 6.56 (s, 1H), 4.11 (dd, $J_{H\text{-}H}$ = 13.3 Hz, $J_{P\text{-}H}$ = 2.6 Hz, 1H), 4.02 (dd, $J_{H\text{-}H}$ = 13.6 Hz, $J_{P\text{-}H}$ = 3.5 Hz, 1H), 3.86 (s, 2H), 3.86 (s, 3H), 3.85 (s, 3H), 3.83 (s, 1H), 3.77 (s, 4H), 3.74 (s, 4H), 3.73 (s, 4H), 3.72 (s, 4H), 3.69 (s, 4H), 3.11 (s, 4H), 3.10 (s, 3H).

^{13}C{^{1}H} NMR (101 MHz, $CDCl_3$) δ (ppm) = 160.4 (C), 160.1 (C), 151.2 (C), 151.1 (C), 151.0 (C), 150.9 (C), 150.9 (C), 150.7 (C), 150.6 (C), 150.6 (C), 150.5 (C), 145.3 (C), 145.1 (C), 144.4 (C), 144.2 (C), 140.4 (C), 140.3 (C), 139.9 (C), 139.8 (C), 139.7 (C), 139.6 (C), 136.8 (C), 136.8 (C), 135.8 (CH), 135.6 (CH), 135.2 (CH), 135.2 (CH), 135.0 (CH), 134.7 (CH), 134.7 (CH), 134.6 (CH), 134.5 (CH), 134.4 (CH), 132.8 (CH, bs), 132.7 (CH), 132.6 (CH), 132.6 (C), 132.4 (C), 132.4 (C), 132.0 (C), 132.0 (C), 131.7 (C), 131.7 (C), 131.7 (C), 131.6 (C), 130.3 (C), 130.2 (C), 128.9 (C), 128.9 (C), 128.8 (C), 128.7 (C), 128.7 (C), 128.6 (C), 128.0 (C), 127.7 (C), 127.6 (C), 127.6 (C), 127.5 (C), 127.1 (C), 123.3 (CH, bs), 123.2 (q, J = 273.3 Hz) (C), 123.1 (q, J = 273.3 Hz) (C), 114.9 (CH), 114.5 (CH), 114.4 (CH), 114.3 (CH), 114.2 (CH), 114.2 (CH), 114.1 (CH), 114.1 (CH), 113.9 (CH), 113.6 (CH), 113.2 (CH), 113.1 (CH), 56.0 (CH_3), 55.9 (CH_3), 55.8 (CH_3), 55.7 (CH_3), 55.6 (CH_3), 55.4 (CH_3), 55.4 (CH_3), 55.3 (CH_3), 55.3 (CH_3), 33.9 (CH_2), 33.7 (CH_2), 33.4 (CH_2), 33.3 (CH_2), 30.0 (CH_2), 29.4 (CH_2).

^{31}P{^{1}H} NMR (162 MHz, $CDCl_3$) δ (ppm) = –10.06, –15.16.

^{19}F{^{1}H} NMR (282 MHz, $CDCl_3$) δ (ppm) = –62.90, –62.95.

HRMS (ESI$^+$) *m/z*: calculated for $C_{73}H_{65}F_{12}O_{10}P_2$ $[M+H]^+$: 1391.3857; found: 1391.3857.

IR (neat) ν (cm^{-1}): 2927, 2887, 1497, 1398, 1209, 1175, 1045. **Melting point:** 181-183 °C.

A1-(bis(4-methoxyphenyl)phosphine)-A2-(dicyclohexylphosphine)-pillar[5]arene (14b)

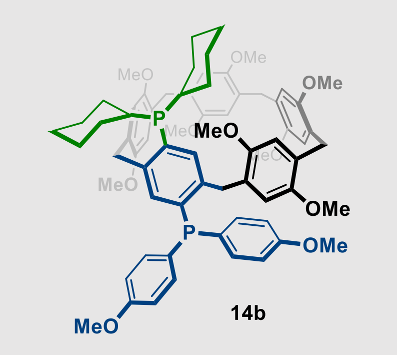

Synthesized following General Procedure VI, using **5a** (0.09 mmol, 1.0 equiv.) and **2d** (0.18 mmol, 2.0 equiv.). Purification by flash column chromatography over silica gel (eluent: *n*-pentane/CH_2Cl_2/Et_2O = 8:1:1) afforded **14b** as a white solid. (62 mg, 59% yield).

TLC: 0.4, *n*-pentane/CH_2Cl_2/Et_2O = 8:1:1

^{1}H NMR (400 MHz, $CDCl_3$) δ (ppm) = 7.46 (dd, $J_{P\text{-}H}$ = 4.9, 2.9 Hz, 1H), 7.14 (dd, $J_{H\text{-}H}$ = 8.7 Hz, $J_{P\text{-}H}$ = 7.0 Hz, 2H), 7.08 – 7.00 (m, 2H), 6.93 (d, $J_{P\text{-}H}$ = 1.1 Hz, 1H), 6.89 – 6.82 (m, 5H), 6.79 (m, 3H), 6.77 (s, 1H), 6.76 (s, 2H), 6.50 (s, 1H), 4.51 (dd, $J_{H\text{-}H}$ = 13.4 Hz, $J_{P\text{-}H}$ = 4.6 Hz, 1H), 4.16 (dd, $J_{H\text{-}H}$ = 13.8 Hz, $J_{P\text{-}H}$ = 3.4 Hz, 1H), 3.86 – 3.82 (m, 2H), 3.81 (s, 4H), 3.81 (s, 4H), 3.80 – 3.72 (m, 4H), 3.70 (s, 3H), 3.69 (s, 3H), 3.66 (s, 6H), 3.60 (s, 3H), 3.48 (s, 3H), 3.44 (s, 3H), 3.24 (s, 3H), 2.00 – 1.46 (m, 9H), 1.41 – 0.78 (m, 12H), 0.66 – 0.43 (m, 1H).

^{13}C{^{1}H} NMR (101 MHz, $CDCl_3$) δ (ppm) = 160.1 (C), 160.1 (C), 151.1 (C), 151.0 (C), 151.00 (C), 150.8 (C), 150.8 (C), 150.8 (C), 150.4 (C), 146.0 (C), 145.7 (C), 142.6 (C), 142.5 (C), 136.2 (CH), 136.0 (CH), 135.8 (C), 135.6 (C), 135.2 (CH), 135.1, (CH) 134.9 (CH), 134.7 (C), 134.2 (C), 134.0 (C), 129.5 (C), 129.4 (C), 128.9 (C), 128.8 (C), 128.6 (C), 128.6 (C), 128.5 (C), 128.4 (C), 128.3 (C), 128.2 (C), 128.0 (C), 115.1 (CH), 115.0 (CH), 114.4 (CH), 114.3 (CH), 114.2 (CH), 114.1 (CH), 114.0 (CH), 113.9 (CH), 113.2 (CH), 56.2 (CH_3), 56.2 (CH_3), 56.0 (CH_3), 55.9 (CH_3), 55.9 (CH_3), 55.8 (CH_3), 55.6 (CH_3), 55.4 (CH_3), 55.3 (CH_3), 55.2 (CH_3), 36.3 (CH), 36.2 (CH), 33.9 (CH_2), 33.7 (CH_2), 33.2 (CH_2), 33.1 (CH_2), 32.8 (CH), 32.7 (CH), 31.3 (CH_2), 31.1 (CH_2), 30.7 (CH_2), 30.6 (CH_2), 30.4 (CH_2), 30.0 (CH_2), 29.9 (CH_2), 29.8 (CH_2), 29.7 (CH_2), 28.2 (CH_2), 28.2 (CH_2), 27.7 (CH_2), 27.6 (CH_2), 27.5 (CH_2), 27.4 (CH_2), 27.2 (CH_2), 27.1 (CH_2), 26.9 (CH_2), 26.7 (CH_2), 26.5 (CH_2), 26.4 (CH_2).

^{31}P{^{1}H} NMR (162 MHz, $CDCl_3$) δ (ppm) = –14.53, –15.18.

HRMS (ESI$^+$) *m/z*: calculated for $C_{69}H_{81}O_{10}P_2$ $[M+H]^+$: 1131.5300; found: 1131.5216.

IR (neat) ν (cm^{-1}): 2927, 2827, 1497, 1462, 1398, 1209, 1175, 1045.

Melting point: 140-142 °C.

A1-(dicyclohexylphosphine)-A2-(bis(3,5-bis(trifluoromethyl)phenyl)phosphine)-pillar[5]arene (14c)

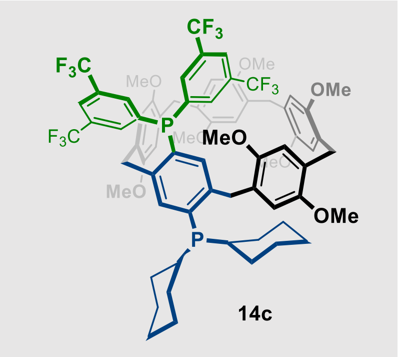

Synthesized following General Procedures VI and V, using **5d•O** (24 μmol, 1.0 equiv.) and **2d** (48 μmol, 2.0 equiv.). Purification by flash column chromatography over silica gel (eluent: *n*-pentane/CH_2Cl_2/Et_2O = 10:1:1) afforded **14c** as a white solid (18 mg, 45% yield over two steps).

TLC: 0.35, *n*-pentane/CH_2Cl_2/Et_2O = 10:1:1

^{1}H NMR (400 MHz, $CDCl_3$) δ (ppm) = 7.97 (s, 1H), 7.87 (s, 1H), 7.74 (d, $J_{P\text{-}H}$ = 6.9 Hz, 2H), 7.66 (dd, $J_{P\text{-}H}$ = 5.8, 2.6 Hz, 1H), 7.56 (d, $J_{P\text{-}H}$ = 5.7 Hz, 2H), 7.31 (d, $J_{P\text{-}H}$ = 2.4 Hz, 1H), 6.92 (s, 1H), 6.86 (s, 1H), 6.84 (s, 1H), 6.83 (s, 1H), 6.79 (s, 1H), 6.75 (dd, $J_{P\text{-}H}$ = 5.5, 4.0 Hz, 1H), 6.72 (s, 1H), 6.48 (s, 1H), 4.43 (dd, $J_{P\text{-}H}$ = 12.9, $J_{H\text{-}H}$ = 3.6 Hz, 1H), 4.04 – 3.96 (m, 2H), 3.84 (s, 1H), 3.81 (s, 3H), 3.77 (s, 1H), 3.75 (s, 4H), 3.72 (s, 9H), 3.68 (s, 6H), 3.64 – 3.58 (m, 1H), 3.56 (s, 3H), 3.03 (s, 3H), 1.96 – 1.61 (m, 8H), 1.58 – 1.47 (m, 2H), 1.24 – 0.93 (m, 8H), 0.88 – 0.83 (m, 1H), 0.79 – 0.68 (m, 1H), 0.65 – 0.55 (m, 1H), 0.45 – 0.30 (m, 1H).

^{13}C{^{1}H} NMR (101 MHz, $CDCl_3$) δ (ppm) = 150.9 (C), 150.9 (C), 150.7 (C), 150.5 (C), 150.4 (C), 150.3 (C), 150.1 (C), 147.1 (C), 146.8 (C), 143.3 (C), 143.1 (C), 140.7 (C), 140.5 (C), 139.3 (C), 139.1 (C), 137.6 (C), 137.3 (C), 135.6 (CH), 135.4 (CH), 135.2 (CH), 134.6 (CH), 134.6 (CH), 132.5 (CH), 132.3 (CH), 132.2 (C), 132.1 (C), 131.9 (C), 131.9 (C), 131.8 (C), 131.5 (C), 129.1 (C), 129.0 (C), 128.9 (C), 128.8 (C), 128.6 (C), 128.5 (C), 128.2 (C), 127.7 (C), 127.4 (C), 127.1 (C), 123.5 (CH), 123.0 (q, *J* = 273.0 Hz) (C), 123.0 (q, *J* = 273.0 Hz) (C), 122.9 (CH), 114.9 (CH), 114.8 (CH), 114.6 (CH), 113.9 (CH), 113.8 (CH), 113.7 (CH), 113.6 (CH), 113.4 (CH), 113.0 (CH), 113.0 (CH), 56.0 (CH_3), 55.9 (CH_3), 55.7 (CH_3), 55.6(CH_3), 55.6 (CH_3), 55.5 (CH_3), 55.3 (CH_3), 36.8 (CH), 36.7 (CH), 33.9 (CH_2), 33.8 (CH_2), 33.5 (CH_2), 33.3 (CH_2), 33.2 (CH), 33.1 (CH), 31.3 (CH_2), 31.2 (CH_2), 30.7 (CH_2), 30.5 (CH_2), 30.1 (CH_2), 29.7 (CH_2), 29.6 (CH_2), 29.4 (CH_2), 29.3 (CH_2), 29.1 (CH_2), 28.2 (CH_2), 28.2 (CH_2), 27.6 (CH_2), 27.4 (CH_2), 27.2 (CH_2), 27.2 (CH_2), 27.1 (CH_2), 27.0 (CH_2), 26.8 (CH_2), 26.3 (CH_2), 26.3 (CH_2), 26.2 (CH_2).

^{31}P{^{1}H} NMR (162 MHz, $CDCl_3$) δ (ppm) = –9.67, –14.19.

^{19}F{^{1}H} NMR (282 MHz, $CDCl_3$) δ (ppm) = –62.89, –63.05.

HRMS (ESI$^+$) *m/z*: calculated for $C_{71}H_{73}F_{12}O_8P_2$ $[M+H]^+$: 1345.4651; found: 1345.4681.

IR (neat) ν (cm^{-1}): 2942, 2853, 1502, 1462, 1356, 1278, 1211, 1042.

Melting point: 108-110 °C.

Synthesis of A1/A2-di-(3,5-bis(trifluoromethyl)phenyl)phosphine-pillar[5]arene (3f)

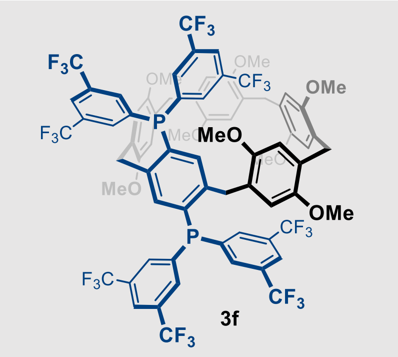

Synthesized following General Procedure VI, using **5f** (0.23 mmol, 1.0 equiv.) and **2f** (0.46 mmol, 2.0 equiv.). Purification by flash column chromatography over silica gel (eluent: *n*-pentane/CH_2Cl_2/Et_2O = 20:1:1) afforded **3f** as a light purple solid (299 mg, 80% yield).

TLC: 0.35, *n*-pentane/CH_2Cl_2/Et_2O = 20:1:1

^{1}H NMR (400 MHz, $CDCl_3$) δ (ppm) = 7.94 (s, 2H), 7.90 (s, 2H), 7.66 (d, J = 6.8 Hz, 4H), 7.60 (dd, $J_{P\text{-}H}$ = 6.4 Hz, $J_{H\text{-}H}$ = 1.5 Hz, 4H), 7.02 (ta, $J_{P\text{-}H}$ = 5.1 Hz, 2H), 6.94 (s, 2H), 6.84 (s, 2H), 6.79 (s, 2H), 6.46 (s, 2H), 4.00 (dd, $J_{P\text{-}H}$ = 13.8, 2.8 Hz, 2H), 3.89 – 3.79 (m, 4H), 3.77 (s, 6H), 3.75 (s, 6H), 3.73 – 3.68 (m, 4H), 3.56 (s, 6H), 2.97 (s, 6H).

^{13}C{^{1}H} NMR (101 MHz, $CDCl_3$) δ (ppm) = 151.2 (C), 151.0 (C), 150.8 (C), 150.7 (C), 146.1 (C), 145.8 (C), 140.2 (C), 140.0 (C), 138.9 (C), 138.7 (C), 136.6 (CH), 136.5 (CH), 134.6 (bs, CH), 134.4 (bs, CH), 133.3 (C), 133.2 (C), 133.1 (C), 133.0 (C), 132.7 (C), 132.7 (C), 132.5 (C), 132.5 (C), 132.4 (C), 132.4 (C), 132.3 (C), 132.2 (CH), 132.1 (CH), 132.1 (C), 132.0 (C), 131.8 (C), 131.8 (C), 129.6 (C), 128.7 (C), 127.3 (C), 126.3 (C), 123.6 (bs, CH), 123.0 (q, $J_{C\text{-}F}$ = 273.0 Hz) (C), 115.5 (CH), 114.0 (CH), 113.5 (CH), 112.9 (CH), 112.8 (CH), 55.7 (CH_3), 55.6 (CH_3), 55.5 (CH_3), 55.5 (CH_3), 34.6 (CH_2), 34.4 (CH_2), 29.6 (CH_2), 29.4 (CH_2).

^{31}P{^{1}H} NMR (162 MHz, $CDCl_3$) δ (ppm) = –11.23.

^{19}F{^{1}H} NMR (282 MHz, $CDCl_3$) δ (ppm) = –62.91, –63.29.

HRMS (ESI$^+$) *m/z*: calculated for $C_{75}H_{57}F_{24}O_8P_2$ $[M+H]^+$: 1603.3141; found: 1603.3083.

IR (neat) ν (cm^{-1}): 2942, 1503, 1352, 1275, 1130, 1095.

Melting point: 116-118 °C.

10. Syntheses of gold complexes

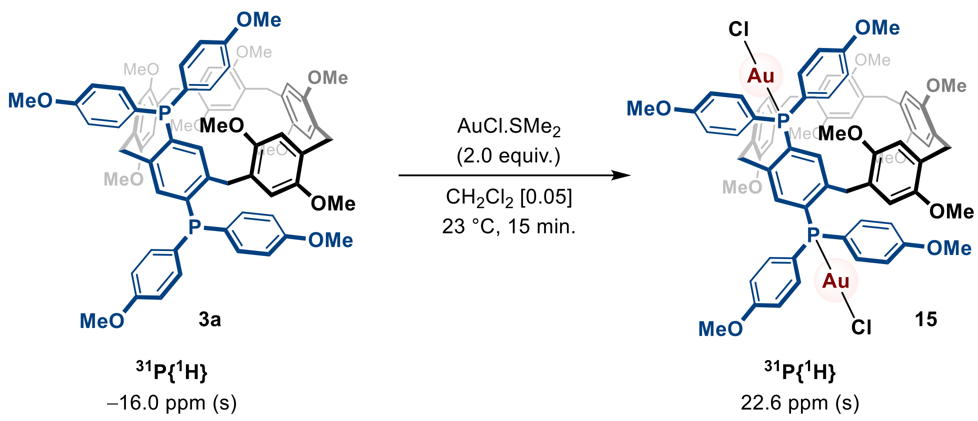

Figure S12. Synthesis of Au(I) complex **15**

In a glovebox, in an oven-dried 3 mL vial **3a** (15 mg, 13 µmol, 1.0 equiv.) was dissolved in CH_2Cl_2 (0.12 mL) and stirred at 23 °C. After 5 min., [(Me_2S)AuCl] (7.7 mg, 26 µmol, 2.0 equiv.) was added and the walls of the vial rinsed with 0.12 mL of CH_2Cl_2 (final concentration 0.050 M). After 15 min., the solution was analyzed by ^{31}P NMR, which indicated complete consumption of the starting material. The product was precipitated with *n*-hexane. The suspension was filtered and the solid obtained washed with *n*-hexane to afford **15** as a white powder (15 mg, 71% yield).

A1/A2-(bis(4-methoxyphenyl)phosphine)-pillar[5]arene bis gold chloride complex (17)

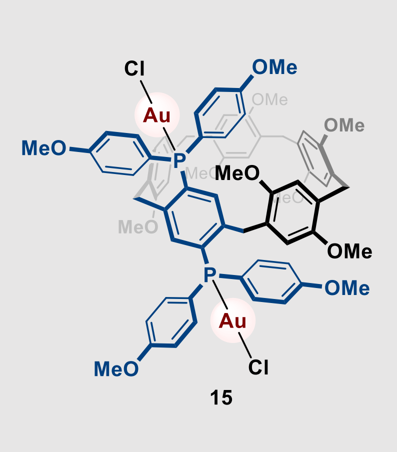

The compound is air and moisture sensitive.

^{1}H NMR (400 MHz, CD_2Cl_2) δ (ppm) = 7.35 – 7.27 (m, 8H), 7.05 (dd, $J_{P\text{-}H}$ = 13.1, 5.1 Hz, 2H), 6.97 (dd, $J_{H\text{-}H}$ = 8.9 Hz, $J_{P\text{-}H}$ = 1.8 Hz, 4H), 6.89 (s, 2H), 6.82 (s, 2H), 6.78 (dd, $J_{H\text{-}H}$ = 8.8 Hz, $J_{P\text{-}H}$ = 1.5 Hz, 4H), 6.41 (s, 2H), 6.11 (s, 2H), 4.62 (d, $J_{H\text{-}H}$ = 14.4 Hz, 2H), 3.89 (s, 6H), 3.85 (s, 6H), 3.83 (m, 4H), 3.74 (s, 6H), 3.66 (s, 6H), 3.62 – 3.50 (m, 4H), 3.30 (s, 6H), 3.26 (s, 6H).

^{13}C{^{1}H} NMR (101 MHz, CD_2Cl_2) δ (ppm) = 163.1 (C), 162.9 (C), 151.4 (C), 151.3 (C), 151.0 (C), 150.8 (C), 144.4 (C), 144.3 (C), 144.2 (C), 138.2 (CH), 138.1 (CH), 138.1 (CH), 136.8 (CH), 136.6 (CH), 136.1 (CH), 136.0 (CH), 131.0 (C), 130.4 (C), 130.0 (C), 129.2 (C), 128.4 (C), 125.7 (C), 121.8 (C), 121.1 (C), 119.6 (C), 118.9 (C), 115.3 (CH), 115.1 (CH), 114.7 (CH) 114.3 (CH), 113.9 (CH), 113.8 (CH), 56.5 (CH_3), 56.4 (CH_3), 56.2 (CH_3), 55.9 (CH_3), 55.9 (CH_3), 55.7 (CH_3), 34.9 (CH_2), 34.8 (CH_2), 30.2 (CH_2), 30.0 (CH_2).

^{31}P{^{1}H} NMR (162 MHz, CD_2Cl_2) δ (ppm) = 22.62.

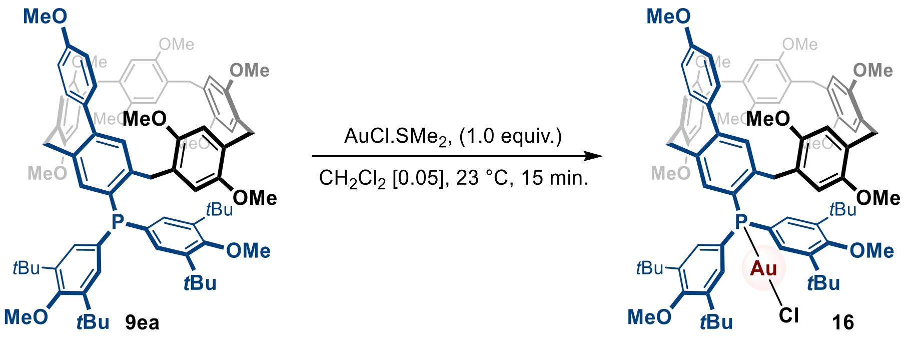

^{31}P{^{1}H}
−17.1 ppm (s)

^{31}P{^{1}H}
25.0 ppm (s)

Figure S13. Synthesis of Au(I) complex **16**

In a glovebox, in an oven-dried 1 mL vial **9ea** (10 mg, 7.5 µmol, 1.0 equiv.) was dissolved in CH_2Cl_2 (0.07 mL) and stirred at 23 °C. After 5 min, [(Me_2S)AuCl] (2.3 mg, 7.5 µmol, 1.0 equiv.) was added and the walls of the vial rinsed with CH_2Cl_2 (0.07 mL, final concentration 0.050 M). After 15 min., the solution was analyzed by ^{31}P NMR, which indicated complete consumption of the starting material. The product was precipitated with *n*-hexane. The suspension was filtered and the solid obtained washed with *n*-hexane to afford **16** as a white powder (10 mg, 82% yield).

A1-(bis((3,5-dimethoxy-4-*tert*-butyl)phenyl)phosphine)-A2-(4-(methoxy)phenyl)-pillar[5]arene gold chloride complex (16)

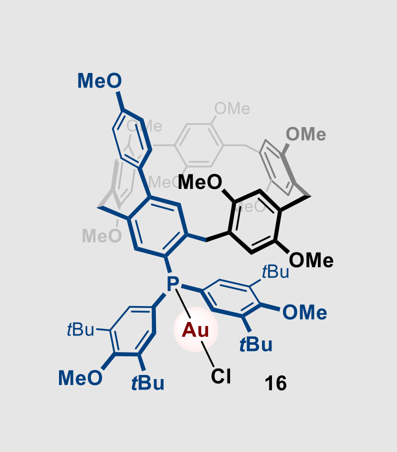

The compound is air and moisture sensitive.

^{1}H NMR (400 MHz, CD_2Cl_2) δ (ppm) = 7.55 (d, $J_{P\text{-}H}$ = 13.9 Hz, 2H), 7.30 (d, $J_{P\text{-}H}$ = 13.8 Hz, 2H), 7.10 (d, $J_{P\text{-}H}$ = 5.4 Hz, 1H), 7.03 (s, 1H), 6.97 (d, $J_{P\text{-}H}$ = 13.2 Hz, 1H), 6.94 (s, 1H), 6.88 (s, 1H), 6.84 (s, 1H), 6.83 (s, 1H), 6.79 (d, $J_{H\text{-}H}$ = 8.1 Hz, 2H), 6.75 (s, 1H), 6.71 (d, $J_{H\text{-}H}$ = 8.2 Hz, 2H), 6.56 (s, 1H), 5.40 (s, 1H), 4.52 (dd, $J_{P\text{-}H}$ = 14.2 Hz, $J_{H\text{-}H}$ = 2.0 Hz, 1H), 3.89 – 3.83 (m, 2H), 3.82 (s, 3H), 3.79 (d, $J_{H\text{-}H}$ = 5.1 Hz, 2H), 3.77 (s, 6H), 3.76 (s, 3H), 3.76 – 3.74 (m, 5H), 3.69 (s, 3H), 3.66 (s, 3H), 3.65 (s, 3H), 3.62 (s, 5H), 3.49 (d, $J_{\text{H-H}}$ = 13.3 Hz, 1H), 3.31 (s, 3H), 3.19 (s, 3H), 1.39 (s, 9H), 1.35 (s, 9H).

^{13}C{^{1}H} NMR (101 MHz, CD_2Cl_2) δ (ppm) = 163.23 (C), 163.21 (C), 163.1 (C), 163.1 (C), 159.2 (C), 151.6 (C), 151.5 (C), 151.5 (C), 151.1 (C), 150.9 (C), 150.9 (C), 150.9 (C), 150.8 (C), 150.5 (C), 150.2 (C), 145.3 (C), 145.2 (C), 145.1 (C), 145.0 (C), 144.5 (C), 144.4 (C), 136.9 (CH), 136.8 (CH), 136.6 (C), 136.5 (C), 134.7 (CH), 134.6 (CH), 134.3 (C), 134.3 (C), 133.9 (CH), 133.8 (CH), 132.6 (CH), 132.5 (CH), 130.6 (CH), 129.2 (C), 129.1 (C), 129.0 (C), 128.8 (C), 128.7 (C), 128.5 (C), 127.6 (C), 126.5 (C), 124.8 (C), 124.2 (C), 124.1 (C), 124.0 (C), 123.5 (C), 123.4 (C), 115.0 (CH), 114.6 (CH), 114.3 (CH), 114.2 (CH),114.1(CH), 114.0 (CH), 114.0 (CH), 113.9 (CH), 113.4 (CH), 113.0 (CH), 65.0 (CH_3), 56.6 (CH_3), 56.3 (CH_3), 56.2 (CH_3), 56.2 (CH_3), 56.2 (CH_3), 55.9 (CH_3), 55.8 (CH_3), 55.6 (CH_3), 55.4 (CH_3), 36.4 (C), 36.3 (C), 34.8 (CH_2), 33.3 (CH_2), 33.2 (CH_2), 32.0 (CH_2), 32.0 (CH_3), 31.2 (CH_2), 29.2 (CH_2), 28.9 (CH_2).

^{31}P{^{1}H} NMR (162 MHz, CD_2Cl_2) δ (ppm) = 25.03.

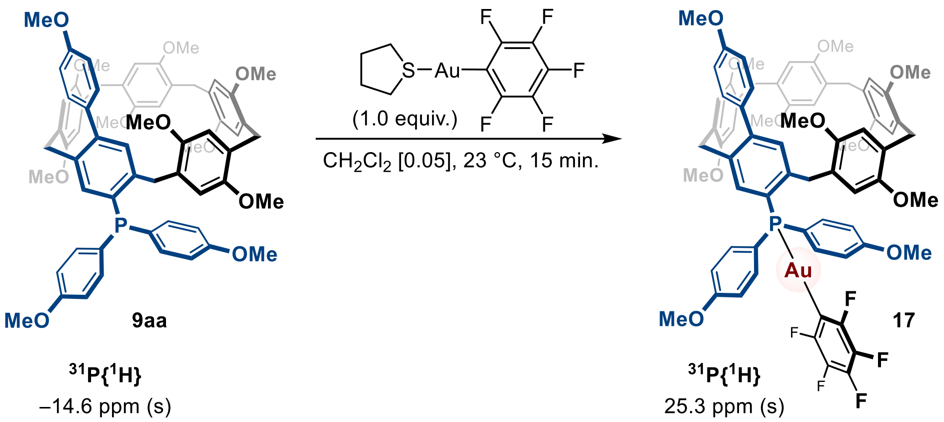

Figure S14. Synthesis of Au(I) complex **17**

In a glovebox, in an oven-dried 3 mL vial **9aa** (20 mg, 19 µmol, 1.0 equiv.) was dissolved in CH_2Cl_2 (0.16 mL) and stirred at 23 °C. After 5 min., [tetrahydrothiophen)Au(C_6F_5)] (9.1 mg, 19 µmol, 1.0 equiv.) was added and the walls of the vial rinsed with CH_2Cl_2 (0.16 mL, final concentration 0.050 M). After 15 min., the solution was analyzed by ^{31}P NMR, which indicated complete consumption of the starting material. The product was precipitated with MeOH. The suspension was filtered and the solid obtained was washed with MeOH to afford **17** as a white powder (19 mg, 70% yield).

A1-(bis(4-methoxyphenyl)phosphine)-A2-(4-(methoxy)phenyl)-pillar[5]arene gold pentafluorophenyl complex (17)

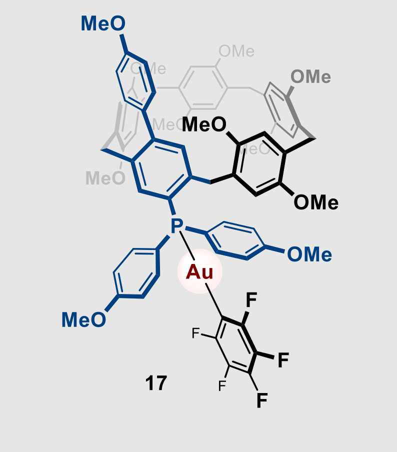

Single crystals were obtained by layering a concentrated solution in CH_2Cl_2 with *n*-pentane.

^{1}H NMR (400 MHz, $CDCl_3$) δ(ppm) = 7.69 (dd, $J_{P\text{-}H}$ = 12.0 Hz, $J_{H\text{-}H}$ = 8.8 Hz, 2H), 7.65 (dd, $J_{P\text{-}H}$ = 12.0 Hz, $J_{H\text{-}H}$ = 8.8 Hz, 2H), 7.05 (dd, $J_{H\text{-}H}$ = 8.8 Hz, $J_{P\text{-}H}$ = 1.7 Hz, 2H), 7.04 (dd, $J_{H\text{-}H}$ = 8.7 Hz, $J_{P\text{-}H}$ = 1.7 Hz, 2H), 6.88 (s, 1H), 6.85 (d, $J_{P\text{-}H}$ = 12.6 Hz, 1H), 6.73 (s, 1H), 6.70 (s,1H), 6.60 (s, 1H), 6.53 (s, 1H), 6.50 (s, 1H), 6.43 (s, 1H), 6.35 (d, $J_{P\text{-}H}$ = 5.1 Hz, 1H), 6.25 (d, $J_{H\text{-}H}$ = 7.9 Hz, 2H), 6.17 (d, $J_{H\text{-}H}$ = 7.7 Hz, 2H), 5.34 (s, 1H), 4.56 (dd, $J_{H\text{-}H}$ = 15.5 Hz, $J_{H\text{-}H}$ = 1.9 Hz, 1H), 3.99 (d, $J_{H\text{-}H}$ = 14.2 Hz, 1H), 3.88 (s, 3H), 3.88 (s, 3H), 3.84 (m, 1H), 3.82 (s, 3H), 3.81 – 3.78 (m, 2H), 3.75 (s, 3H), 3.74 – 3.65 (m, 5H), 3.63 (s, 3H), 3.49 (s, 3H), 3.45 (s, 3H), 3.38 (s, 3H), 3.31 (s, 3H), 3.28 (s, 6H).

^{13}C{^{1}H} NMR (101 MHz, $CDCl_3$) δ(ppm) = 162.4 (C), 162.3 (C), 162.3 (C), 158.2 (C), 151.6 (C), 151.3 (C), 151.2 (C), 151.2 (C), 151.1 (C), 150.7 (C), 150.6 (C), 150.3 (C), 145.1 (C), 145.0 (C), 143.5 (C), 143.3 (C), 137.3 (C), 137.2 (C), 137.0 (CH), 136.8 (CH), 136.3 (CH), 136.2 (CH), 136.1 (CH), 135.9 (CH), 134.0 (C), 132.3 (CH), 132.2 (CH), 129.5 (C), 129.3 (CH), 128.9 (C), 128.8 (C), 128.4 (C), 128.2 (C), 128.1 (C), 127.0 (C), 126.8 (C), 125.5 (C), 125.0 (C), 122.0 (C), 121.43 (C), 121.38 (C), 120.8 (C), 115.2 (CH), 115.1 (CH), 115.01 (CH), 115.0 (CH), 114.9 (CH), 114.6 (CH), 114.5 (CH), 114.3 (CH), 114.3 (CH), 113.3 (CH), 113.1 (CH), 113.0 (CH), 56.5 (CH_3), 56.3 (CH_3), 56.1 (CH_3), 55.9 (CH_3), 55.8 (CH_3), 55.6 (CH_3), 55.3 (CH_3), 55.3 (CH_3), 54.8 (CH_3), 36.3 (CH_2), 35.0 (CH_2), 34.9 (CH_2), 30.9 (CH_2), 30.8 (CH_2), 28.8 (CH_2).

^{31}P{^{1}H} NMR (162 MHz, $CDCl_3$) δ(ppm) = 33.83 (quintet, $J_{P\text{-}F}$ = 9.0 Hz).

^{19}F{^{1}H} NMR ($CDCl_3$, 282 MHz) δ(ppm) = –115.35 – –115.97 (m, 2F), –158.61 (t, J = 19.9 Hz, 1F), –161.81 – –162.87 (m, 2F).

HRMS (ESI$^+$) *m/z*: calculated for $C_{70}H_{69}F_5NO_{11}PAu$ $[M+NH_4]^+$: 1422.4189; found: 1422.4194.

IR (neat) ν (cm^{-1}): 2936, 2831, 1966, 1499, 1252, 1211, 1178, 1043. **Melting point:** 245 °C (decomp.).

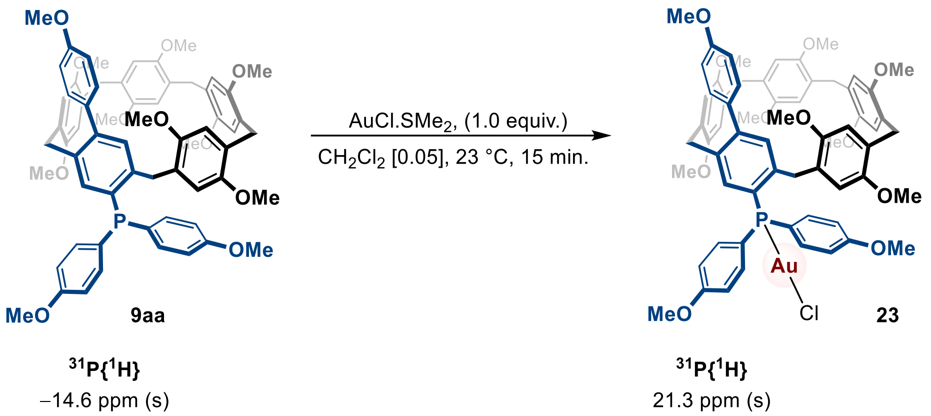

Figure S15. Synthesis of Au(I) complex **23**

In a glovebox, in an oven-dried 10 mL vial **9aa** (50 mg, 48.0 μmol, 1.0 equiv.) was dissolved in CH_2Cl_2 (0.5 mL) and stirred at 23 °C. After 5 min., [(Me_2S)AuCl] (14.9 mg, 48 μmol, 1.0 equiv.) was added and the walls of the vial rinsed with CH_2Cl_2 (0.5 mL, final concentration 0.050 M). After 30 min., the solution was analyzed by ^{31}P NMR, which indicated complete consumption of the starting material. The solution was concentrated inside the glovebox to *ca.* 0.5 mL, and the product was precipitated with *n*-hexane. The suspension was filtered and the solid obtained washed with *n*-hexane to afford **22** as a white powder (53.8 mg, 88% yield).

A1-(bis(4-methoxyphenyl)phosphine)-A2-(4-(methoxy)phenyl)-pillar[5]arene gold chloride complex (23)

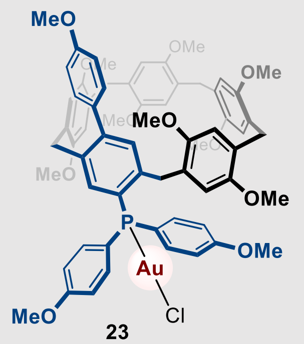

23

The compound is air and moisture sensitive.

^{1}H NMR (500 MHz, CD_2Cl_2) δ (ppm) = 7.61 (dd, $J_{P\text{-}H}$ = 12.8 Hz, $J_{H\text{-}H}$ = 8.8 Hz, 2H), 7.46 (dd, $J_{P\text{-}H}$ = 12.3 Hz, $J_{H\text{-}H}$ = 8.8 Hz, 2H), 7.17 (d, $J_{P\text{-}H}$ = 5.5 Hz, 1H), 7.03 (ddd, $J_{P\text{-}H}$ = 8.8 Hz, $J_{P\text{-}H}$ = 4.9 Hz, $J_{H\text{-}H}$ = 1.9 Hz, 4H), 6.94 – 6.92 (m, 2H), 6.90 (d, $J_{H\text{-}H}$ = 1.9 Hz, 2H), 6.87 – 6.83 (m, 3H), 6.79 (d, $J_{P\text{-}H}$ = 8.8 Hz, 2H), 6.59 (s, 1H), 6.27 (s, 1H), 5.40 (s, 1H), 4.55 (dd, $J_{P\text{-}H}$ = 14.2 Hz, $J_{H\text{-}H}$ = 1.7 Hz, 1H), 4.11 (d, $J_{H\text{-}H}$ = 13.9 Hz, 1H), 3.89 (s, 3H), 3.87 (s, 4H), 3.84 (s, 3H), 3.80 (s, 8H), 3.78 – 3.75 (m, 3H), 3.74 (s, 1H), 3.73 (s, 1H), 3.70 (s, 1H), 3.69 – 3.67 (m, 2H), 3.66 (s, 6H), 3.49 (s, 1H), 3.46 (s, 3H), 3.45 (s, 3H), 3.34 (s, 3H).

^{13}C{^{1}H} NMR (125 MHz, CD_2Cl_2) δ (ppm) = 163.1 (C), 163.1 (C), 162.8 (C), 162.8 (C), 159.3 (C), 151.5 (C), 151.4 (C), 151.0 (C), 150.9 (C), 150.7 (C), 150.7 (C), 149.9 (C), 145.0 (C), 145.0 (C), 144.2 (C), 144.1 (C), 137.3 (CH), 137.2 (CH), 137.2 (CH), 137.1 (CH), 136.4 (C), 136.3 (C), 136.1 (CH), 136.0 (CH), 134.3 (C), 134.3 (CH), 134.2 (CH), 130.7 (CH), 129.4 (C), 129.0 (C), 129.0 (C), 128.9 (C), 128.9 (C), 128.4 (C), 127.3 (C), 125.9 (C), 123.9 (C), 123.4 (C), 121.9 (C), 121.3 (C), 120.7 (C), 120.1 (C), 115.3 (CH), 115.2 (CH), 115.1 (CH), 114.8 (CH), 114.4 (CH), 114.2 (CH), 114.0 (CH), 113.9 (CH), 113.5 (CH), 113.4 (CH), 112.9 (CH), 56.2 (CH_3), 56.1 (CH_3), 56.1 (CH_3), 56.0 (CH_3), 55.9 (CH_3), 55.6 (CH_3), 55.4 (CH_3), 55.3 (CH_3), 35.0 (CH_2), 33.9 (CH_2), 33.8 (CH_2), 31.3 (CH_2), 28.9 (CH_2), 28.7 (CH_2).

^{31}P{^{1}H} NMR (162 MHz, CD_2Cl_2) δ (ppm) = 21.3.

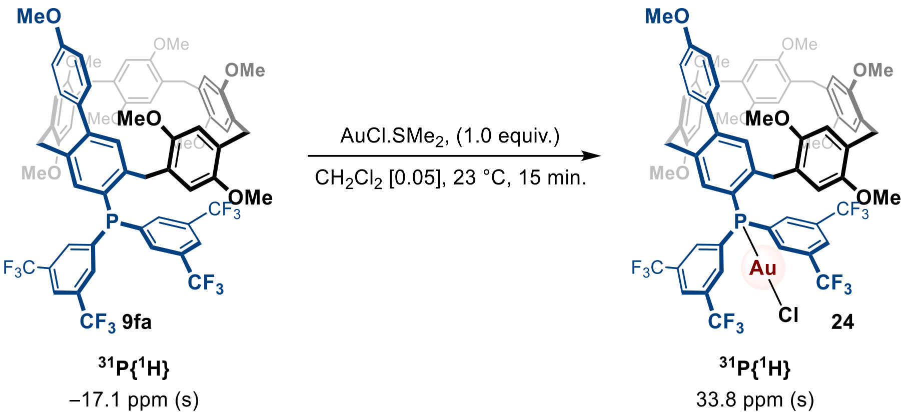

Figure S16. Synthesis of Au(I) complex **24**

In a glovebox, in an oven-dried 10 mL vial **9fa** (60 mg, 47.8 μmol, 1.0 equiv.) was dissolved in CH_2Cl_2 (0.5 mL) and stirred at 23 °C. After 5 min., [Me_2S)AuCl] (14.8 mg, 47.8 μmol, 1.0 equiv.) was added and the walls of the vial rinsed with CH_2Cl_2 (0.5 mL, final concentration 0.050 M). After 30 min., the solution was analyzed by ^{31}P NMR, which indicated complete consumption of the starting material. The solution was concentrated inside the glovebox to *ca.* 0.5 mL, and the product was precipitated with MeOH. The suspension was filtered and the solid obtained washed with MeOH to afford **24** as a white powder (58 mg, 82% yield).

A1-(bis(3,5-bis(trifluoromethyl)phenyl)phosphine)-A2-(4-(methoxy)phenyl)-pillar[5]arene gold chloride complex (24)

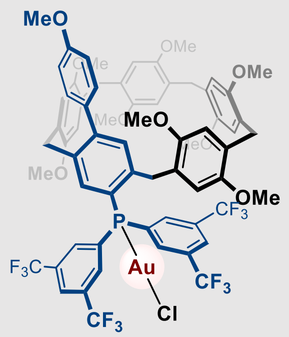

24

The compound is air and moisture sensitive.

^{1}H NMR (500 MHz, CD_2Cl_2) δ (ppm) = 8.22 (s, 1H), 8.16 – 8.10 (m, 3H), 7.98 (d, $J_{P\text{-}H}$ = 12.8 Hz, 2H), 7.36 (d, $J_{P\text{-}H}$ = 6.2 Hz, 1H), 6.96 (d, $J_{P\text{-}H}$ = 8.0 Hz, 2H), 6.87 (s, 3H), 6.84 (d, $J_{P\text{-}H}$ = 13.7 Hz, 2H), 6.83 – 6.78 (m, 4H), 6.64 (d, $J_{H\text{-}H}$ = 8.0 Hz, 2H), 5.41 (s, 1H), 4.49 (dd, $J_{H\text{-}H}$ = 14.2, $J_{P\text{-}H}$ 2.1 Hz, 1H), 4.03 (d, $J_{H\text{-}H}$ = 14.2 Hz, 1H), 3.94 – 3.84 (m, 2H), 3.84 (s, 4H), 3.83 – 3.79 (m, 3H), 3.79 (s, 6H), 3.78 – 3.72 (m, 2H), 3.71 (s, 3H), 3.67 (s, 4H), 3.66 – 3.63 (m, 3H), 3.57 (s, 3H), 3.56 (s, 3H), 3.50 (d, $J_{H\text{-}H}$ = 13.3 Hz, 1H), 3.33 (s, 3H), 3.29 (s, 3H).

^{13}C{^{1}H} NMR (125 MHz, CD_2Cl_2) δ (ppm) = 159.6 (C), 151.4 (C), 151.3 (C), 151.0 (C), 150.8 (C), 150.7 (C), 150.7 (C), 150.4 (C), 146.9 (C), 146.9 (C), 145.0 (C), 144.9 (C), 138.4 (C), 138.3 (C), 137.1 (CH), 137.0 (CH), 135.4 (CH), 135.3 (CH), 135.2 (CH), 135.2 (CH), 134.4 (C), 133.9 (C), 133.8 (CH), 133.8 (CH), 133.5 (C), 133.4 (q, $J_{C\text{-}F}$ = 35.1 Hz) (C), 133.4 (q, $J_{C\text{-}F}$ = 35.1 Hz) (C), 132.9 (C), 132.4 (C), 131.2 (CH), 130.7 (CH), 130.1 (C), 129.5 (C), 129.0 (C), 129.0 (C), 128.6 (C), 128.1 (C), 127.1 (CH), 127.1 (CH), 126.9 (CH), 126.8 (CH), 125.9 (C), 125.9 (C), 124.1 (C), 124.1 (C), 122.0 (C), 121.9 (C), 120.6 (CH), 119.8 (C), 119.8 (CH), 119.7 (C), 118.6 (C), 118.1 (C), 114.8 (CH), 114.4 (CH), 114.2 (CH), 114.2 (CH), 114.1 (CH), 114.0 (CH), 113.9 (CH), 113.9 (CH), 113.2 (CH), 112.9 (CH), 56.4 (CH_3), 56.1 (CH_3), 56.1 (CH_3), 56.0 (CH_3), 55.8 (CH_3), 55.7 (CH_3), 55.7 (CH_3), 55.4 (CH_3), 34.7 (CH_2), 34.6 (CH_2), 34.3 (CH_2), 30.7 (CH_2), 30.1 (CH_2), 29.2 (CH_2), 28.9 (CH_2).

^{31}P{^{1}H} NMR (202 MHz, CD_2Cl_2) δ (ppm) = 27.9.

^{19}F{^{1}H} NMR (282 MHz, CD_2Cl_2) δ (ppm) = –63.09, –63.3.

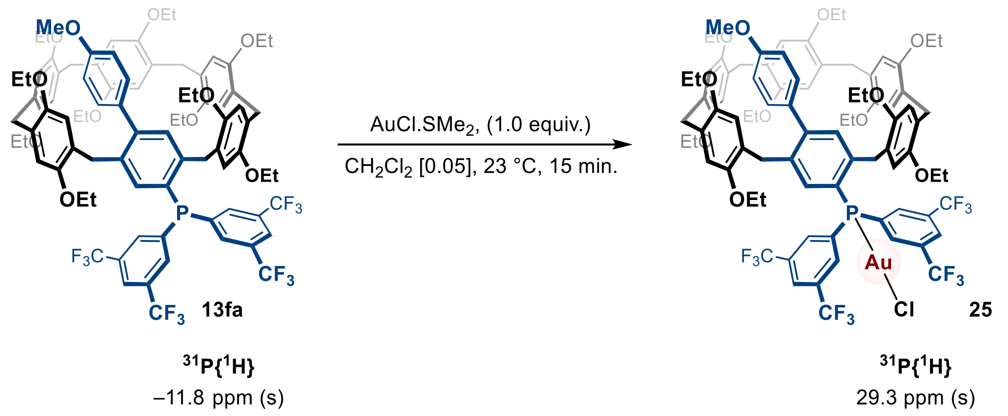

Figure S17. Synthesis of Au(I) complex **25**

In a glovebox, in an oven-dried 10 mL vial **13fa** (11.0 mg, 71.3 µmol, 1.0 equiv.) was dissolved in CH_2Cl_2 (0.2 mL) and stirred at 23 °C. After 5 min., [(Me_2S)AuCl] (2.1 mg, 71.3 µmol, 1.0 equiv.) was added and the walls of the vial rinsed with CH_2Cl_2 (0.3 mL, final concentration 0.012 M). After 30 min., the solution was analyzed by ^{31}P NMR, which indicated complete consumption of the starting material. The solution was concentrated inside the glovebox to *ca.* 0.2 mL, and the product was precipitated with MeOH. The suspension was filtered and the solid obtained washed with MeOH to afford **25** as a white powder (11 mg, 89% yield).

A1-(bis(3,5-bis(trifluoromethyl)phenyl)phosphine)-A2-triflate-ethoxy-pillar[6]arene gold chloride complex (25)

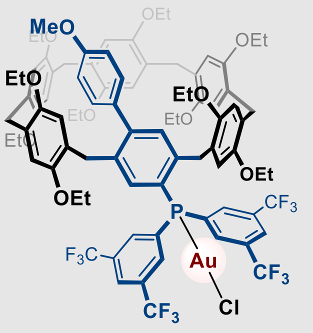

25

The compound is air and moisture sensitive.

^{1}H NMR (400 MHz, CD_2Cl_2) δ (ppm) = 8.20 – 8.08 (m, 4H), 7.99 (d, $J_{P\text{-}H}$ = 12.4 Hz, 2H), 6.91 (d, $J_{P\text{-}H}$ = 13.7 Hz, 1H), 6.87 (d, $J_{P\text{-}H}$ = 5.9 Hz, 1H), 6.81 (s, 1H), 6.77 (s, 1H), 6.73 – 6.71 (m, 3H), 6.70 (s, 1H), 6.68 (s, 1H), 6.66 (s, 1H), 6.63 (s, 1H), 6.53 (d, $J_{P\text{-}H}$ = 8.8 Hz, 2H), 6.47 (s, 2H), 5.51 (s, 1H), 4.46 (d, $J_{H\text{-}H}$ = 15.7 Hz, 1H), 3.92 – 3.87 (m, 3H), 3.87 – 3.83 (m, 7H), 3.83 – 3.80 (m, 5H), 3.80 – 3.77 (m, 3H), 3.75 – 3.69 (m, 2H), 3.65 (s, 3H), 3.65 – 3.61 (m, 3H), 3.60 – 3.54 (m, 2H), 3.51 – 3.45 (m, 2H), 3.44 – 3.38 (m, 2H), 1.37 (d, $J_{H\text{-}H}$ = 7.0 Hz, 3H), 1.33 (d, $J_{H\text{-}H}$ = 2.3 Hz, 2H), 1.33 – 1.31 (m, 4H), 1.31 – 1.30 (m, 4H), 1.29 – 1.28 (m, 3H), 1.28 – 1.26 (m, 4H), 1.26 (s, 2H), 1.24 (s, 1H), 1.11 (t, $J_{H\text{-}H}$ = 6.9 Hz, 3H), 0.92 (t, $J_{H\text{-}H}$ = 7.0 Hz, 3H), 0.81 (t, $J_{H\text{-}H}$ = 7.0 Hz, 3H).

^{13}C{^{1}H} NMR (101 MHz, CD_2Cl_2) δ (ppm) = 159.3 (C), 151.1 (C), 151.0 (C), 150.9 (C), 150.7 (C), 150.7 (C), 150.5 (C), 150.1 (C), 147.6 (C), 147.6 (C), 144.0 (C), 143.9 (C), 139.1 (C), 139.1 (C), 136.4 (CH), 136.3 (CH), 135.6 (CH), 135.4 (CH), 133.8 (CH), 133.7 (CH), 133.6 (CH), 133.2 (C), 133.0 (C), 131.9 (C), 131.4 (C), 130.3 (C), 130.1 (CH), 128.7 (C), 128.5 (C), 128.5 (C), 128.2 (C), 128.1 (C), 127.9 (C), 127.9 (C), 127.1 (CH), 126.8 (CH), 126.5 (C), 124.8 (C), 123.4 (q, J = 274.0 Hz) (C), 123.4 (q, J = 274.0 Hz) (C), 119.9 (C), 119.4 (C), 116.4 (CH), 116.0 (CH), 115.8 (CH), 115.6 (CH), 115.4 (CH), 115.3 (CH), 115.2 (CH), 115.1 (CH), 114.8 (CH), 113.7 (CH), 113.7 (CH), 64.8 (CH_2), 64.7 (CH_2), 64.6 (CH_2), 64.5 (CH_2), 64.4 (CH_2), 64.4 (CH_2), 64.3 (CH_2), 64.3 (CH_2), 64.2 (CH_2), 63.7 (CH_2), 55.4 (CH_3), 36.7 (CH_2), 36.6 (CH_2), 35.6 (CH_2), 31.6 (CH_2), 31.4 (CH_2), 31.3 (CH_2), 30.8 (CH_2), 30.1 (CH_2), 15.5 (CH_3), 15.5 (CH_3), 15.4 (CH_3), 15.4 (CH_3), 15.3 (CH_3), 15.2 (CH_3), 14.9 (CH_3).

^{31}P{^{1}H} NMR (162 MHz, CD_2Cl_2) δ (ppm) = 29.3.

^{19}F{^{1}H} NMR (282 MHz, CD_2Cl_2) δ (ppm) = –62.27.

11. Au(I)-catalyzed cycloisomerization of 1,6-enyne

11.1 General procedure

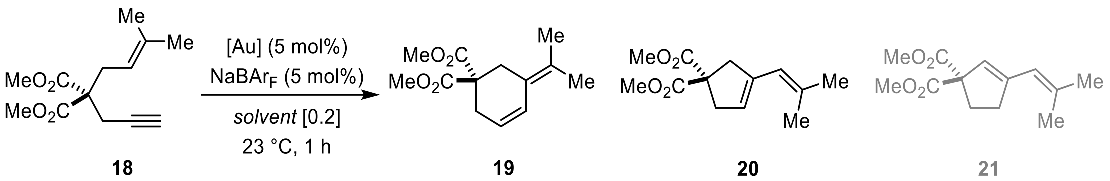

In a glovebox, in an oven-dried 3 mL screw cap vial equipped with a stirring bar, **18** (23.8, 0.1 mmol, 1.0 equiv.) was dissolved in the appropriate solvent (0.4 mL). In another 3 mL oven-dried screw cap vial equipped with a stirring bar either a combination of [(Me_2S)AuCl] (1.5 mg, 5 µmol, 5.0 mol%) and the appropriate ligand (5 mol%) – or alternatively the isolated gold complex (5 mol%) – were dissolved in the solvent of choice (0.4 mL). After 10 min., the appropriate scavenger (5 mol%) was added to the vial containing the gold precatalyst. This solution was transferred to the vial containing **18** (final concentration 0.125 M). After 1 h, mesitylene was added as the internal standard, the reaction mixture was transferred to an NMR tube and analyzed by ^{1}H NMR spectroscopy.[14]

Note: the reactions using $AgSbF_6$ must be performed in the absence of light.

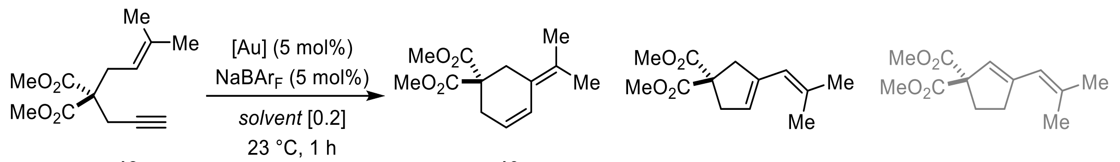

entry	ligand	scavenger	solvent	conv. (%)[a]	**19 : 20: 21**[a]
1	**22**	$NaBAr_F$	CD_2Cl_2	96	1 : 8 : 0
2	Ph_3P	$NaBAr_F$	CD_2Cl_2	94	1 : 8 : 0
3	Ph_3P	$NaBAr_F$	1,2-dichloroethane	96	1 : 6.3 : 0
4	Ph_3P	$NaBAr_F$	chlorocyclohexane	97	1 : 4.6 : 0
5	**22**	$NaBAr_F$	1,4-dibromobutane	>99	1 : 3.8 : 0
6	**3a**	$NaBAr_F$	CD_2Cl_2	97	1 : 10.8 : 0
7	**14a**	$NaBAr_F$	CD_2Cl_2	95	1 : 9.4 : 0
8	**9aa**	$AgSbF_6$	CD_2Cl_2	96	1 : 5.9 : 0
9	**23**	$NaBAr_F$	CD_2Cl_2	97	1 : 6.7 : 0
10	**9aa**	$NaBAr_F$	CD_2Cl_2	>99	1 : 7.0 : 0
11	**9aa**	$NaBAr_F$	1,2-dichloroethane	95	1 : 5.1 : 0
12	**9aa**	$NaBAr_F$	chlorocyclohexane	94	1 : 4.3 : 0
13	**23**	$NaBAr_F$	1,4-dibromobutane	>99	1 : 1.5 : 0.8
14	**9da**	$NaBAr_F$	CD_2Cl_2	97	1 : 16.1 : 0
15	**9ea**	$AgSbF_6$	CD_2Cl_2	86	1 : 4.5 : 0
16	**9ea**	$NaBAr_F$	CD_2Cl_2	97	1 : 3.1 : 1
17	**9ga**	$NaBAr_F$	CD_2Cl_2	95	1 : 9 : 1
18	**9fa**	$AgSbF_6$	CD_2Cl_2	74	1 : 14 : 0
19	**24**	$NaBAr_F$	CD_2Cl_2	98	1 : 22.2 : 1
20	**9fa**	$NaBAr_F$	CD_2Cl_2	96	1 : 16 : 0
21	**9fa**	$NaBAr_F$	1,2-dichloroethane	95	1 : 9.9 : 0
22	**9fa**	$NaBAr_F$	chlorocyclohexane	73	1 : 7.8 : 0
23	**24**	$NaBAr_F$	1,4-dibromobutane	>99	1 : 10.2 : 0.5
24	**9fb**	$AgSbF_6$	CD_2Cl_2	73	1 : 13.7 : 0
25	**13fa**	$AgSbF_6$	CD_2Cl_2	73	1 : 11.1 : 0
26	**13fa**	$NaBAr_F$	CD_2Cl_2	98	1 : 10.6 : 0
27	**13fa**	$NaBAr_F$	1,2-dichloroethane	96	1 : 7.2 : 0
28	**13fa**	$NaBAr_F$	chlorocyclohexane	95	1 : 6.3 : 0
29	**25**	$NaBAr_F$	1,4-dibromobutane	>99	1 : 2.4 : 3.2

Table S3. Reaction conditions: **18** (0.1 mmol), $[Me_2S(AuCl)]$ (5 mol%), scavenger (5 mol%) in 0.8 mL of solvent. [a] Determined by ^1H NMR spectroscopy using an internal standard.

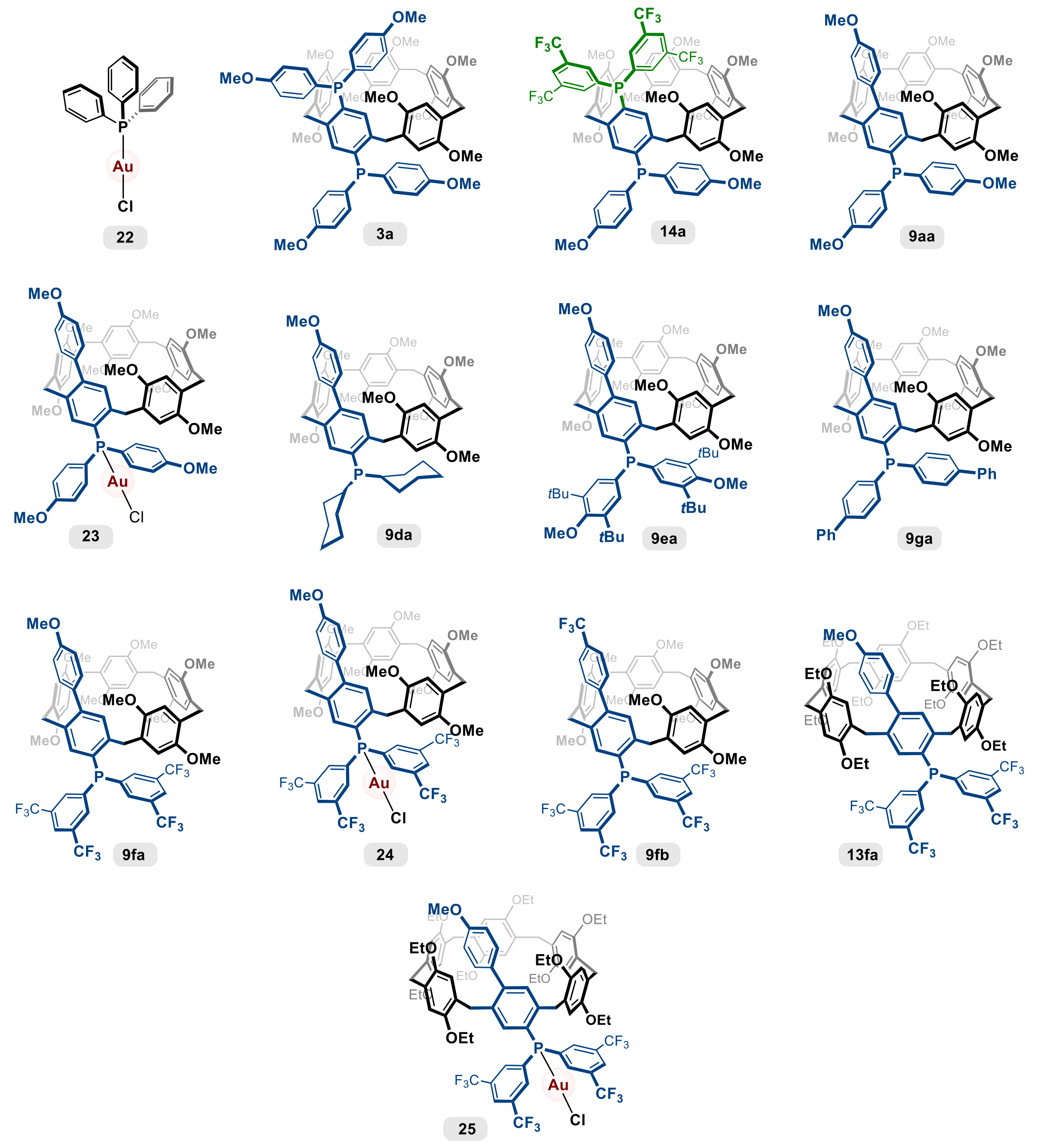

Figure S18. Ligands and isolated gold complexes surveyed

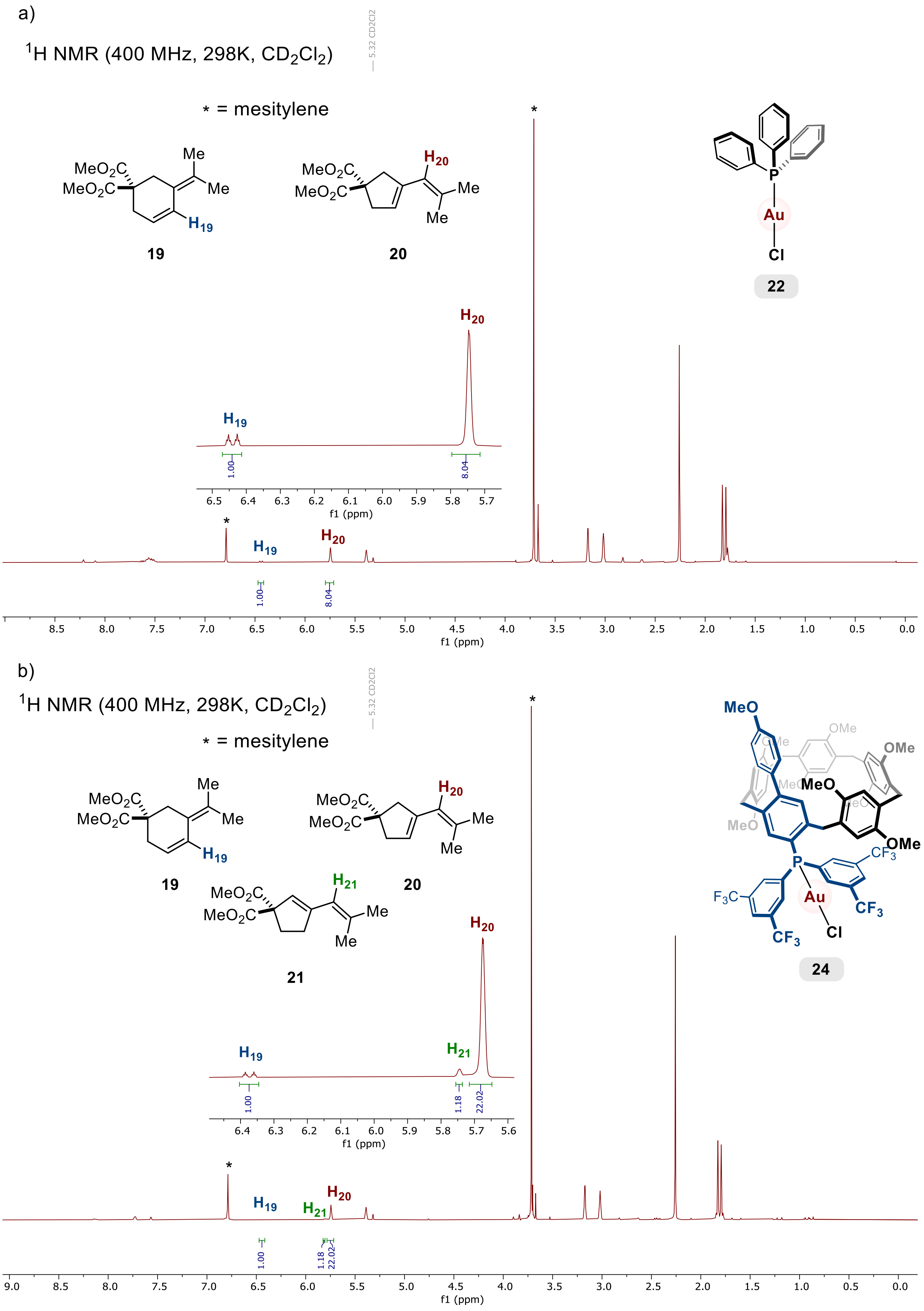

Figure S19. Representative ^{1}H NMR spectra of the reaction mixtures performed with a) **22** after 1 h and b) **25** after 1 h.

12. X-ray analyses

A suitable crystal was selected, and X-ray intensity data were collected on a Rigaku XtaLAB Synergy, Dualflex, HyPix-Arc 150° diffractometer using Cu Kα radiation (λ = 1.54184 Å). Using Olex2, the structure was solved with the SHELXT structure solution program using dual space methods and refined with the SHELXL refinement package using least squares minimization.[15–17] Summaries of crystal data and structure refinement parameters are given in the tables below.

Table S4. Crystal data and structure refinement for **3b**

CCDC Number	**2553581**	
Empirical formula	C72 H74 Cl2 O8 P2	
Formula weight	1200.15	
Temperature	120.00(10) K	
Wavelength	1.54184 Å	
Crystal system	Orthorhombic	
Space group	$Pna2_1$	
Unit cell dimensions	a = 20.05404(7) Å	α= 90°
	b = 12.53725(4) Å	β= 90°
	c = 25.19386(8) Å	γ = 90°
Volume	6334.30(4) $Å^3$	
Z	4	
Density (calculated)	1.258 Mg/m^3	
Absorption coefficient	1.844 mm^{-1}	
F(000)	2536	
Crystal size	0.505 x 0.283 x 0.174 mm^3	
Theta range for data collection	3.509 to 73.844°.	
Index ranges	-24<=h<=23, -15<=k<=15, -31<=l<=31	
Reflections collected	185224	
Independent reflections	12714 [R(int) = 0.0468]	
Completeness to theta = 67.684°	100.0 %	
Absorption correction	Gaussian	
Max. and min. transmission	1.000 and 0.157	
Refinement method	Full-matrix least-squares on F^2	
Data / restraints / parameters	12714 / 1 / 770	
Goodness-of-fit on F^2	1.068	
Final R indices [I>2sigma(I)]	R1 = 0.0425, wR2 = 0.1166	
R indices (all data)	R1 = 0.0430, wR2 = 0.1172	
Absolute structure parameter	0.014(14)	
Largest diff. peak and hole	0.320 and -0.359 e.$Å^{-3}$	

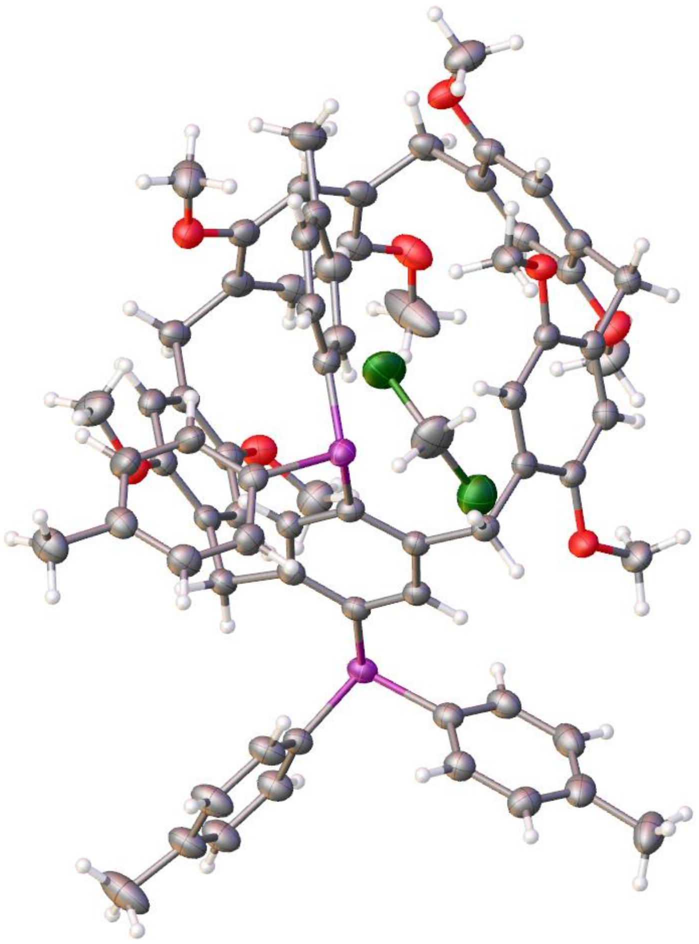

Figure S19. View of the asymmetric unit, with displacement ellipsoids at 50 percent probability level.

Table S5. Crystal data and structure refinement for **9fa**

CCDC Number	**2553580**	
Empirical formula	C67.20 H59.40 Cl2.40 F12 O9 P	
Formula weight	1355.10	
Temperature	120.00(10) K	
Wavelength	1.54184 Å	
Crystal system	Monoclinic	
Space group	I 1 2/c 1	
Unit cell dimensions	a = 27.41068(16) Å	α= 90°
	b = 12.09829(7) Å	β= 103.6530(7)°
	c = 39.6992(3) Å	γ = 90°
Volume	12793.13(14) Å^3	
Z	8	
Density (calculated)	1.407 Mg/m^3	
Absorption coefficient	2.105 mm^{-1}	
F(000)	5588	
Crystal size	0.32 x 0.058 x 0.044 mm^3	
Theta range for data collection	2.291 to 74.711°.	
Index ranges	-33≤h≤33, -11≤k≤14, -47≤l≤49	
Reflections collected	53679	
Independent reflections	12763 [R(int) = 0.0207]	
Completeness to theta = 67.684°	99.9 %	
Absorption correction	Gaussian	
Max. and min. transmission	1.000 and 0.654	
Refinement method	Full-matrix least-squares on F^2	
Data / restraints / parameters	12763 / 91 / 890	
Goodness-of-fit on F^2	1.040	
Final R indices [I>2sigma(I)]	R1 = 0.0408, wR2 = 0.1012	
R indices (all data)	R1 = 0.0472, wR2 = 0.1051	
Largest diff. peak and hole	0.936 and -0.936 e.Å^{-3}	

Comments

One CH_2Cl_2 molecule is disordered and was refined as 2 components. The following restraints and constraints were used:

DFIX 1.77 Cl1A C19A Cl1B C19B Cl3 C19B Cl2 C19A
DANG 2.92 Cl3 Cl1B
DANG 2.92 Cl1A Cl2
RIGU Cl1A Cl2 C19A
RIGU Cl1B Cl3 C19B
SIMU 0.04 0.08 1 Cl1A Cl2 C19A H19A H19B Cl1B Cl3 C19B H19C H19D
EADP Cl2 Cl3

One methoxy group is disordered, with a disordered CH_2Cl_2 molecule nearby, only present when the disordered part with the smallest occupancy is present. The following restraints were used:

SADI O31 C32 O31 C12
DANG 2.92 Cl5 Cl6
DFIX 1.771 0.01 Cl5 C19 Cl6 C19
SIMU Cl5 Cl6 C19

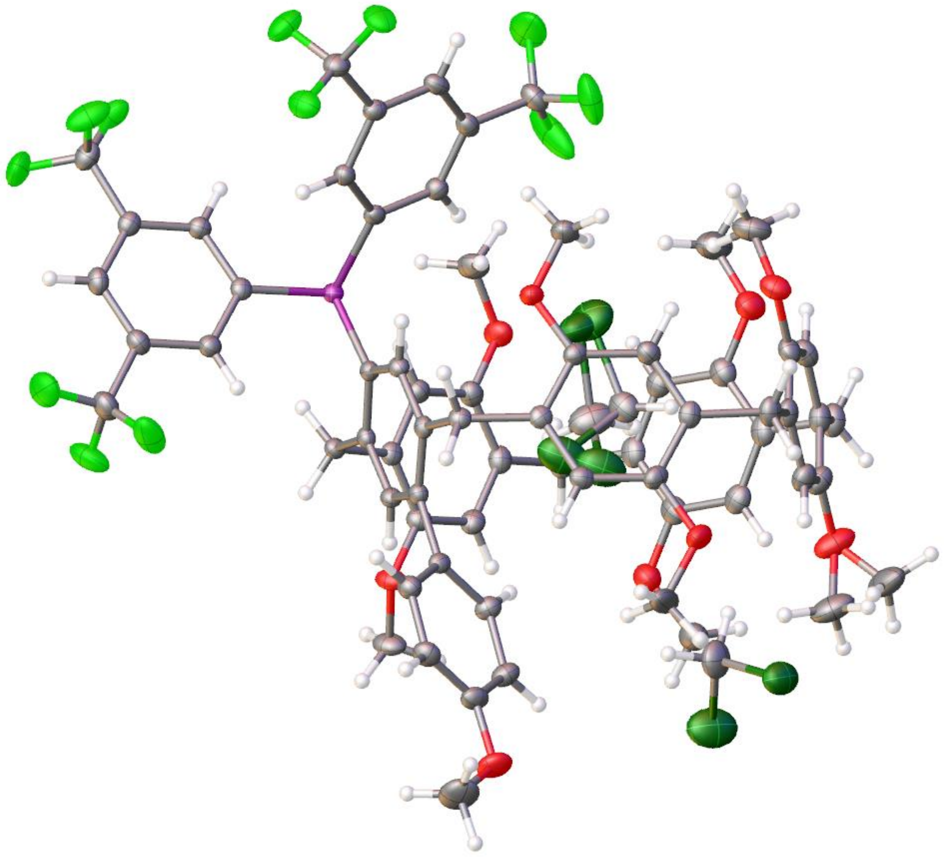

Figure S20. View of the asymmetric unit with displacement ellipsoids at 50 percent probability level.

Table S5. Crystal data and structure refinement for **14a**

CCDC Number	**2553582**	
Empirical formula	C73 H64 F12 O10 P2	
Formula weight	1391.18	
Temperature	120.00(10) K	
Wavelength	1.54184 Å	
Crystal system	Triclinic	
Space group	P-1	
Unit cell dimensions	a = 13.1736(2) Å	α= 105.254(2)°
	b = 17.3316(3) Å	β= 107.9810(10)°
	c = 18.0745(3) Å	γ = 96.6090(10)°
Volume	3699.24(11) Å^3	
Z	2	
Density (calculated)	1.249 Mg/m^3	
Absorption coefficient	1.264 mm^{-1}	
F(000)	1440	
Crystal size	0.361 x 0.244 x 0.114 mm^3	
Theta range for data collection	2.705 to 75.707°.	
Index ranges	-16 ≤ h ≤ 16, -21 ≤ k ≤ 21, -21 ≤ l ≤ 22	
Reflections collected	64872	
Independent reflections	14887 [R(int) = 0.0429]	
Completeness to theta = 67.684°	99.9 %	
Absorption correction	Gaussian	
Max. and min. transmission	1.000 and 0.387	
Refinement method	Full-matrix least-squares on F^2	
Data / restraints / parameters	14887 / 484 / 1011	
Goodness-of-fit on F^2	1.045	
Final R indices [I>2sigma(I)]	R1 = 0.0617, wR2 = 0.1709	
R indices (all data)	R1 = 0.0782, wR2 = 0.1821	
Extinction coefficient	n/a	
Largest diff. peak and hole	1.219 and -0.476 e.Å^{-3}	

Comments

Some trifluoromethyl groups are disordered:

The one bound to C62 was refined using one C and 2 groups of 3 F. The following restraints were used:

SADI F12A C66 F10B C66 F10A C66 F12B C66 F11A C66 F11B C66

SADI 0.04 F10B F11B F11B F12B F12B F10B F10A F11A F11A F12A F12A F10A

RIGU F10A F11A F12A C66

RIGU F10B F11B F12B C66

SIMU 0.04 0.08 1.7 F10A > F12B

The one bound to C54 was refined using one C and 2 groups of 3 F. The following restraints were used:

SADI F3B C58 F3A C58 F2A C58 F2B C58 F1B C58 F1A C58

SADI F2B C58 F1B C58 F1A C58 F3A C58 F3B C58 F2A C58

SADI 0.04 F1A F2A F2A F3A F3A F1A F1B F2B F2B F3B F3B F1B

SIMU F1A > F3B C58

RIGU F1A F2A F3A C58

RIGU F1B F2B F3B C58

The one bound to C56 was refined using 2 trifluoromethyl groups.

SADI C56 C59B C59A C56

SADI F4B C59B F6B C59B F6A C59A F5A C59A F5B C59B F4A C59A

RIGU F4A F5A F6A C59A

RIGU F4B F5B F6B C59B

SIMU F4A > F6B C59A C59B

Some methoxy groups are also disordered. They were refined using the following restraints:

DFIX 1.37 O7B C17 O74 C17

DFIX 1.42 O7B C48 O74 C75

DANG 2.37 C17 C48

DANG 2.37 C75 C17

RIGU C17 O74 C75

RIGU C17 O7B C48

SIMU C48 O7B O74

EADP C75 C48

RIGU O78 C27 C79

RIGU O78B C27 C79B

DFIX 1.37 O78 C27 O78B C27

DFIX 1.42 O78B C79B O78 C79

DANG 2.37 C27 C79B

DANG 2.37 C27 C79

Finally, the squeeze/bypass method as implemented in Olex2 was used to take care of disordered solvent. Four solvent-accessible voids were found (one located in the macrocycle, one outside) with about 40 electrons each. This electron count is consistent with the presence of either *n*-pentane or CH_2Cl_2 molecules, so that a mix of them could be envisioned. Unfortunately, modelling this disorder was not straightforward and the squeeze/bypass method was preferred.

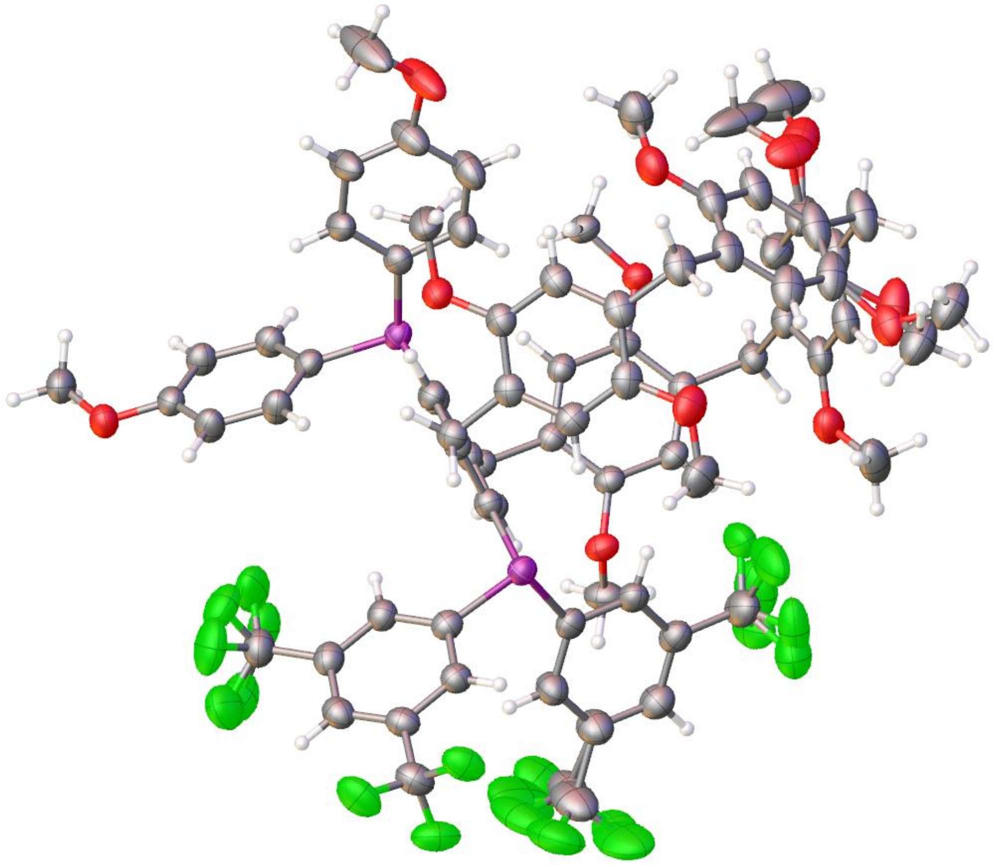

Figure S21. View of the asymmetric unit with displacement ellipsoids at 50 percent probability level.

Table S6. Crystal data and structure refinement for **15**

CCDC Number	**2553584**	
Empirical formula	C71 H72 Au2 Cl2 O12 P2	
Formula weight	1644.05	
Temperature	119.99(10) K	
Wavelength	1.54184 Å	
Crystal system	Monoclinic	
Space group	P 1 21/c 1	
Unit cell dimensions	a = 12.6168(2) Å	α= 90°
	b = 17.5419(3) Å	β= 99.8042(15)°
	c = 29.3008(4) Å	γ = 90°
Volume	6390.24(17) $Å^3$	
Z	4	
Density (calculated)	1.709 Mg/m^3	
Absorption coefficient	10.273 mm^{-1}	
F(000)	3264	
Crystal size	0.052 x 0.05 x 0.024 mm^3	
Theta range for data collection	2.948 to 74.675°.	
Index ranges	-15≤h≤15, -21≤k≤17, -35≤l≤36	
Reflections collected	79722	
Independent reflections	12946 [R(int) = 0.0493]	
Completeness to theta = 67.684°	99.9 %	
Absorption correction	Gaussian	
Max. and min. transmission	0.823 and 0.638	
Refinement method	Full-matrix least-squares on F^2	
Data / restraints / parameters	12946 / 424 / 915	
Goodness-of-fit on F^2	1.119	
Final R indices [I>2sigma(I)]	R1 = 0.0692, wR2 = 0.1481	
R indices (all data)	R1 = 0.0855, wR2 = 0.1543	
Largest diff. peak and hole	1.262 and -1.573 e.$Å^{-3}$	

Comments

There is a disorder on the substituent of the P atom bound to C20. Two parts were refined. Geometrical restraints were used:

SADI Au2B P2B Au2A P2A

SADI P2A C70 P2B C70 P2B C20 P2A C20

FLAT C80A C81A C82A C83A C86A C87A

DFIX 1.35 O84A C83A

DFIX 1.4 O84A C85A

DFIX 1.39 C87A C80A C81A C80A C82A C81A C82A C83A C83A C86A C86A C87A

SADI P2B C80B P2A C80A P2A C20 P2B C20

DANG 2.75 C87A P2A

DANG 2.75 P2A C81A

RIGU P2B C80B C81B C82B C83B O84B C85B C86B C87B Au2B Cl2B C70

RIGU Cl2A Au2A P2A C70 C80A C81A C82A C83A C86A C87A O84A C85A

SIMU Au2A Cl2A Cl2B P2A P2B O84A O84B C80B C81A C81B C82A C82B C83A C83B C85A C85B C86B C87B Au2B C80A C86A C87A

One methoxy group is probably disordered but was not split.

Restraints were applied on displacement parameters.

RIGU O13 C14

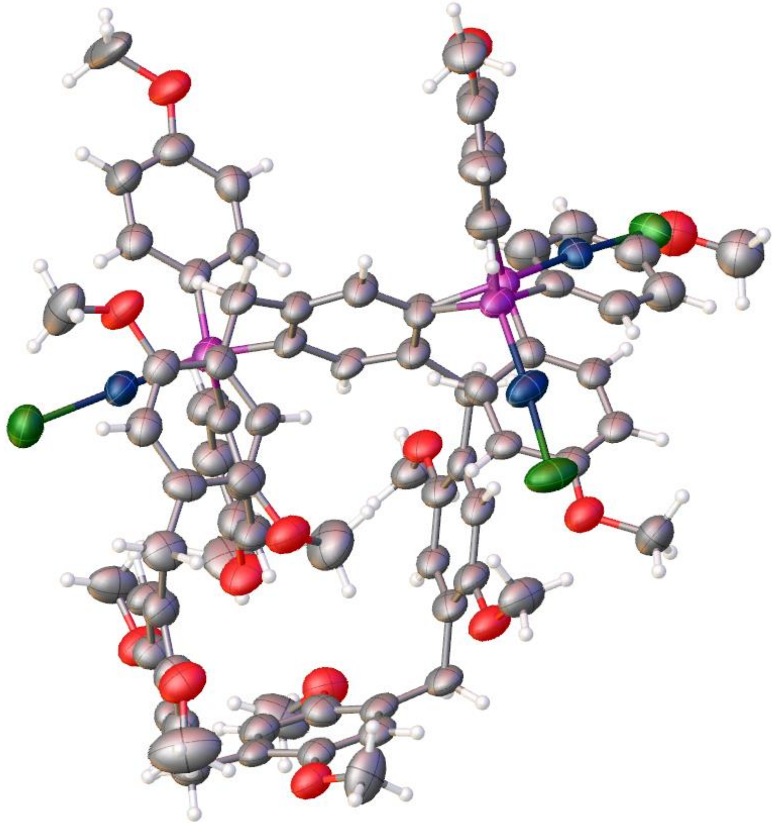

Figure S22. View of the asymmetric unit with displacement ellipsoids at 50 percent probability level.

Table S7. Crystal data and structure refinement for **16**

CCDC Number	**2553585**	
Empirical formula	C85 H109.67 Au0.59 Cl0.59 O11.74 P	
Formula weight	1486.24	
Temperature	120.00(10) K	
Wavelength	1.54184 Å	
Crystal system	Triclinic	
Space group	P-1	
Unit cell dimensions	a = 11.9491(2) Å	α= 71.4410(10)°
	b = 17.2607(2) Å	β= 89.4790(10)°
	c = 21.2443(3) Å	γ = 70.2180(10)°
Volume	3884.69(10) $Å^3$	
Z	2	
Density (calculated)	1.271 Mg/m^3	
Absorption coefficient	2.984 mm^{-1}	
F(000)	1570	
Crystal size	0.333 x 0.133 x 0.046 mm^3	
Theta range for data collection	2.207 to 75.979°.	
Index ranges	-14<=h<=15, -21<=k<=21, -26<=l<=19	
Reflections collected	99335	
Independent reflections	15682 [R(int) = 0.0475]	
Completeness to theta = 67.684°	99.8 %	
Absorption correction	Gaussian	
Max. and min. transmission	1.000 and 0.430	
Refinement method	Full-matrix least-squares on F^2	
Data / restraints / parameters	15682 / 77 / 1004	
Goodness-of-fit on F^2	1.251	
Final R indices [I>2sigma(I)]	R1 = 0.0629, wR2 = 0.1516	
R indices (all data)	R1 = 0.0657, wR2 = 0.1528	
Largest diff. peak and hole	1.511 and -1.032 e.$Å^{-3}$	

Comments

The complex is partly oxidized with 59 percent AuCl and 41 percent O bound to the phosphorus atom.

One *tert*-butyl group is disordered and was refined using 6 carbon atoms in two components.

The following constraints were used:
EADP C86A C86B

One *n*-pentane molecule is disordered and was refined using two components, with the following restraints/constraints:
SADI C99 C98 C94 C93 C98 C97 C93 C92 C92 C91 C97 C96 C91 C90 C96 C95
SADI 0.04 C95 C97 C96 C98 C97 C94
SADI 0.04 C90 C92 C91 C93 C92 C94
RIGU C98 C99 C97 C96 C95
RIGU C97 C99 C98 C96 C95
SIMU 0.04 0.08 1 C92 C94 C93 C91 C90 C97 C99 C98 C96 C95

Finally, a residual density was assigned to a water molecule whose occupancy was fixed at 1/3.
The attempts to refine simultaneously occupancy and displacement parameter for this water molecule failed.

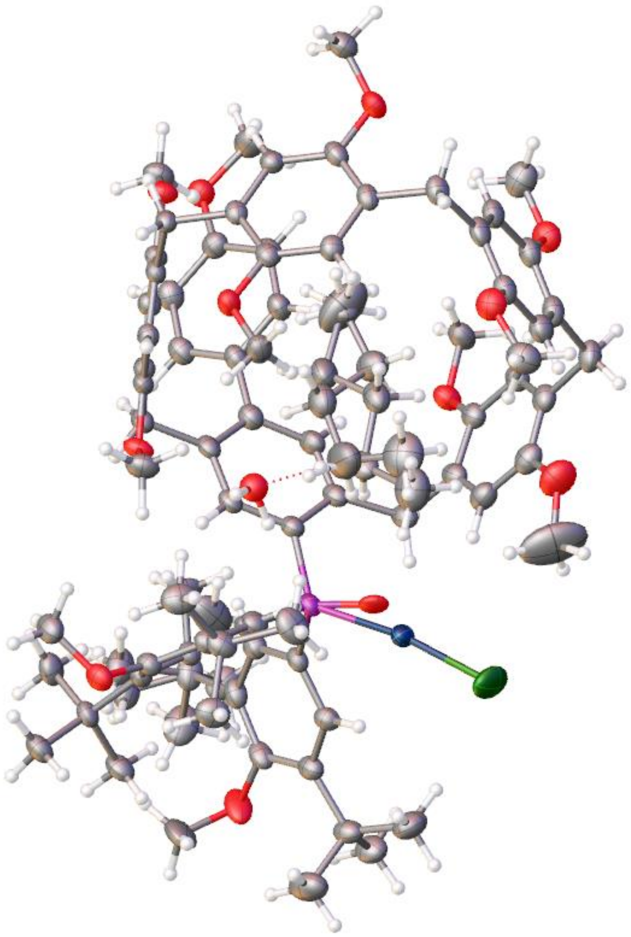

Figure S23. View of the asymmetric unit with displacement ellipsoids at 50 percent probability level.

Table S8. Crystal data and structure refinement for **17**

CCDC Number	**2553583**	
Empirical formula	C72 H69 Au Cl4 F5 O11 P	
Formula weight	1575.00	
Temperature	120.00(10) K	
Wavelength	1.54184 Å	
Crystal system	Monoclinic	
Space group	C 1 2/c 1	
Unit cell dimensions	a = 40.9463(2) Å	α= 90°
	b = 15.90780(10) Å	β= 105.0840(10)°
	c = 21.77730(10) Å	γ = 90°
Volume	13696.24(14) Å^3	
Z	8	
Density (calculated)	1.528 Mg/m^3	
Absorption coefficient	6.314 mm^{-1}	
F(000)	6368	
Crystal size	0.195 x 0.106 x 0.047 mm^3	
Theta range for data collection	2.235 to 76.099°.	
Index ranges	-49 ≤ h ≤ 51, -18 ≤ k ≤ 19, -27 ≤ l ≤ 27	
Reflections collected	173885	
Independent reflections	14079 [R(int) = 0.0338]	
Completeness to theta = 67.684°	100.0 %	
Absorption correction	Gaussian	
Max. and min. transmission	0.980 and 0.404	
Refinement method	Full-matrix least-squares on F^2	
Data / restraints / parameters	14079 / 284 / 966	
Goodness-of-fit on F^2	1.061	
Final R indices [I>2sigma(I)]	R1 = 0.0347, wR2 = 0.0862	
R indices (all data)	R1 = 0.0391, wR2 = 0.0887	
Extinction coefficient	n/a	
Largest diff. peak and hole	0.861 and -0.987 e.Å^{-3}	

Comments

One of the methoxybenzene moieties is disordered and was refined using 2 components.

SADI C82B C81 C82 C81 C82B C83B C84B C83B C84 C83 C82 C83 C81 C86B C81 C86 =
C85B C86B C85 C86 C84B C85B C84 C85
RIGU O87 C81 C82 C83 C84 C85 C86 C88
RIGU O87B C81 C82B C83B C84B C85B C86B C88B
SADI O87 C84 O87B C84B
SADI O87 C88 O87B C88B

One disordered CH_2Cl_2 was refined. The majority part with the following restraints:
DFIX 1.771 0.01 Cl3 C80 Cl4 C80
DFIX 2.916 0.03 Cl3 Cl4
SIMU Cl3 Cl4 C80
RIGU Cl3 Cl4 C80

The minority part as a rigid body.

Finally, C9 ellipsoid showed abnormal behavior. We hypothesize that this is caused by a small disorder around P1, with a gold atom located in the vicinity of C9. A disordered model was made. As this disorder only refined to about 5 percent, it was challenging to refine the lighter parts of the ligands. Rigid bodies were used with the following restraints:

DFIX 2.28 Au1B P1
EADP C9 Au1B
DFIX 1.39 C9 C10 C9 C14
DFIX 1.84 P1 C9B P1 C9
EADP C17B C18B C20B C22B C24B C26B
EADP F19B F21B F23B F25B F27B
DANG 2.75 P1 C14B
DANG 2.75 P1 C10B
DANG 4.61 P1 C12B
FLAT P1 C9B C10B > C14B
DANG 2.9 C1 C9B
DANG 2.9 C28 C9B
RIGU C17 C18 F19 C20 F21 C22 F23 C24 F25 C26 F27

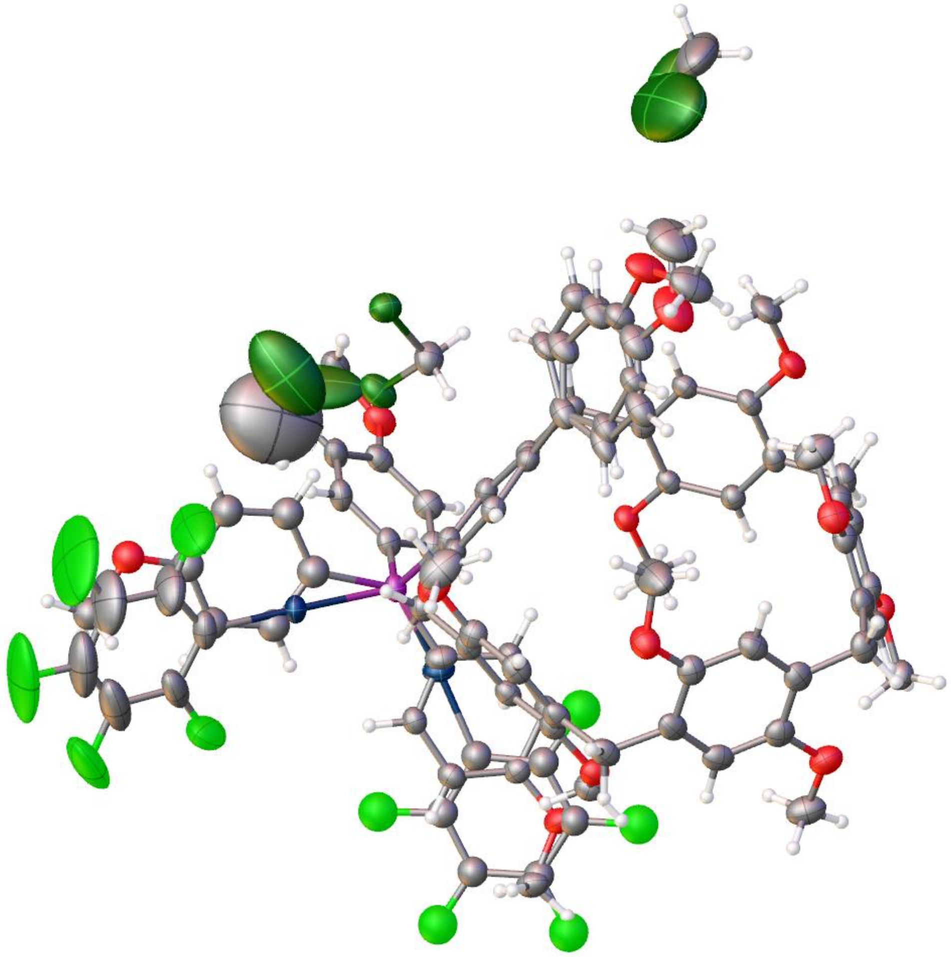

Figure S24. View of the asymmetric unit with displacement ellipsoids at 50 percent probability level.

13. References

1 1 H. Zhu, Q. Li, B. Shi, H. Xing, Y. Sun, S. Lu, L. Shangguan, X. Li, F. Huang and P. J. Stang, Formation of Planar Chiral Platinum Triangles via Pillar[5]arene for Circularly Polarized Luminescence, *J. Am. Chem. Soc.*, 2020, **142**, 17340–17345.

2 W. Xiong, W. Huang, M. Zhang, P. Hu, H. Cui and Q. Zhang, Pillar[5]quinone-Carbon Nanocomposites as High-Capacity Cathodes for Sodium-Ion Batteries, *Chem. Mater.*, 2019, **31**, 8069–8075.

3 K. Wada, S. Ohtani, K. Kato and T. Ogoshi, Stable Planar Chirality of Arylated Pillar[6]Arene and Its Thermal Response, *Tetrahedron Lett.*, 2024, **135**, 154891.

4 R. Uson, A. Laguna, M. Laguna, D. A. Briggs, H. H. Murray and J. P. Fackler, (Tetrahydrothiophene)Au(I) or Gold(III) Complexes, *Inorg. Synth.*, 1989, 85–91.

5 M. C. Blanco Jaimes, F. Rominger, M. M. Pereira, R. M. B. Carrilho, S. A. C. Carabineiro and A. S. K. Hashmi, Highly Active Phosphite Au(I) Catalysts for Intramolecular Hydroalkoxylation, Enyne Cyclization and Furanyne Cyclization, *Chem. Commun.*, 2014, **50**, 4937–4937.

6 A. K. Al-Sa'Ady, C. A. Mcauliffe, R. V. Parish, J. A. Sandbank, R. A. Potts and W. F. Schneider, A General Synthesis for Au(I) Complexes, *Inorg. Synth.*, 1985, 191–194.

7 N. C. Bruno, M. T. Tudge and S. L. Buchwald, Design and Preparation of New Palladium Precatalysts for C–C and C–N Cross-Coupling Reactions, *Chem. Sci.*, 2013, **4**, 916–920.

8 C. A. Busacca, J. C. Lorenz, N. Grinberg, N. Haddad, M. Hrapchak, B. Latli, H. Lee, P. Sabila, A. Saha, M. Sarvestani, S. Shen, R. Varsolona, X. Wei and C. H. Senanayake, A Superior Method for the Reduction of Secondary Phosphine Oxides, *Org. Lett.*, 2005, **7**, 4277–4280.

9 P. W. Miller, M. Nieuwenhuyzen, X. Xu and S. L. James, Assembly of a Coordination Cage with Four Aromatic Channel Receptors on the Outside, *Chem. Commun.*, 2002, **18**, 2008–2009.

10 A. Panossian, H. Fernández-Pérez, D. Popa and A. Vidal-Ferran, Highly Modular P-OP Ligands in Asymmetric Allylic Substitution, *Tetrahedron: Asymmetry*, 2010, **21**, 2281–2288.

11 S. Ganss and B. Breit, Enantioselective Rhodium-Catalyzed Atom-Economical Macrolactonization, *Angew. Chem., Int. Ed.*, 2016, **55**, 9738–9742.

12 T. Imamoto, T. Kusumoto, N. Suzuki and K. Sato, Phosphine Oxides and Lithium Aluminum Hydride-Sodium Borohydride-Cerium(III) Chloride: Synthesis and Reactions of Phosphine-Boranes, *J. Am. Chem. Soc.*, 1985, **107**, 5301–5303.

13 N. Takeda and T. Imamoto, Use of Cerium(III) Chloride in the Reactions of Carbonyl Compounds with Organolithiums or Grignard Reagents for the Suppression of Abnormal Reactions: 1-Butyl-1,2,3,4-Tetrahydro-1-Naphthol, *Org. Synth.*, 1999, **76**, 228–238.

14 (a) C. Nieto-Oberhuber, M. P. Muñoz, S. López, E. Jimenéz-Núñez, C. Nevado, E. Herrero-Gómez, M. Raducan and A. M. Echavarren*,* Gold(I)-Catalyzed Cyclizations of 1,6-Enynes: Alkoxycyclizations and exo/endo Skeletal Rearrangements, *Chem. Eur. J.*, 2006, **12**, 1677–1693; (b) K. Muratov and F. Gagosz, Confinement-Induced Selectivities in Gold(I) Catalysis—the Benefit of Using Bulky Tri-(Ortho-Biaryl)Phosphine Ligands, *Angew. Chem., Int. Ed.,* 2022, **61**, e202203452.

15 O. V. Dolomanov, L. J. Bourhis, R. J. Gildea, J. A. K. Howard and H. Puschmann, OLEX2: a complete structure solution, refinement and analysis program, *J. Appl. Crystallogr.*, 2009, **42**, 339–341.

16 G. M. Sheldrick, SHELXT – Integrated space-group and crystal-structure determination, *Acta Crystallogr., Sect. A*, 2015, **71**, 3–8.

17 G. M. Sheldrick, Crystal structure refinement with SHELXL, *Acta Crystallogr., Sect. C*, 2015, **71**, 3–8.

14. NMR spectra for new compounds

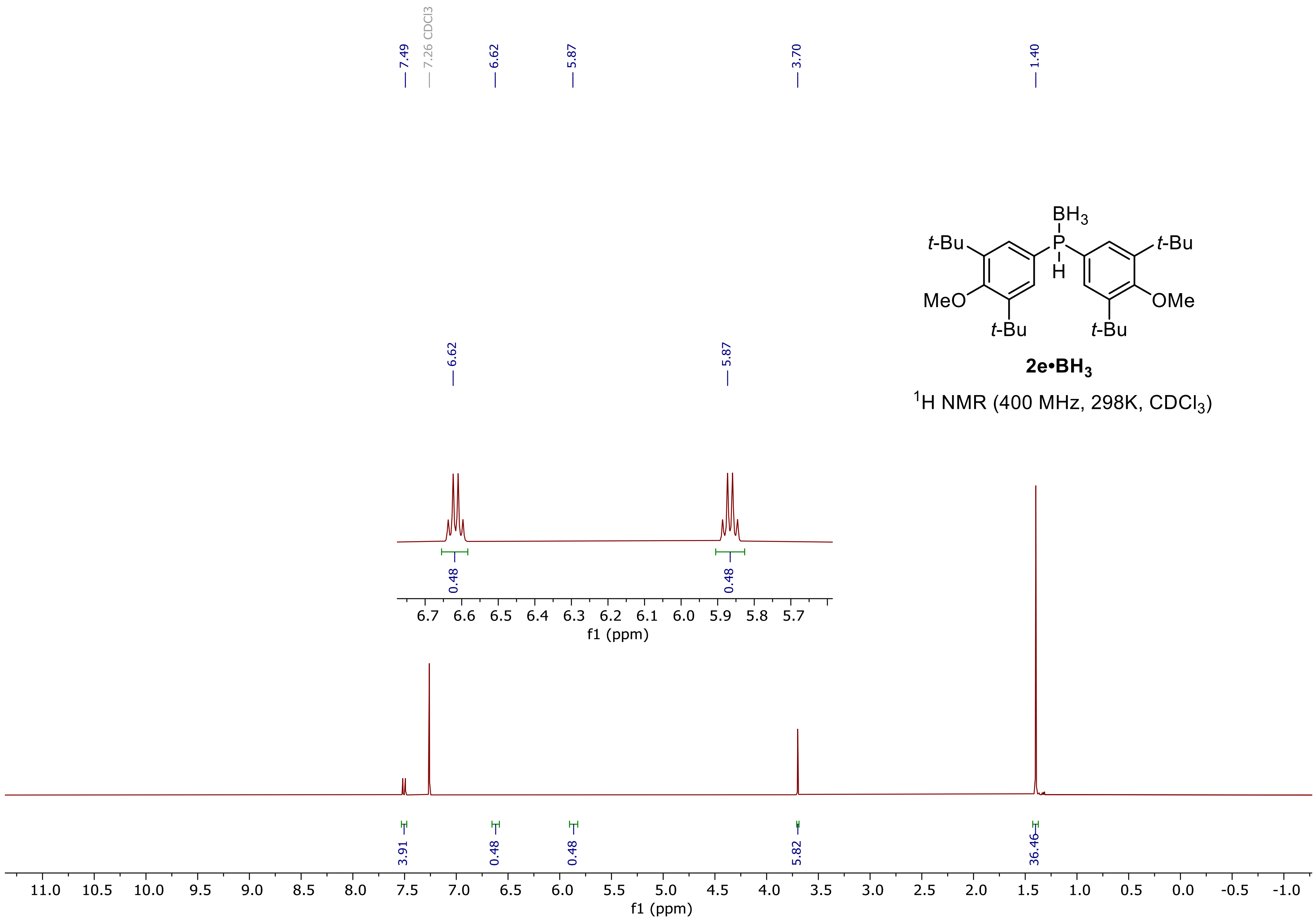

2e•BH$_3$

^{1}H NMR (400 MHz, 298K, CDCl$_3$)

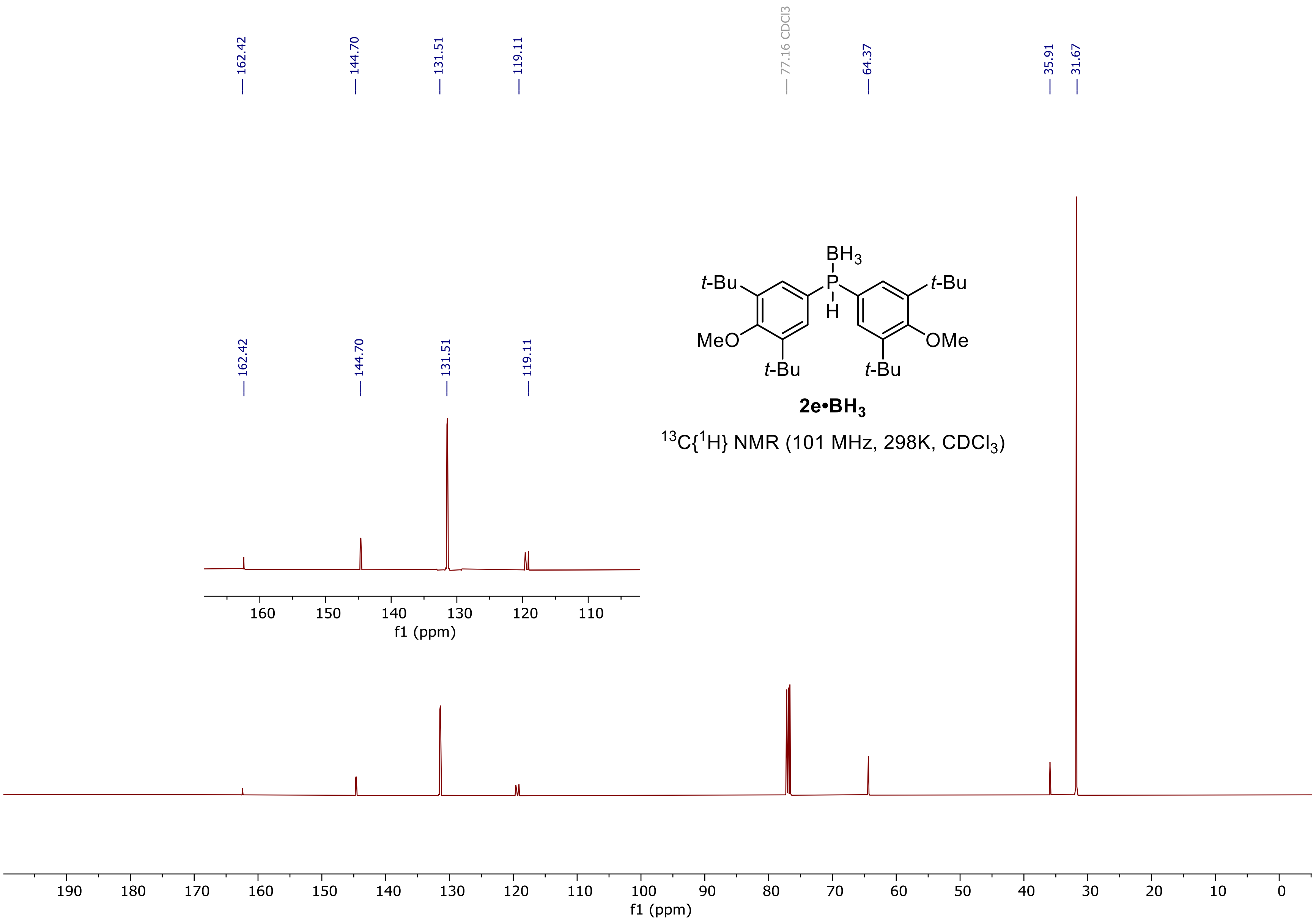

2e•BH$_3$

$^{13}C\{^1H\}$ NMR (101 MHz, 298K, $CDCl_3$)

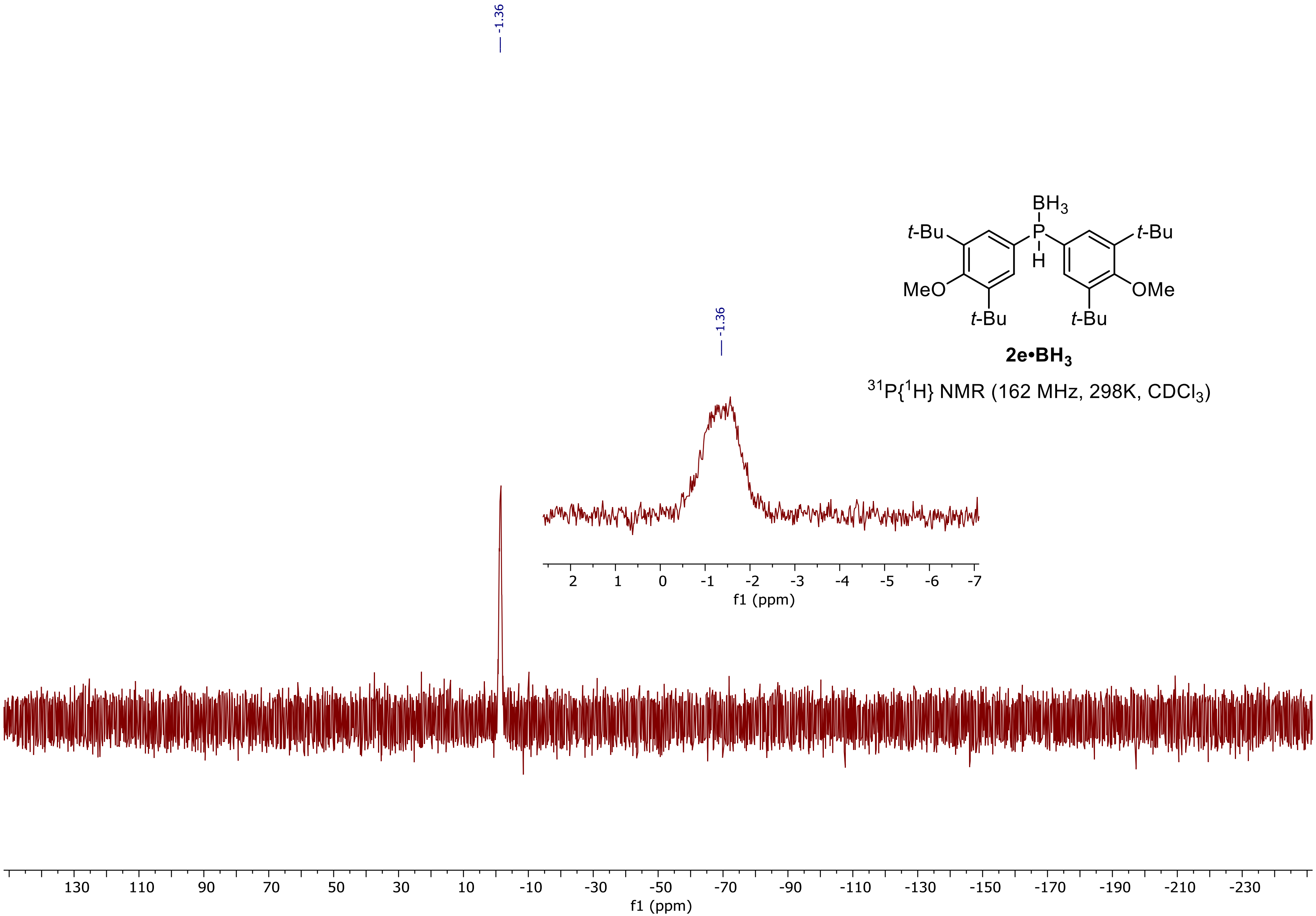

2e•BH$_3$

^{31}P{^{1}H} NMR (162 MHz, 298K, CDCl$_3$)

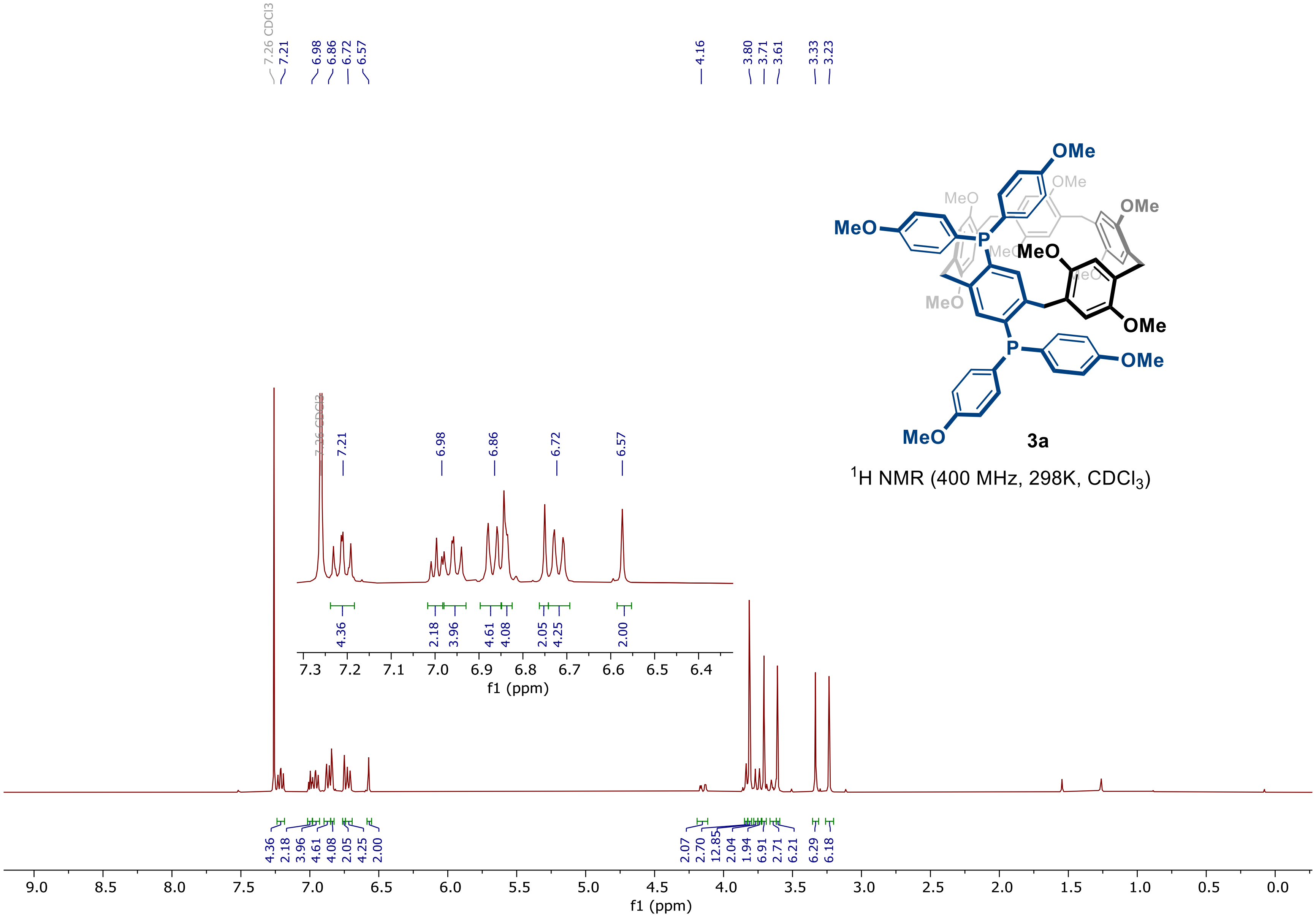

3a

^1H NMR (400 MHz, 298K, $CDCl_3$)

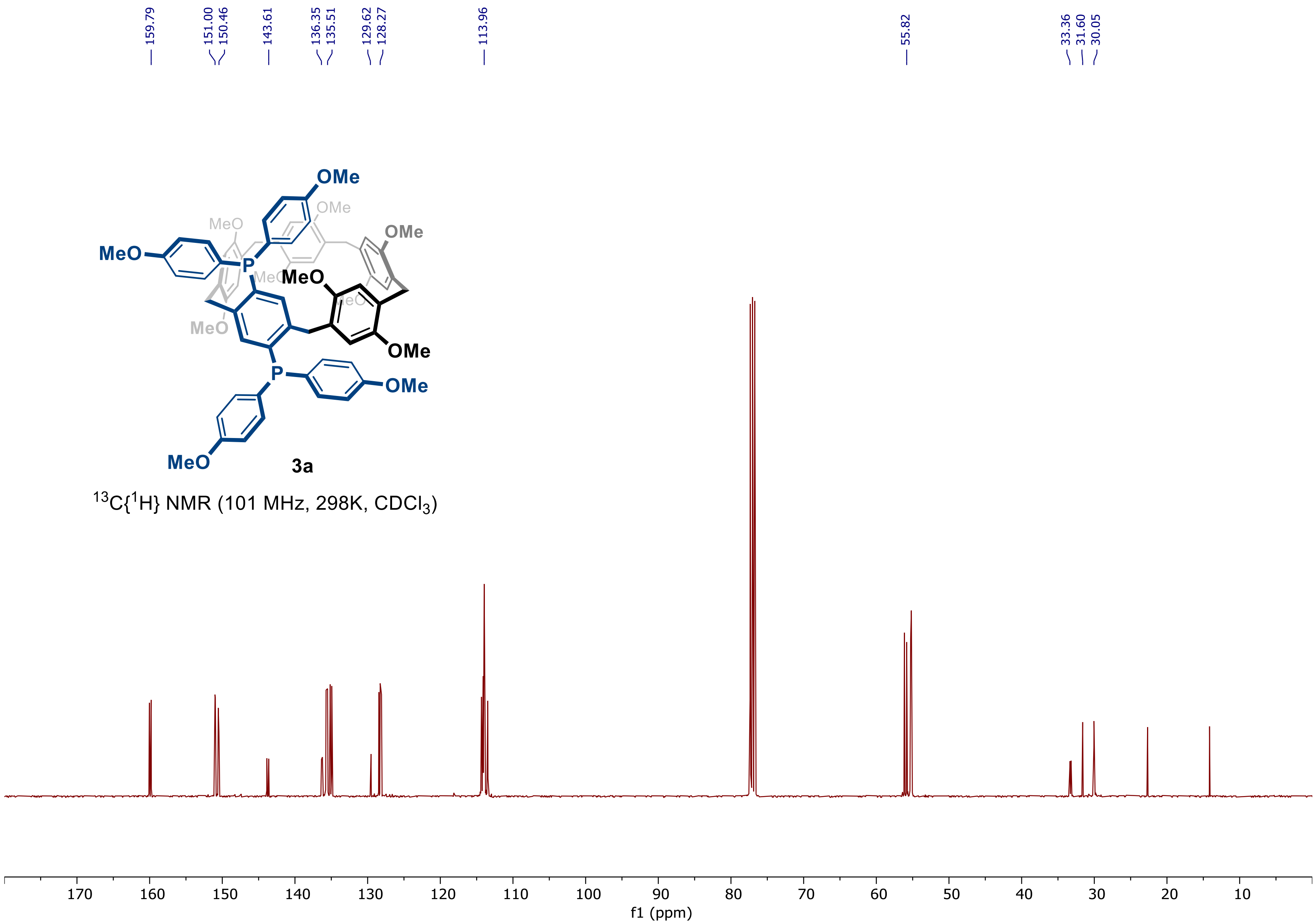

$^{13}C\{^1H\}$ NMR (101 MHz, 298K, $CDCl_3$)

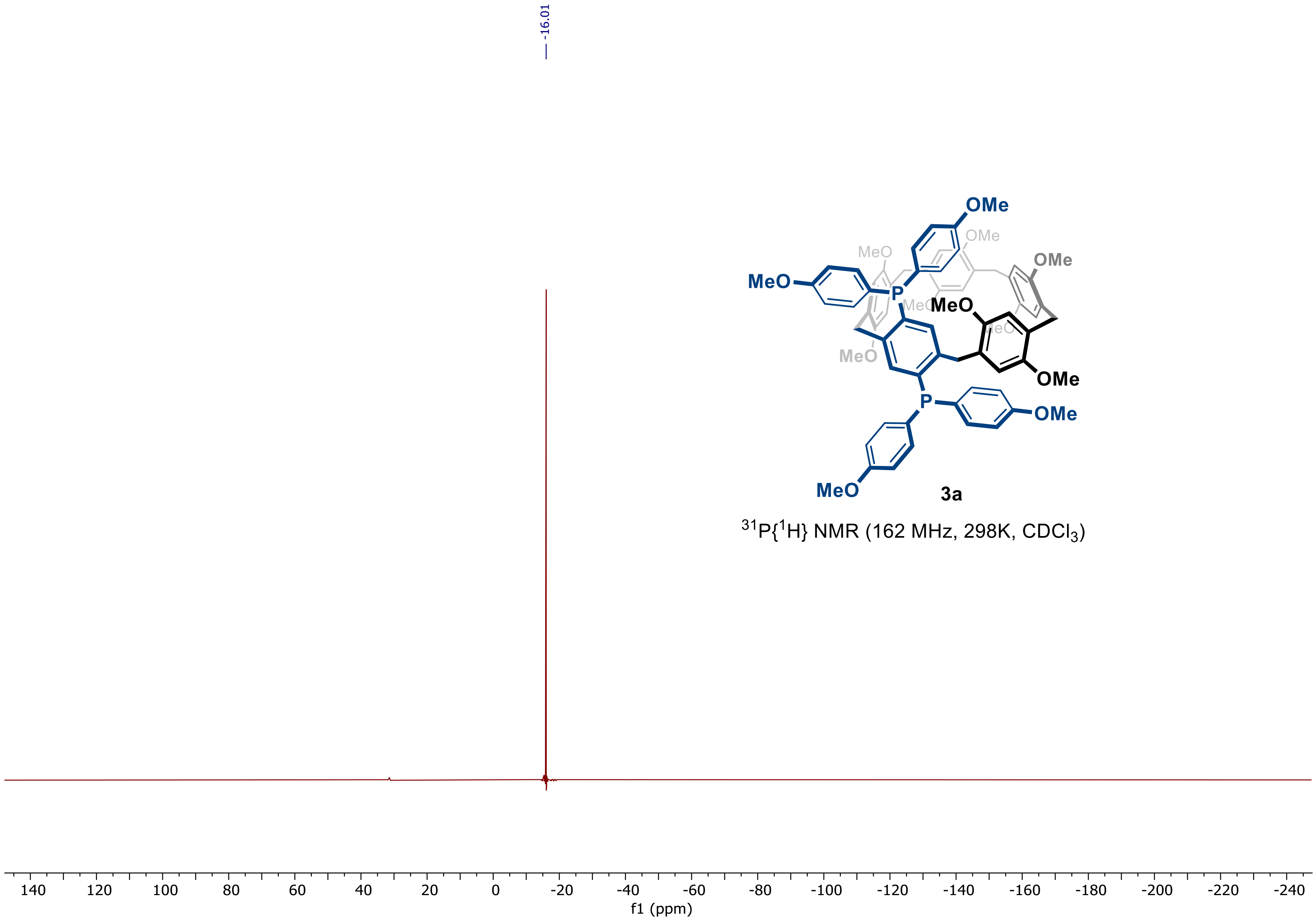

3a

$^{31}P\{^1H\}$ NMR (162 MHz, 298K, $CDCl_3$)

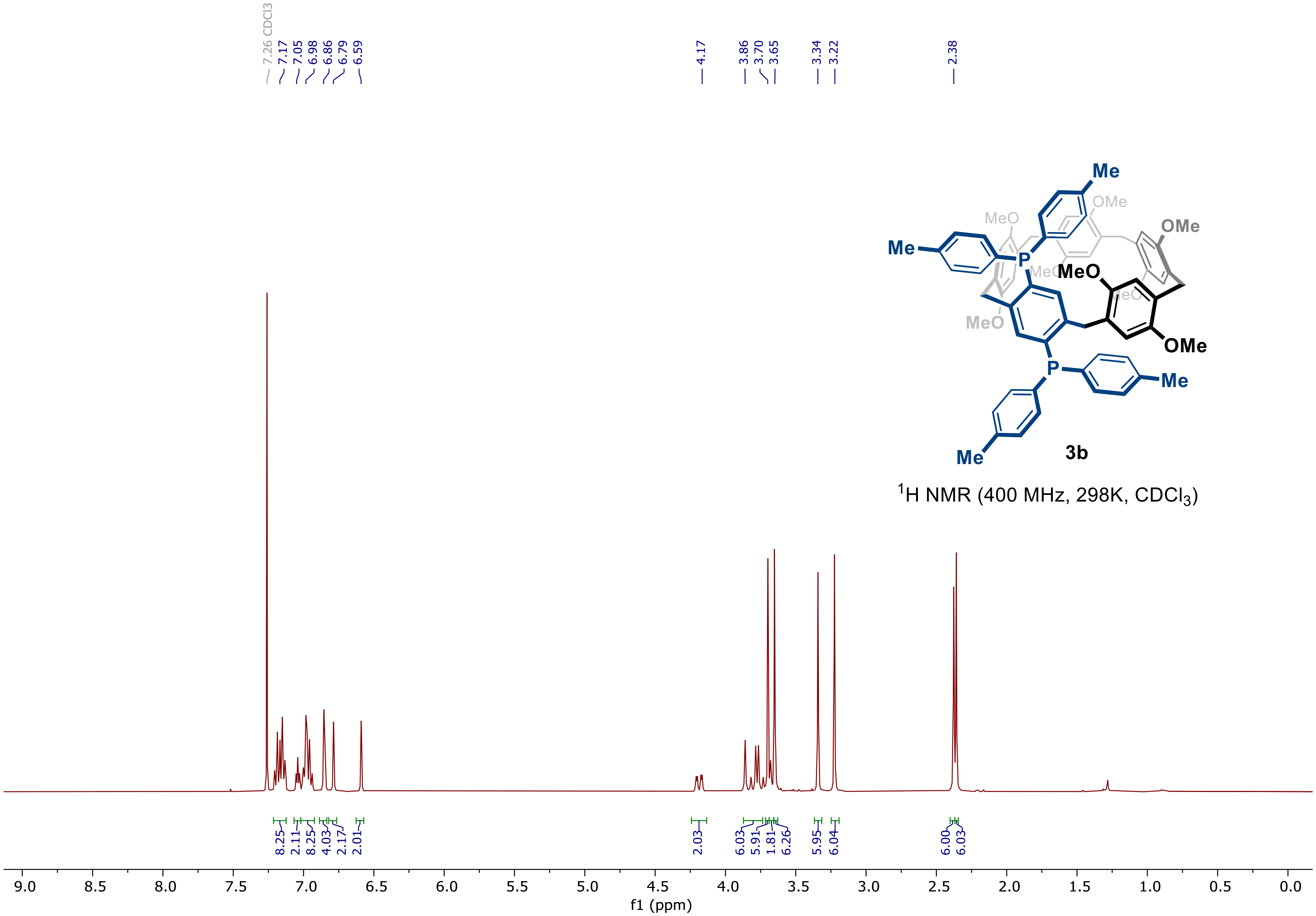
7.26 CDCl3
7.17
7.05
6.98
6.86
6.79
6.59
4.17
3.86
3.70
3.65
3.34
3.22
2.38
Me
Me
Me
Me
P
P
MeO
OMe
OMe
MeO
MeO
OMe
3b
¹H NMR (400 MHz, 298K, CDCl3)
8.25
2.11
8.25
4.03
2.17
2.01
2.03
6.03
5.91
1.81
6.26
5.95
6.04
6.00
6.03
9.0
8.5
8.0
7.5
7.0
6.5
6.0
5.5
5.0
4.5
4.0
3.5
3.0
2.5
2.0
1.5
1.0
0.5
0.0
f1 (ppm)

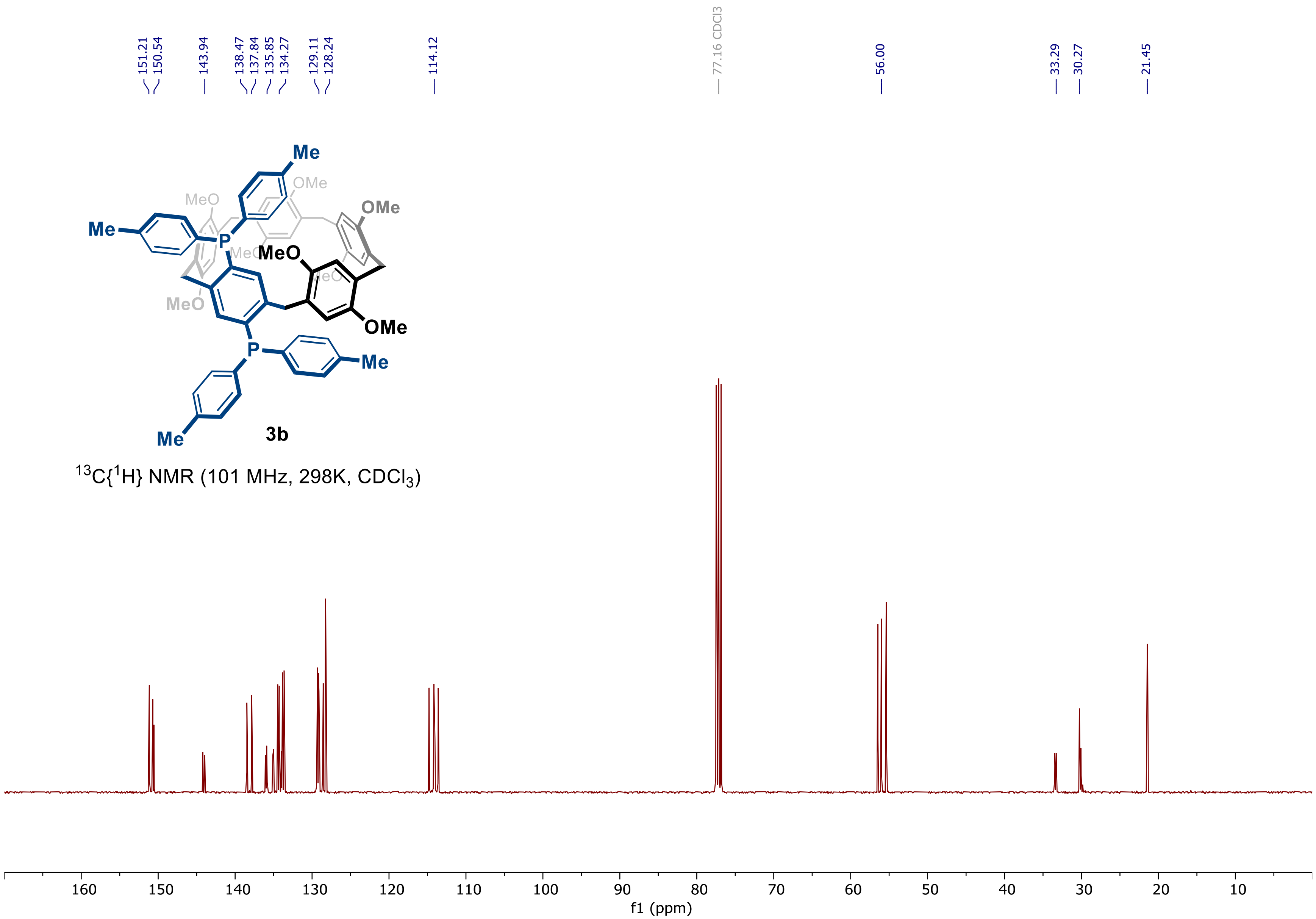
151.21
150.54
143.94
138.47
137.84
135.85
134.27
129.11
128.24
114.12
77.16 CDCl3
56.00
33.29
30.27
21.45
Me
OMe
MeO
OMe
Me
P
MeO
MeO
MeO
OMe
P
Me
Me
3b
$^{13}C\{^{1}H\}$ NMR (101 MHz, 298K, $CDCl_3$)
160
150
140
130
120
110
100
90
80
70
60
50
40
30
20
10
f1 (ppm)

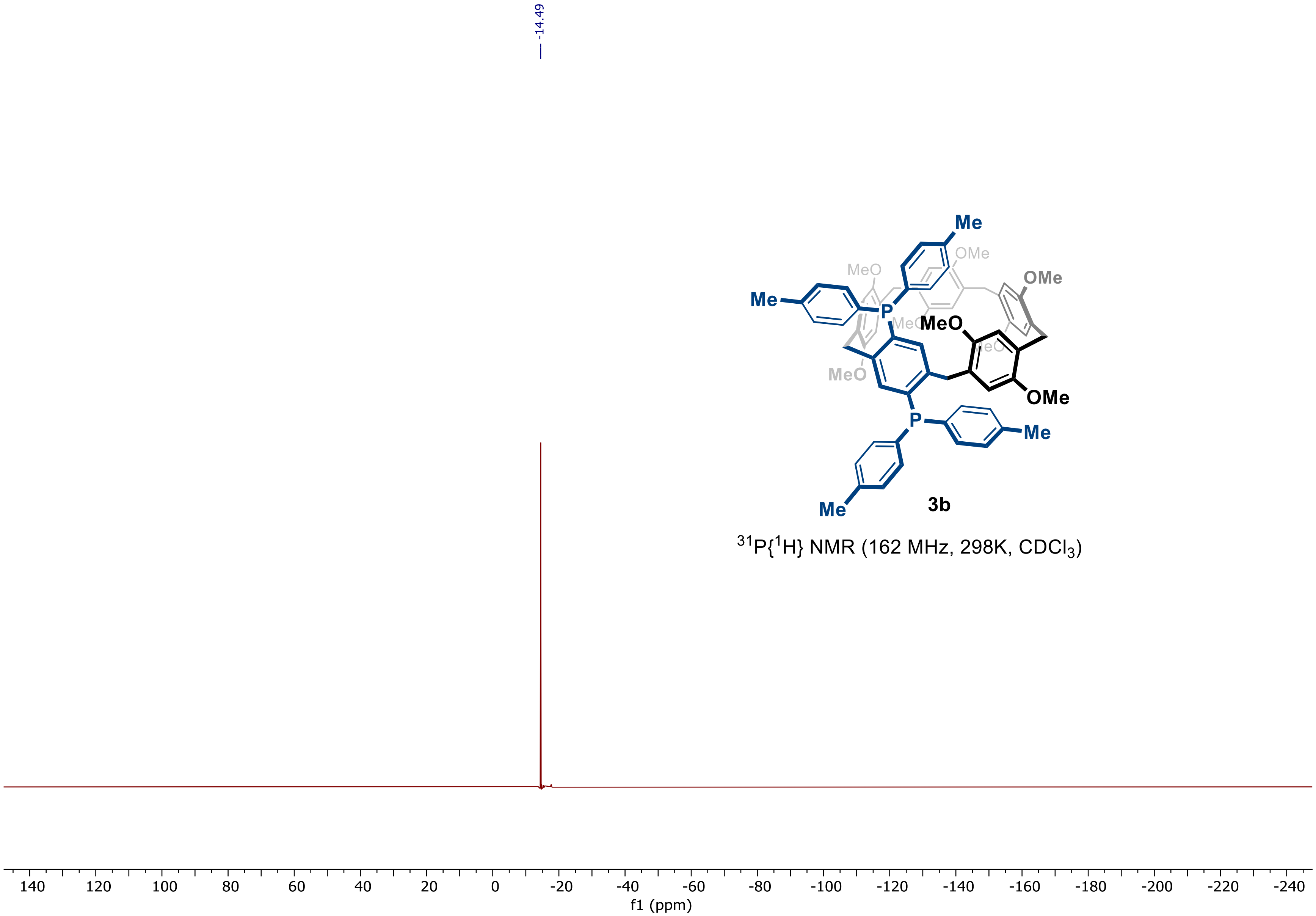

3b

$^{31}P\{^1H\}$ NMR (162 MHz, 298K, $CDCl_3$)

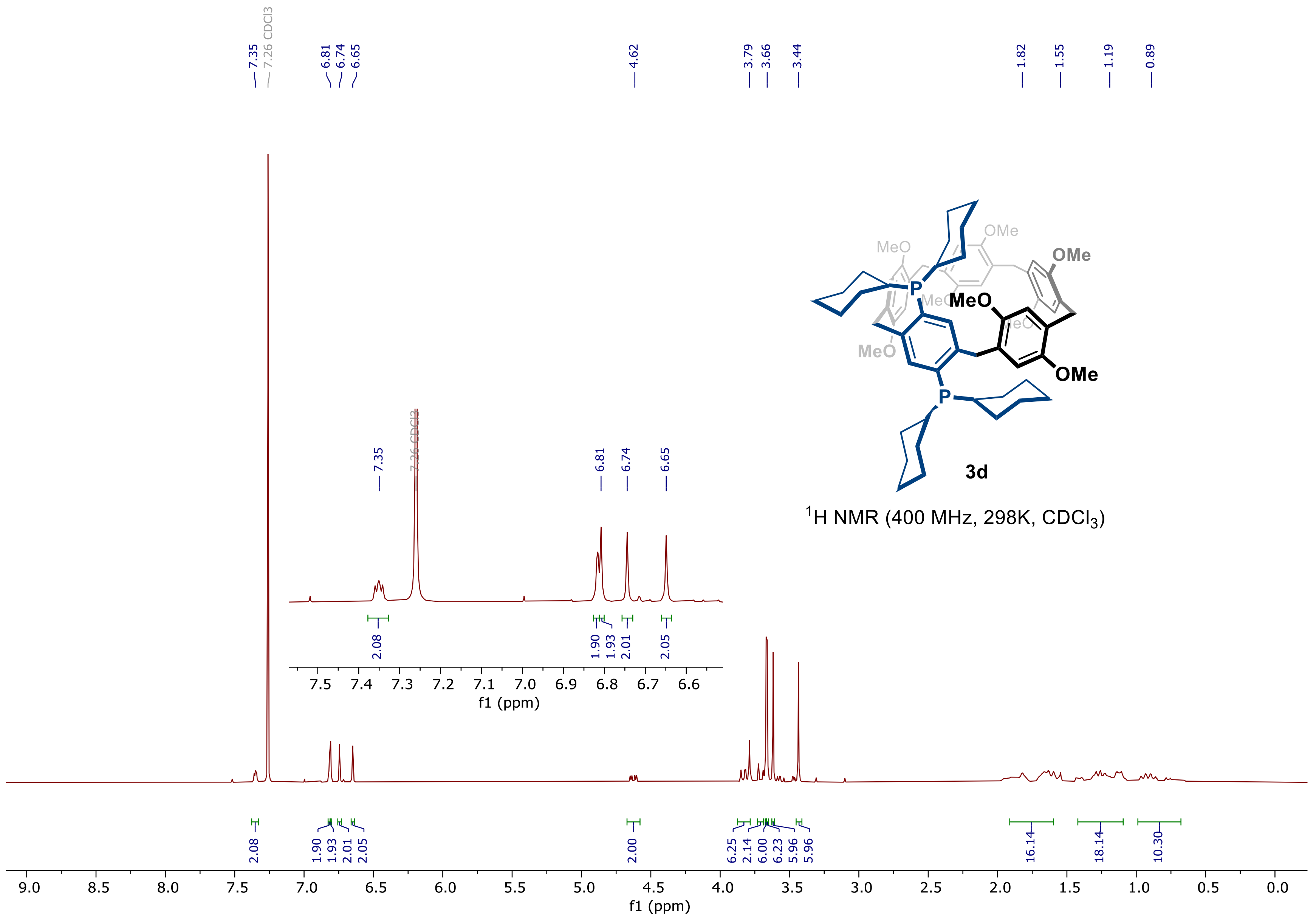

^{1}H NMR (400 MHz, 298K, $CDCl_3$)

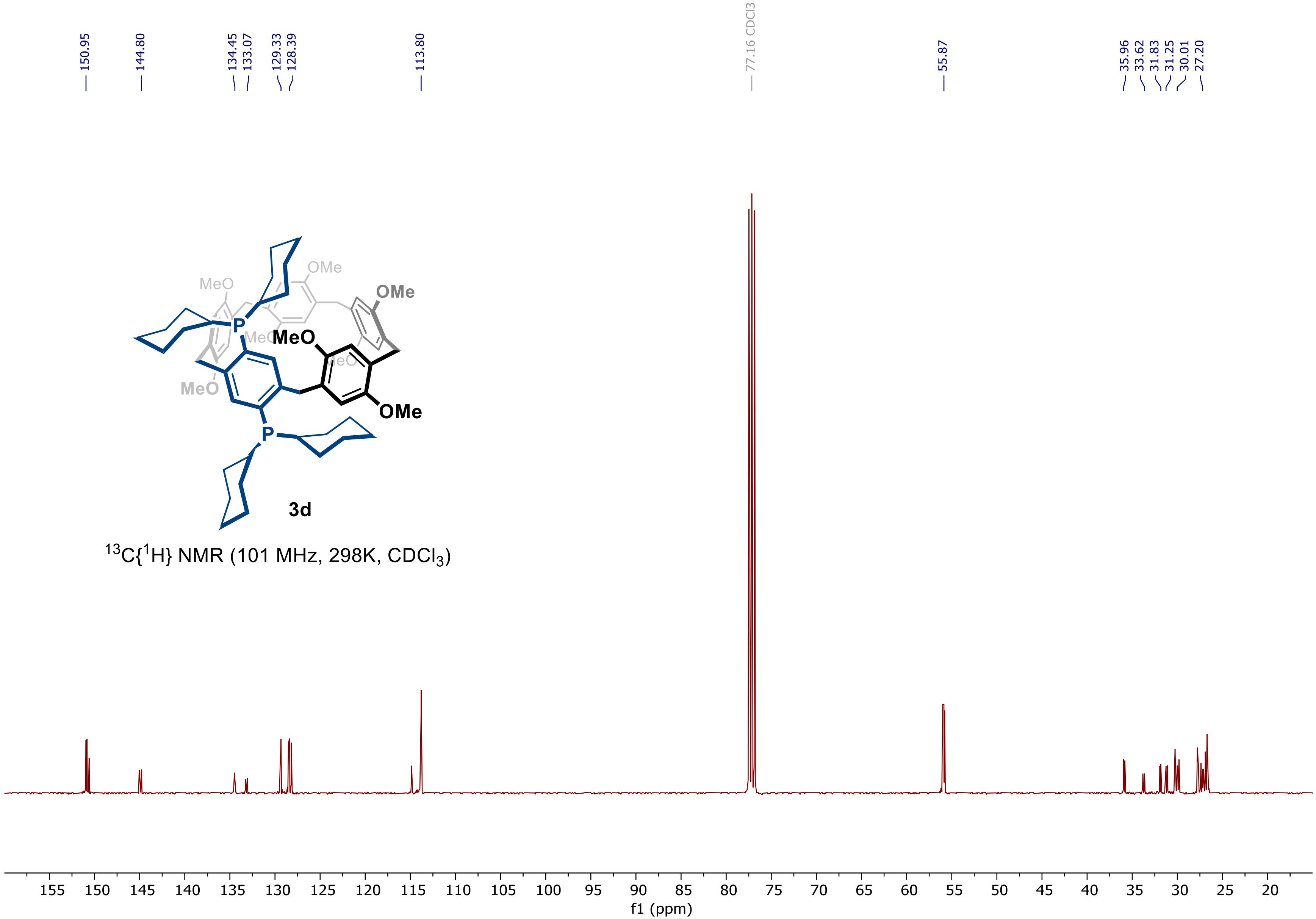
150.95
144.80
134.45
133.07
129.33
128.39
113.80
77.16 CDCl3
55.87
35.96
33.62
31.83
31.25
30.01
27.20
MeO
OMe
OMe
MeO
MeO
OMe
P
P
3d
$^{13}C\{^1H\}$ NMR (101 MHz, 298K, $CDCl_3$)
155 150 145 140 135 130 125 120 115 110 105 100 95 90 85 80 75 70 65 60 55 50 45 40 35 30 25 20
f1 (ppm)

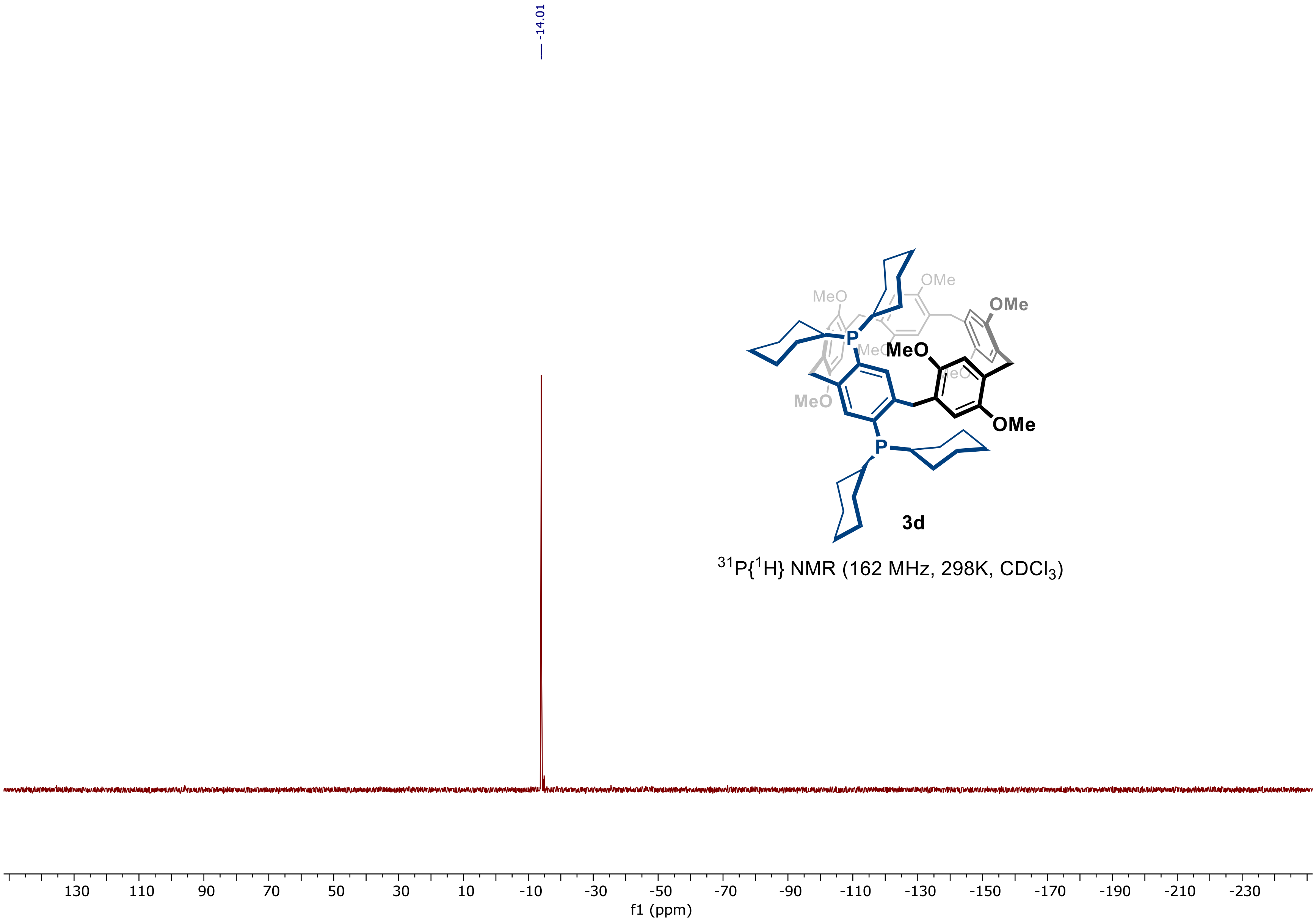

3d

$^{31}P\{^{1}H\}$ NMR (162 MHz, 298K, $CDCl_3$)

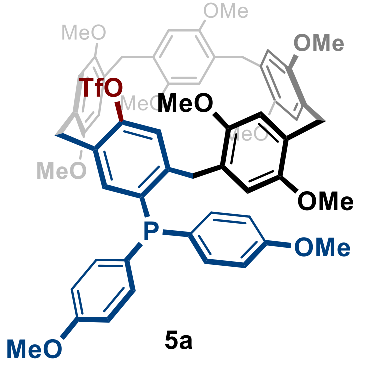

5a

^{1}H NMR (400 MHz, 298K, $CDCl_3$)

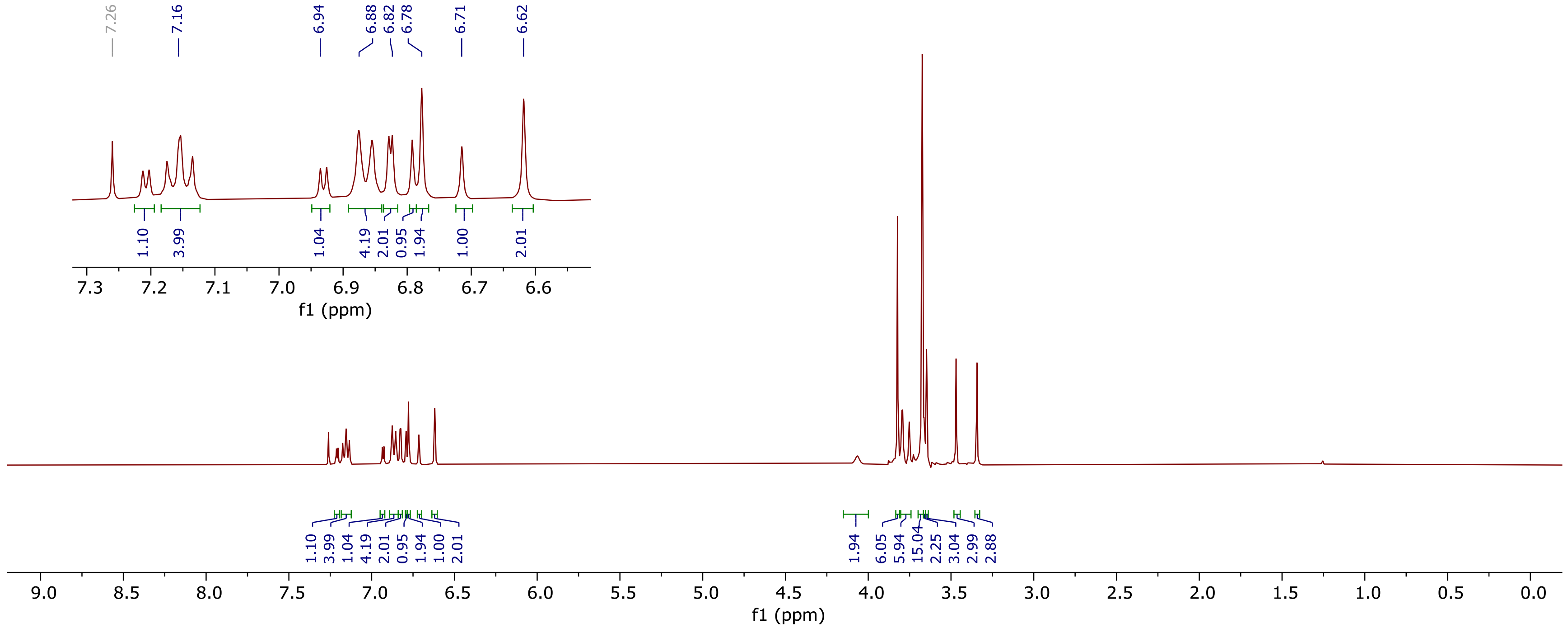

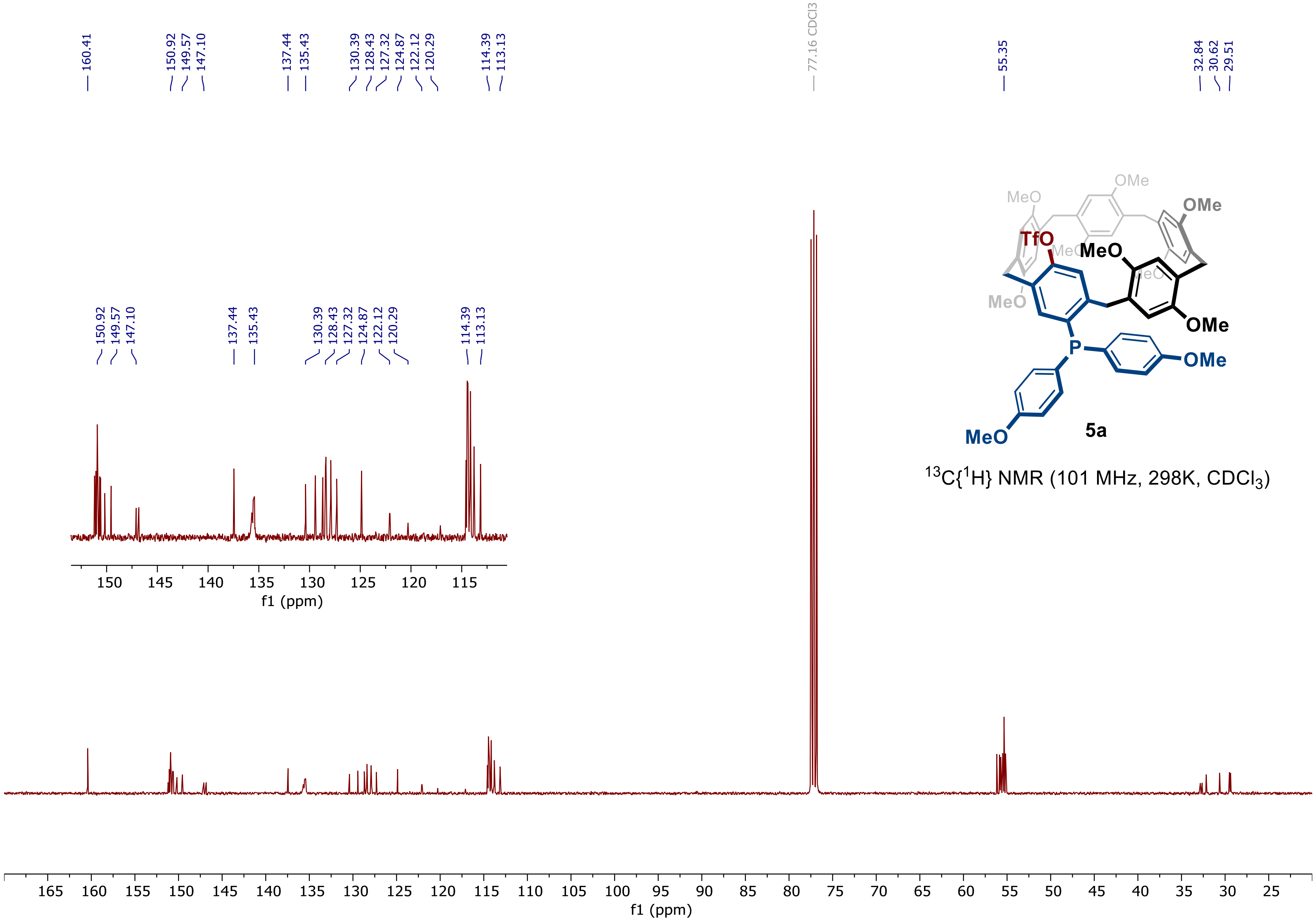

5a

$^{13}C\{^1H\}$ NMR (101 MHz, 298K, $CDCl_3$)

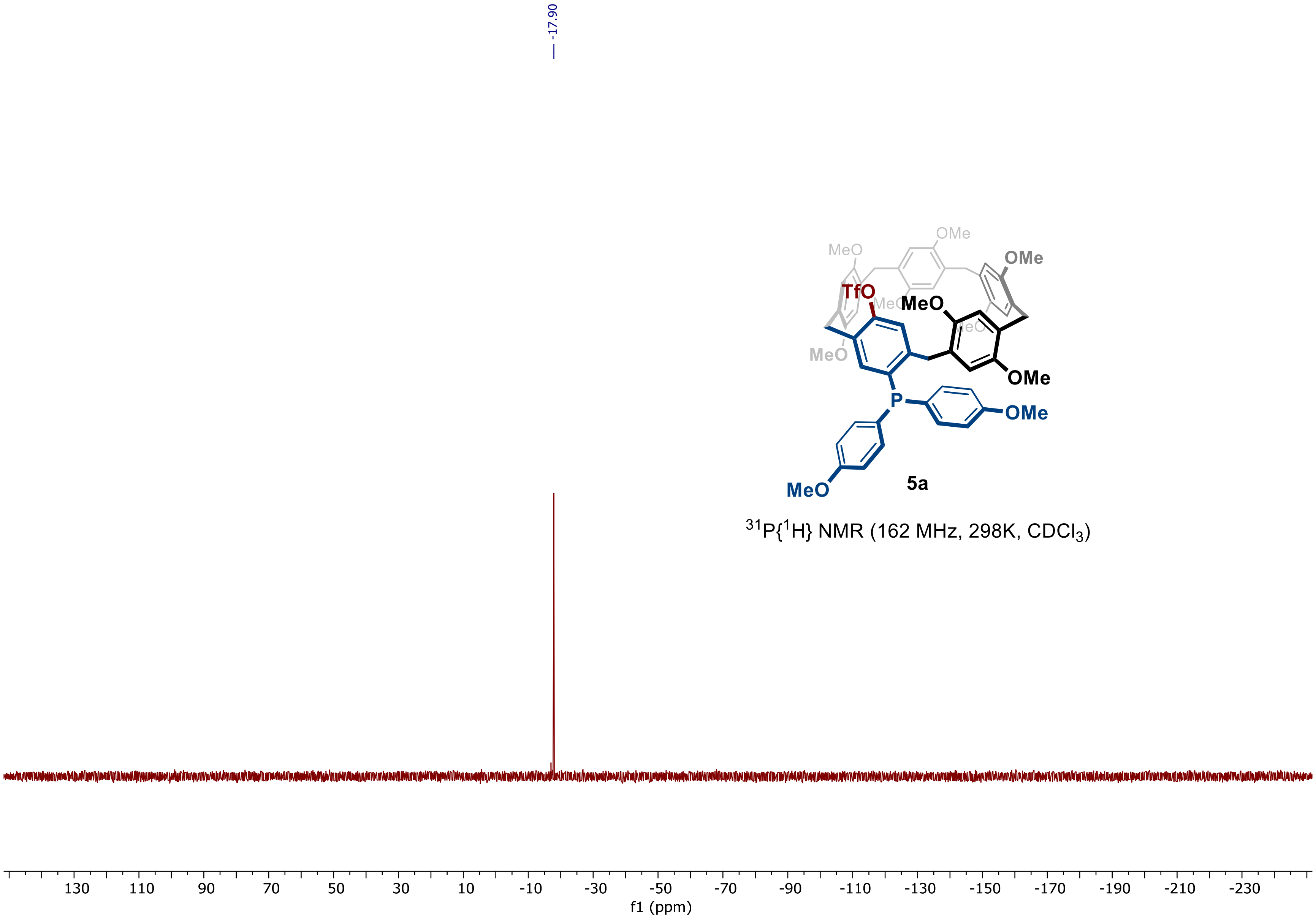

5a

$^{31}P\{^{1}H\}$ NMR (162 MHz, 298K, $CDCl_3$)

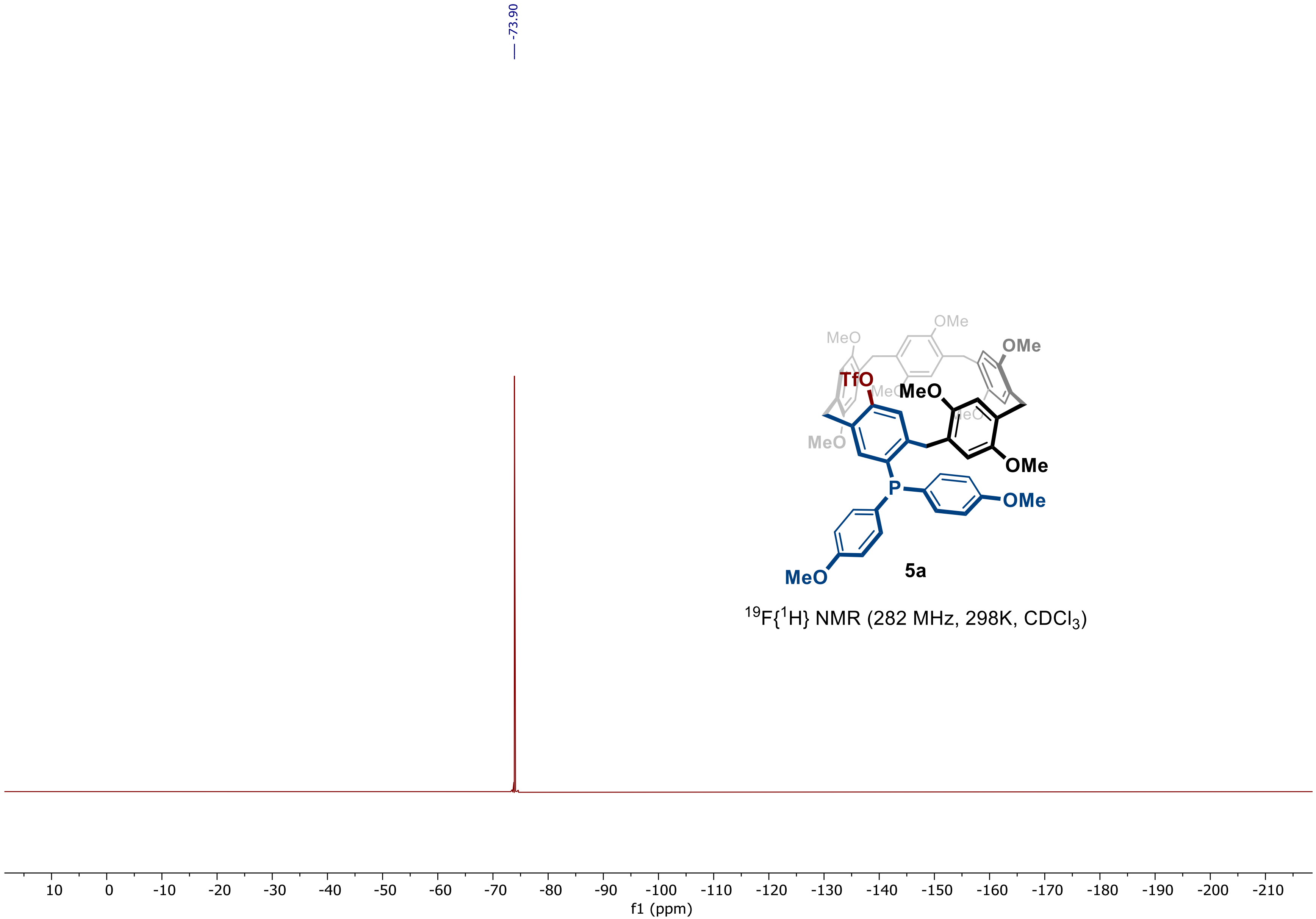

$^{19}F\{^{1}H\}$ NMR (282 MHz, 298K, $CDCl_3$)

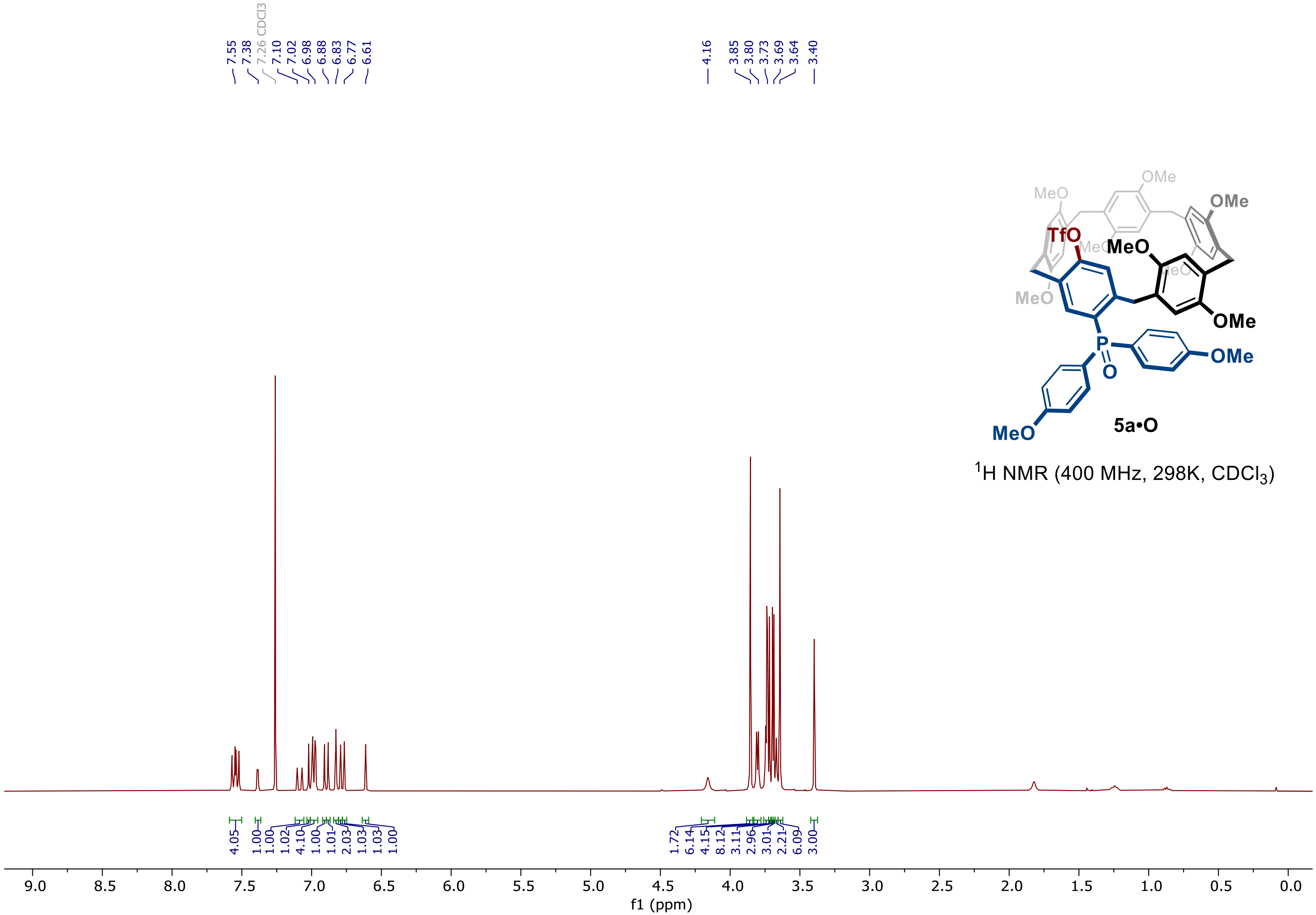

5a•O

^{1}H NMR (400 MHz, 298K, $CDCl_3$)

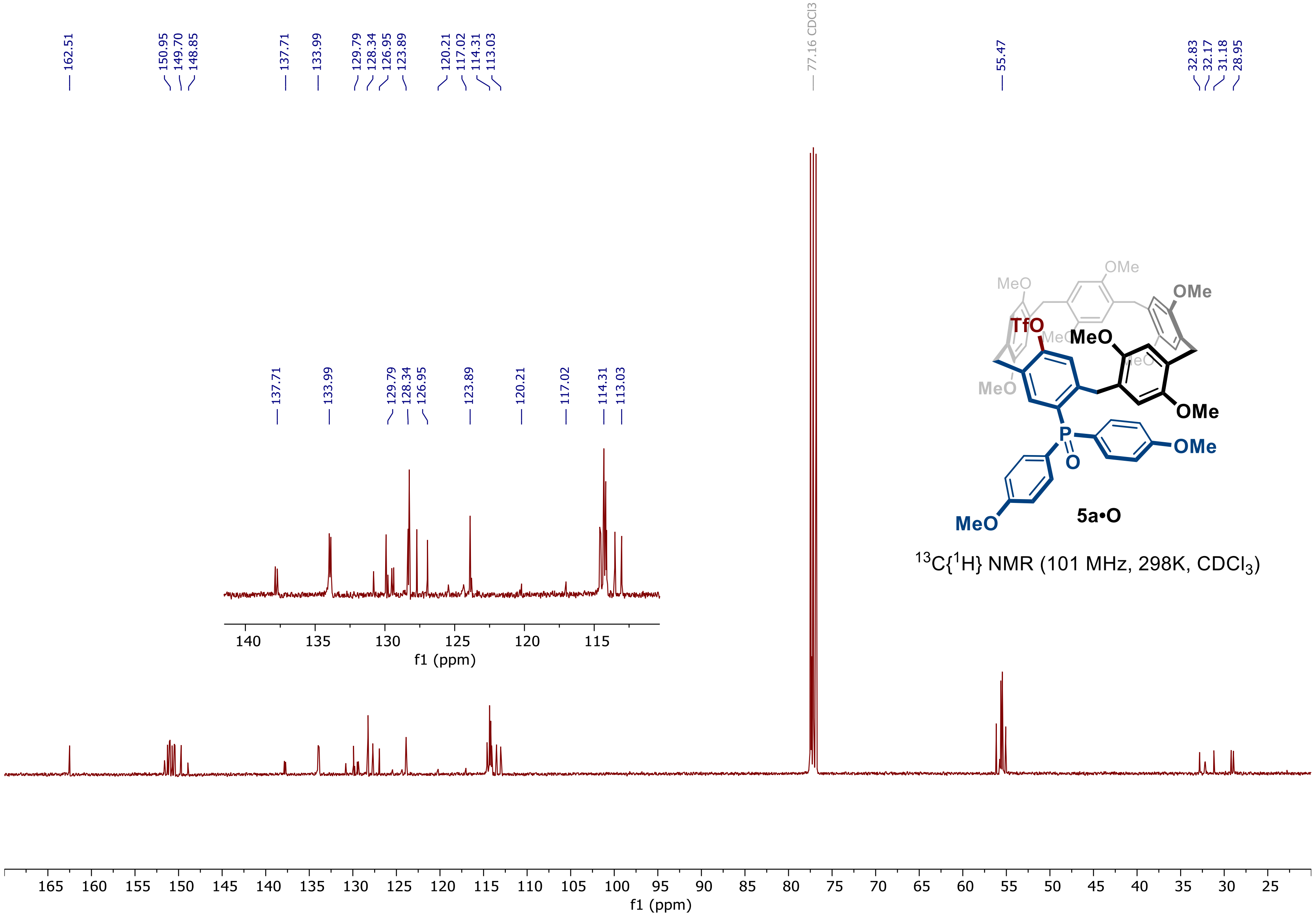
162.51
150.95
149.70
148.85
137.71
133.99
129.79
128.34
126.95
123.89
120.21
117.02
114.31
113.03
77.16 CDCl3
55.47
32.83
32.17
31.18
28.95
MeO
OMe
OMe
TfO
MeO
MeO
OMe
P
O
OMe
MeO
5a•O
$^{13}C\{^{1}H\}$ NMR (101 MHz, 298K, $CDCl_3$)
f1 (ppm)

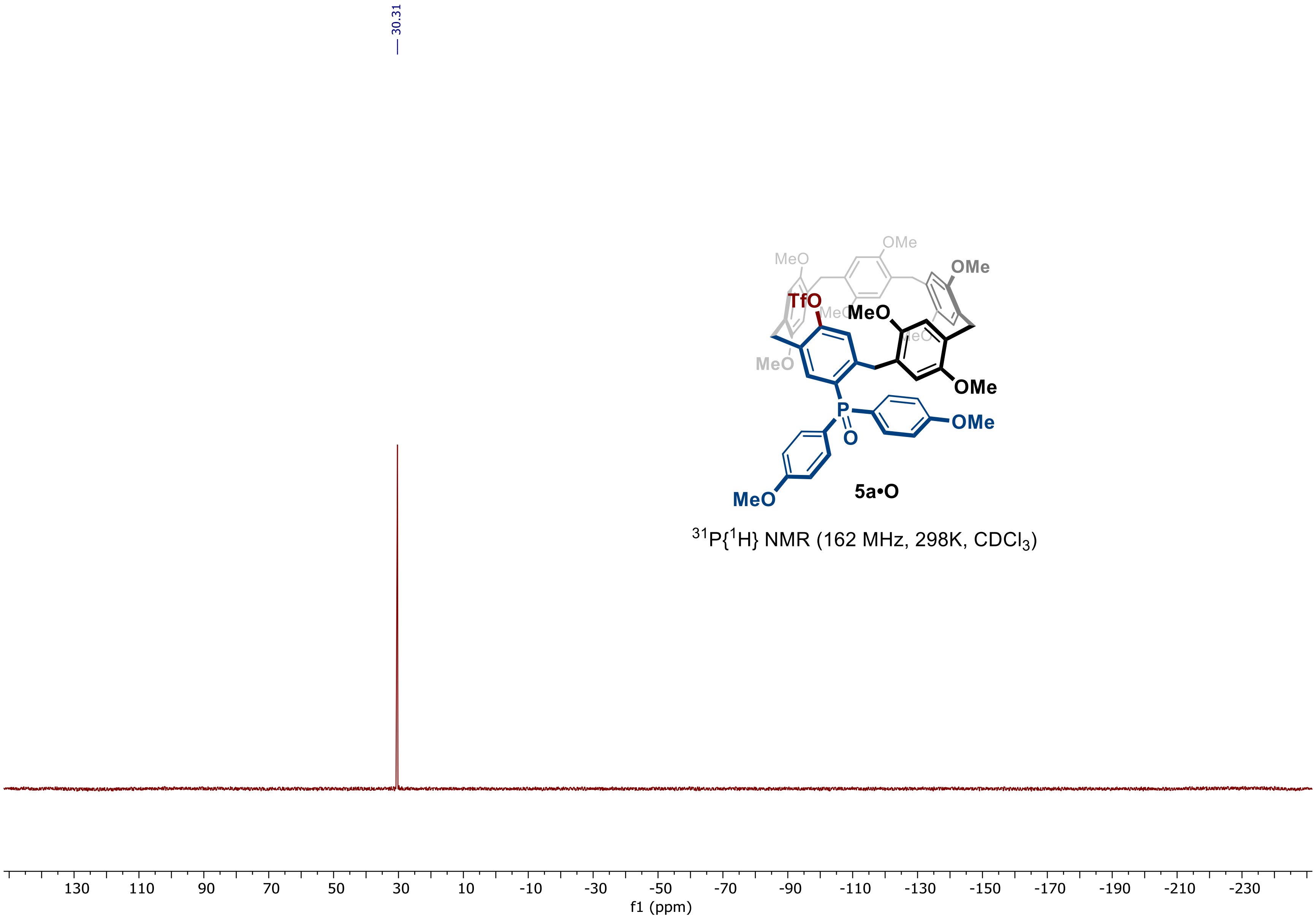

5a•O

$^{31}P\{^{1}H\}$ NMR (162 MHz, 298K, $CDCl_3$)

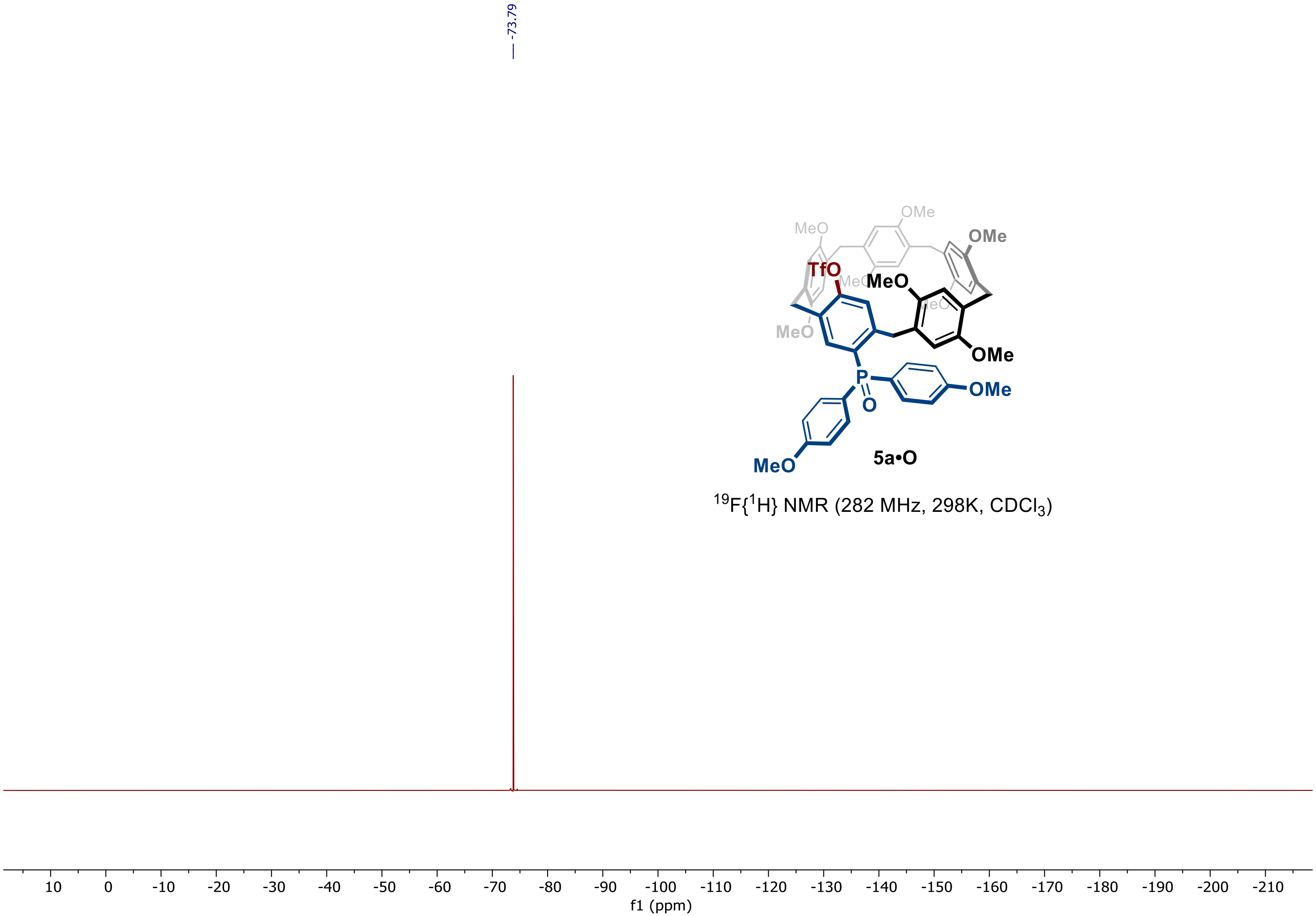

$^{19}F\{^{1}H\}$ NMR (282 MHz, 298K, $CDCl_3$)

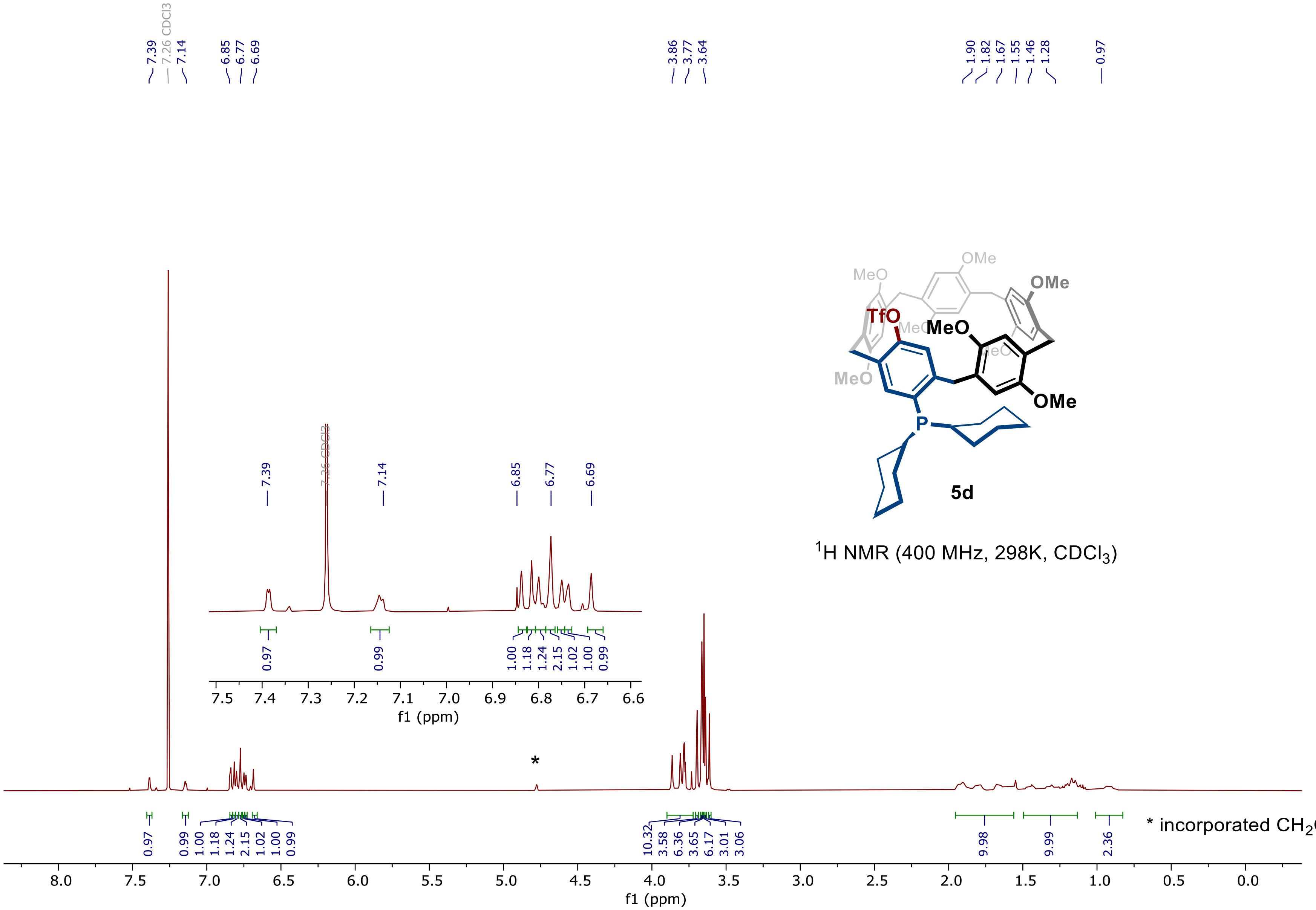

5d

^{1}H NMR (400 MHz, 298K, $CDCl_3$)

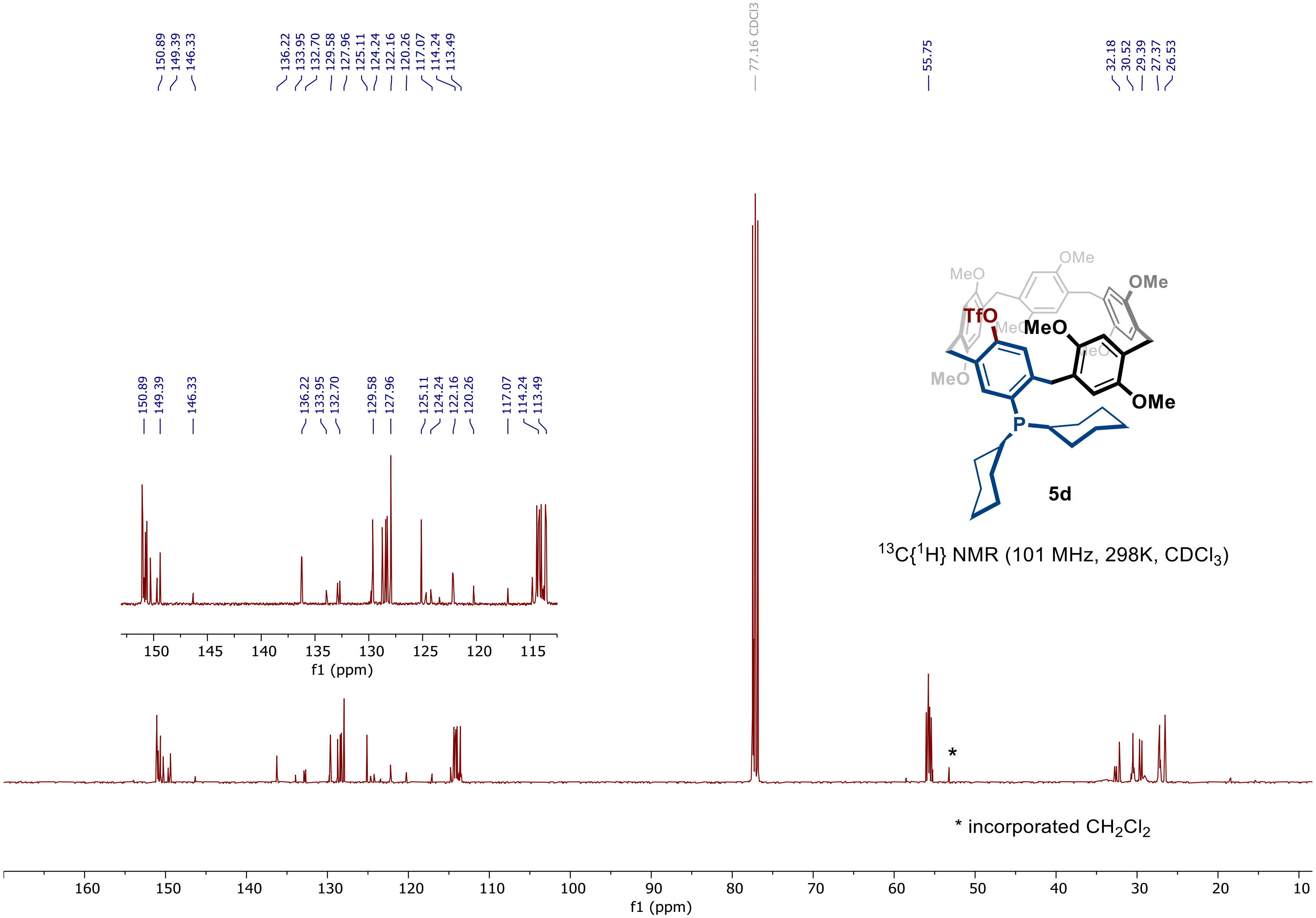

$^{13}C\{^{1}H\}$ NMR (101 MHz, 298K, $CDCl_3$)

* incorporated CH_2Cl_2

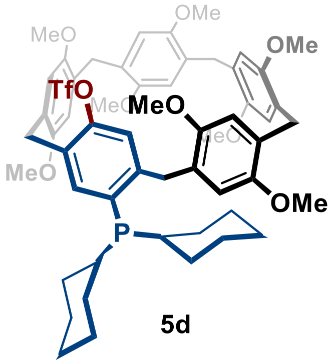

5d

$^{31}P\{^{1}H\}$ NMR (162 MHz, 298K, $CDCl_3$)

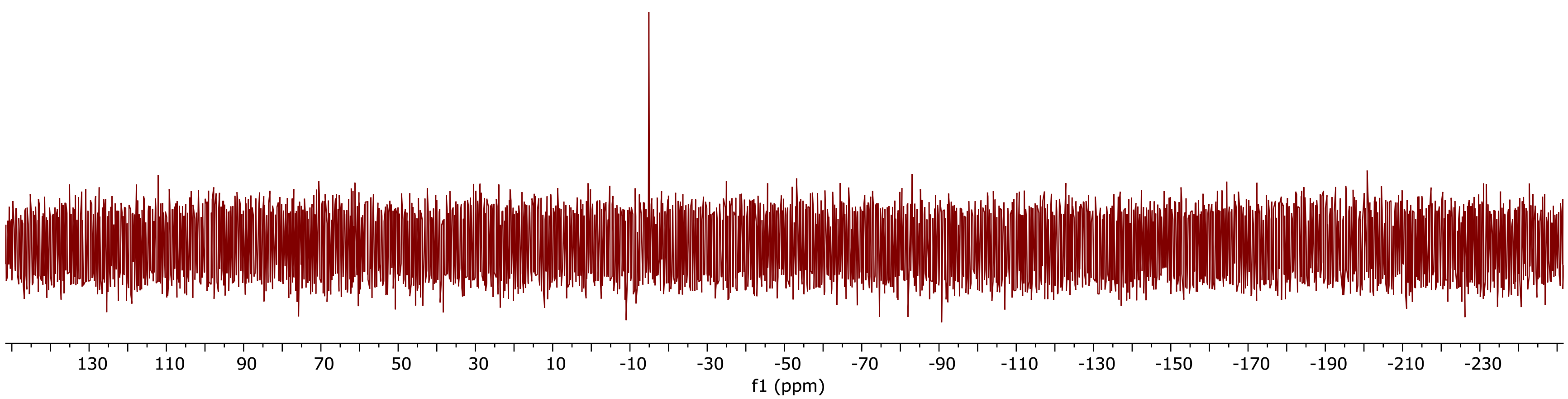

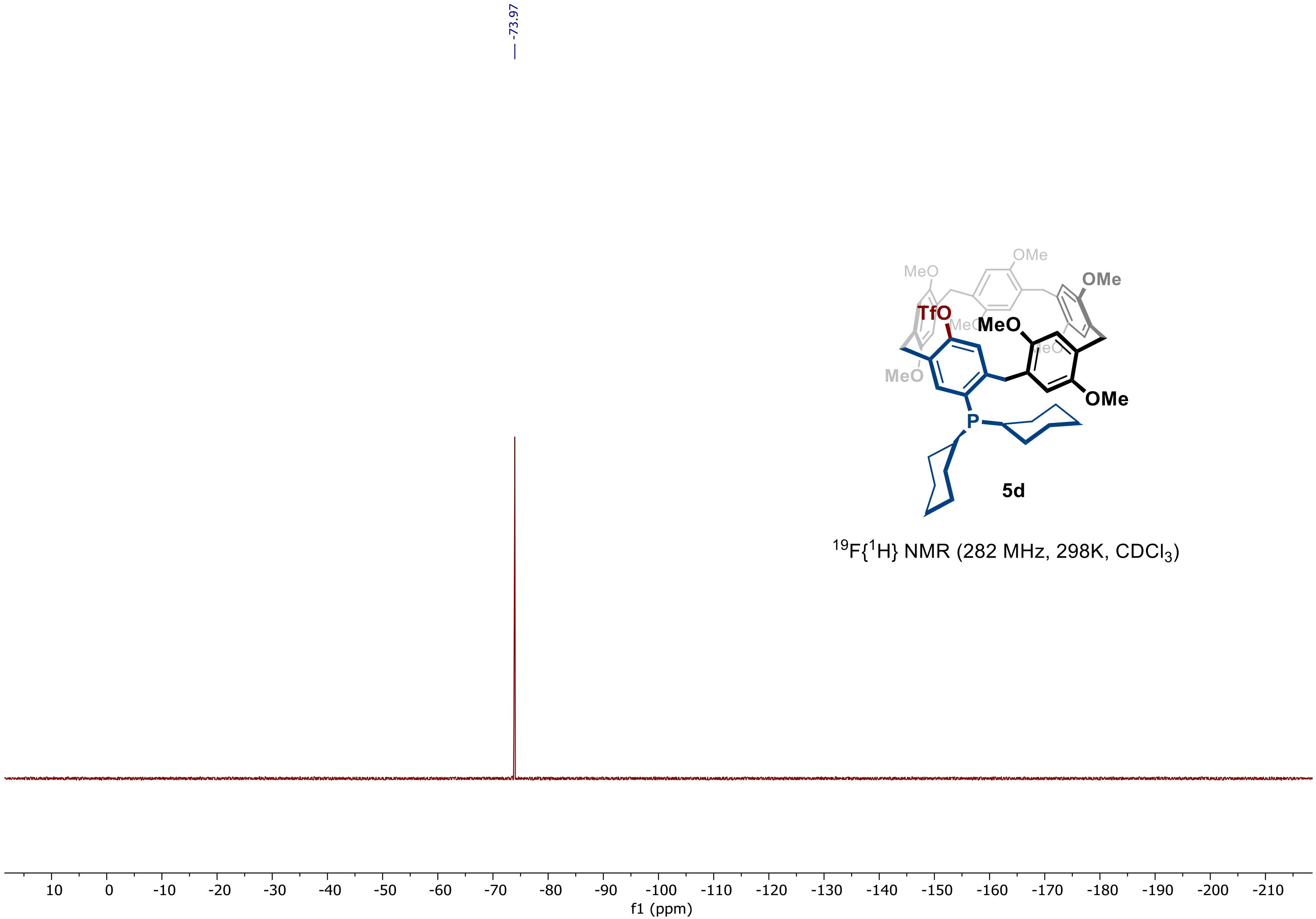

5d

$^{19}F\{^{1}H\}$ NMR (282 MHz, 298K, $CDCl_3$)

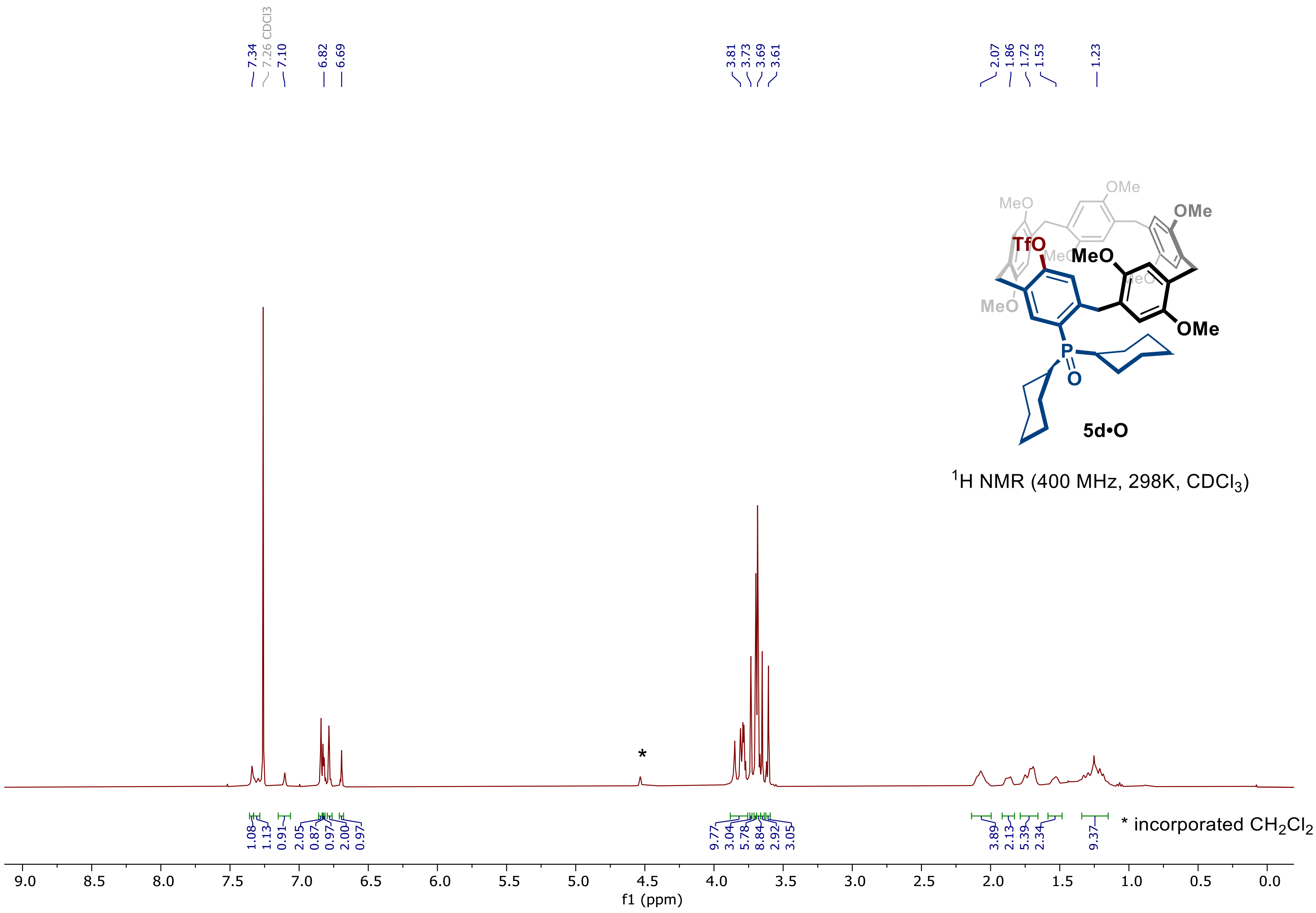
7.34
7.26 CDCl3
7.10
6.82
6.69
3.81
3.73
3.69
3.61
2.07
1.86
1.72
1.53
1.23
MeO
OMe
OMe
TfO
MeO
MeO
OMe
P
O
5d•O
^{1}H NMR (400 MHz, 298K, $CDCl_3$)
*
1.08
1.13
0.91
2.05
0.87
0.97
2.00
0.97
9.77
3.04
5.78
8.84
2.92
3.05
3.89
2.13
5.39
2.34
9.37
* incorporated CH_2Cl_2
9.0
8.5
8.0
7.5
7.0
6.5
6.0
5.5
5.0
4.5
4.0
3.5
3.0
2.5
2.0
1.5
1.0
0.5
0.0
f1 (ppm)

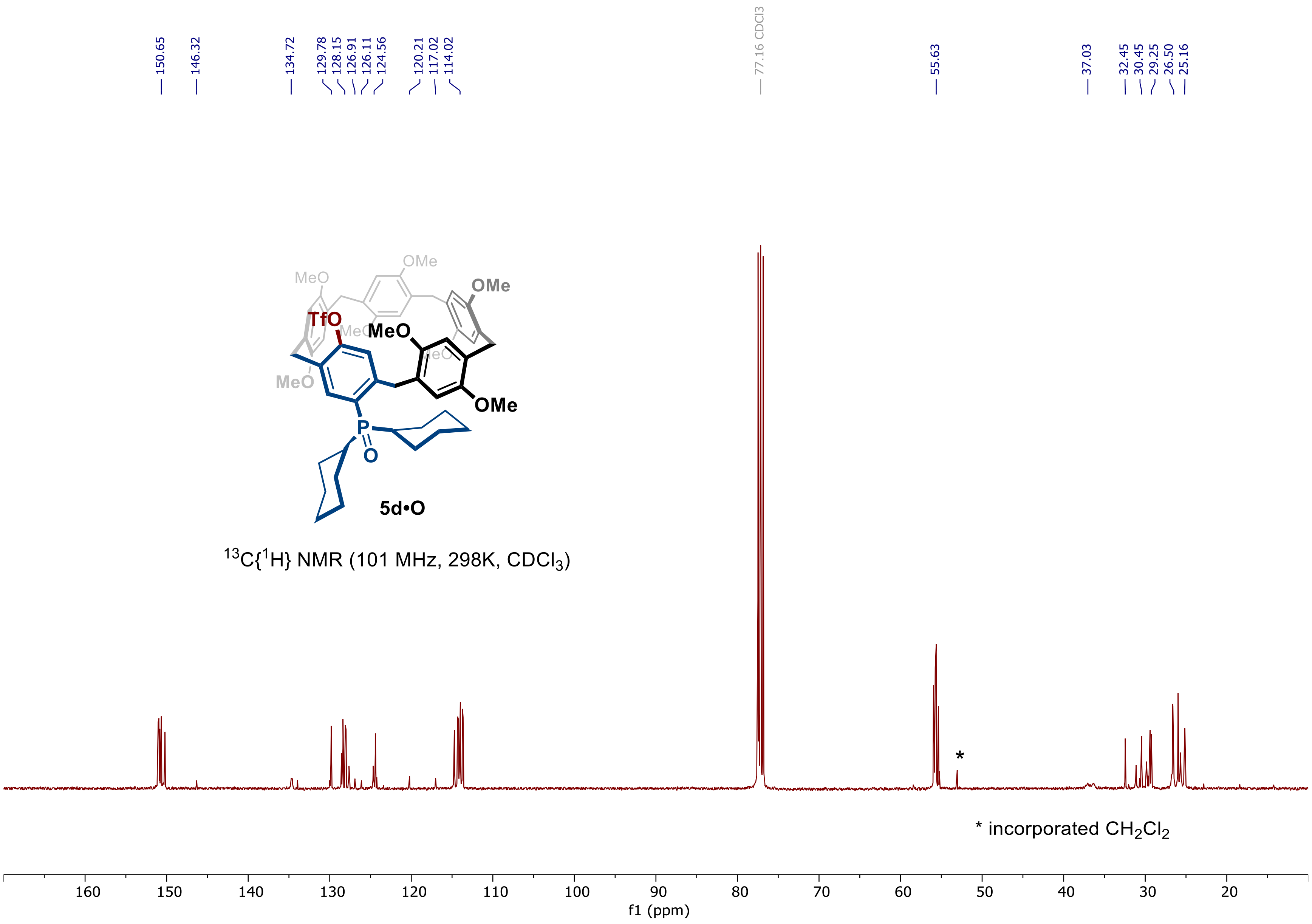
150.65
146.32
134.72
129.78
128.15
126.91
126.11
124.56
120.21
117.02
114.02
77.16 CDCl3
55.63
37.03
32.45
30.45
29.25
26.50
25.16
OMe
MeO
OMe
TfO
MeO
MeO
OMe
P
O
5d•O
$^{13}C\{^1H\}$ NMR (101 MHz, 298K, $CDCl_3$)
*
* incorporated CH_2Cl_2
160
150
140
130
120
110
100
90
80
70
60
50
40
30
20
f1 (ppm)

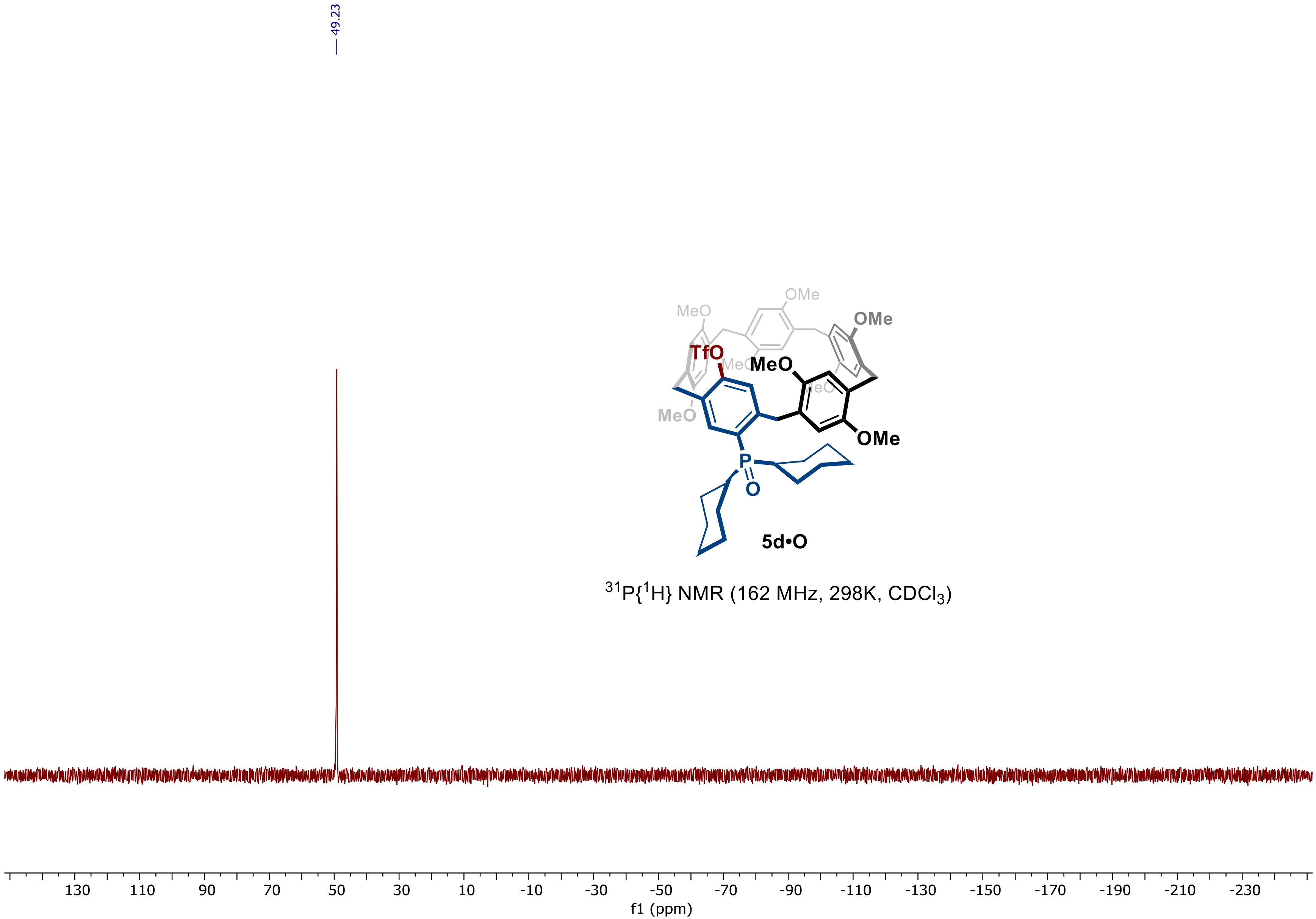

5d•O

$^{31}P\{^{1}H\}$ NMR (162 MHz, 298K, $CDCl_3$)

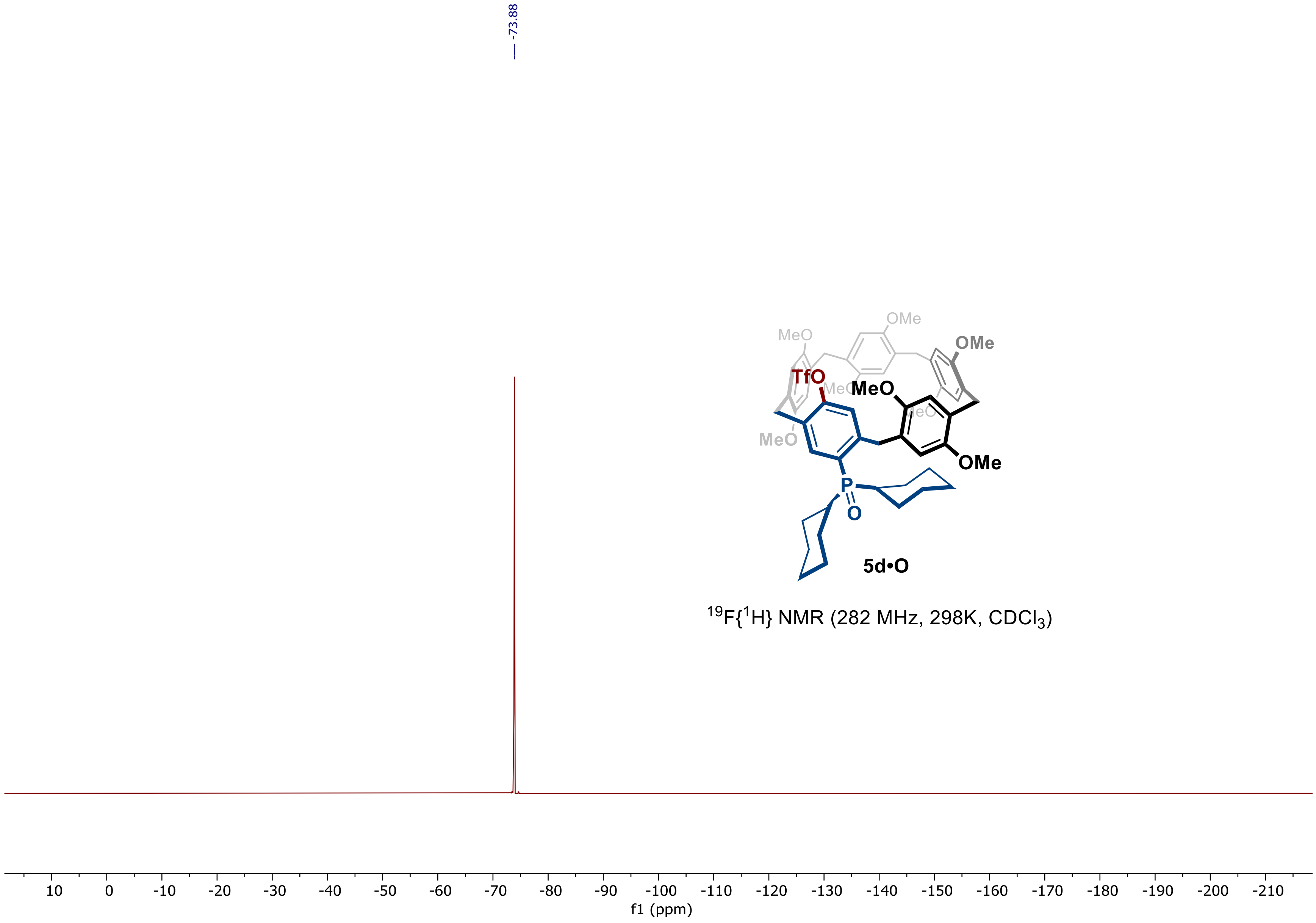

5d•O

$^{19}F\{^{1}H\}$ NMR (282 MHz, 298K, $CDCl_3$)

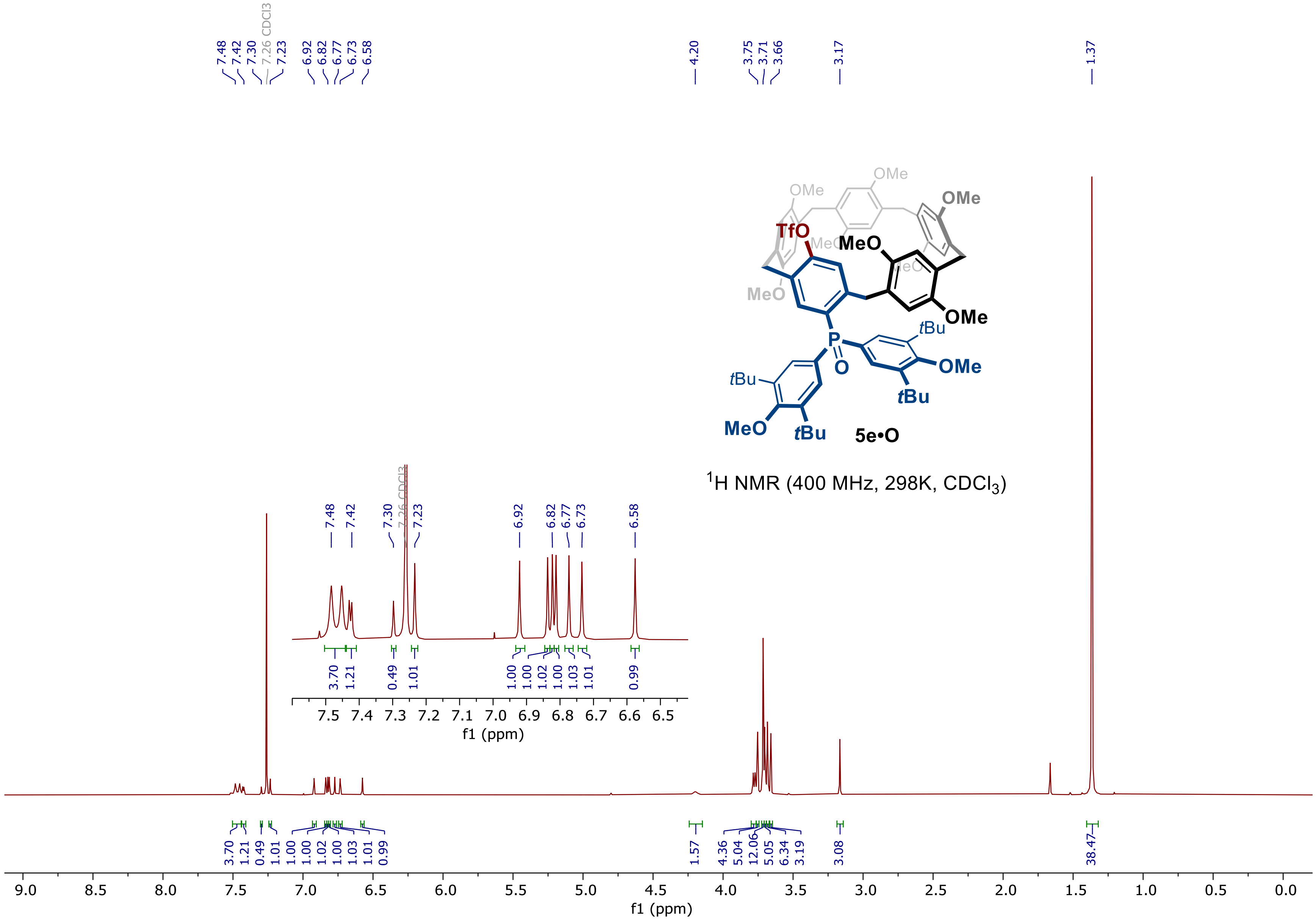

7.48
7.42
7.30
7.26 CDCl3
7.23
6.92
6.82
6.77
6.73
6.58
4.20
3.75
3.71
3.66
3.17
1.37
OMe
OMe
OMe
TfO
MeO
MeO
OMe
P
O
tBu
OMe
tBu
tBu
MeO
tBu
5e•O
^{1}H NMR (400 MHz, 298K, $CDCl_3$)
3.70
1.21
0.49
1.01
1.00
1.00
1.02
1.00
1.03
1.01
0.99
1.57
4.36
5.04
12.06
5.05
6.34
3.19
3.08
38.47
7.5 7.4 7.3 7.2 7.1 7.0 6.9 6.8 6.7 6.6 6.5
f1 (ppm)
9.0 8.5 8.0 7.5 7.0 6.5 6.0 5.5 5.0 4.5 4.0 3.5 3.0 2.5 2.0 1.5 1.0 0.5 0.0
f1 (ppm)

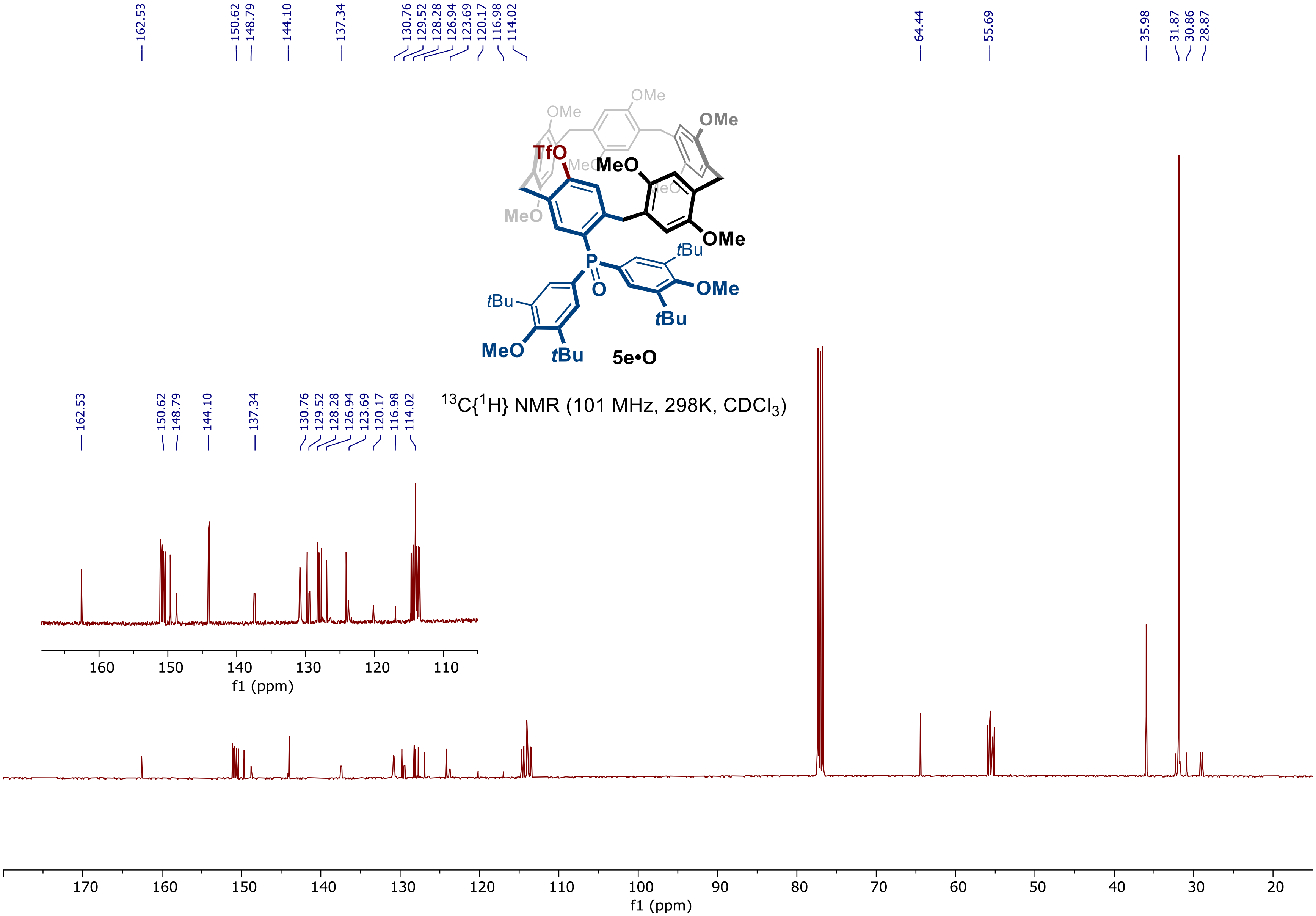

$^{13}C\{^{1}H\}$ NMR (101 MHz, 298K, $CDCl_3$)

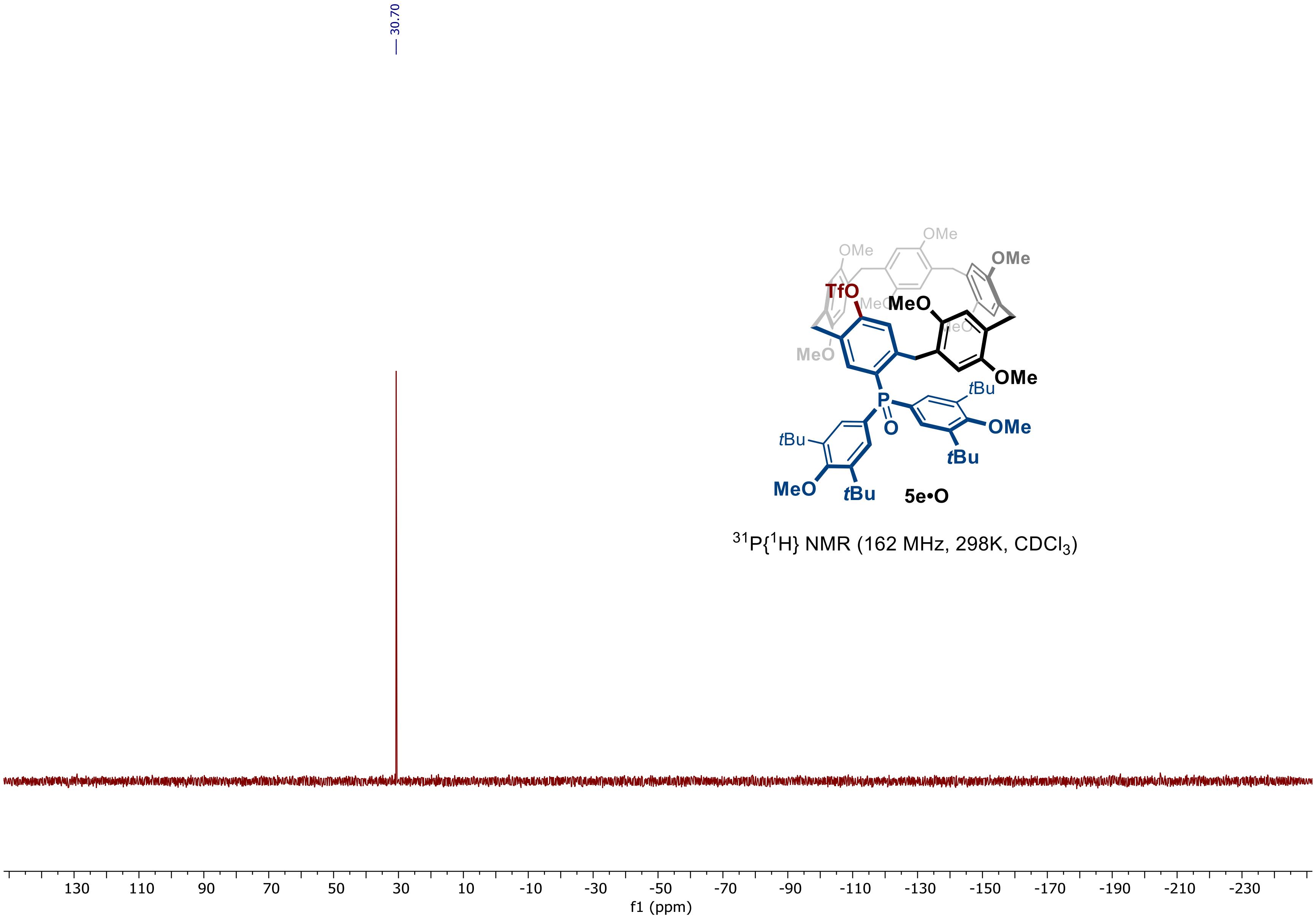

5e•O

$^{31}P\{^{1}H\}$ NMR (162 MHz, 298K, $CDCl_3$)

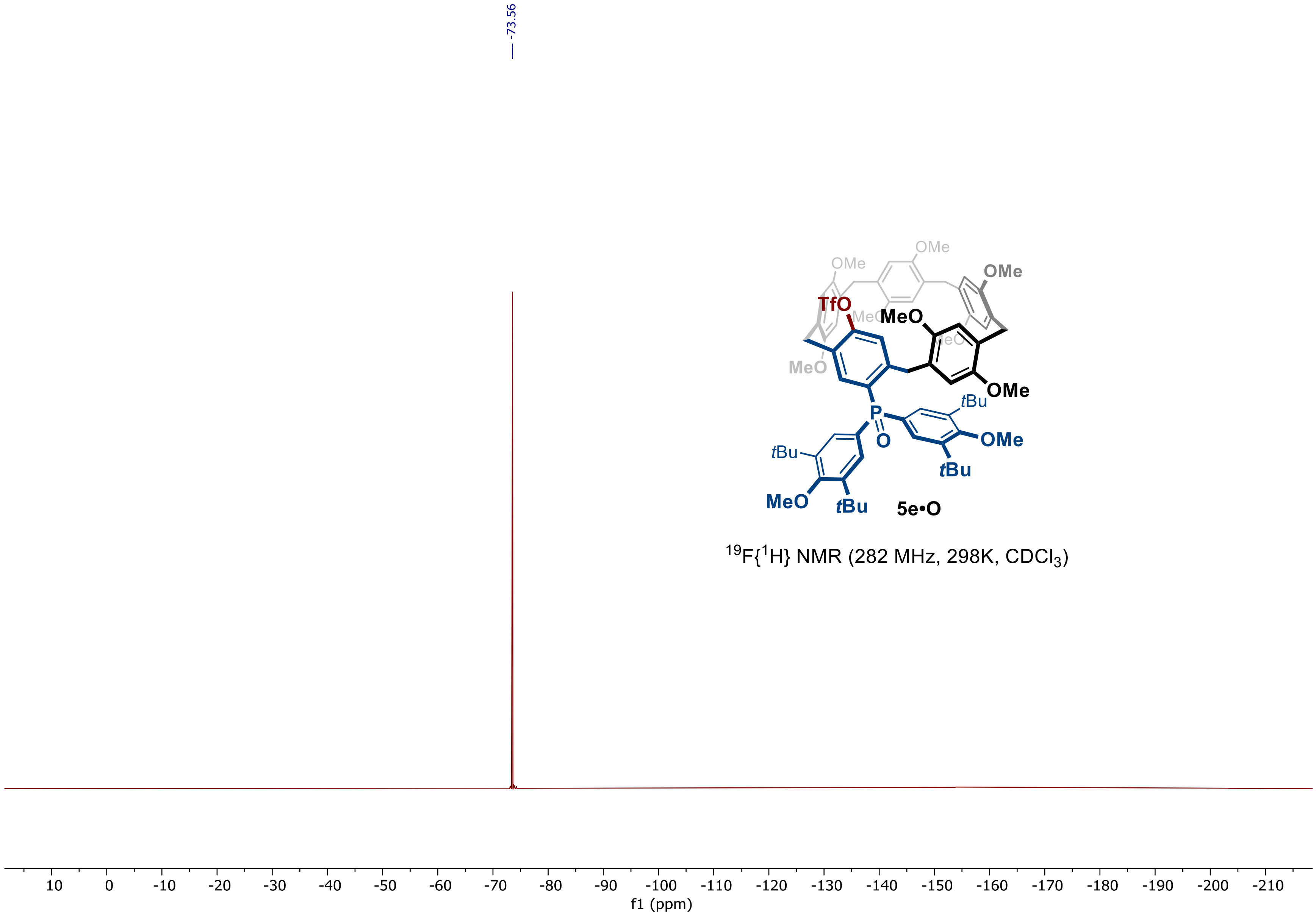

$^{19}F\{^{1}H\}$ NMR (282 MHz, 298K, $CDCl_3$)

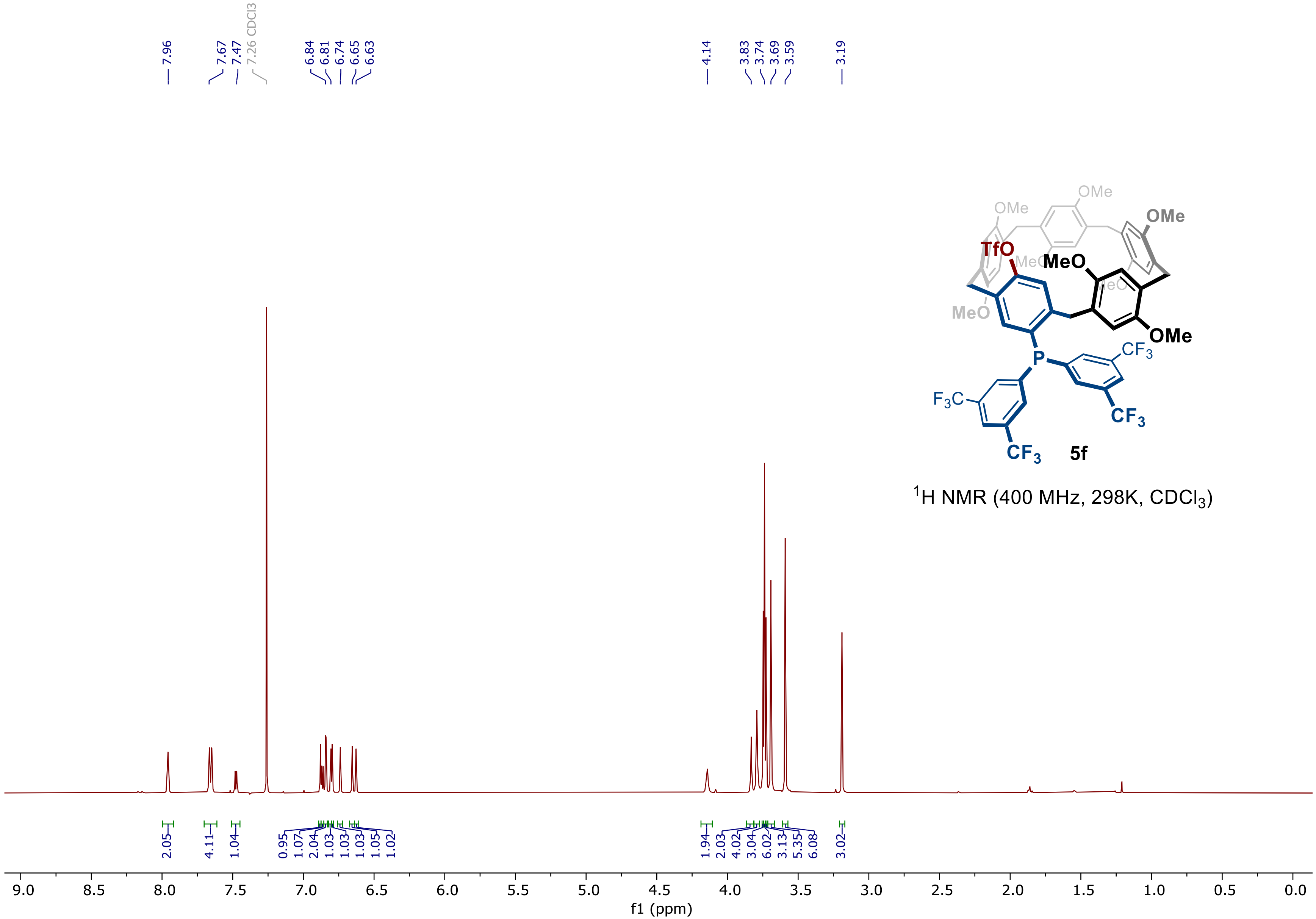

7.96
7.67
7.47
7.26 CDCl3
6.84
6.81
6.74
6.65
6.63
4.14
3.83
3.74
3.69
3.59
3.19
OMe
OMe
OMe
TfO
MeO
MeO
OMe
P
CF3
F3C
CF3
CF3
5f
1H NMR (400 MHz, 298K, CDCl3)
2.05
4.11
1.04
0.95
1.07
2.04
1.03
1.03
1.03
1.05
1.02
1.94
2.03
4.02
3.04
6.02
3.13
5.35
6.08
3.02
9.0
8.5
8.0
7.5
7.0
6.5
6.0
5.5
5.0
4.5
4.0
3.5
3.0
2.5
2.0
1.5
1.0
0.5
0.0
f1 (ppm)

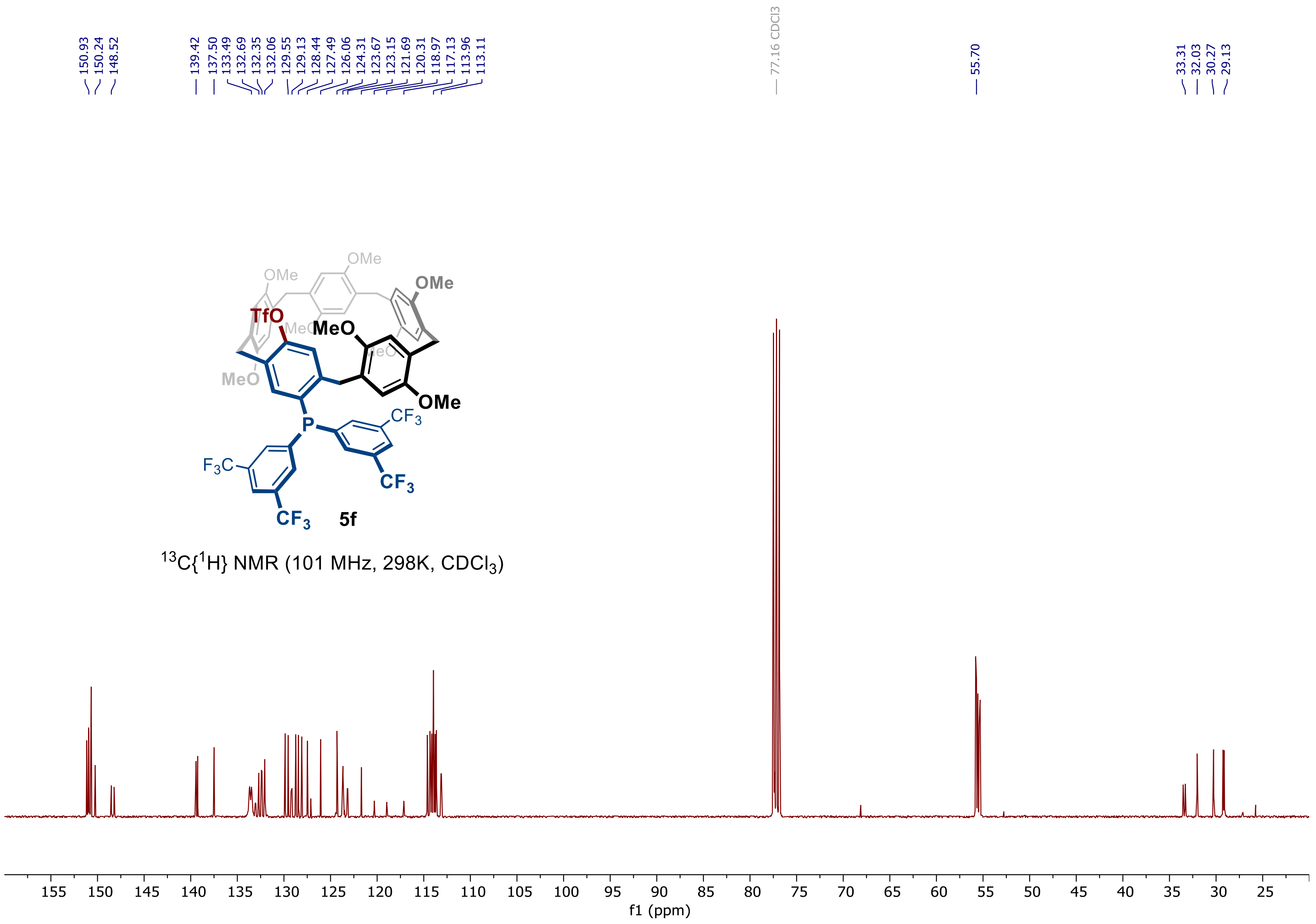

$^{13}C\{^{1}H\}$ NMR (101 MHz, 298K, $CDCl_3$)

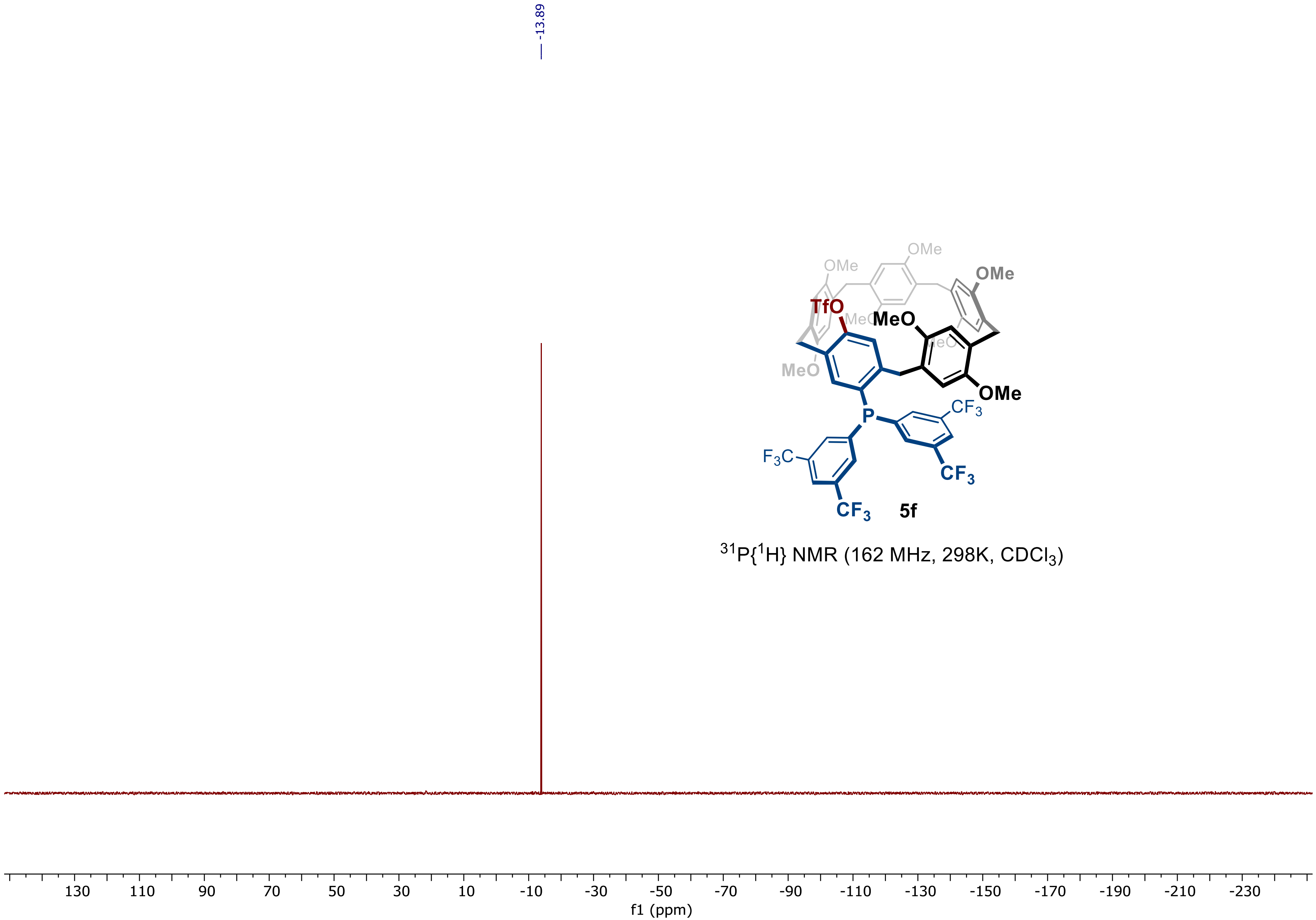

5f

$^{31}P\{^{1}H\}$ NMR (162 MHz, 298K, $CDCl_3$)

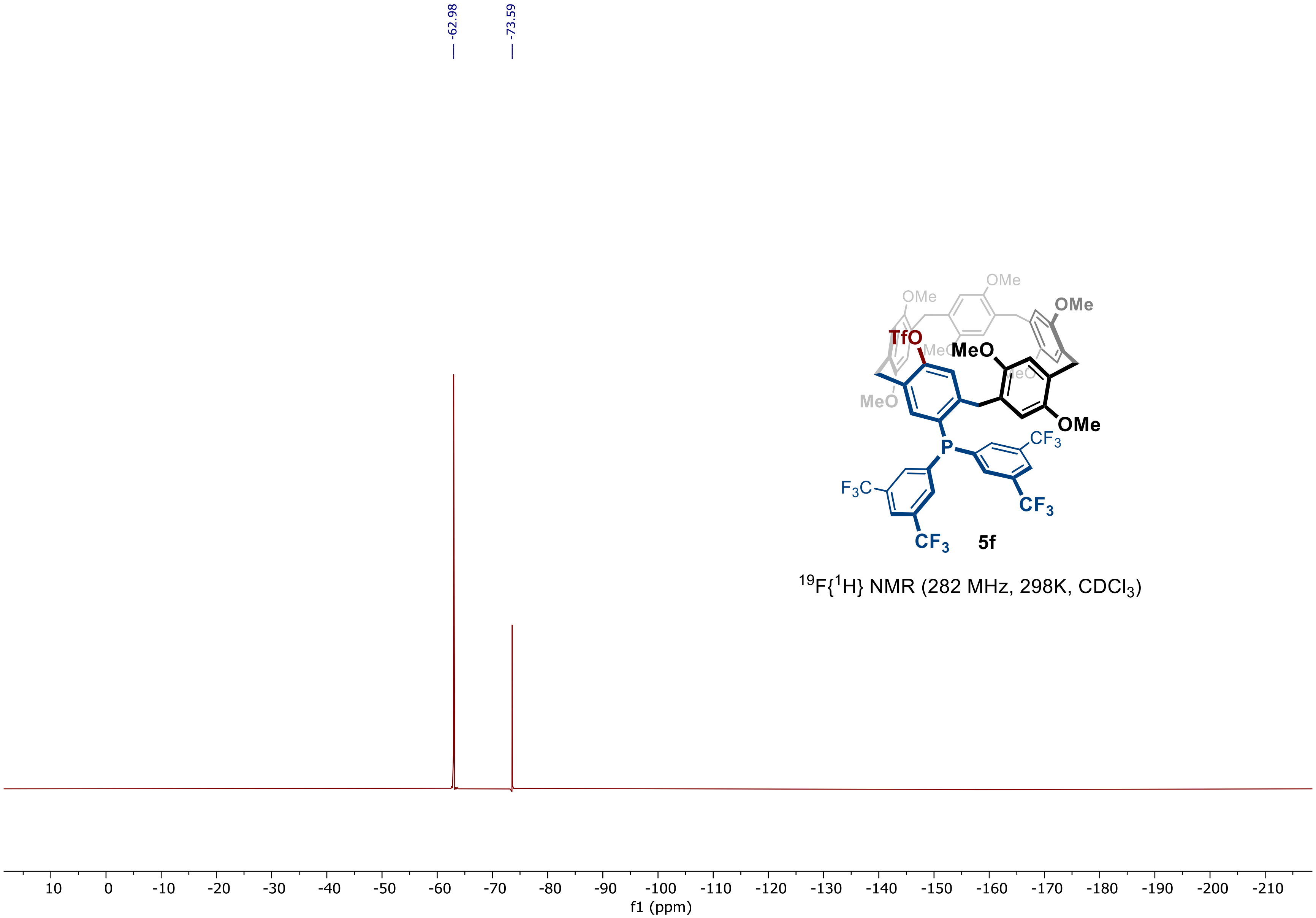

5f

$^{19}F\{^1H\}$ NMR (282 MHz, 298K, $CDCl_3$)

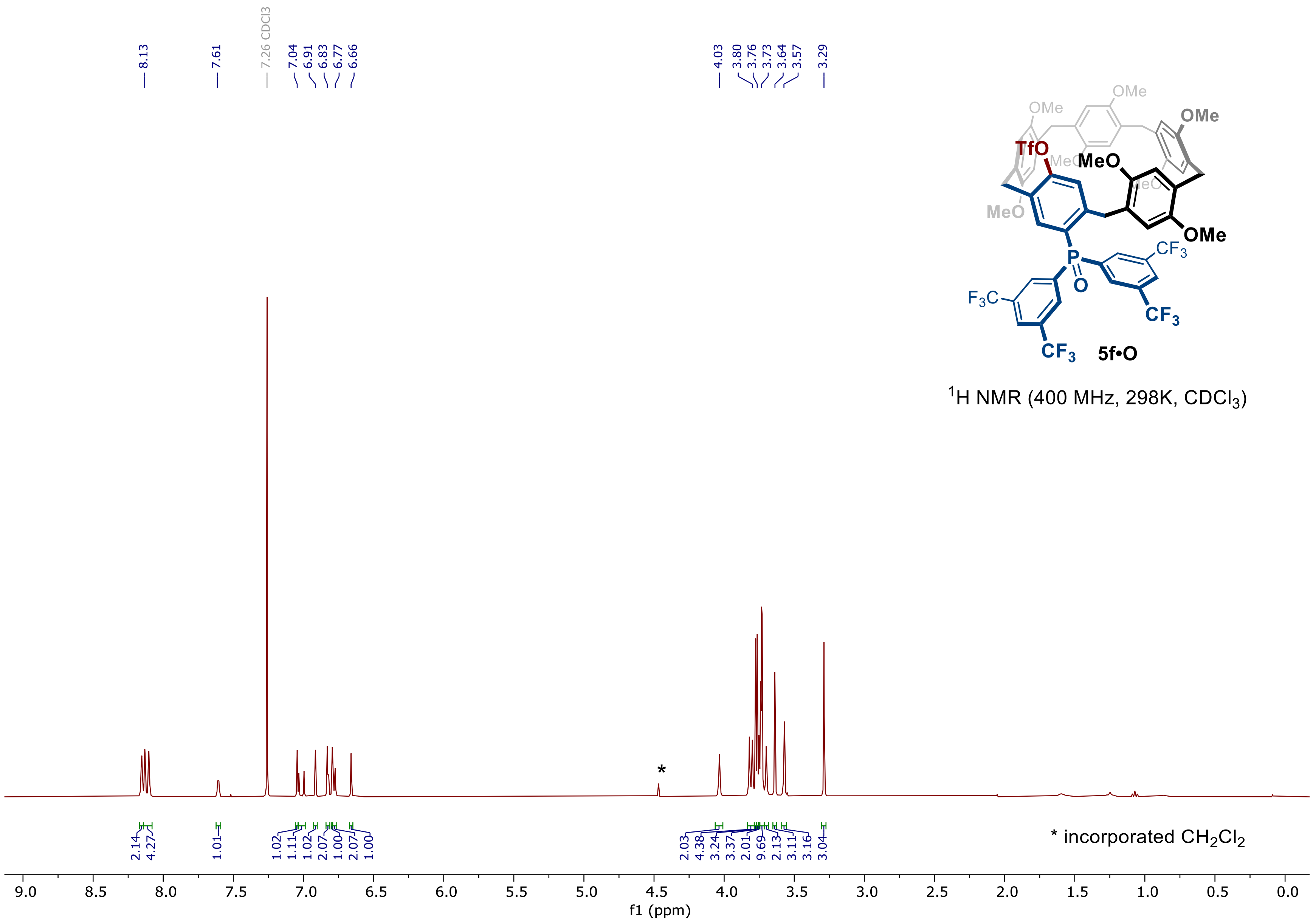
8.13
7.61
7.26 CDCl3
7.04
6.91
6.83
6.77
6.66
4.03
3.80
3.76
3.73
3.64
3.57
3.29
OMe
OMe
OMe
TfO
MeO
MeO
OMe
CF3
P
O
F3C
CF3
CF3
5f•O
1H NMR (400 MHz, 298K, CDCl3)
*
2.14
4.27
1.01
1.02
1.11
1.02
2.07
1.00
2.07
1.00
2.03
4.38
3.24
3.37
2.01
9.69
2.13
3.11
3.16
3.04
* incorporated CH2Cl2
9.0
8.5
8.0
7.5
7.0
6.5
6.0
5.5
5.0
4.5
4.0
3.5
3.0
2.5
2.0
1.5
1.0
0.5
0.0
f1 (ppm)

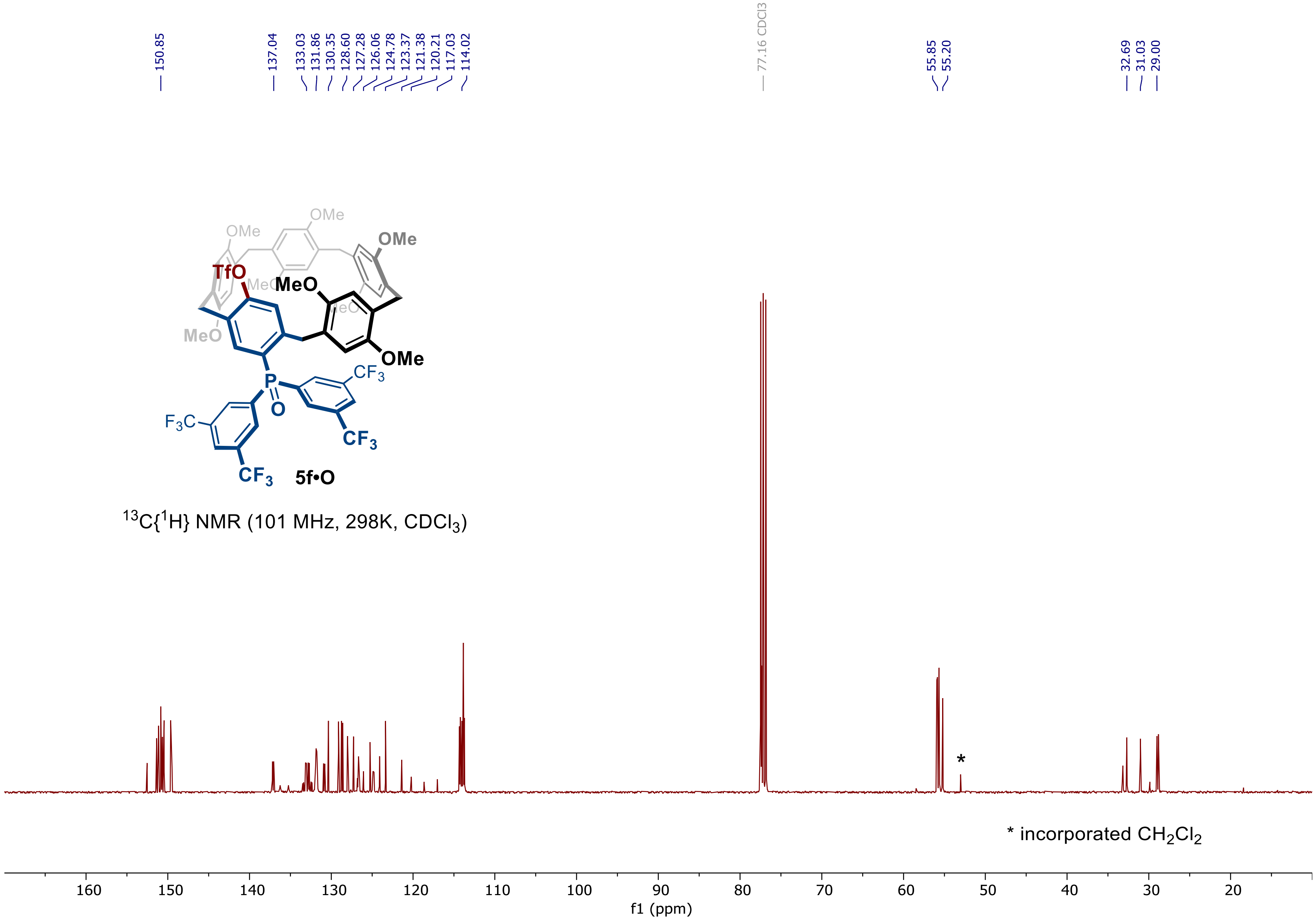

$^{13}C\{^{1}H\}$ NMR (101 MHz, 298K, $CDCl_3$)

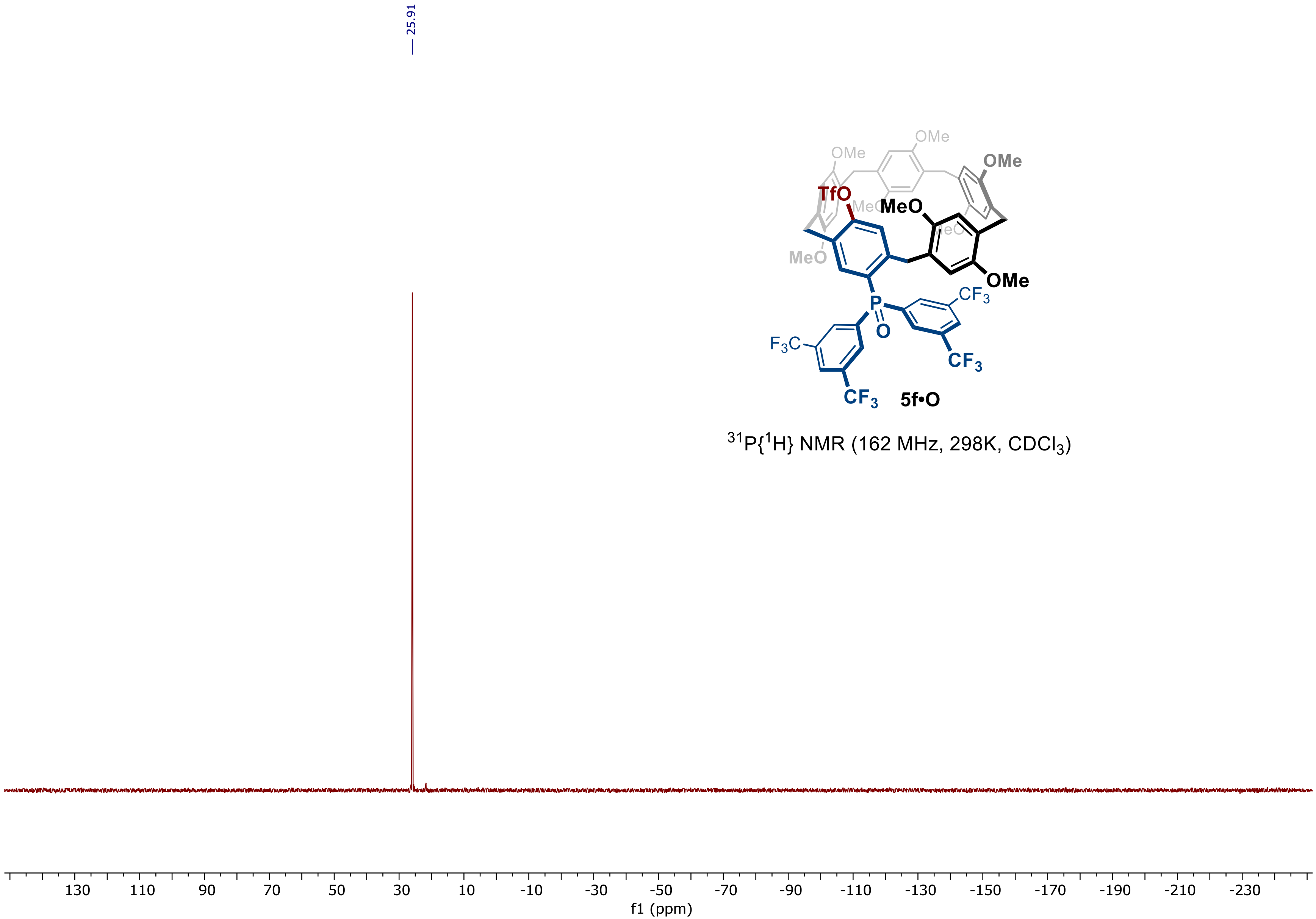

5f•O

$^{31}P\{^{1}H\}$ NMR (162 MHz, 298K, $CDCl_3$)

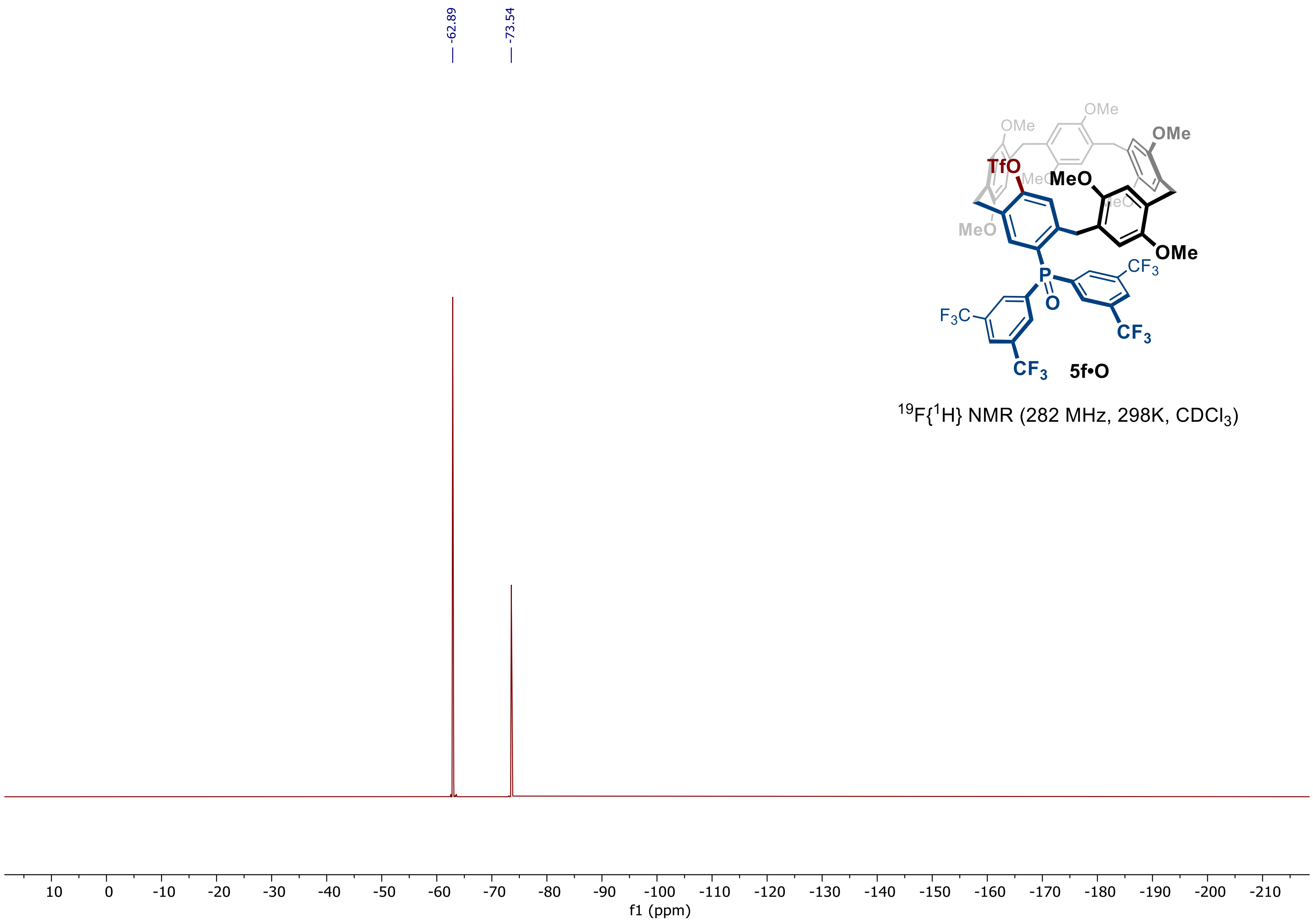

5f•O

$^{19}F\{^1H\}$ NMR (282 MHz, 298K, $CDCl_3$)

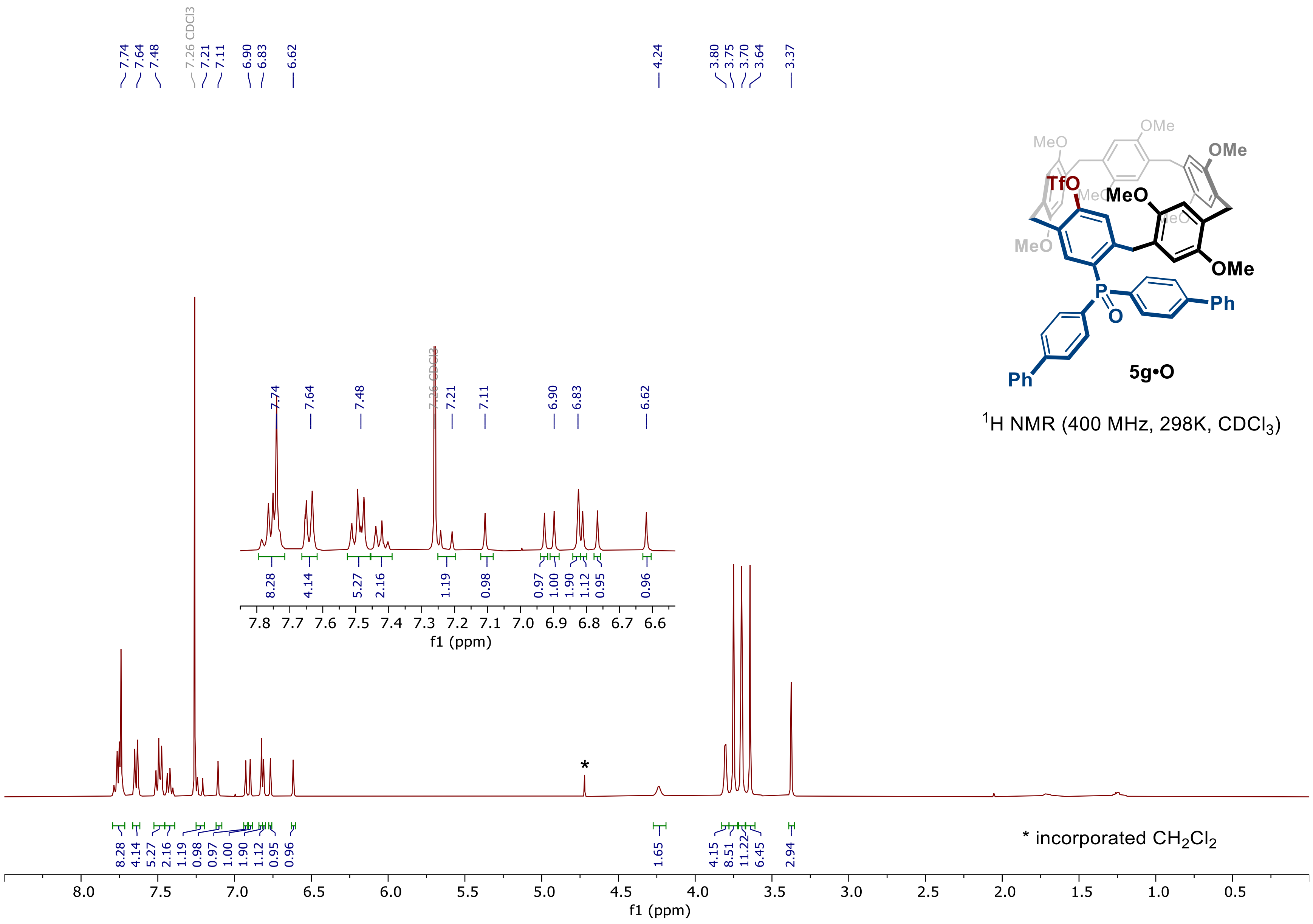

^{1}H NMR (400 MHz, 298K, $CDCl_3$)

* incorporated CH_2Cl_2

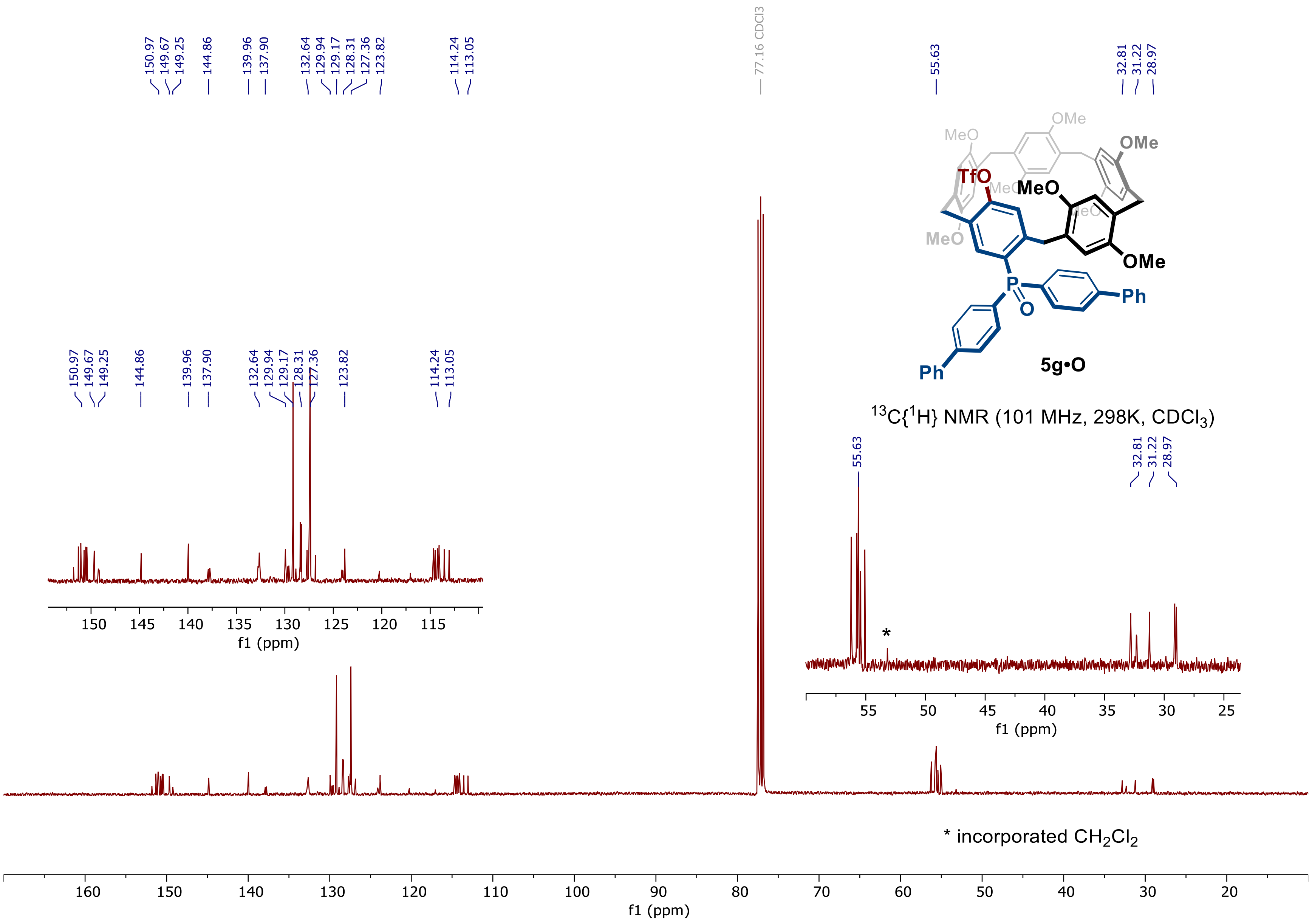
150.97
149.67
149.25
144.86
139.96
137.90
132.64
129.94
129.17
128.31
127.36
123.82
114.24
113.05
77.16 CDCl3
55.63
32.81
31.22
28.97
MeO
OMe
TfO
MeO
MeO
OMe
OMe
P
O
Ph
Ph
5g•O
13C{1H} NMR (101 MHz, 298K, CDCl3)
*
* incorporated CH2Cl2
f1 (ppm)

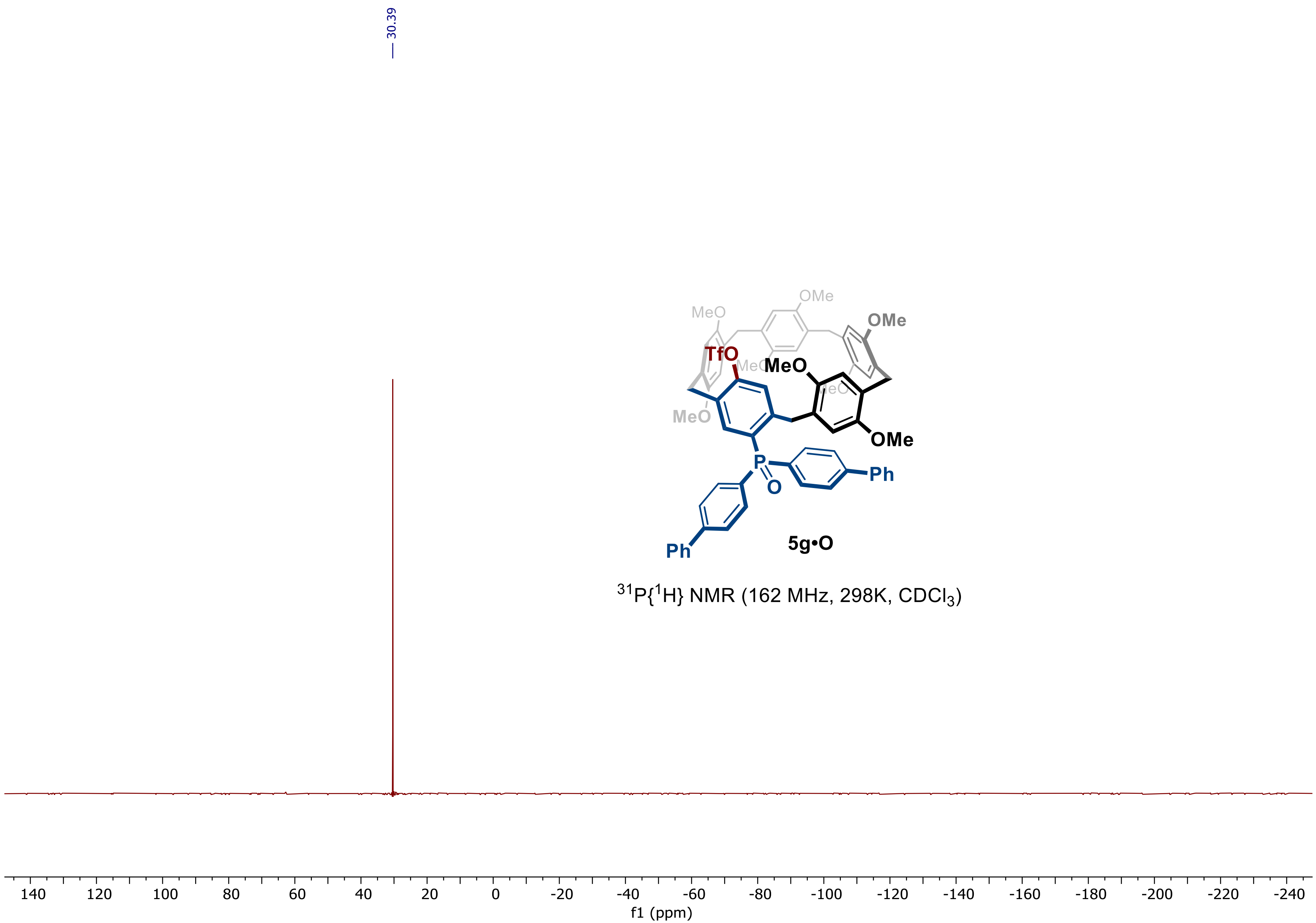

5g•O

$^{31}P\{^{1}H\}$ NMR (162 MHz, 298K, $CDCl_3$)

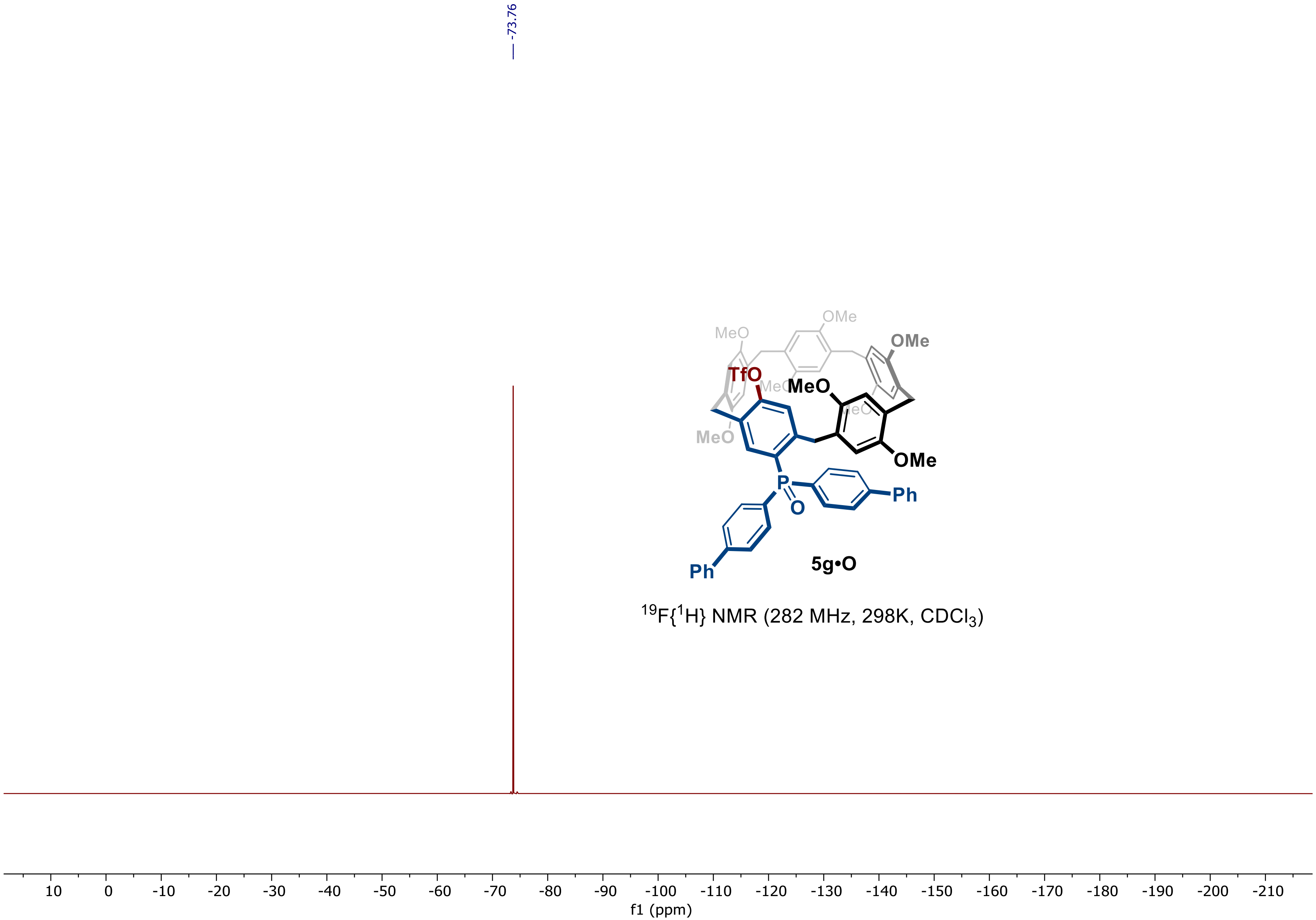

5g•O

$^{19}F\{^1H\}$ NMR (282 MHz, 298K, $CDCl_3$)

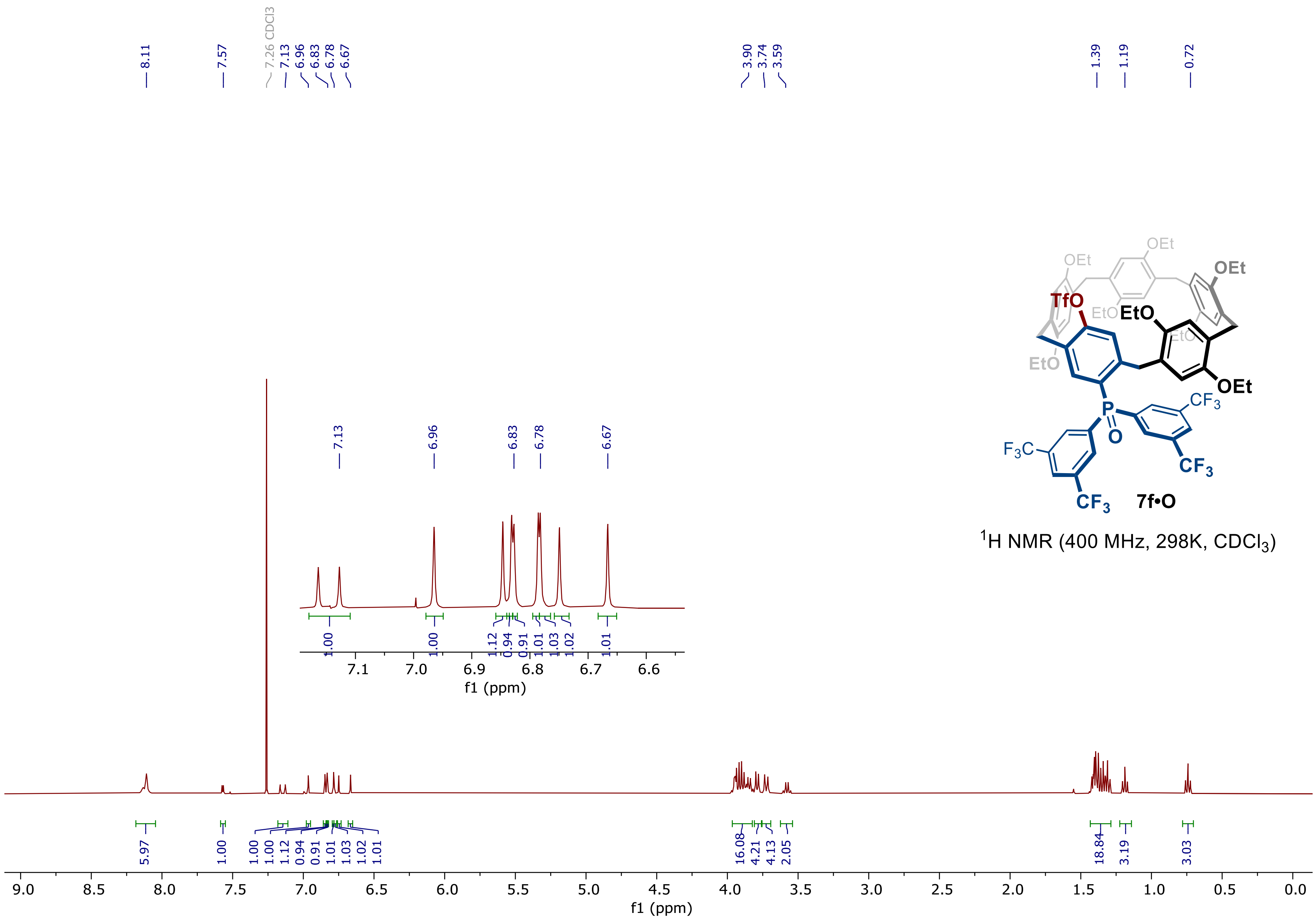

^{1}H NMR (400 MHz, 298K, $CDCl_3$)

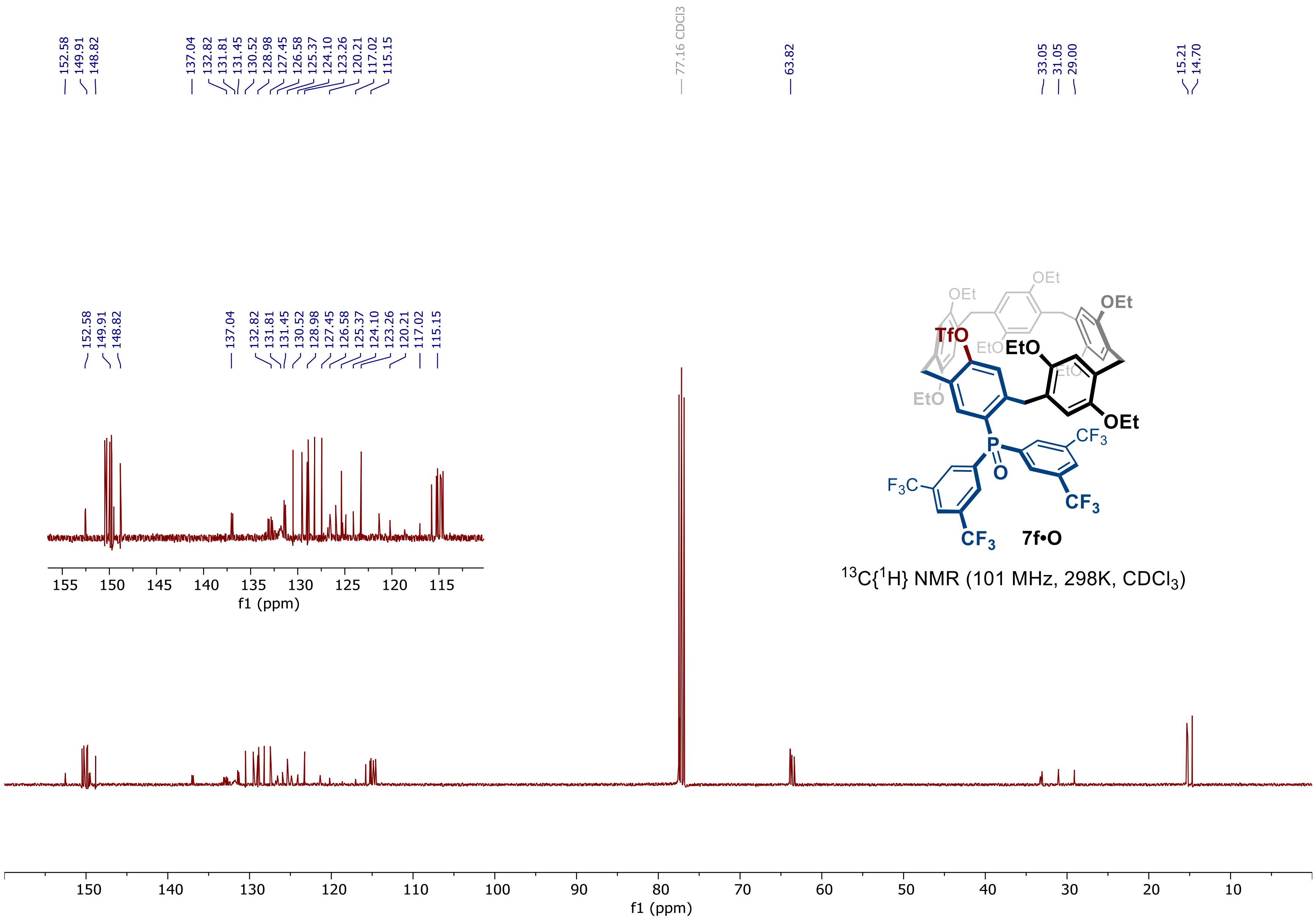

$^{13}C\{^1H\}$ NMR (101 MHz, 298K, $CDCl_3$)

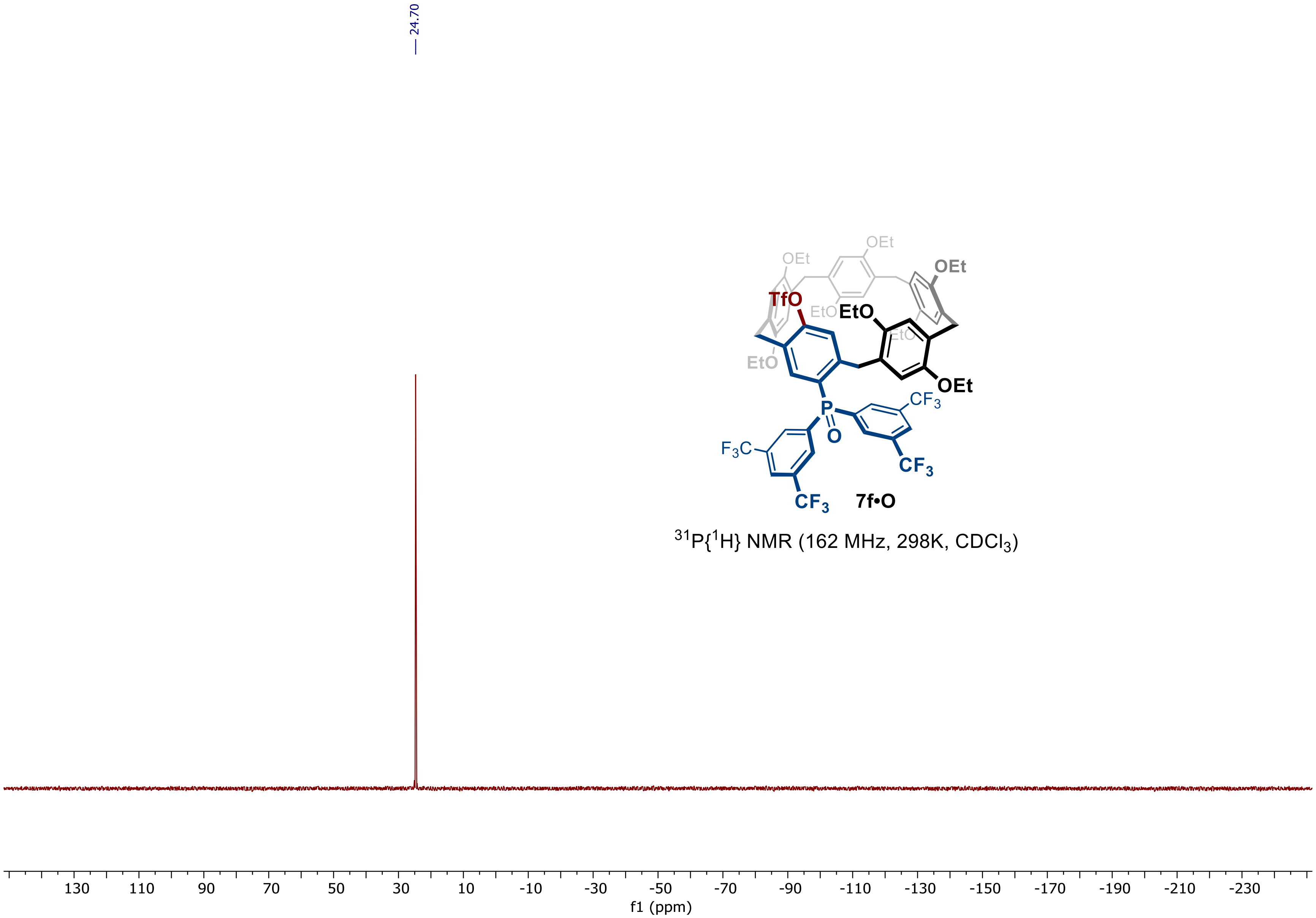

$^{31}P\{^1H\}$ NMR (162 MHz, 298K, $CDCl_3$)

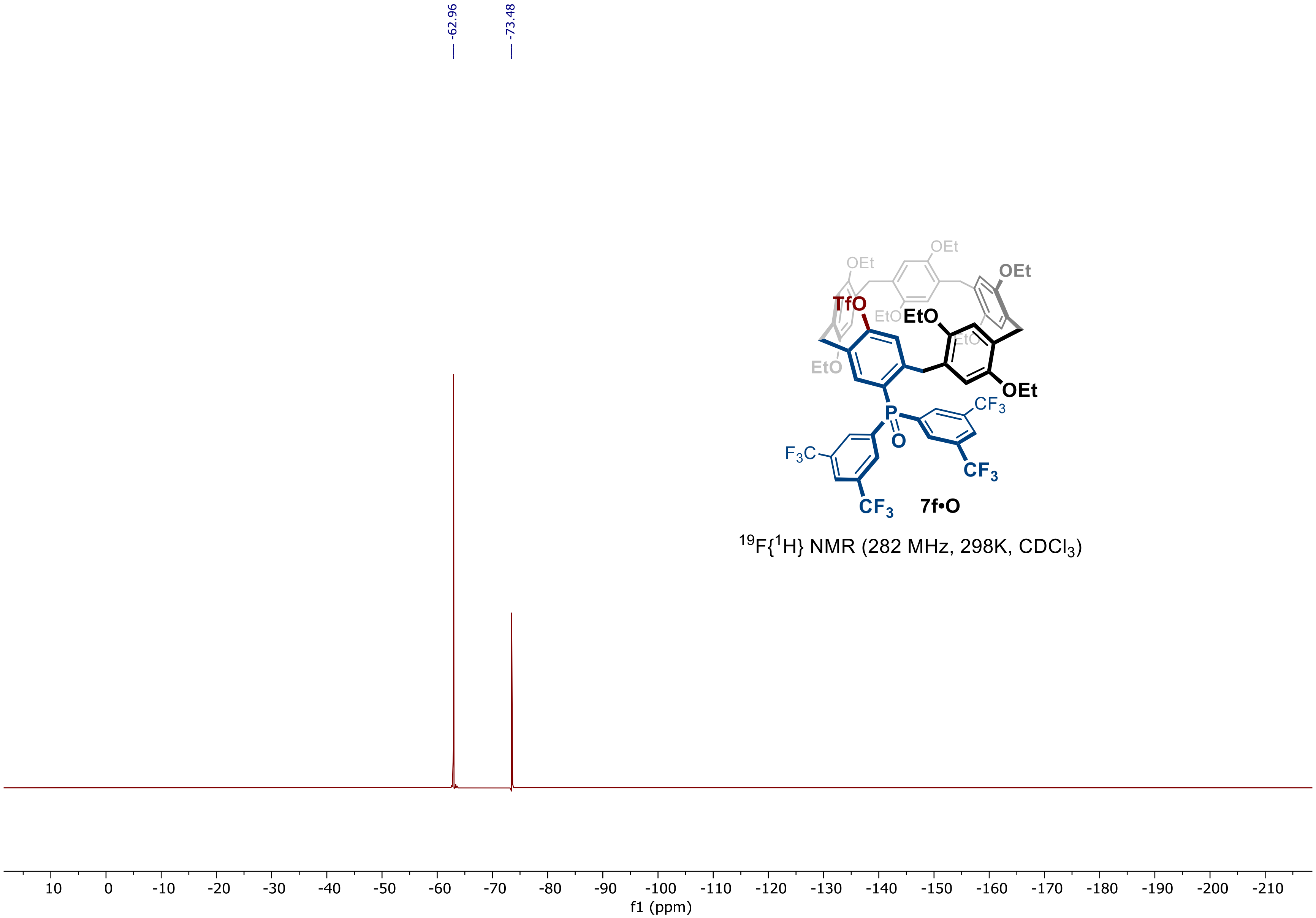
-62.96
-73.48
OEt
OEt
OEt
TfO
EtO
EtO
EtO
EtO
OEt
P
O
CF3
F3C
CF3
CF3
7f•O
19F{1H} NMR (282 MHz, 298K, CDCl3)
10 0 -10 -20 -30 -40 -50 -60 -70 -80 -90 -100 -110 -120 -130 -140 -150 -160 -170 -180 -190 -200 -210
f1 (ppm)

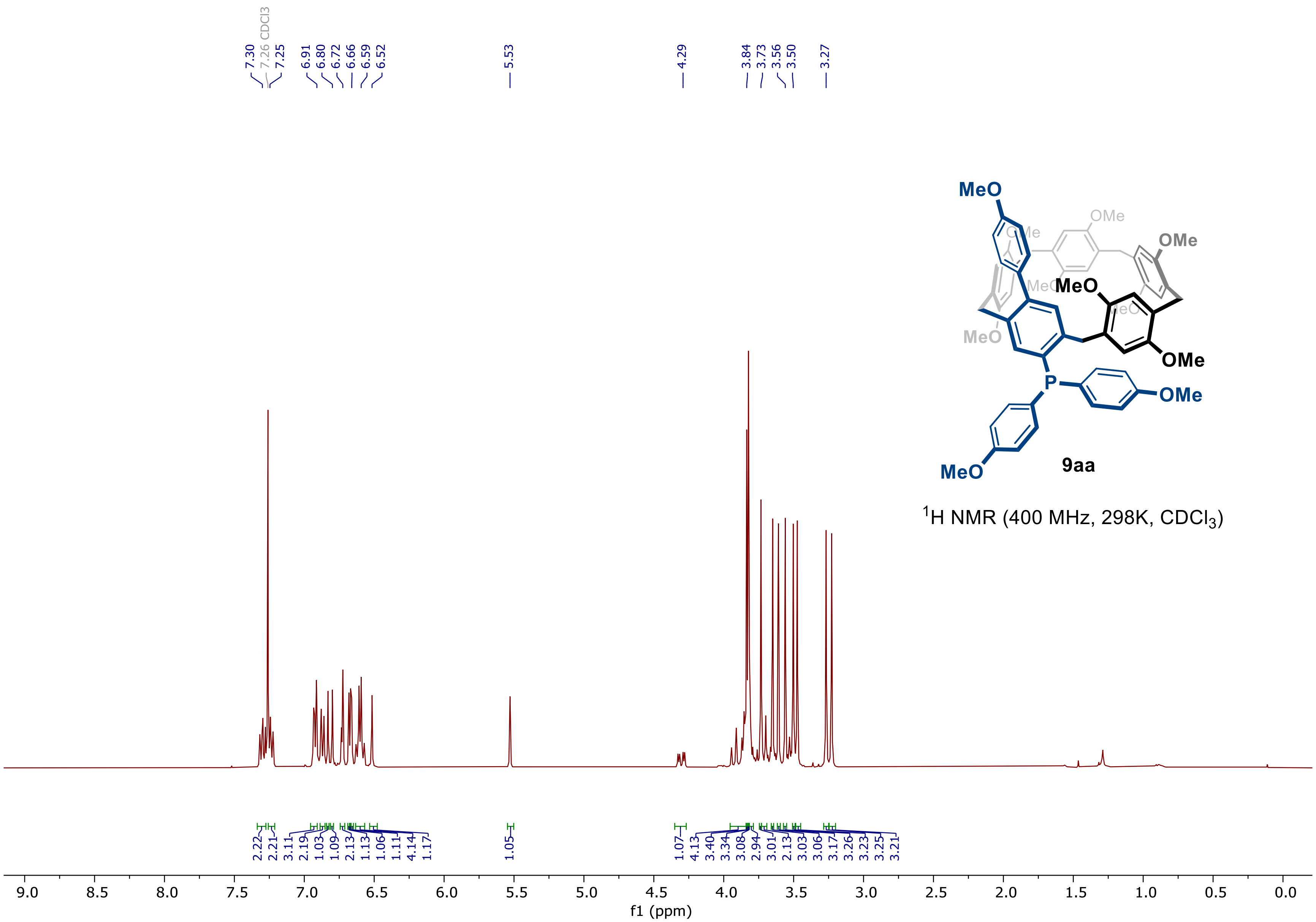

^{1}H NMR (400 MHz, 298K, $CDCl_3$)

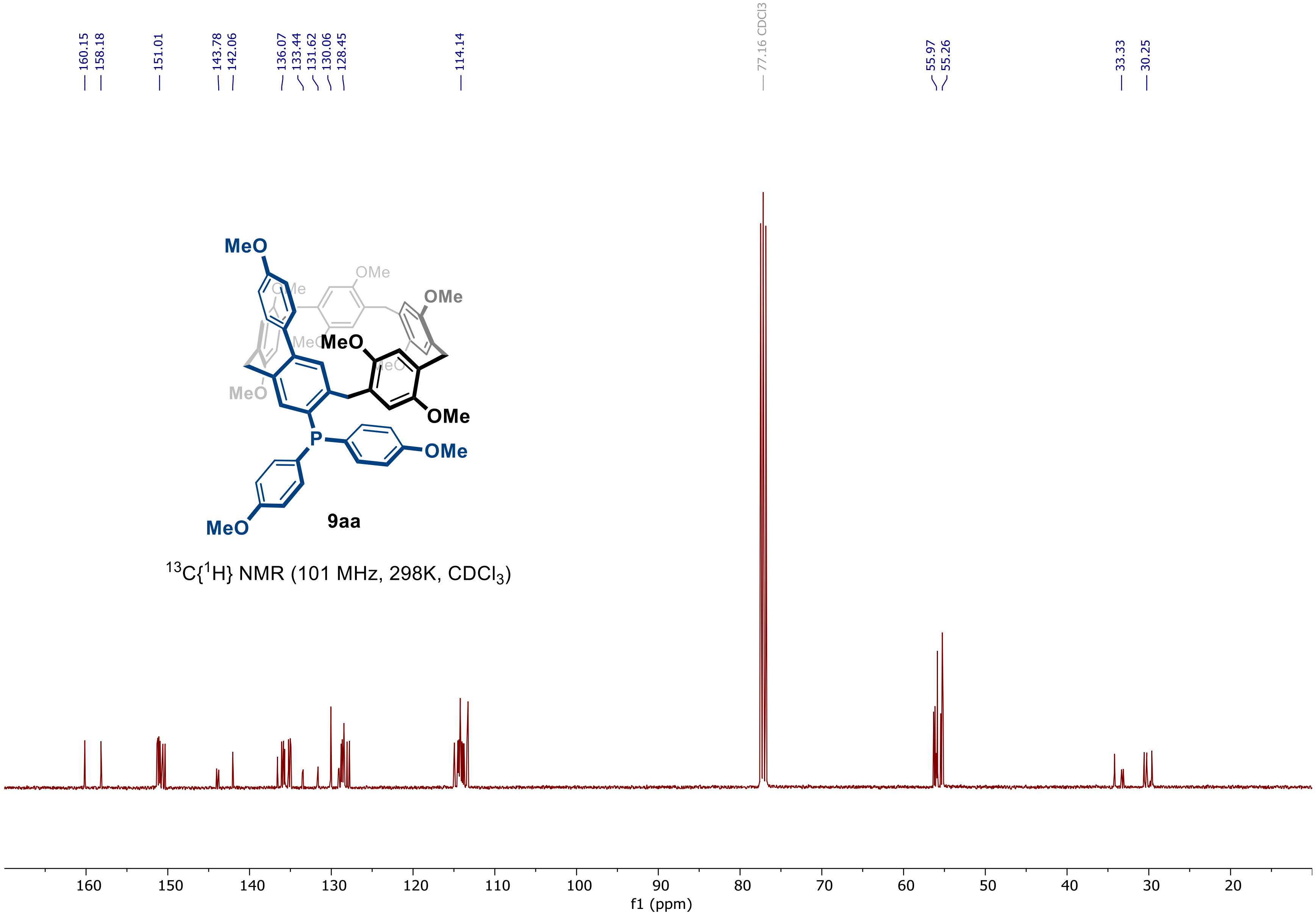

9aa

$^{13}C\{^{1}H\}$ NMR (101 MHz, 298K, $CDCl_3$)

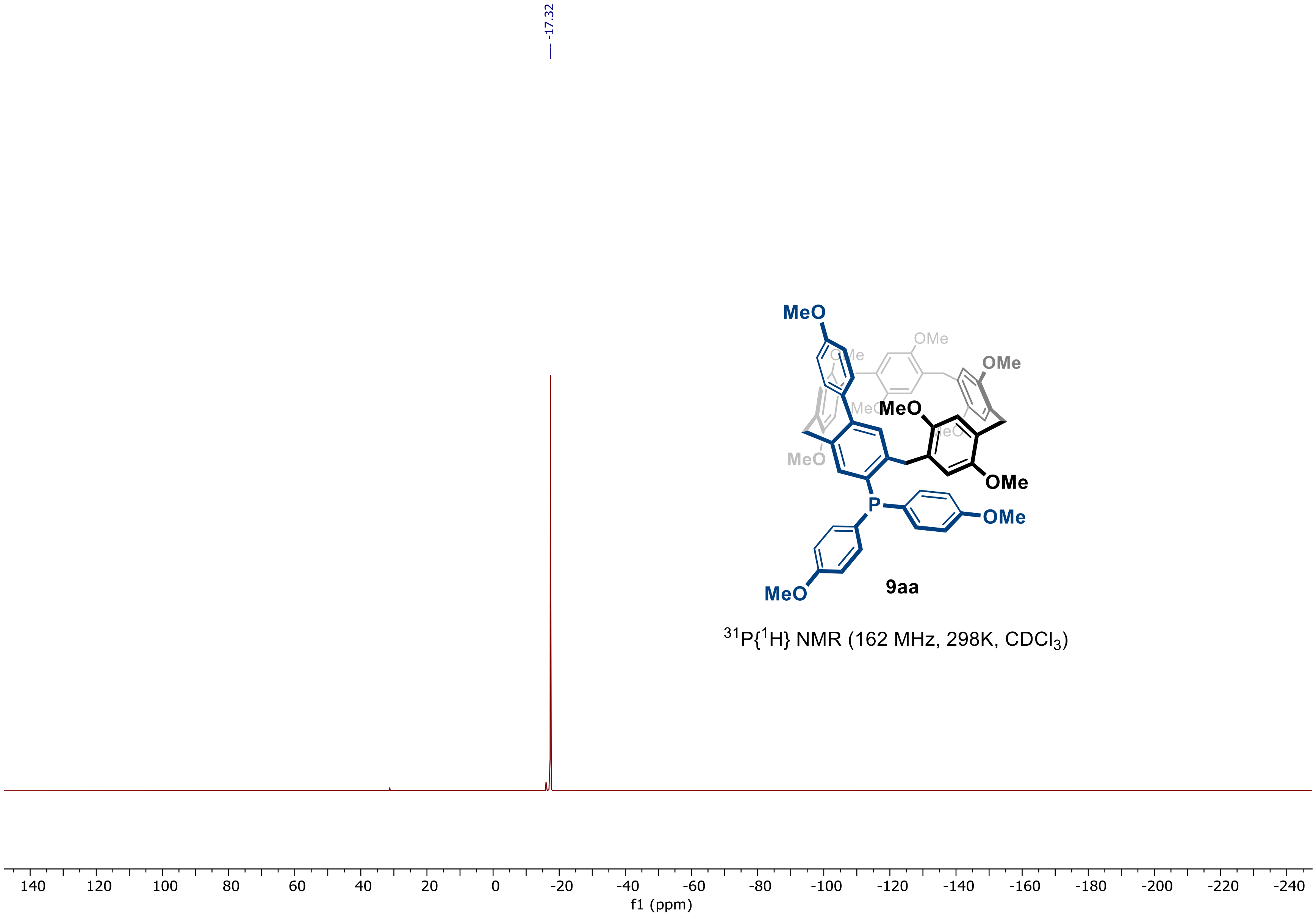

9aa

$^{31}P\{^{1}H\}$ NMR (162 MHz, 298K, $CDCl_3$)

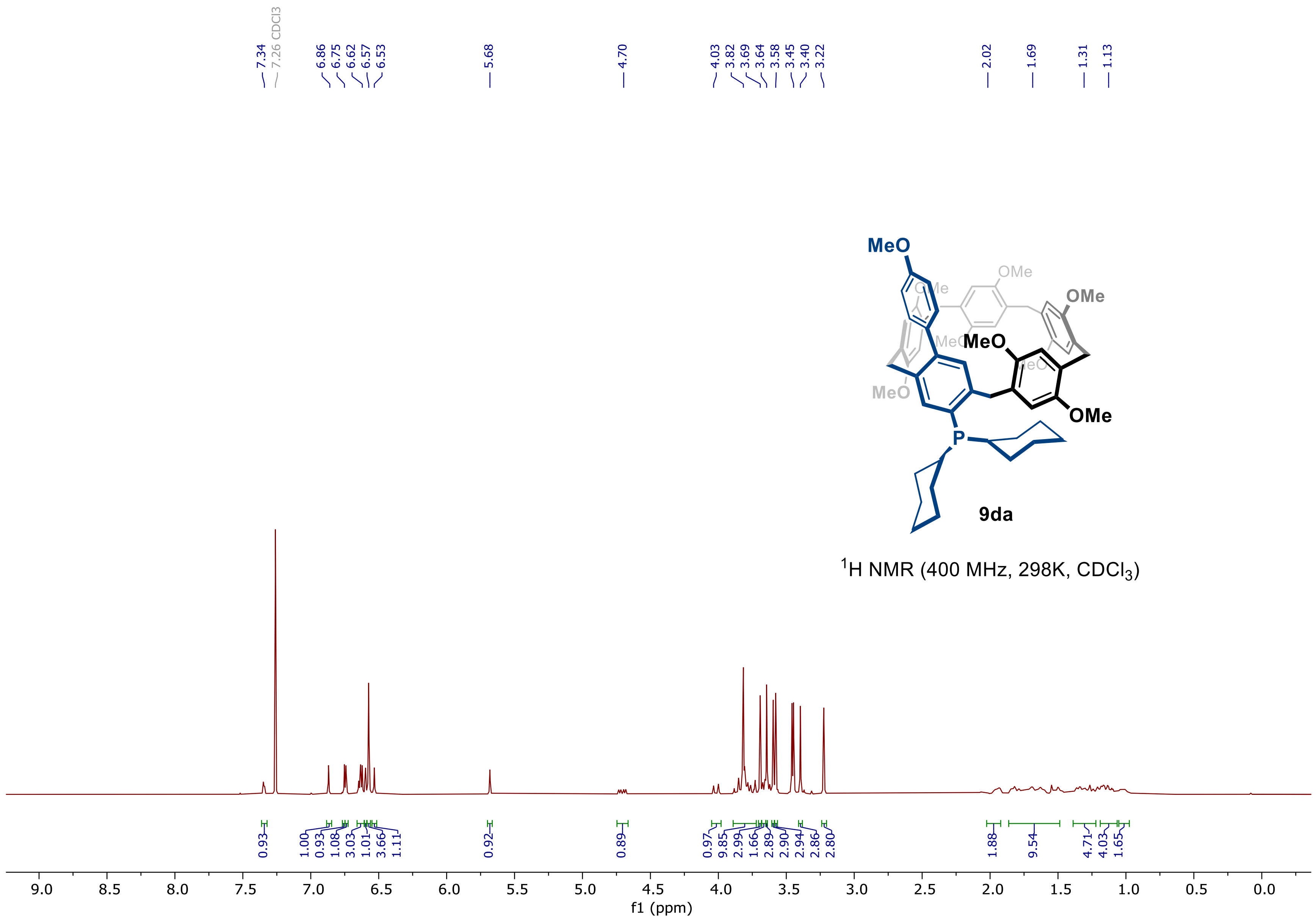

9da

^{1}H NMR (400 MHz, 298K, $CDCl_3$)

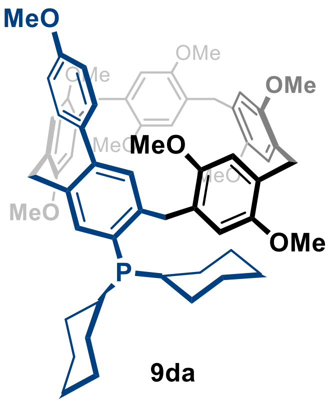

9da

$^{13}C\{^{1}H\}$ NMR (101 MHz, 298K, $CDCl_3$)

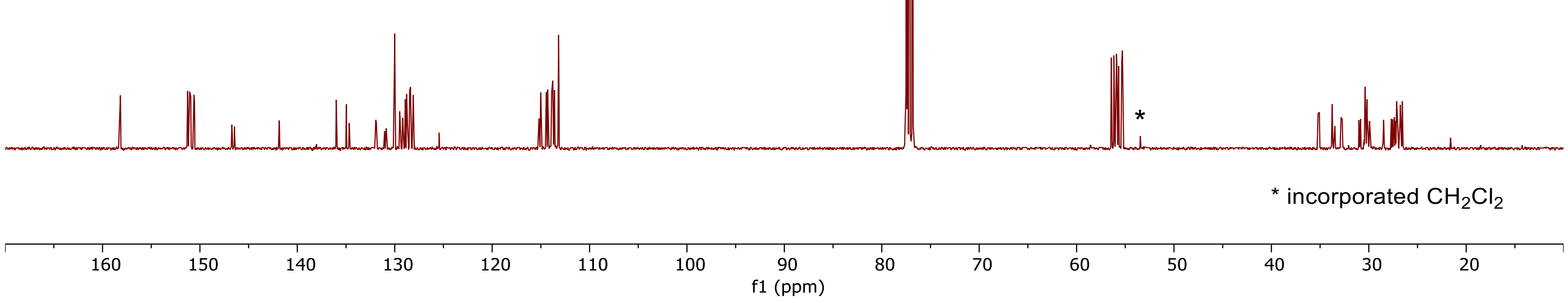

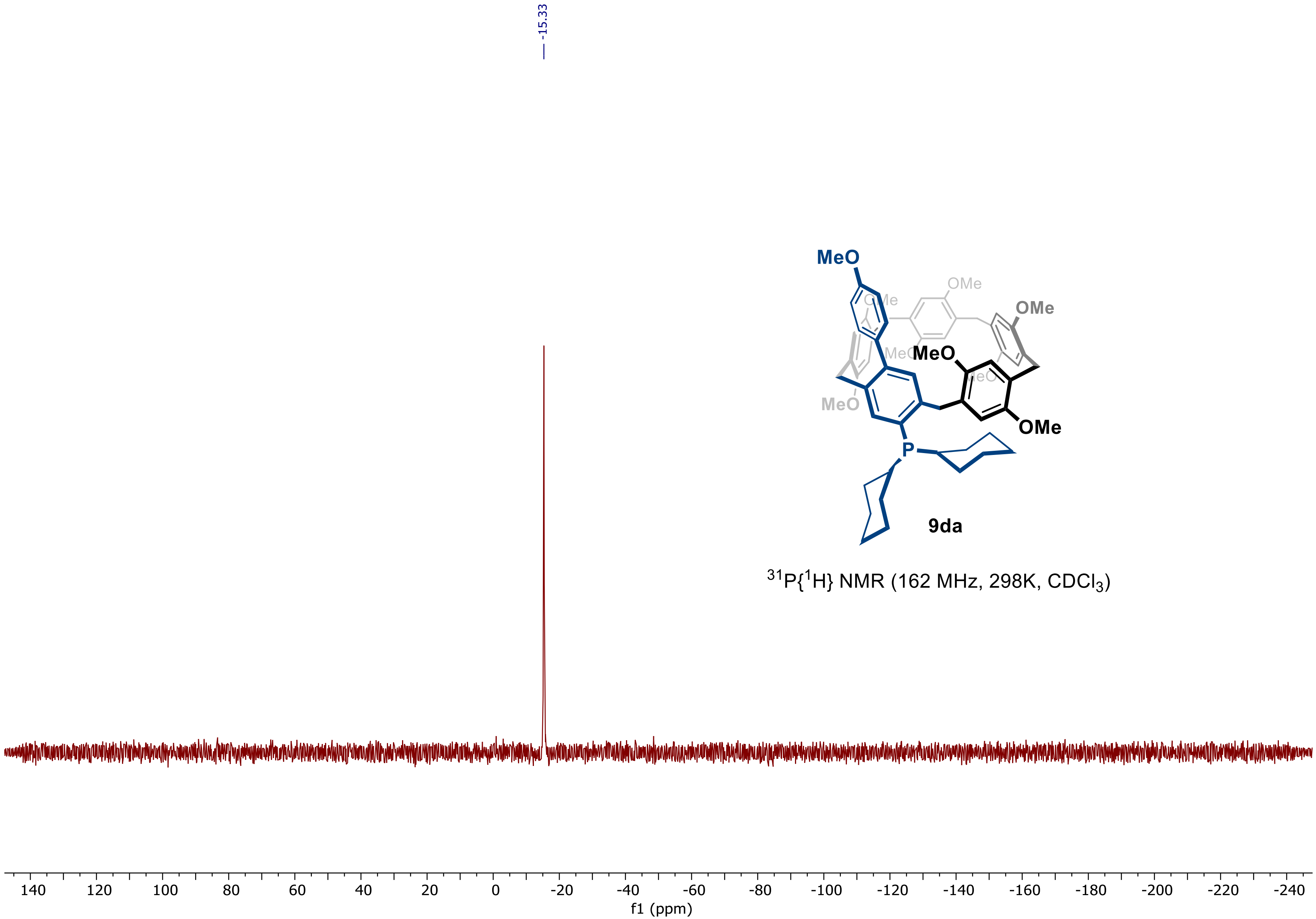

$^{31}P\{^{1}H\}$ NMR (162 MHz, 298K, $CDCl_3$)

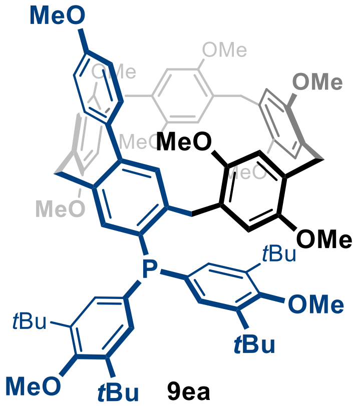

9ea

^{1}H NMR (400 MHz, 298K, $CDCl_3$)

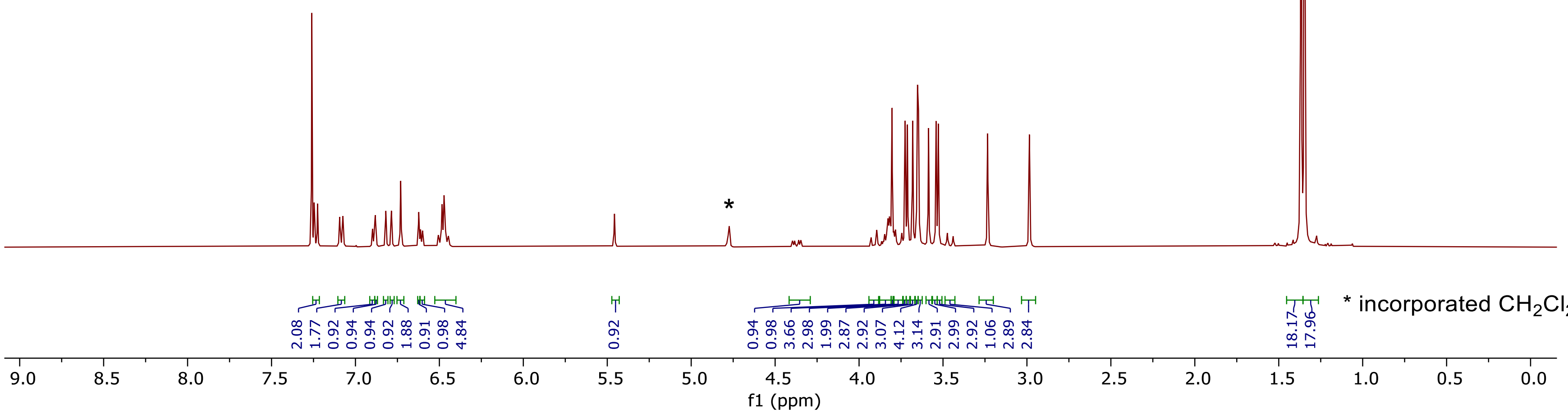

* incorporated CH_2Cl_2

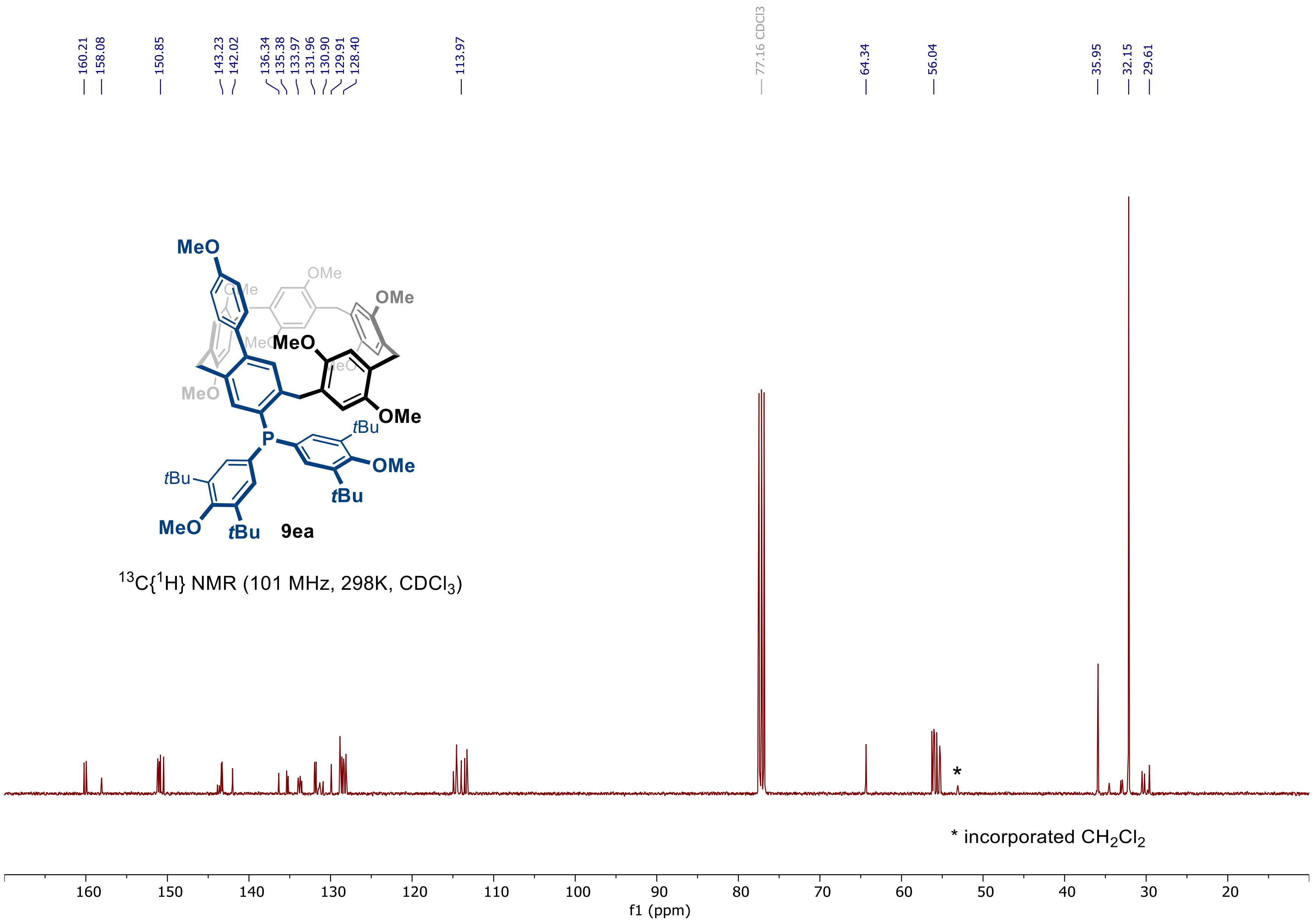
160.21
158.08
150.85
143.23
142.02
136.34
135.38
133.97
131.96
130.90
129.91
128.40
113.97
77.16 CDCl3
64.34
56.04
35.95
32.15
29.61
MeO
OMe
OMe
MeO
OMe
MeO
P
tBu
OMe
tBu
tBu
MeO
tBu
9ea
$^{13}C\{^1H\}$ NMR (101 MHz, 298K, $CDCl_3$)
* incorporated CH_2Cl_2
160
150
140
130
120
110
100
90
80
70
60
50
40
30
20
f1 (ppm)

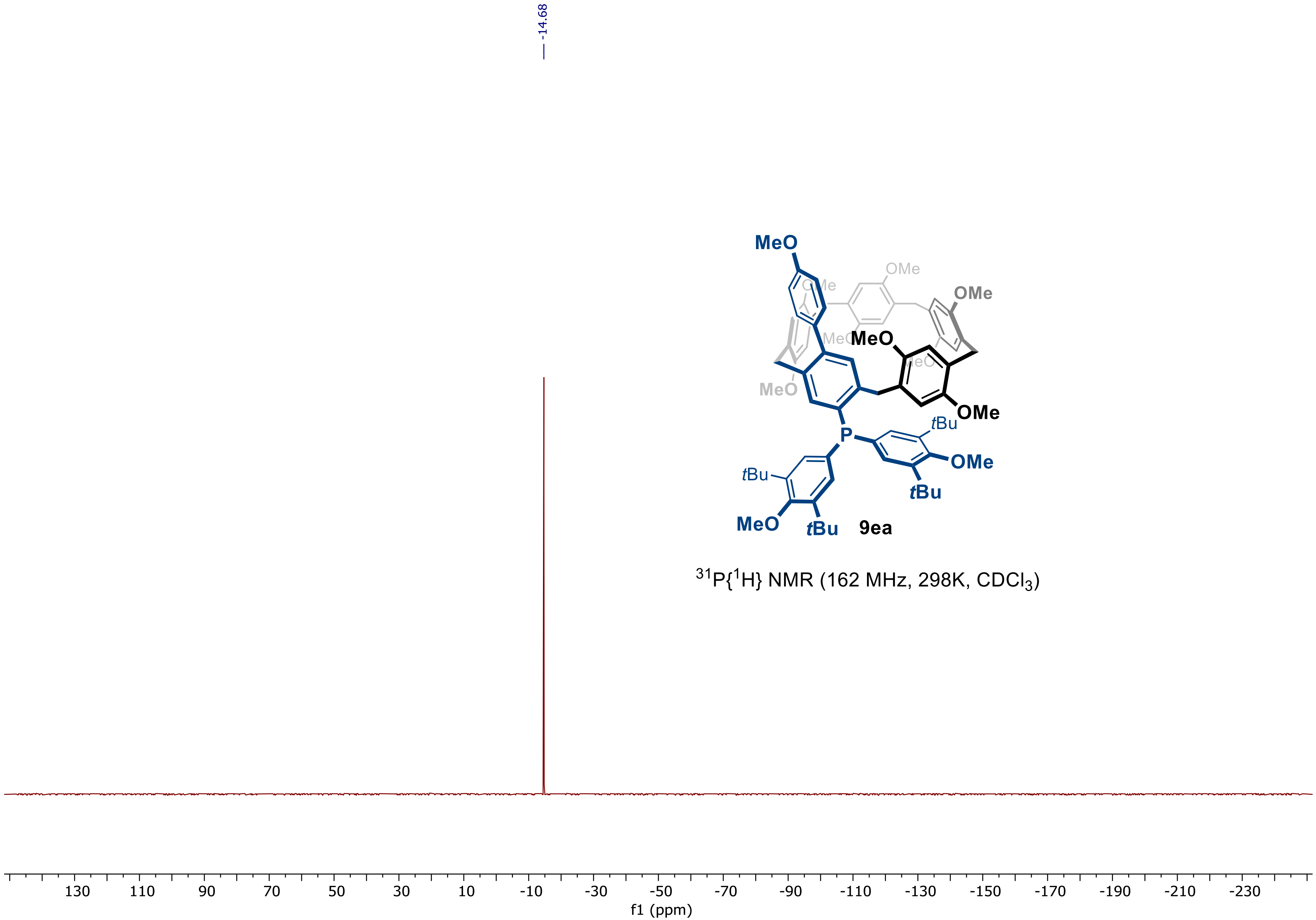

$^{31}P\{^{1}H\}$ NMR (162 MHz, 298K, $CDCl_3$)

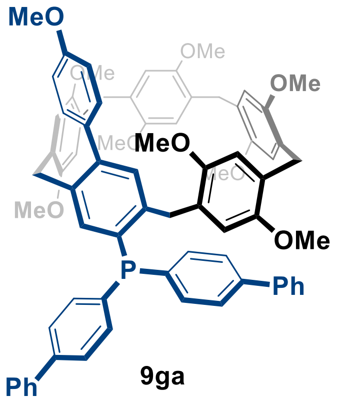

9ga

^{1}H NMR (400 MHz, 298K, $CDCl_3$)

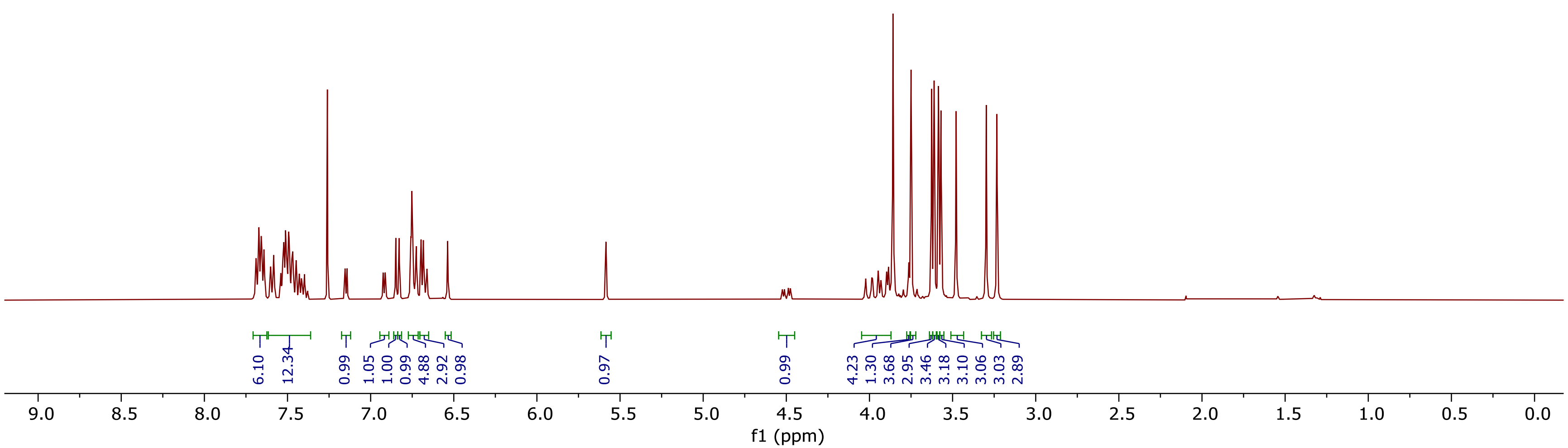

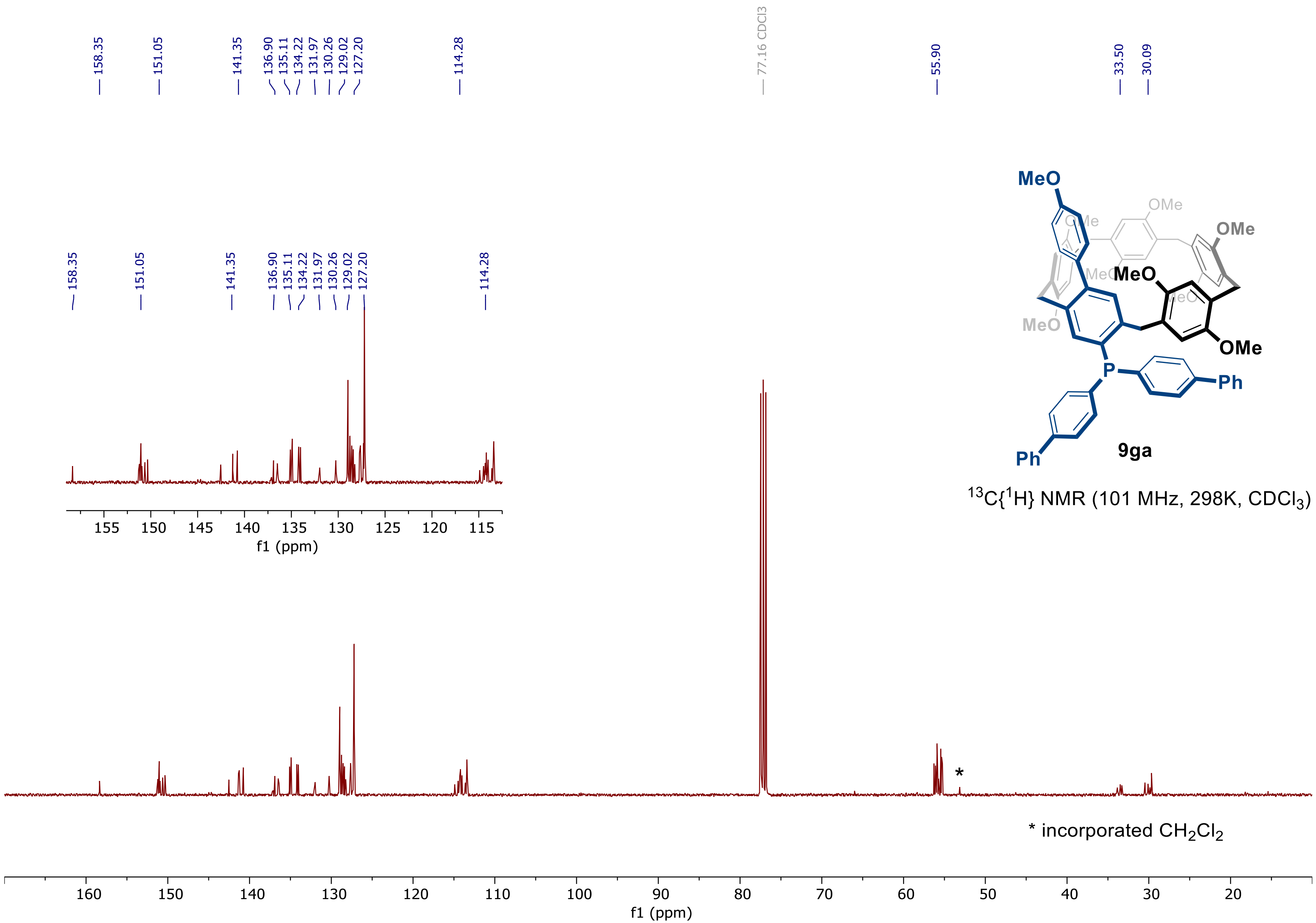

9ga

$^{13}C\{^{1}H\}$ NMR (101 MHz, 298K, $CDCl_3$)

* incorporated CH_2Cl_2

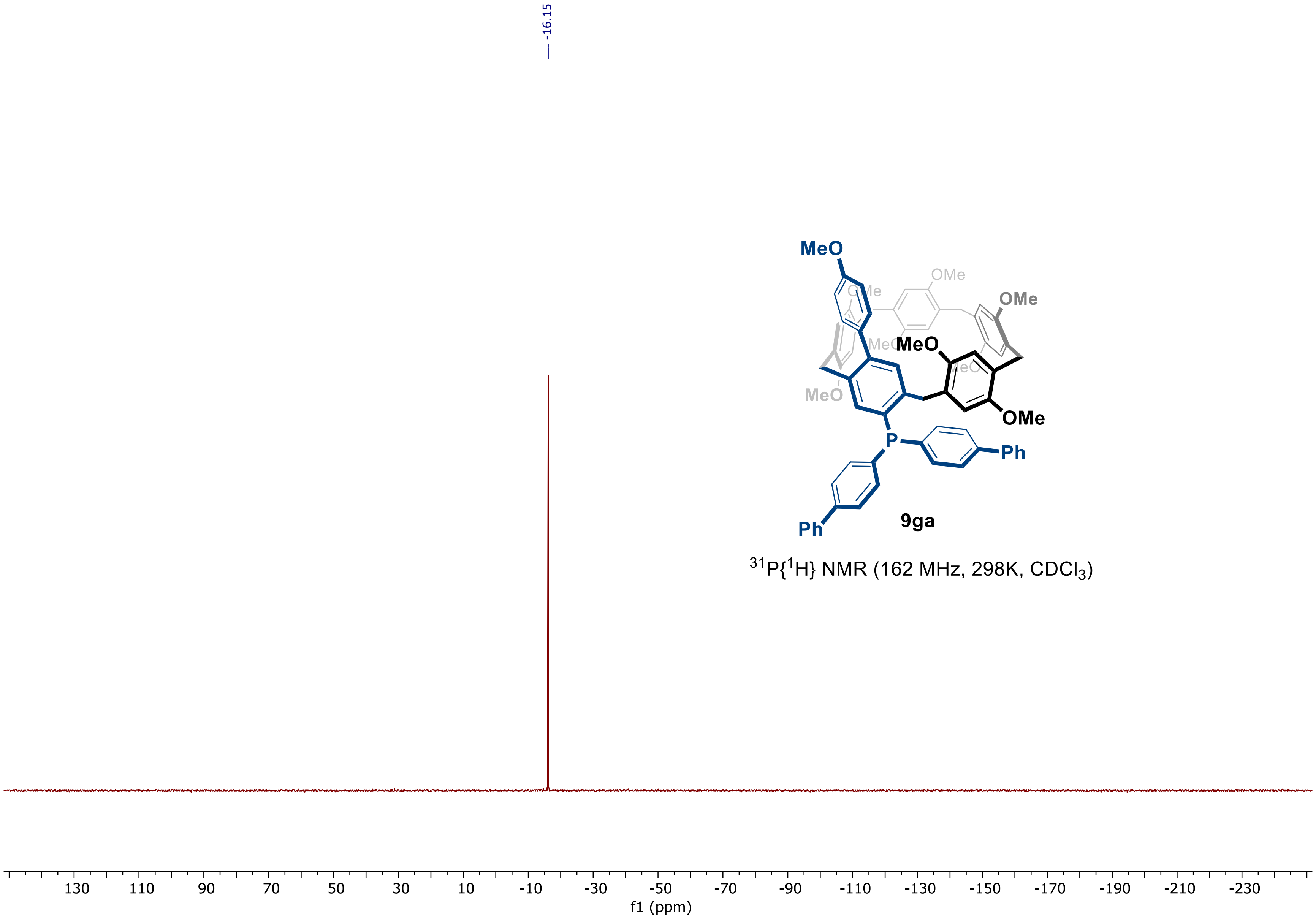

9ga

^{31}P{^{1}H} NMR (162 MHz, 298K, $CDCl_3$)

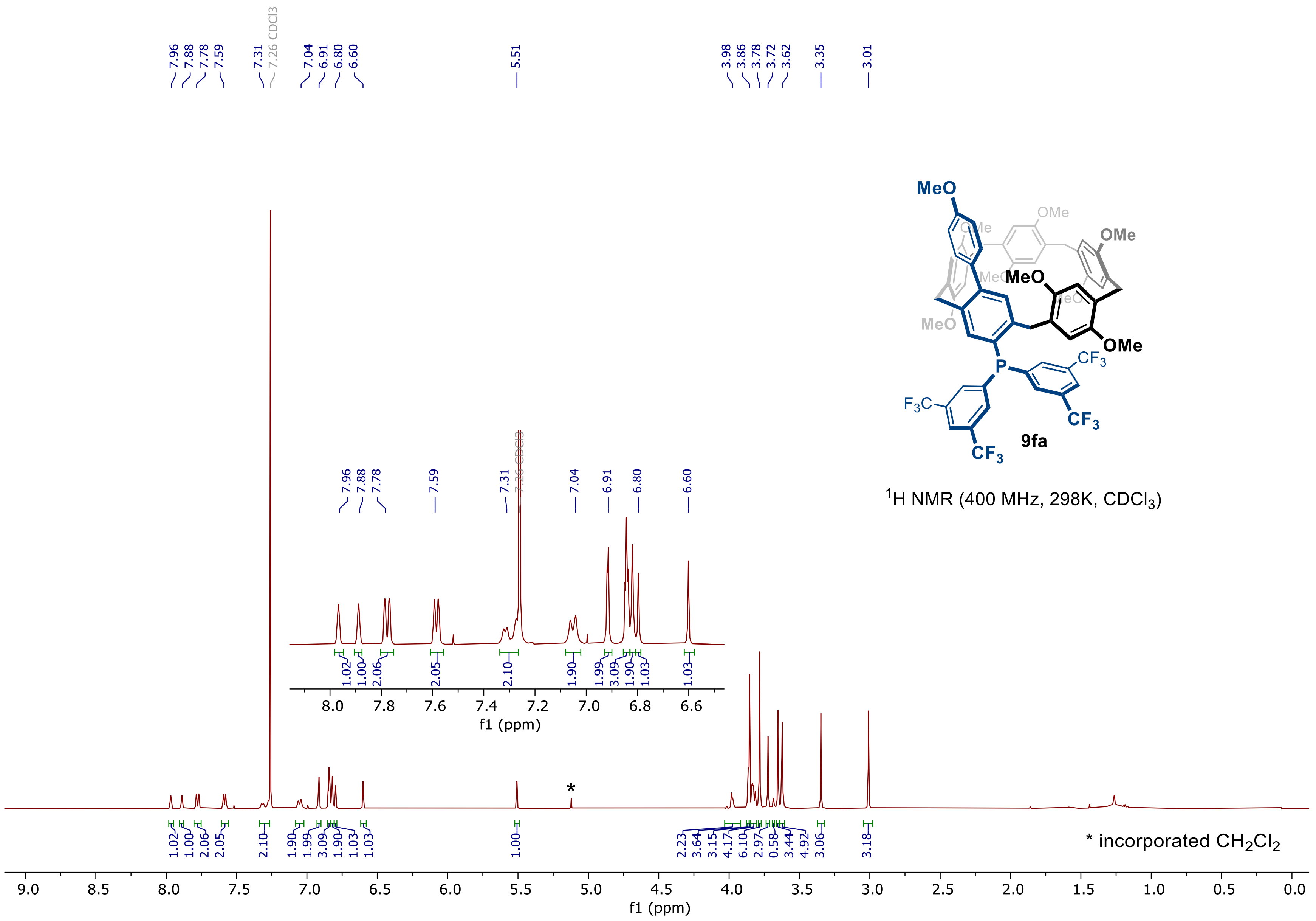
7.96
7.88
7.78
7.59
7.31
7.26 CDCl3
7.04
6.91
6.80
6.60
5.51
3.98
3.86
3.78
3.72
3.62
3.35
3.01
MeO
OMe
OMe
MeO
MeO
OMe
P
CF3
F3C
CF3
CF3
9fa
1H NMR (400 MHz, 298K, CDCl3)
* incorporated CH2Cl2
1.02
1.00
2.06
2.05
2.10
1.90
1.99
3.09
1.90
1.03
1.03
1.00
2.23
3.64
3.15
4.17
6.10
2.97
0.58
3.44
4.92
3.06
3.18
f1 (ppm)

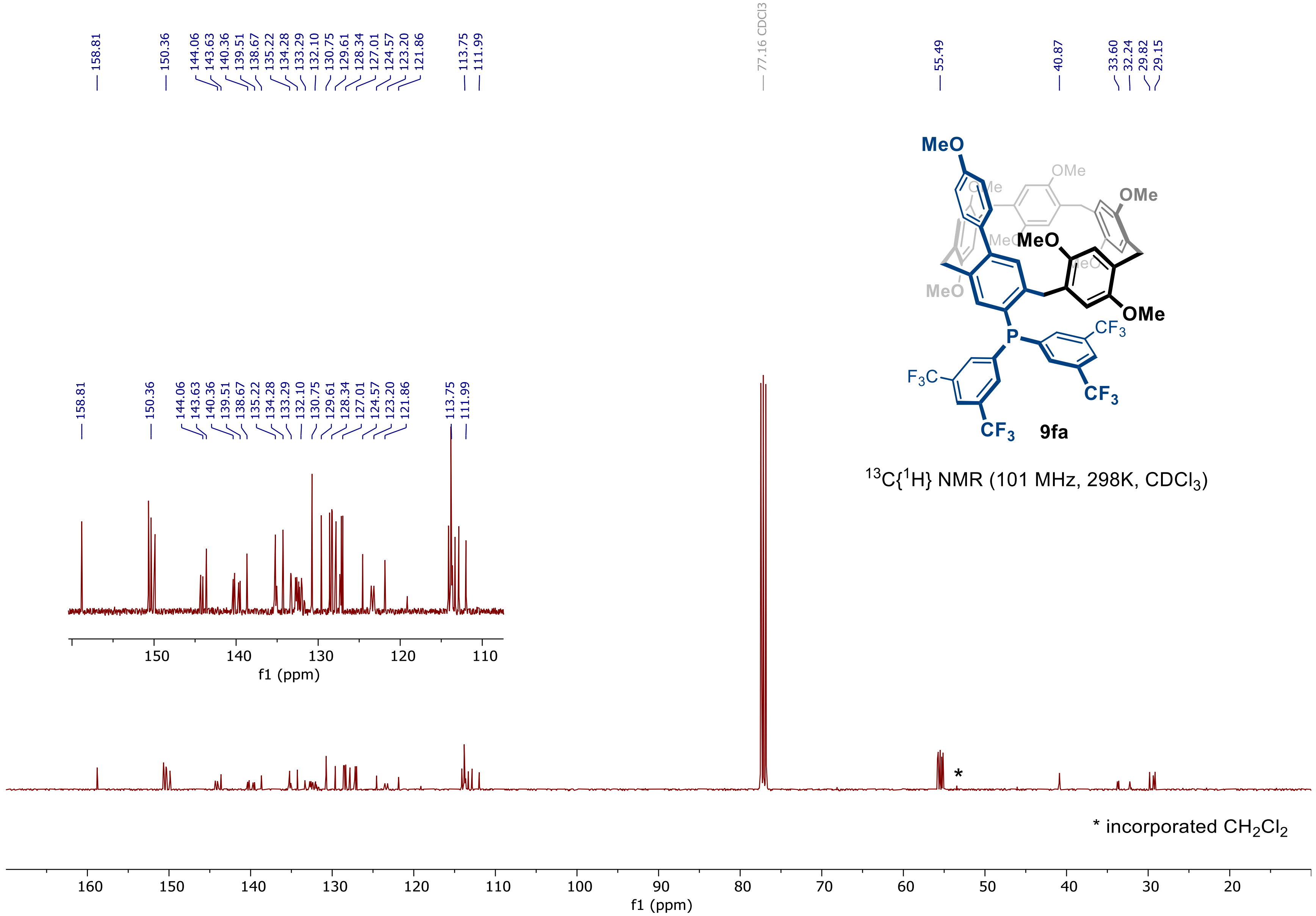
158.81
150.36
144.06
143.63
140.36
139.51
138.67
135.22
134.28
133.29
132.10
130.75
129.61
128.34
127.01
124.57
123.20
121.86
113.75
111.99
77.16 CDCl3
55.49
40.87
33.60
32.24
29.82
29.15
MeO
OMe
MeO
MeO
OMe
P
CF3
F3C
CF3
CF3
9fa
13C{1H} NMR (101 MHz, 298K, CDCl3)
*
* incorporated CH2Cl2
150
140
130
120
110
f1 (ppm)
160
150
140
130
120
110
100
90
80
70
60
50
40
30
20
f1 (ppm)

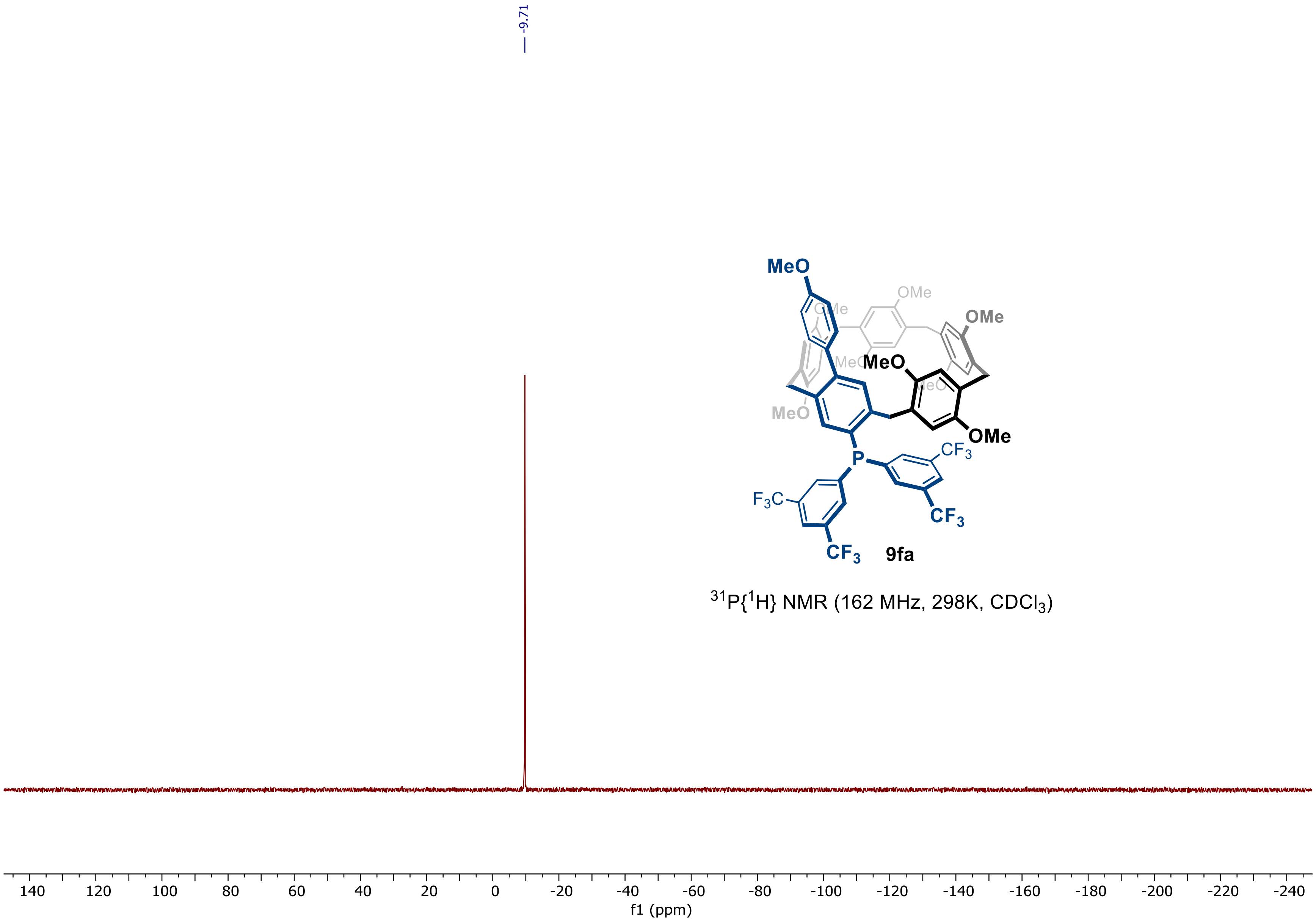

$^{31}P\{^{1}H\}$ NMR (162 MHz, 298K, $CDCl_3$)

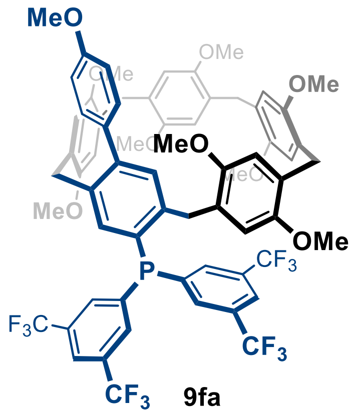

9fa

$^{19}F\{^{1}H\}$ NMR (282 MHz, 298K, $CDCl_3$)

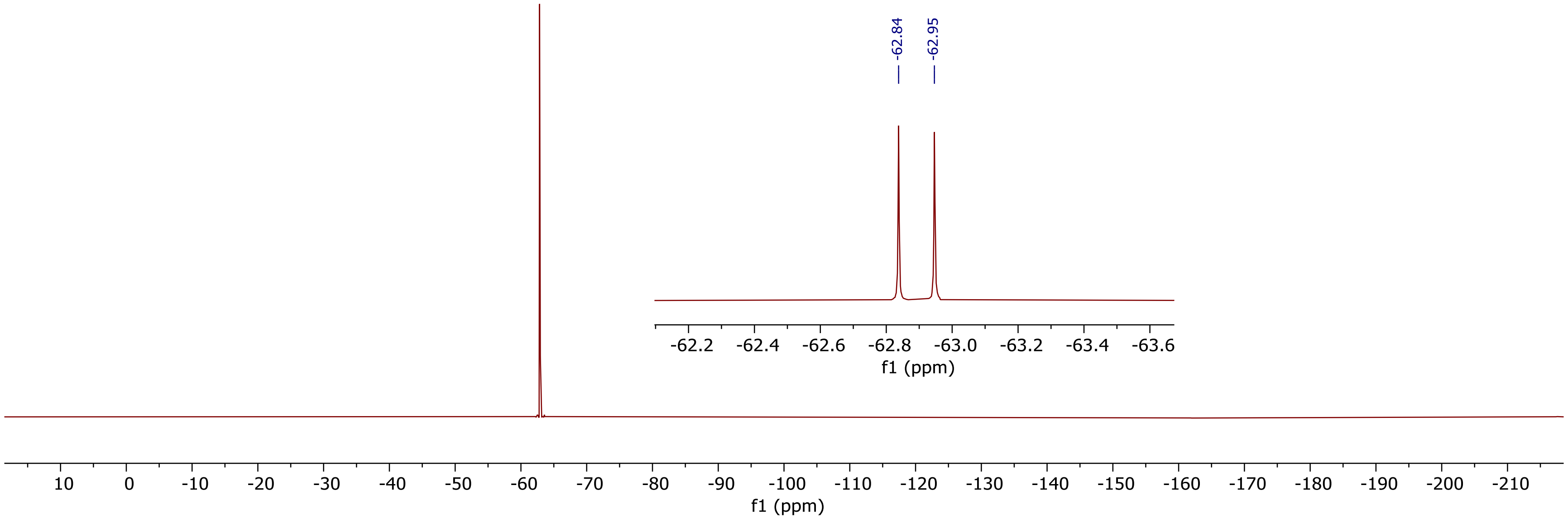

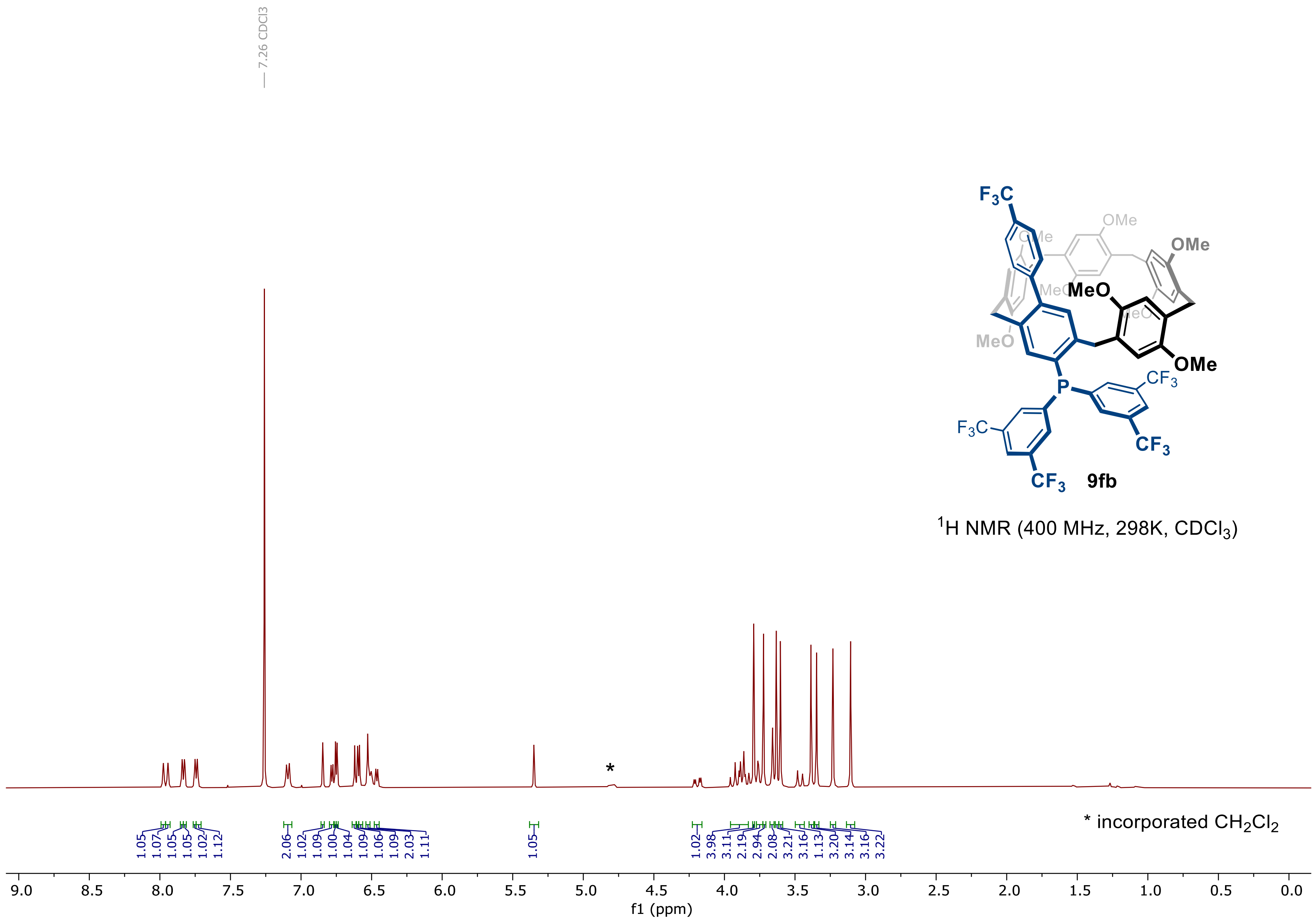
7.26 CDCl3
F3C
OMe
OMe
MeO
MeO
OMe
CF3
P
F3C
CF3
CF3
9fb
1H NMR (400 MHz, 298K, CDCl3)
*
* incorporated CH2Cl2
1.05
1.07
1.05
1.05
1.02
1.12
2.06
1.02
1.09
1.00
1.04
1.09
1.06
1.09
2.03
1.11
1.05
1.02
3.98
3.11
2.19
2.94
2.08
3.21
3.16
1.13
3.20
3.14
3.16
3.22
9.0
8.5
8.0
7.5
7.0
6.5
6.0
5.5
5.0
4.5
4.0
3.5
3.0
2.5
2.0
1.5
1.0
0.5
0.0
f1 (ppm)

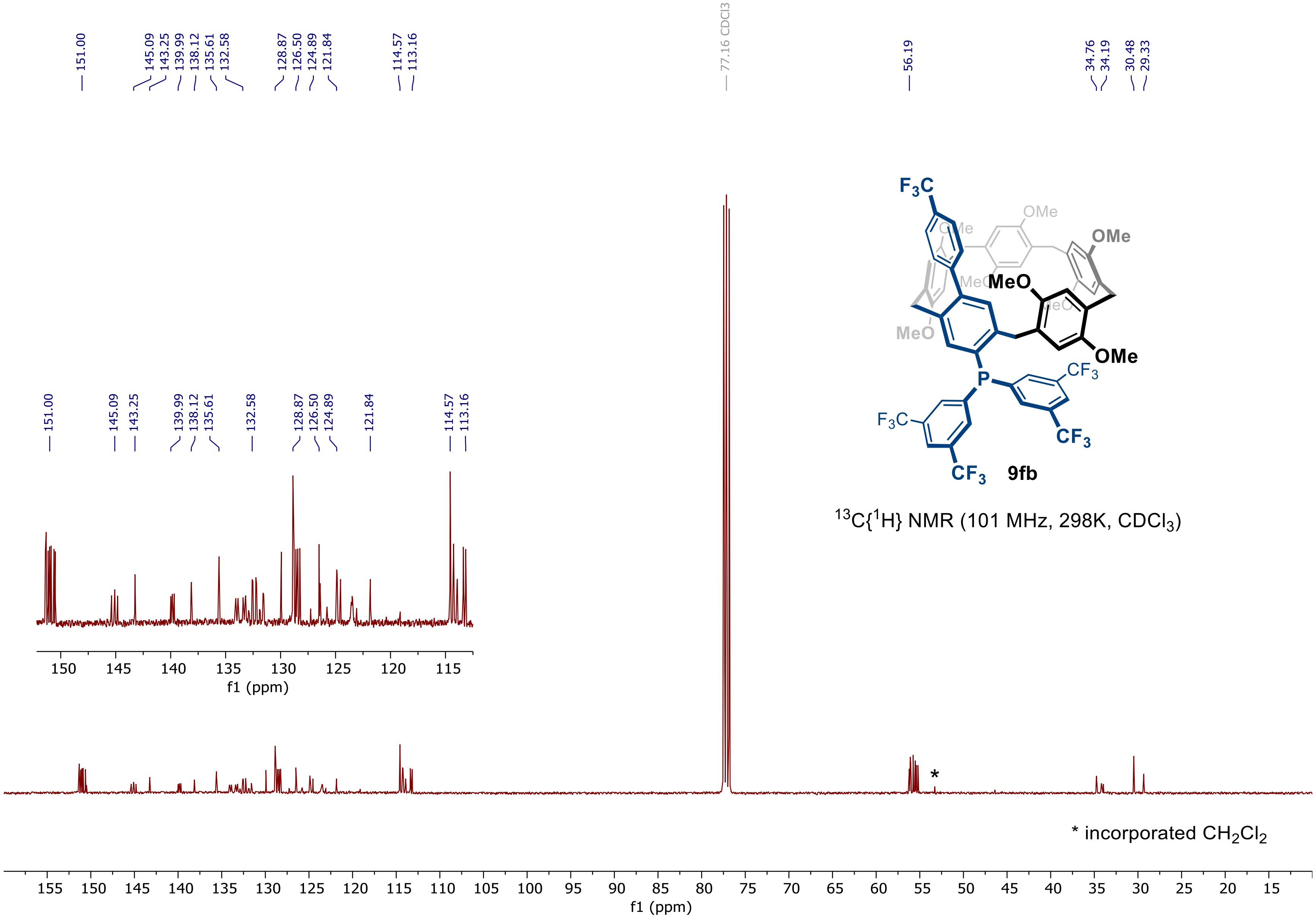

$^{13}C\{^{1}H\}$ NMR (101 MHz, 298K, $CDCl_3$)

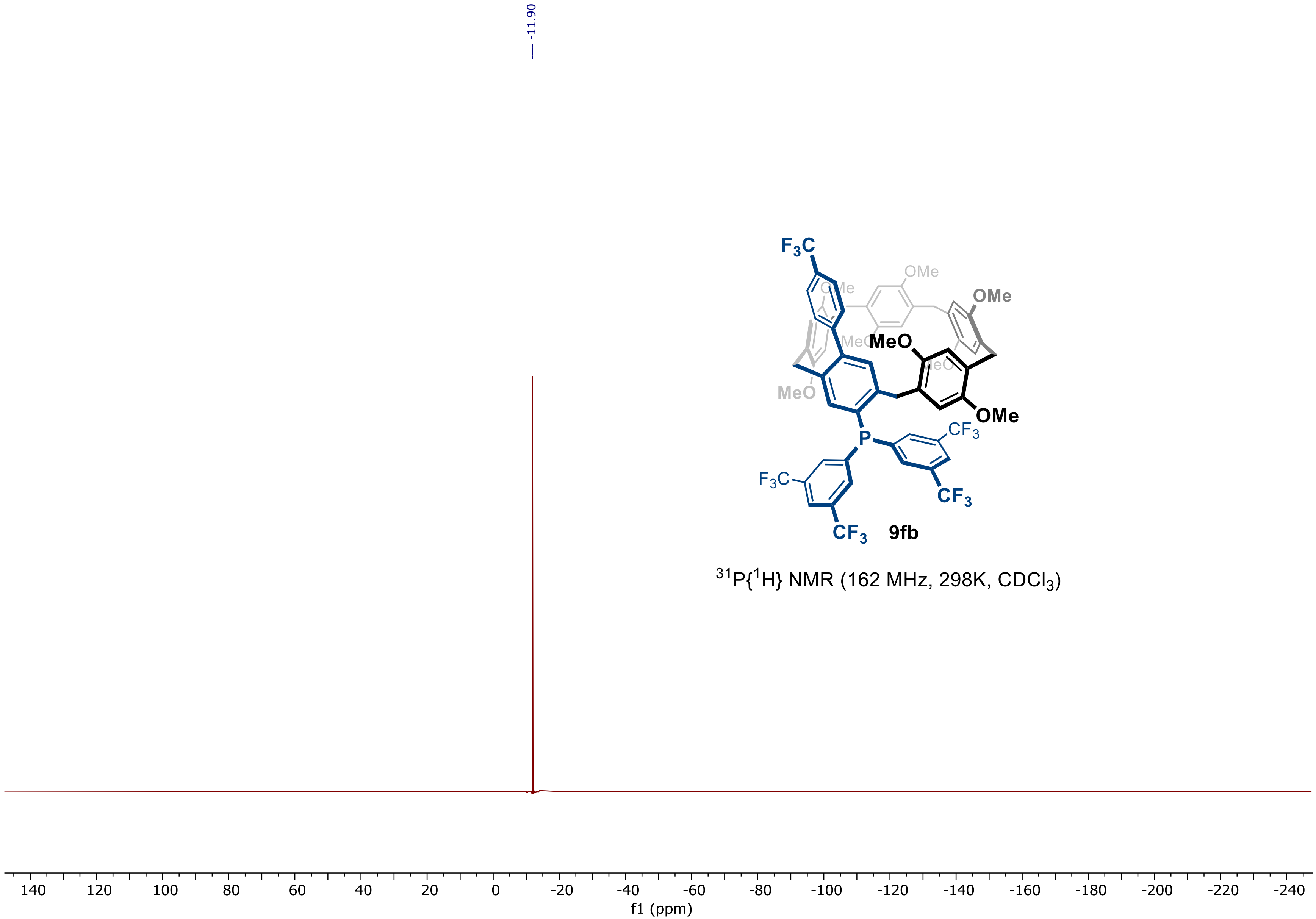
-11.90
F3C
OMe
OMe
MeO
MeO
OMe
P
CF3
F3C
CF3
CF3
9fb
31P{1H} NMR (162 MHz, 298K, CDCl3)
140 120 100 80 60 40 20 0 -20 -40 -60 -80 -100 -120 -140 -160 -180 -200 -220 -240
f1 (ppm)

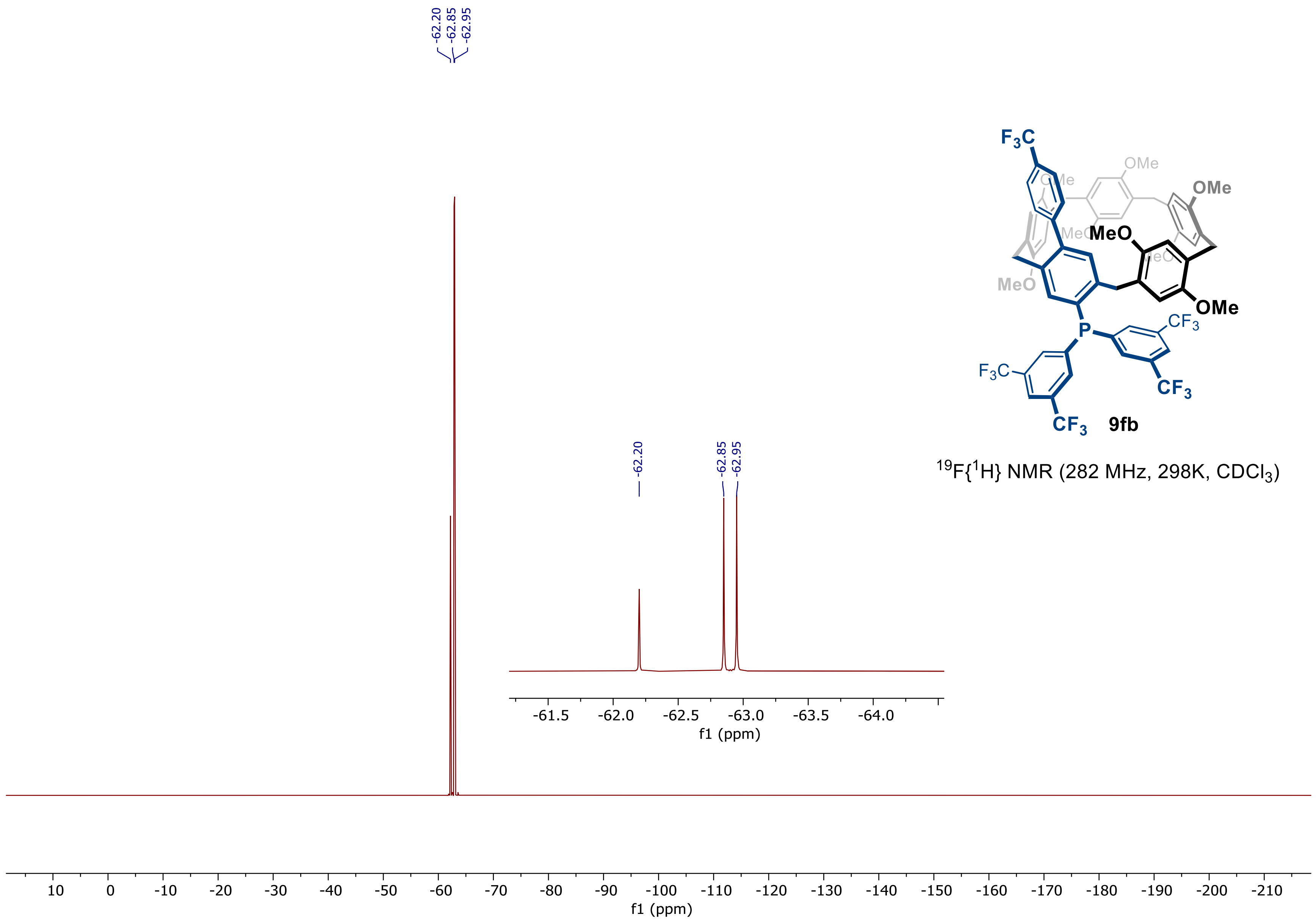

$^{19}F\{^{1}H\}$ NMR (282 MHz, 298K, $CDCl_3$)

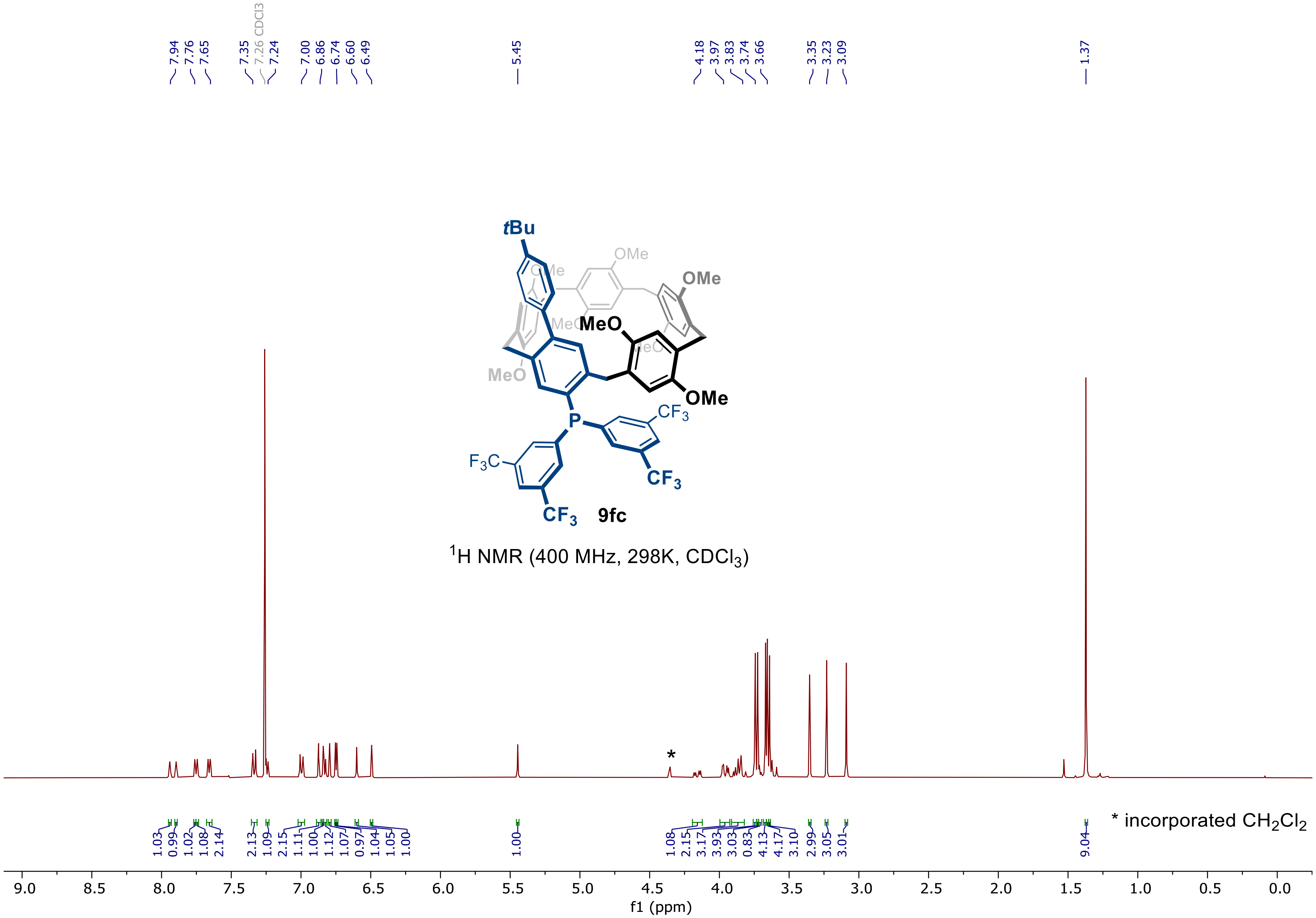

^{1}H NMR (400 MHz, 298K, $CDCl_3$)

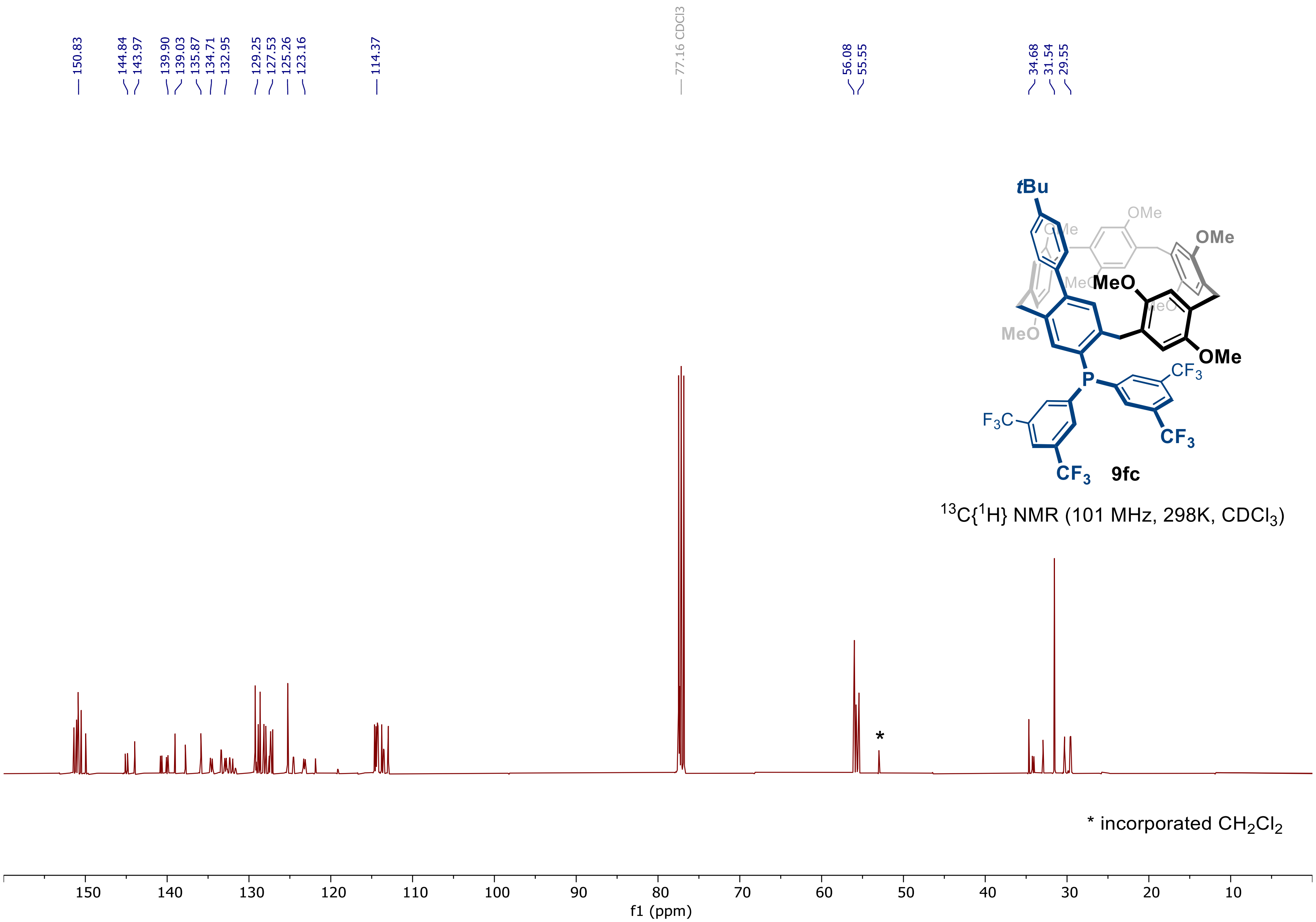

$^{13}C\{^{1}H\}$ NMR (101 MHz, 298K, $CDCl_3$)

* incorporated CH_2Cl_2

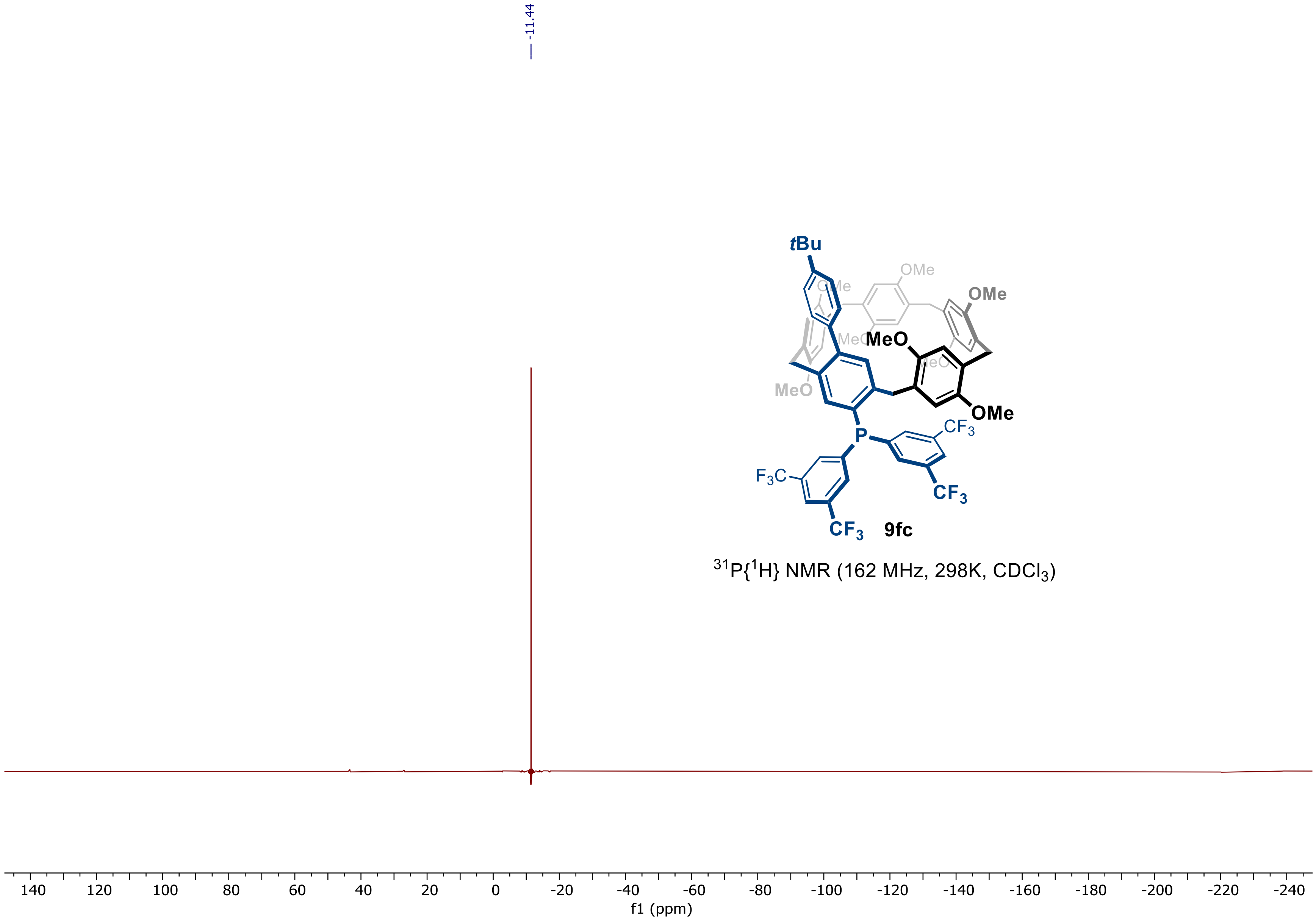

9fc

$^{31}P\{^1H\}$ NMR (162 MHz, 298K, $CDCl_3$)

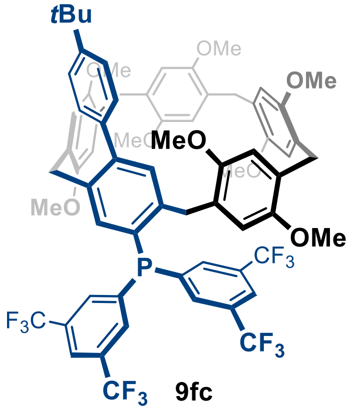

9fc

$^{19}F\{^{1}H\}$ NMR (282 MHz, 298K, $CDCl_3$)

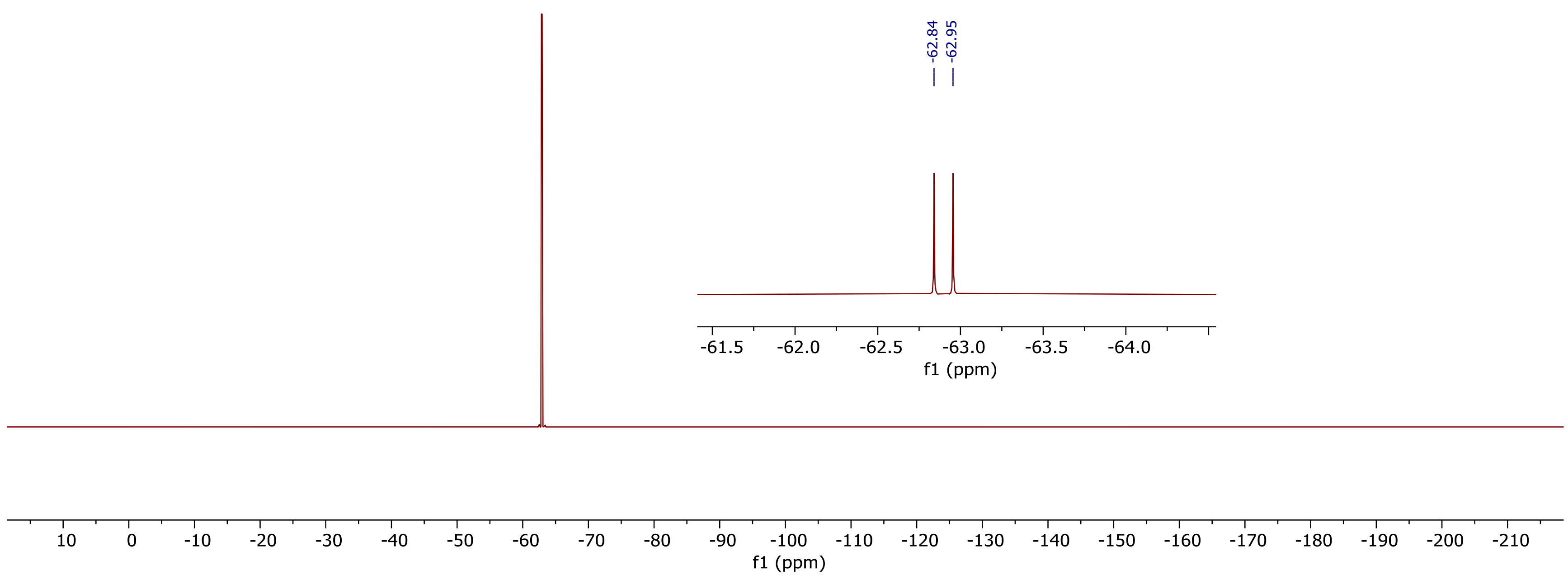

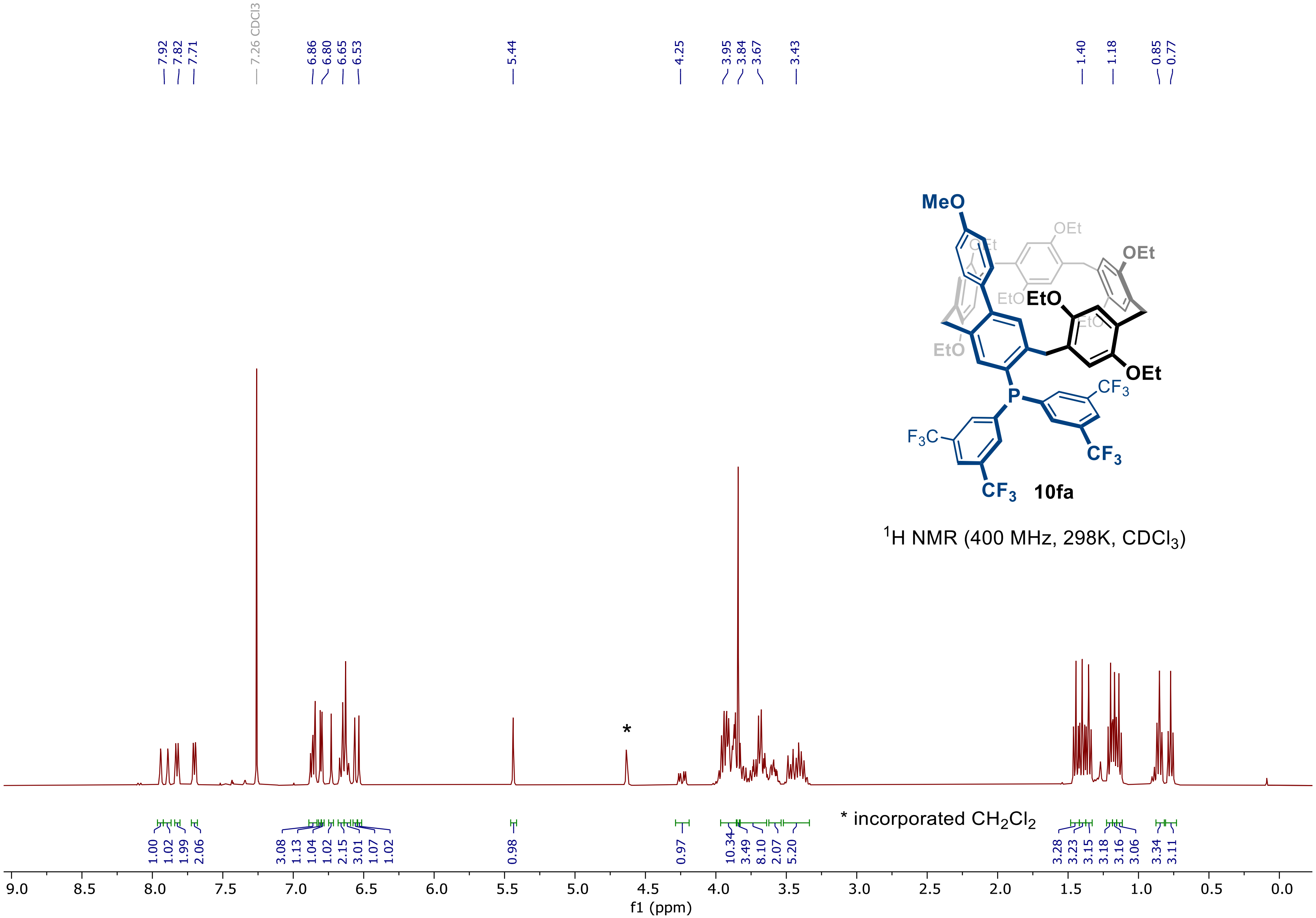
7.92
7.82
7.71
7.26 CDCl3
6.86
6.80
6.65
6.53
5.44
4.25
3.95
3.84
3.67
3.43
1.40
1.18
0.85
0.77
MeO
OEt
OEt
EtO
EtO
EtO
EtO
OEt
P
CF3
F3C
CF3
CF3
10fa
1H NMR (400 MHz, 298K, CDCl3)
*
* incorporated CH2Cl2
1.00
1.02
1.99
2.06
3.08
1.13
1.04
1.02
2.15
3.01
1.07
1.02
0.98
0.97
10.34
3.49
8.10
2.07
5.20
3.28
3.23
3.15
3.18
3.16
3.06
3.34
3.11
9.0
8.5
8.0
7.5
7.0
6.5
6.0
5.5
5.0
4.5
4.0
3.5
3.0
2.5
2.0
1.5
1.0
0.5
0.0
f1 (ppm)

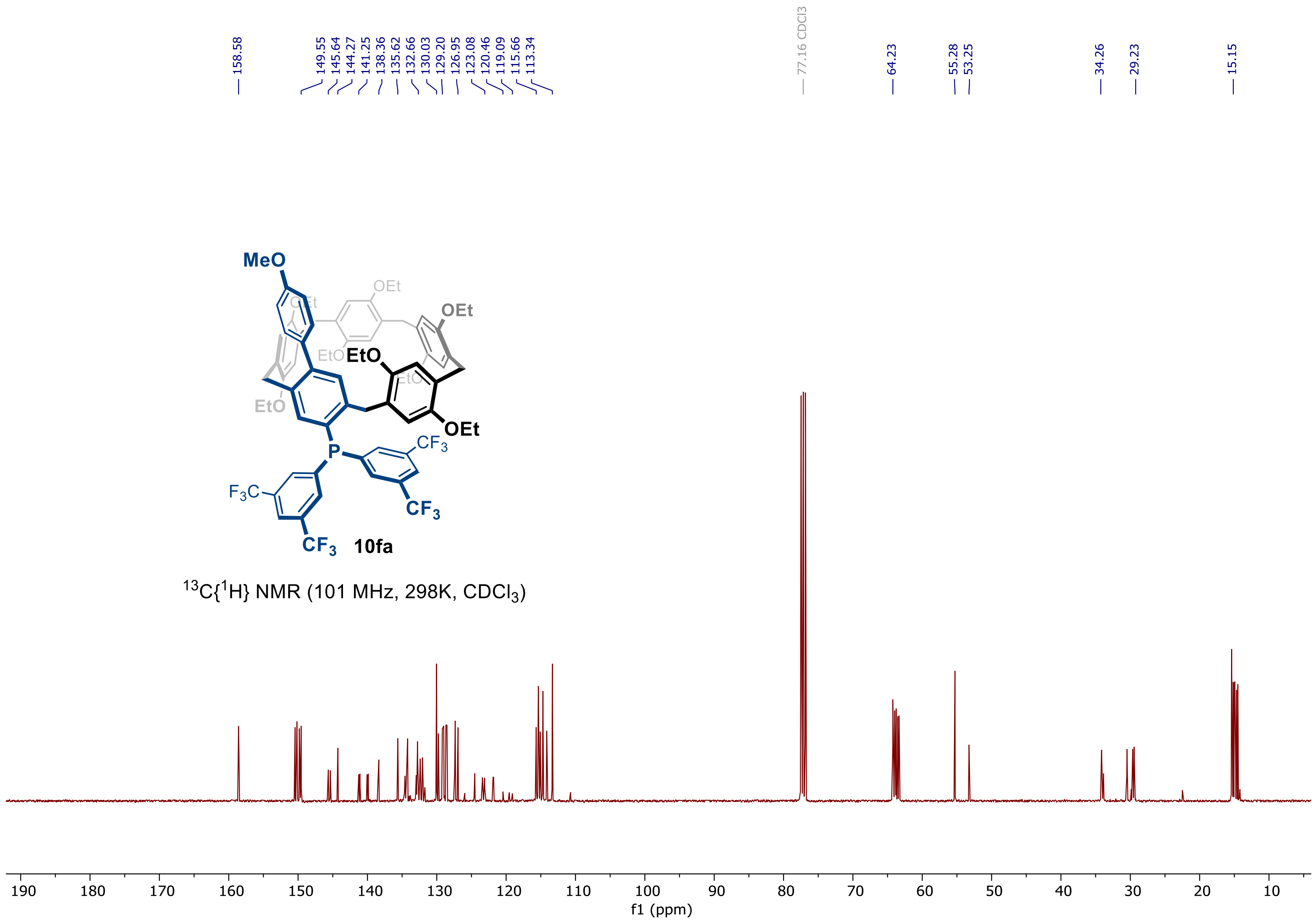
158.58
149.55
145.64
144.27
141.25
138.36
135.62
132.66
130.03
129.20
126.95
123.08
120.46
119.09
115.66
113.34
77.16 CDCl3
64.23
55.28
53.25
34.26
29.23
15.15
MeO
OEt
OEt
EtO
EtO
OEt
EtO
P
CF3
F3C
CF3
CF3
10fa
13C{1H} NMR (101 MHz, 298K, CDCl3)
190
180
170
160
150
140
130
120
110
100
90
80
70
60
50
40
30
20
10
f1 (ppm)

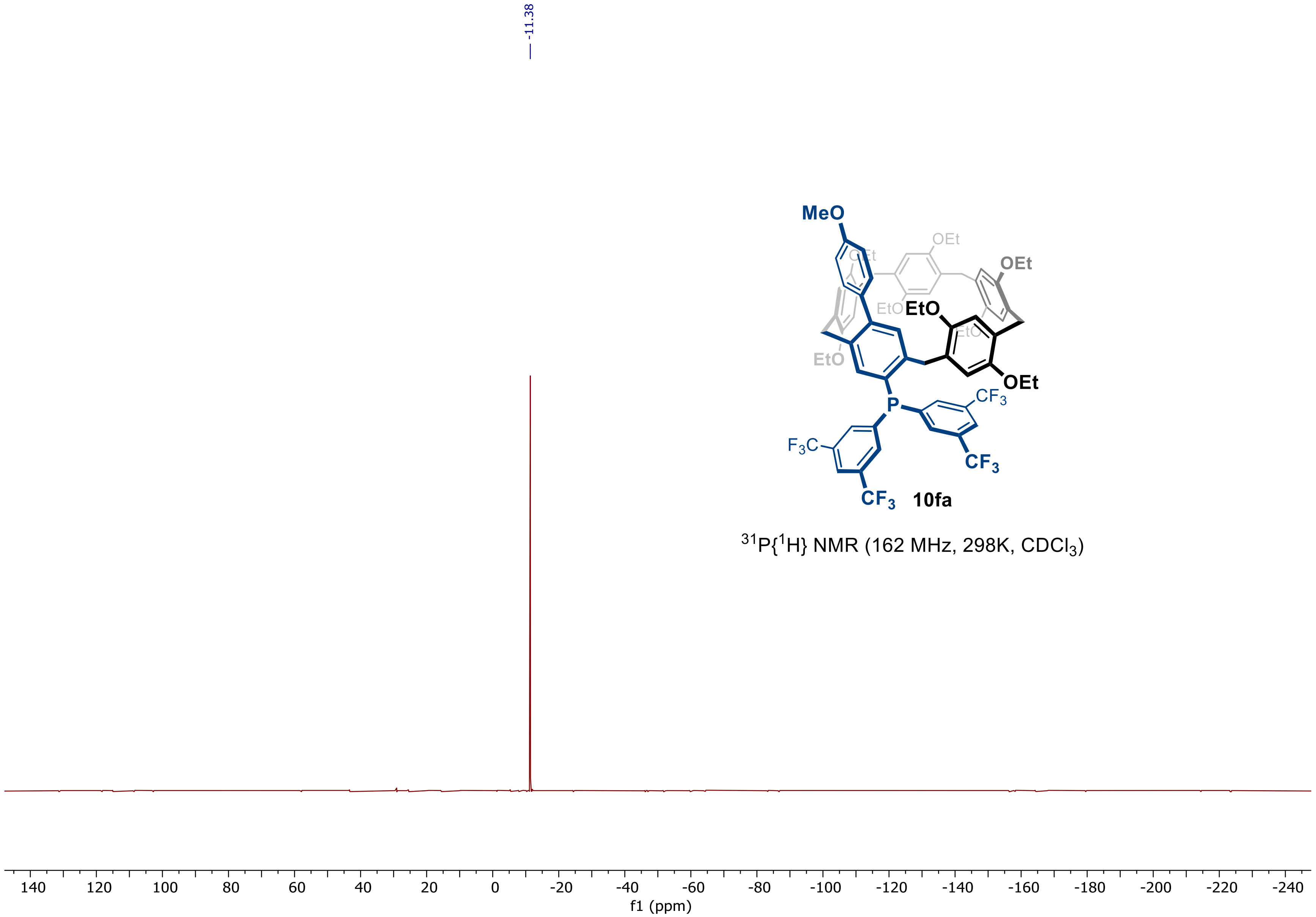

10fa

$^{31}P\{^1H\}$ NMR (162 MHz, 298K, $CDCl_3$)

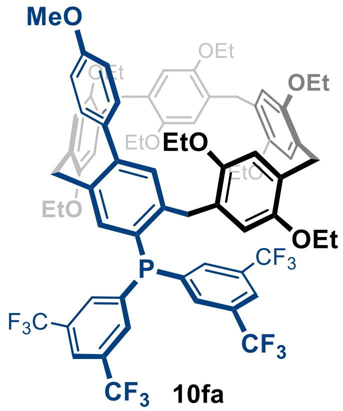

10fa

$^{19}F\{^1H\}$ NMR (282 MHz, 298K, $CDCl_3$)

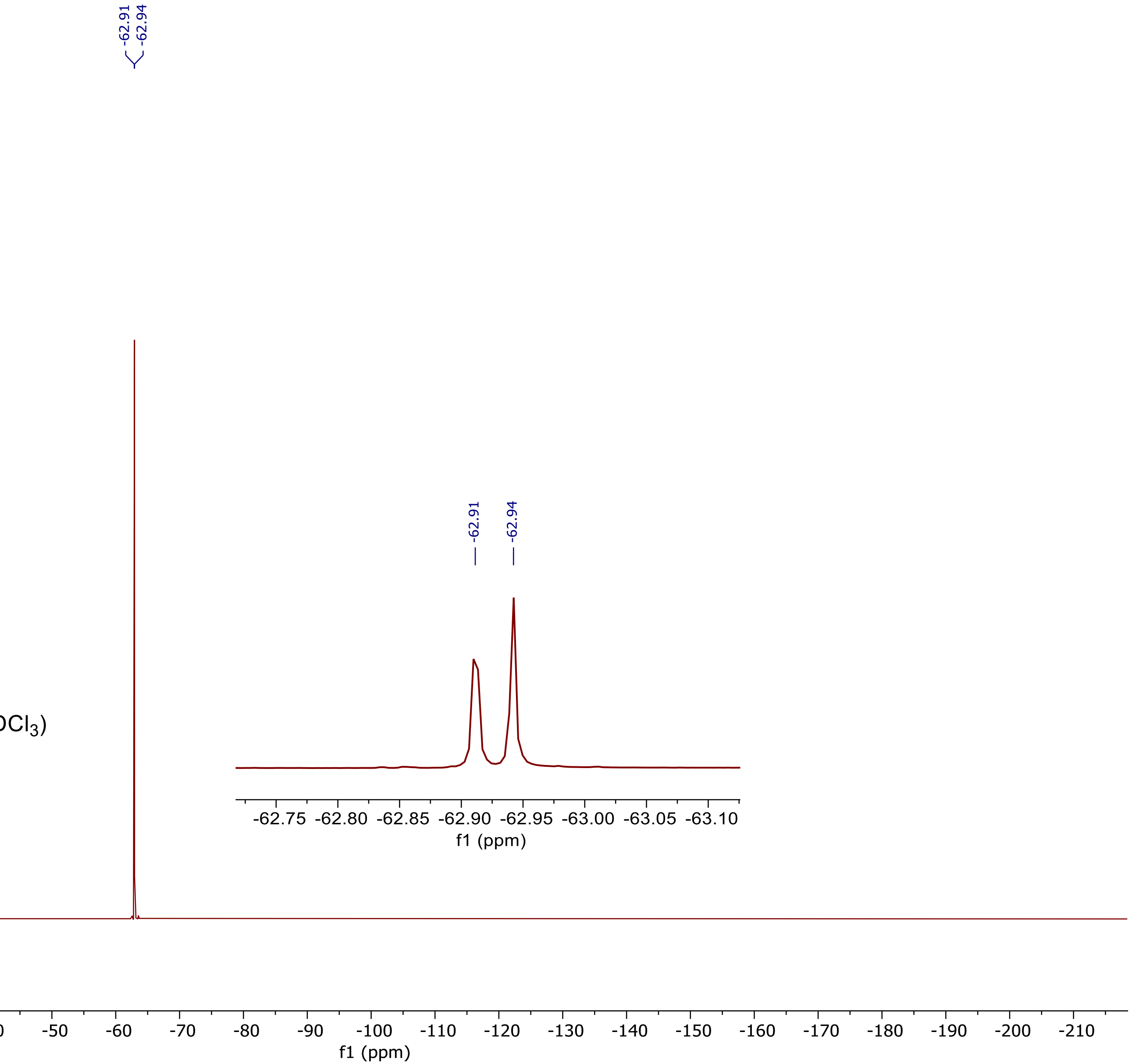

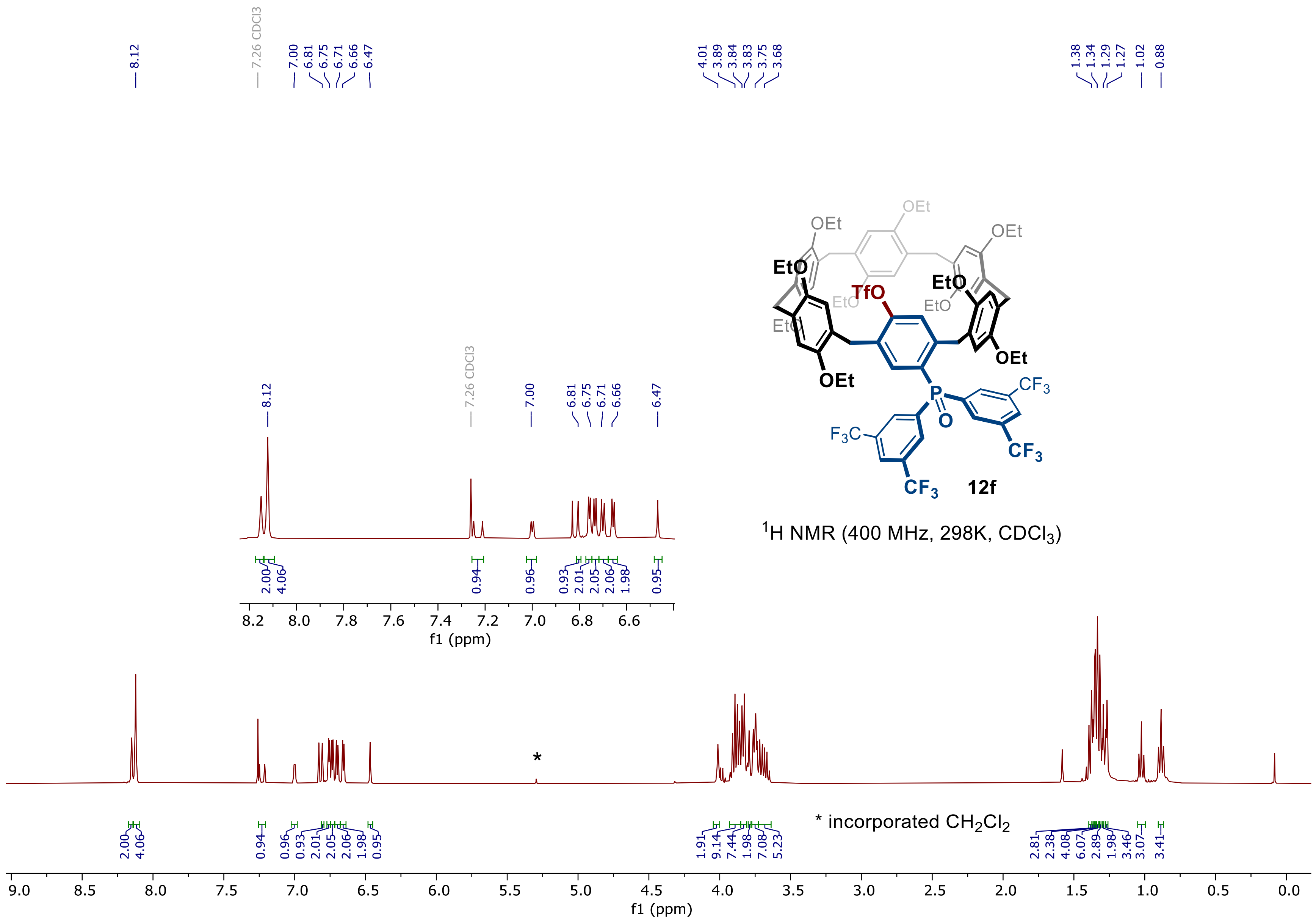

OEt
EtO
TfO
EtO
P
O
CF3
F3C
CF3
CF3
12f
1H NMR (400 MHz, 298K, CDCl3)
* incorporated CH2Cl2
8.12
7.26 CDCl3
7.00
6.81
6.75
6.71
6.66
6.47
4.01
3.89
3.84
3.83
3.75
3.68
1.38
1.34
1.29
1.27
1.02
0.88
f1 (ppm)

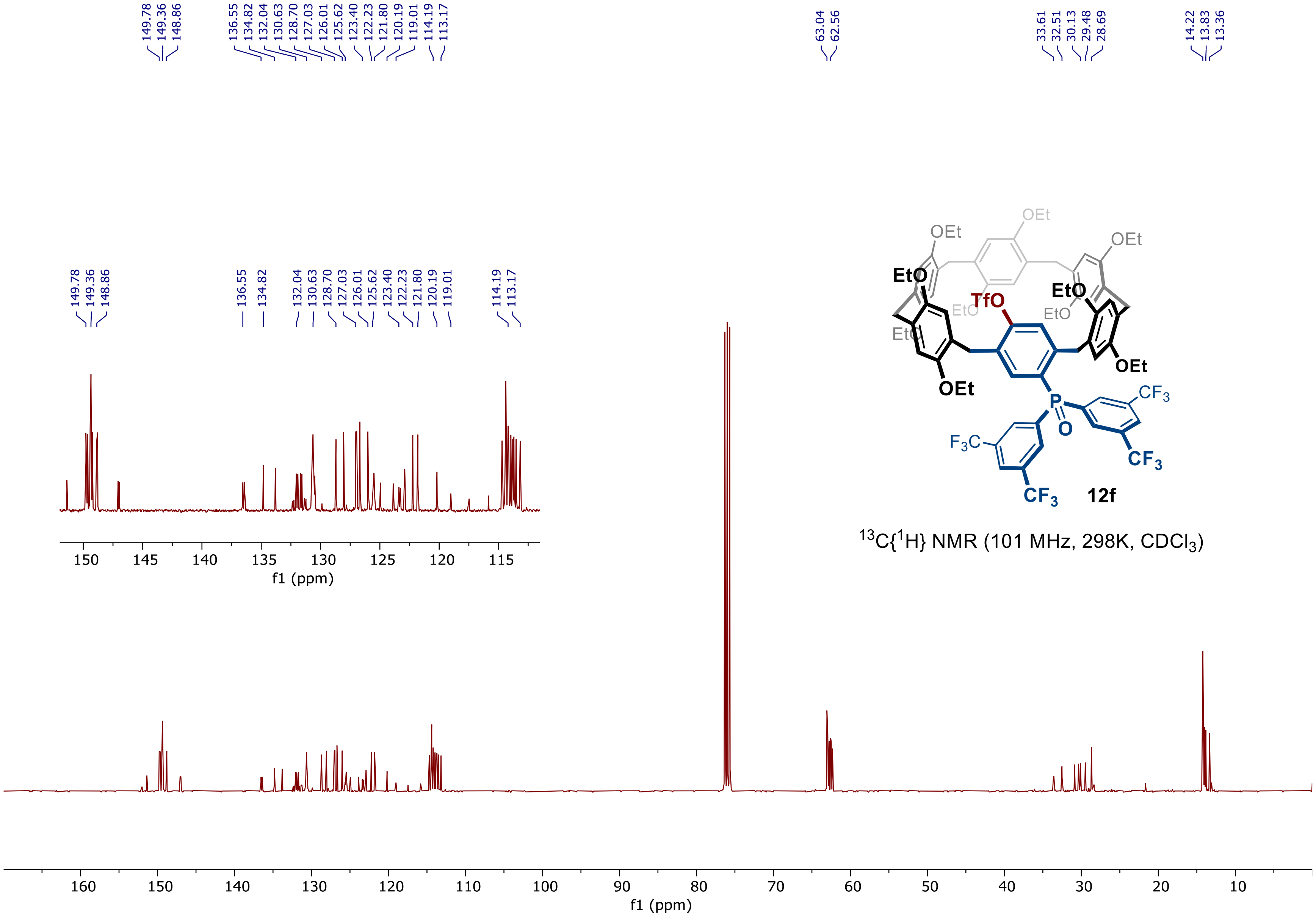

$^{13}C\{^1H\}$ NMR (101 MHz, 298K, $CDCl_3$)

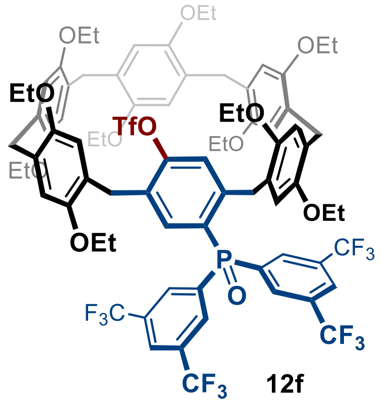

12f

$^{31}P\{^{1}H\}$ NMR (162 MHz, 298K, $CDCl_3$)

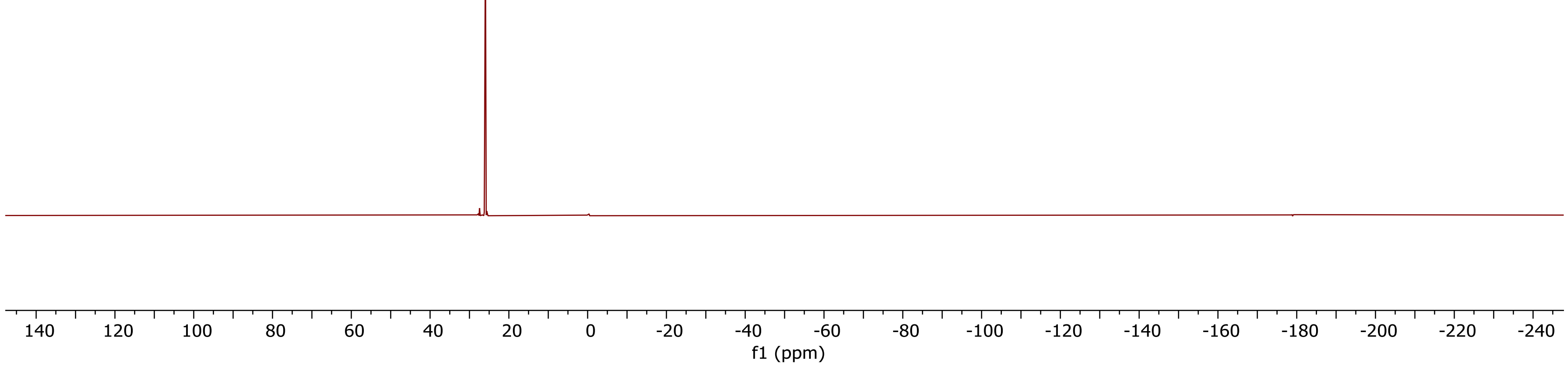

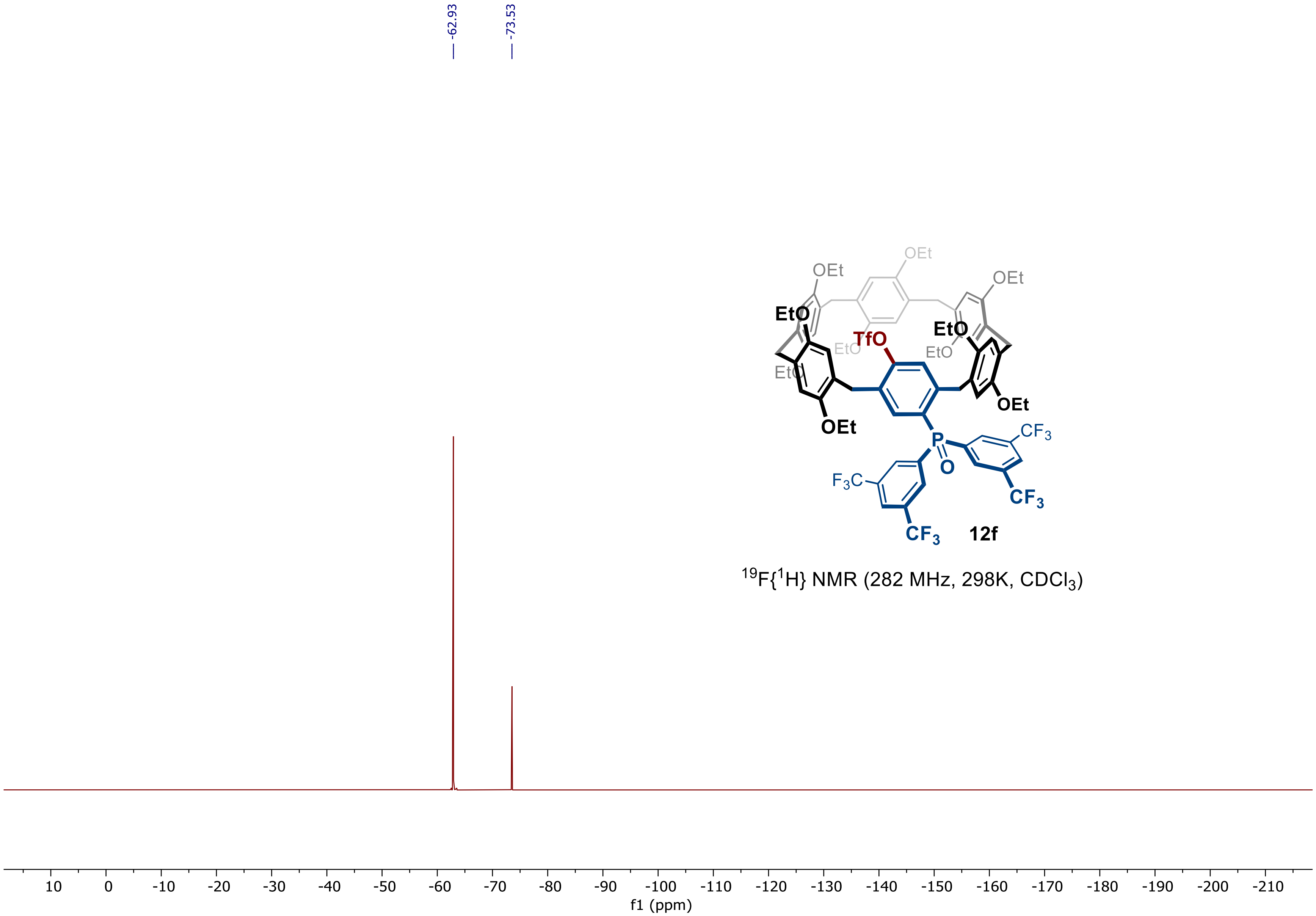
-62.93
-73.53
OEt
OEt
OEt
EtO
TfO
EtO
EtO
EtO
EtO
OEt
OEt
P
O
CF3
F3C
CF3
CF3
12f
19F{1H} NMR (282 MHz, 298K, CDCl3)
10
0
-10
-20
-30
-40
-50
-60
-70
-80
-90
-100
-110
-120
-130
-140
-150
-160
-170
-180
-190
-200
-210
f1 (ppm)

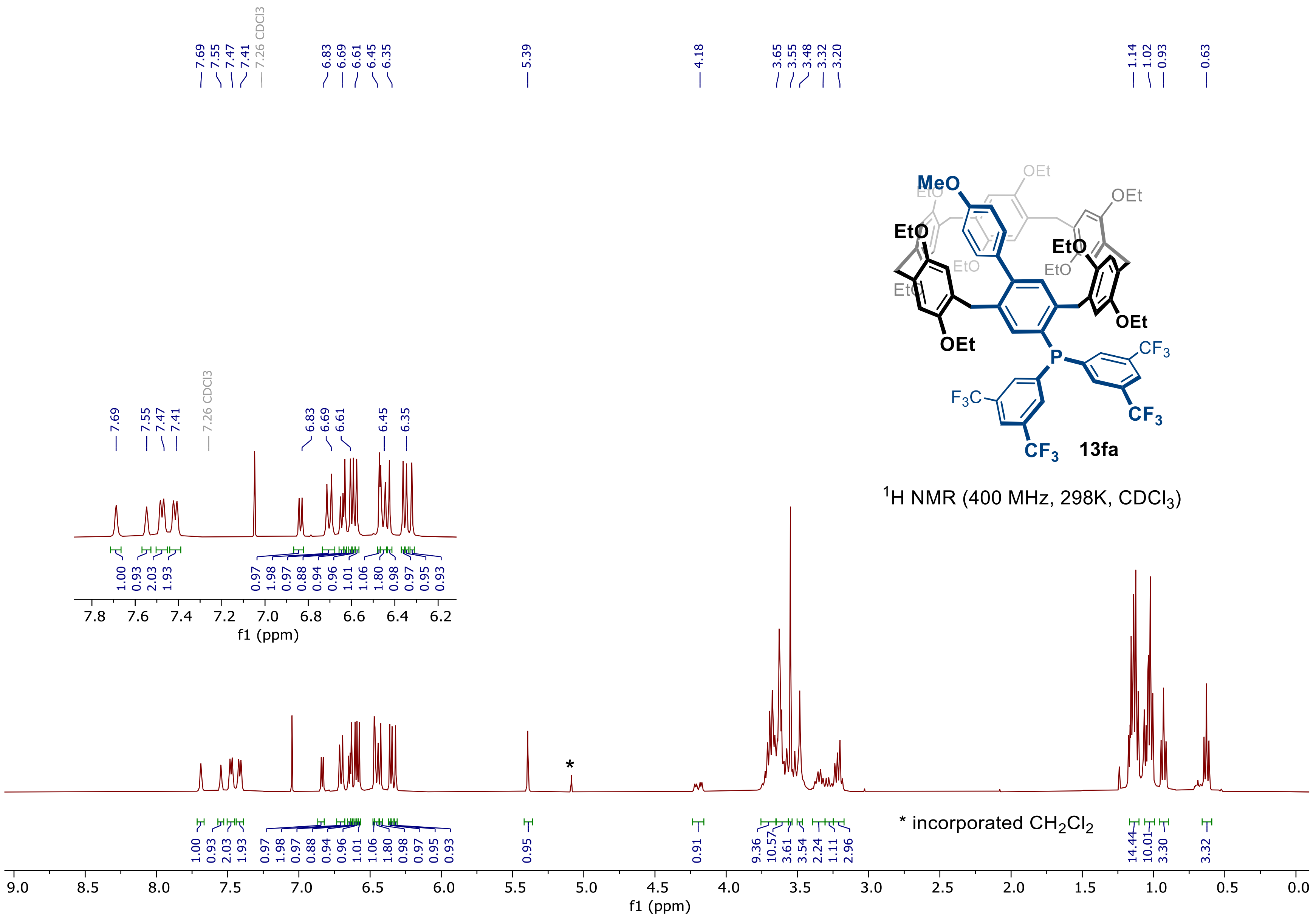

7.69 7.55 7.47 7.41 7.26 CDCl3 6.83 6.69 6.61 6.45 6.35 5.39 4.18 3.65 3.55 3.48 3.32 3.20 1.14 1.02 0.93 0.63
MeO
OEt
EtO
OEt
EtO
EtO
EtO
OEt
OEt
P
CF_3
F_3C
CF_3
CF_3
13fa
^{1}H NMR (400 MHz, 298K, $CDCl_3$)
* incorporated CH_2Cl_2
1.00 0.93 2.03 1.93 0.97 1.98 0.97 0.88 0.94 0.96 1.01 1.06 1.80 0.98 0.97 0.95 0.93 0.95 0.91 9.36 10.57 3.61 3.54 2.24 1.11 2.96 14.44 10.01 3.30 3.32
f1 (ppm)

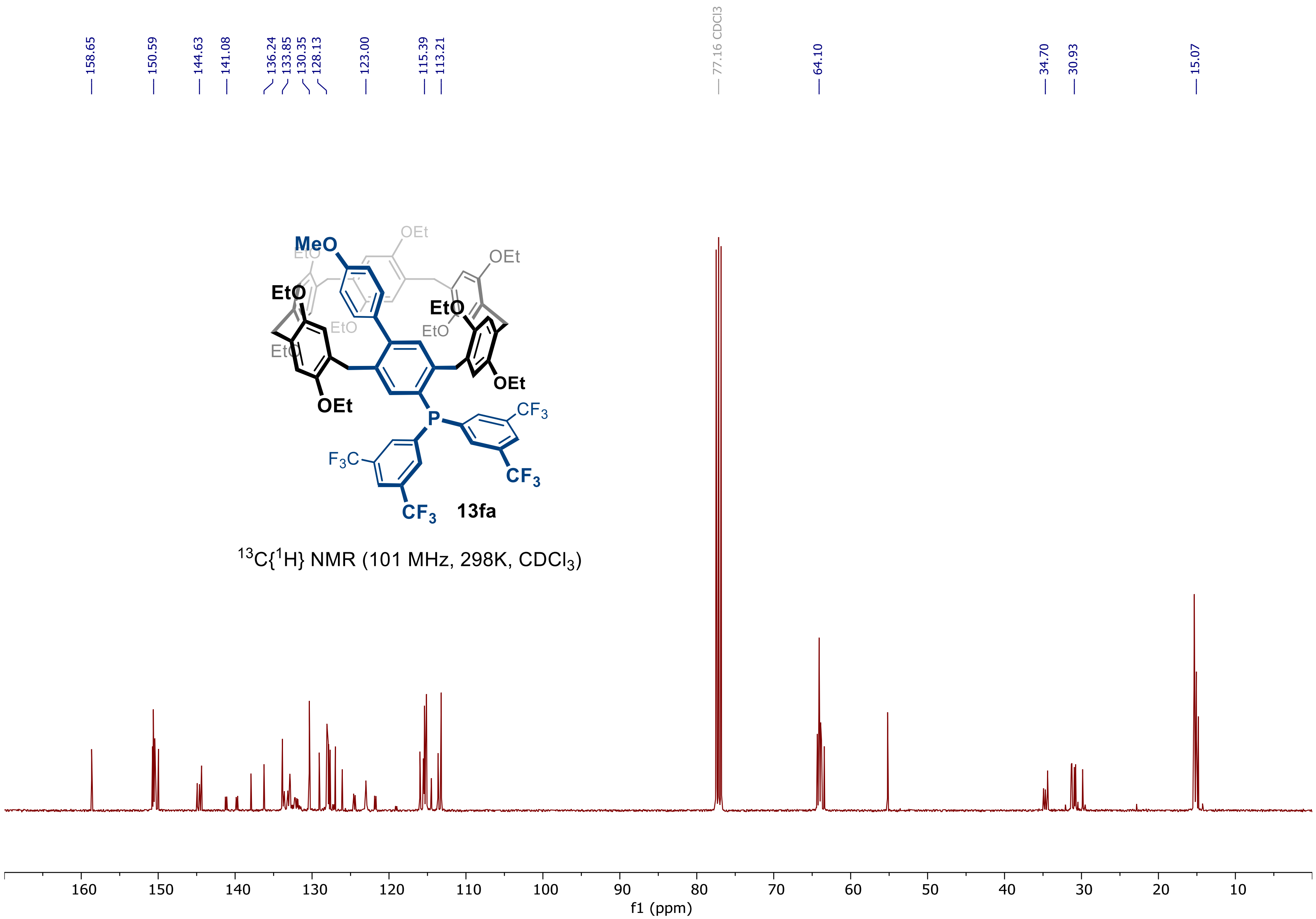
158.65
150.59
144.63
141.08
136.24
133.85
130.35
128.13
123.00
115.39
113.21
77.16 CDCl3
64.10
34.70
30.93
15.07
MeO
EtO
OEt
P
CF3
F3C
13fa
13C{1H} NMR (101 MHz, 298K, CDCl3)
f1 (ppm)

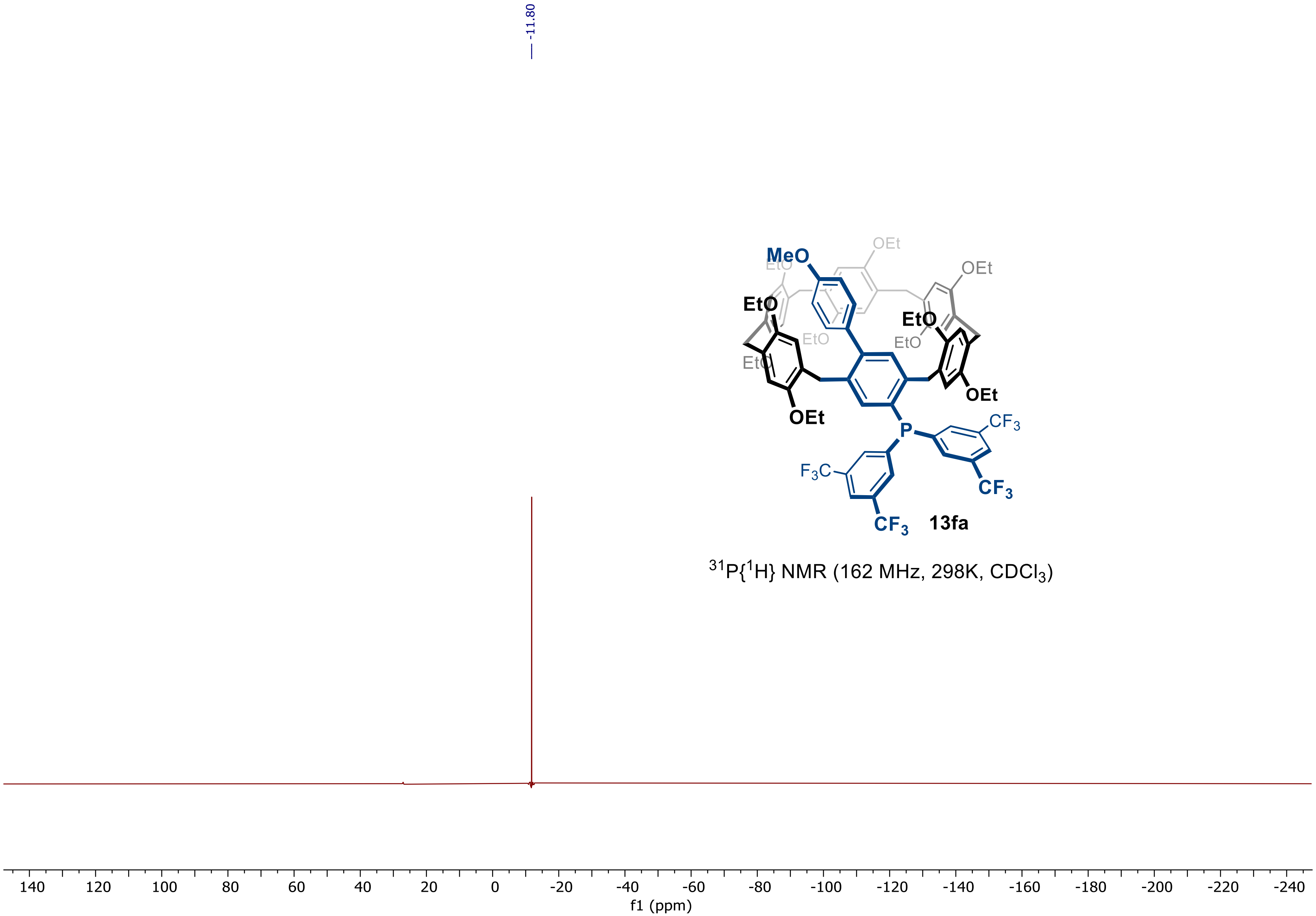

13fa

$^{31}P\{^{1}H\}$ NMR (162 MHz, 298K, $CDCl_3$)

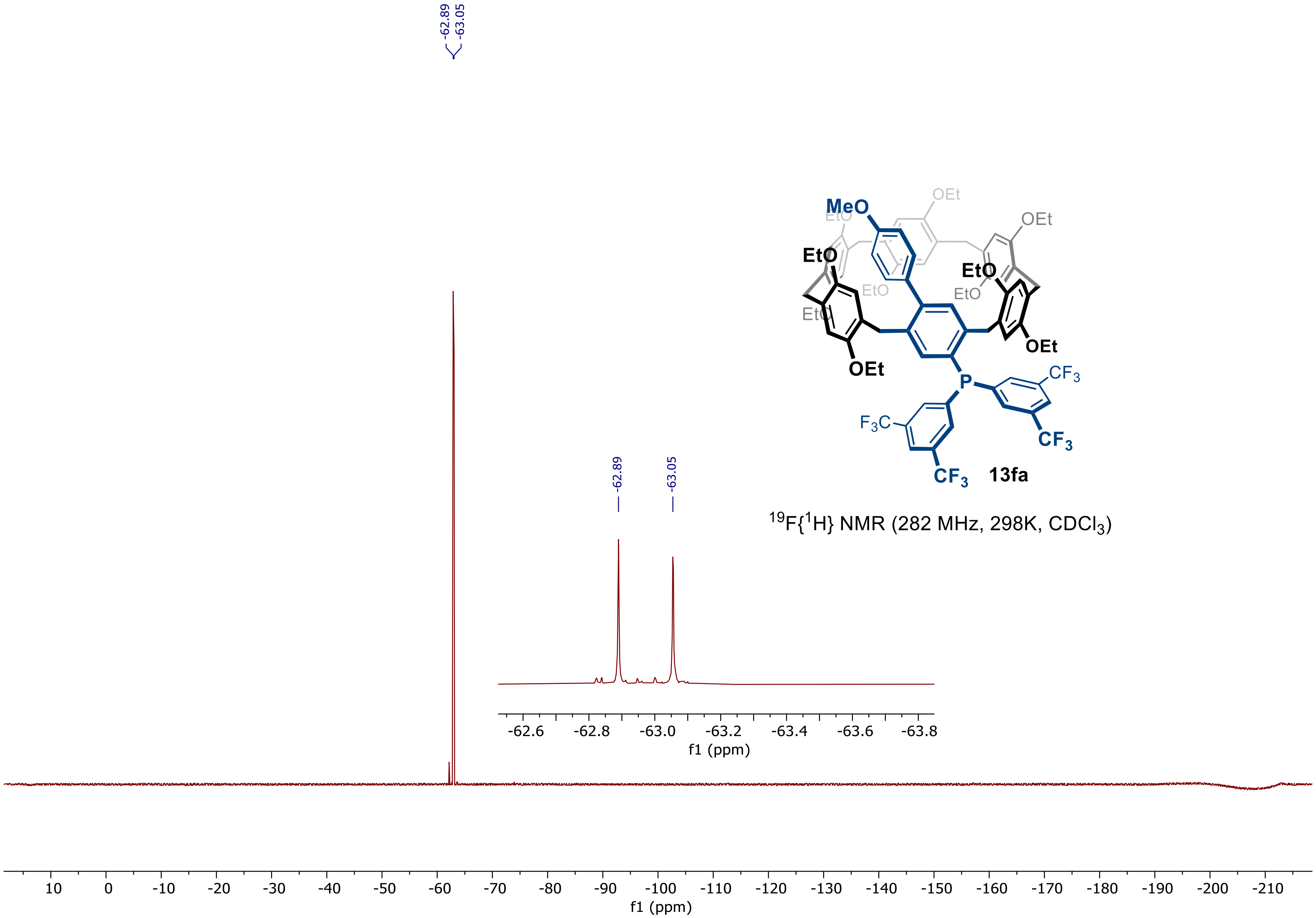

13fa

$^{19}F\{^{1}H\}$ NMR (282 MHz, 298K, $CDCl_3$)

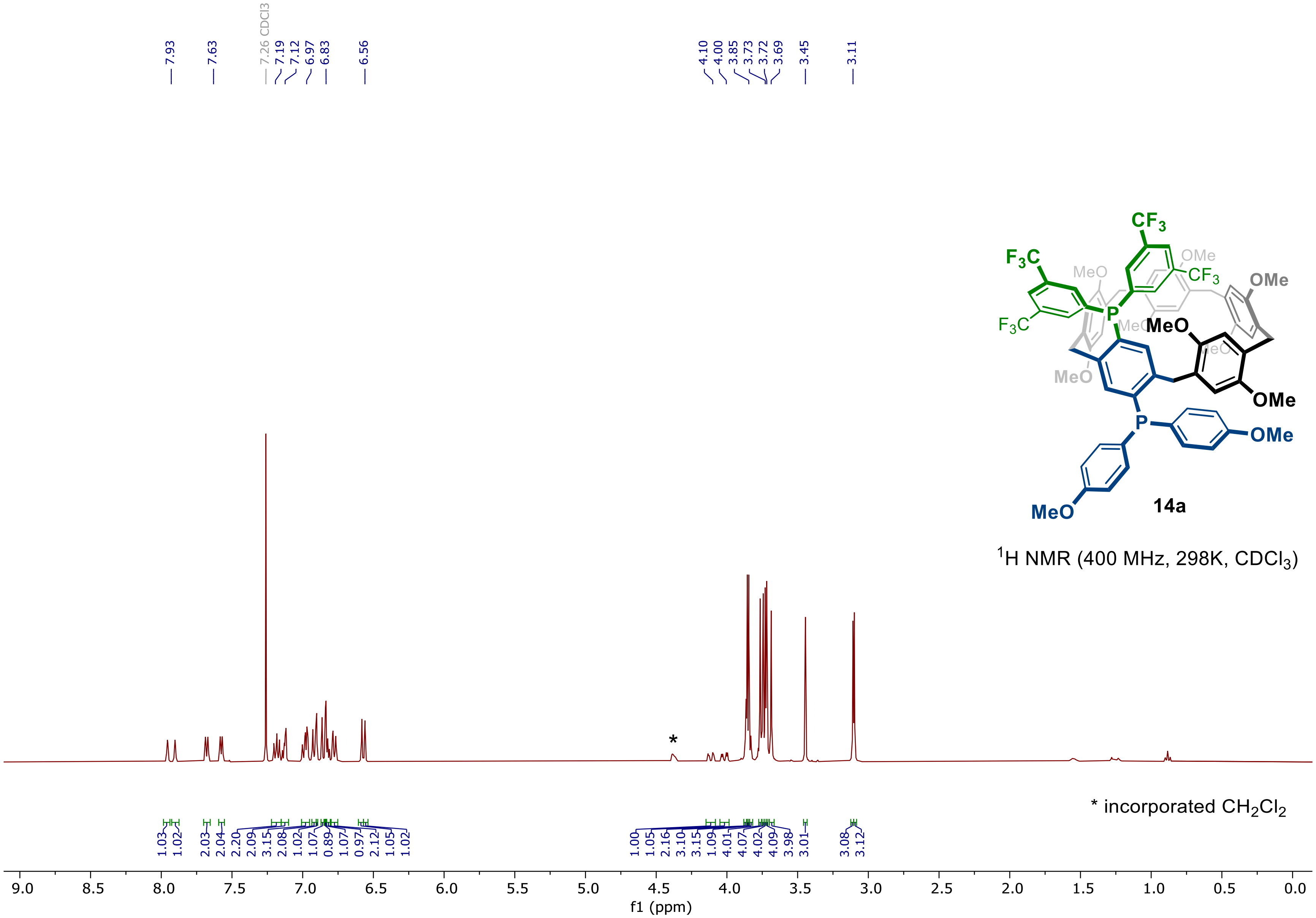
7.93
7.63
7.26 CDCl3
7.19
7.12
6.97
6.83
6.56
4.10
4.00
3.85
3.73
3.72
3.69
3.45
3.11
CF_3
F_3C
F_3C
CF_3
MeO
OMe
OMe
MeO
MeO
OMe
OMe
MeO
P
P
14a
^{1}H NMR (400 MHz, 298K, $CDCl_3$)
*
* incorporated CH_2Cl_2
1.03
1.02
2.03
2.04
2.20
2.09
3.15
2.08
1.02
1.07
0.89
1.07
0.97
2.12
1.05
1.02
1.00
1.05
2.16
3.10
3.15
1.09
4.01
4.07
4.02
4.09
3.98
3.01
3.08
3.12
9.0
8.5
8.0
7.5
7.0
6.5
6.0
5.5
5.0
4.5
4.0
3.5
3.0
2.5
2.0
1.5
1.0
0.5
0.0
f1 (ppm)

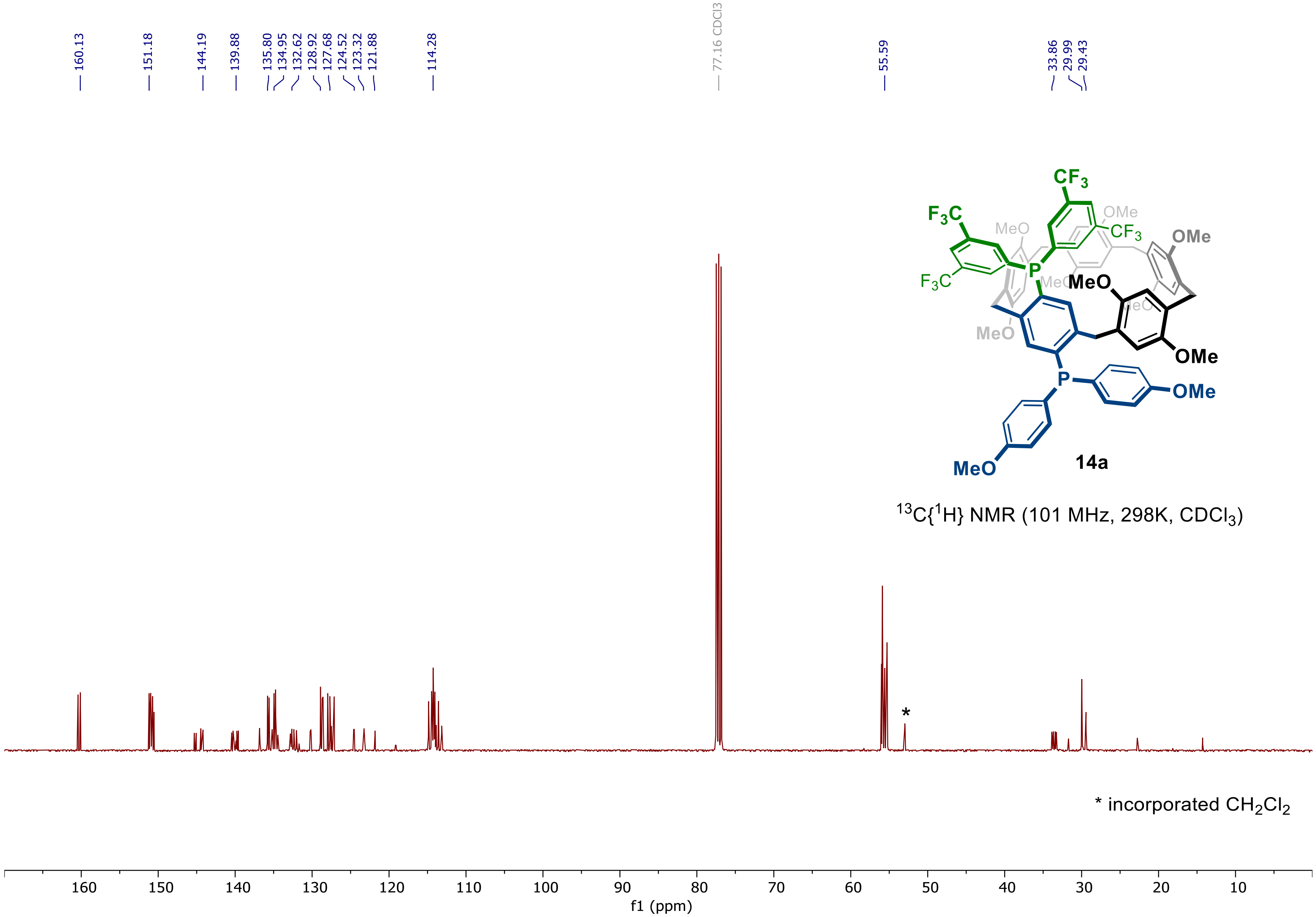
160.13
151.18
144.19
139.88
135.80
134.95
132.62
128.92
127.68
124.52
123.32
121.88
114.28
77.16 CDCl3
55.59
33.86
29.99
29.43
CF_3
F_3C
MeO
OMe
CF_3
OMe
F_3C
P
MeO
MeO
OMe
P
OMe
MeO
14a
$^{13}C\{^1H\}$ NMR (101 MHz, 298K, $CDCl_3$)
*
* incorporated CH_2Cl_2
160
150
140
130
120
110
100
90
80
70
60
50
40
30
20
10
f1 (ppm)

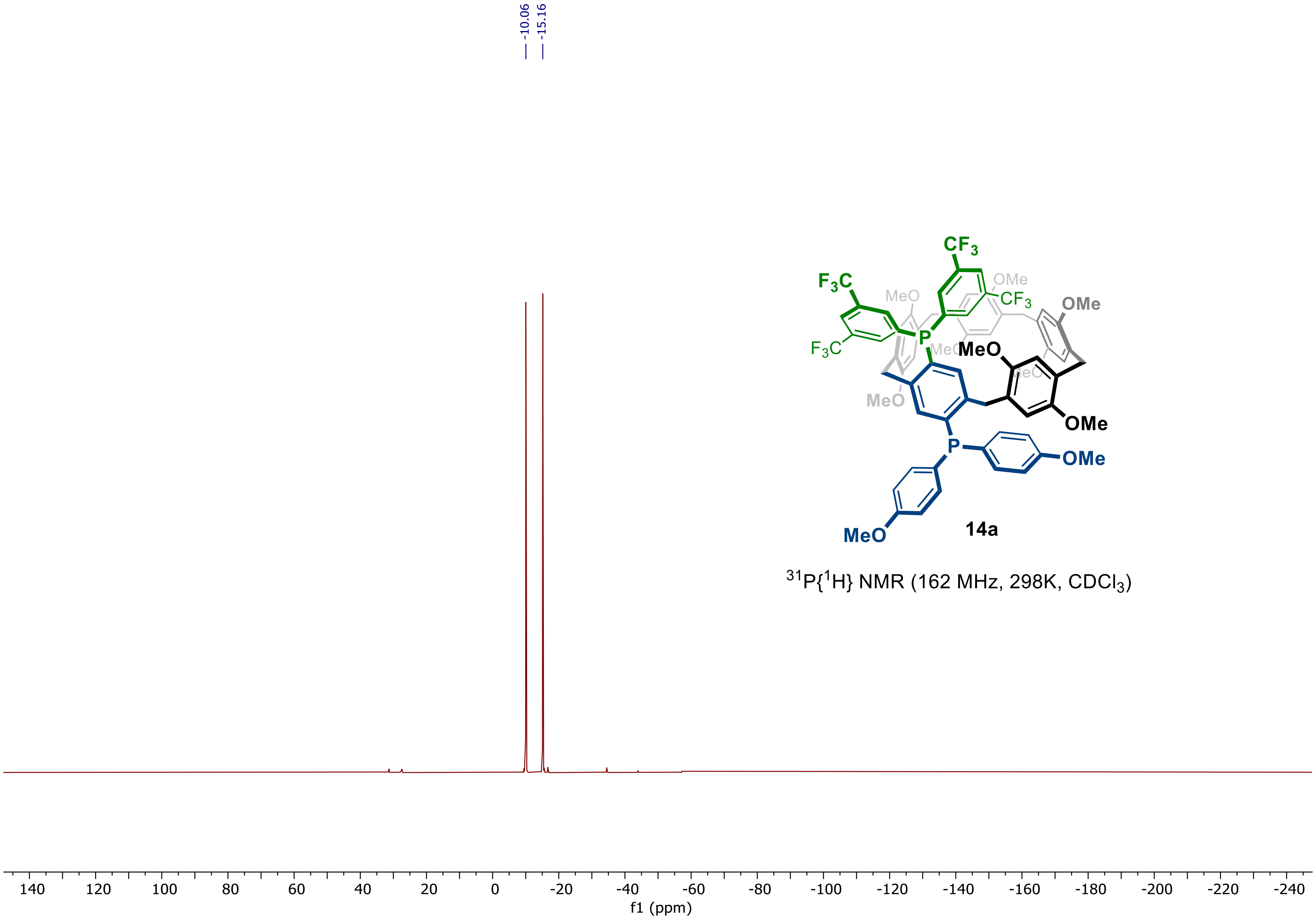

14a

$^{31}P\{^{1}H\}$ NMR (162 MHz, 298K, $CDCl_3$)

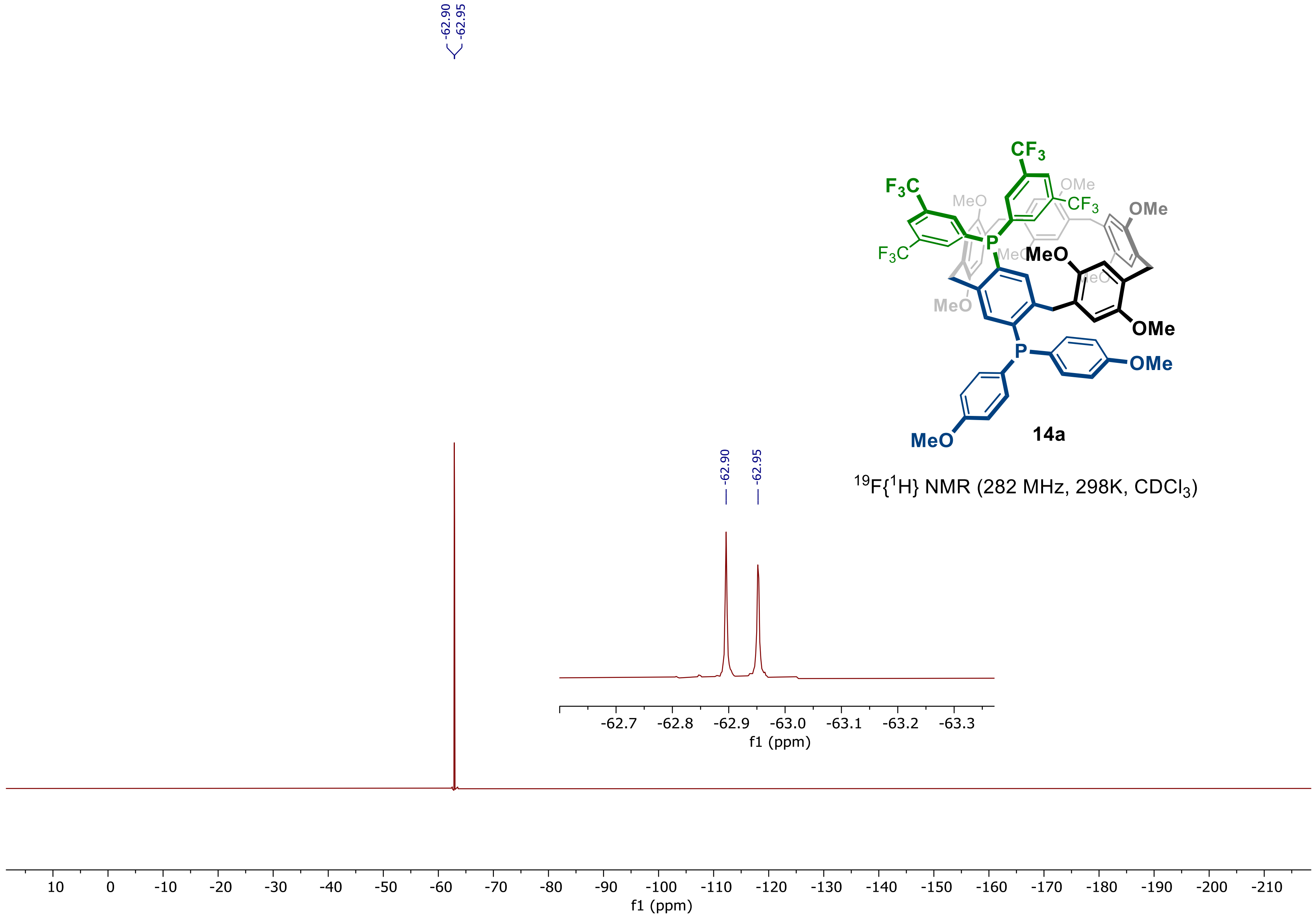
-62.90
-62.95
F3C
CF3
MeO
OMe
CF3
OMe
F3C
P
MeO
MeO
OMe
P
OMe
MeO
14a
19F{1H} NMR (282 MHz, 298K, CDCl3)
-62.90
-62.95
-62.7
-62.8
-62.9
-63.0
-63.1
-63.2
-63.3
f1 (ppm)
10
0
-10
-20
-30
-40
-50
-60
-70
-80
-90
-100
-110
-120
-130
-140
-150
-160
-170
-180
-190
-200
-210
f1 (ppm)

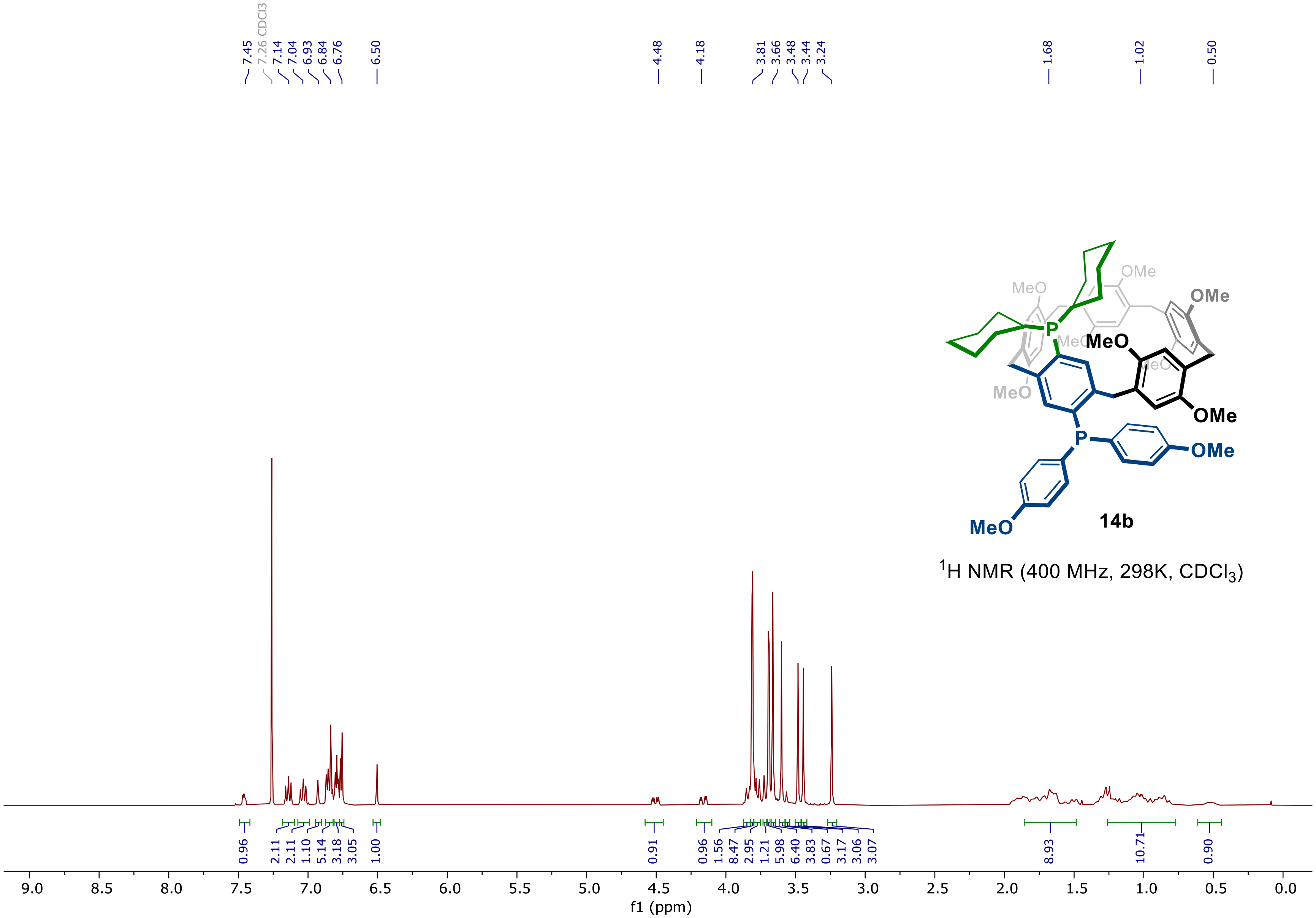

14b

^{1}H NMR (400 MHz, 298K, $CDCl_3$)

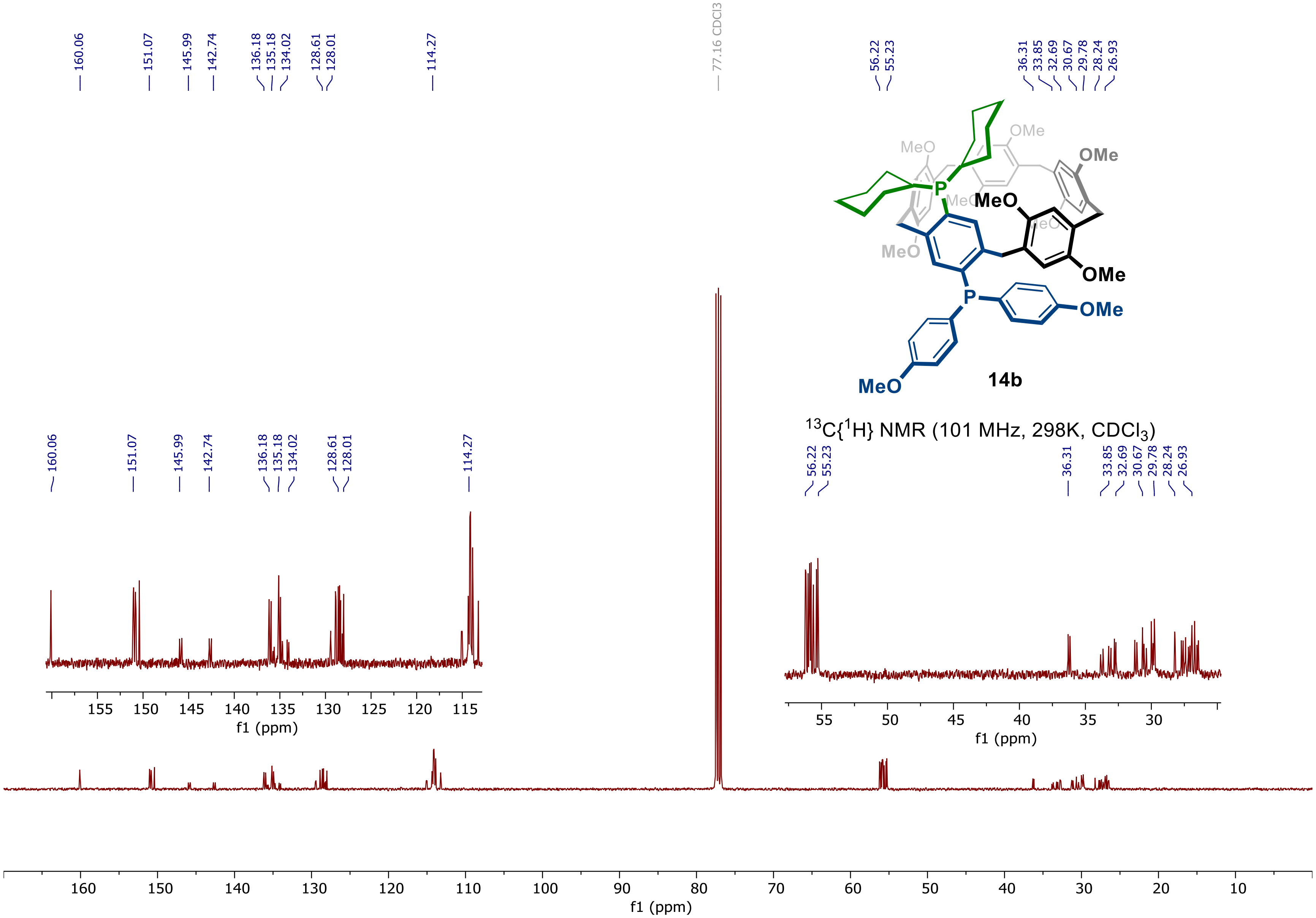
160.06
151.07
145.99
142.74
136.18
135.18
134.02
128.61
128.01
114.27
77.16 CDCl3
56.22
55.23
36.31
33.85
32.69
30.67
29.78
28.24
26.93
MeO
OMe
OMe
P
MeO
MeO
OMe
P
OMe
MeO
14b
$^{13}C\{^1H\}$ NMR (101 MHz, 298K, $CDCl_3$)
f1 (ppm)

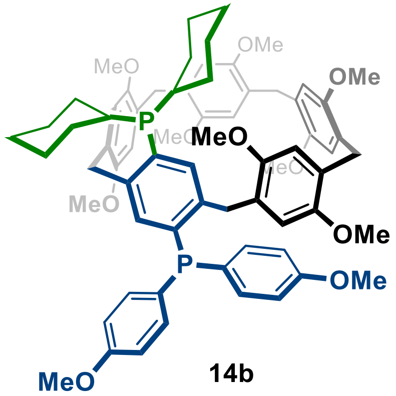

14b

$^{31}P\{^1H\}$ NMR (162 MHz, 298K, $CDCl_3$)

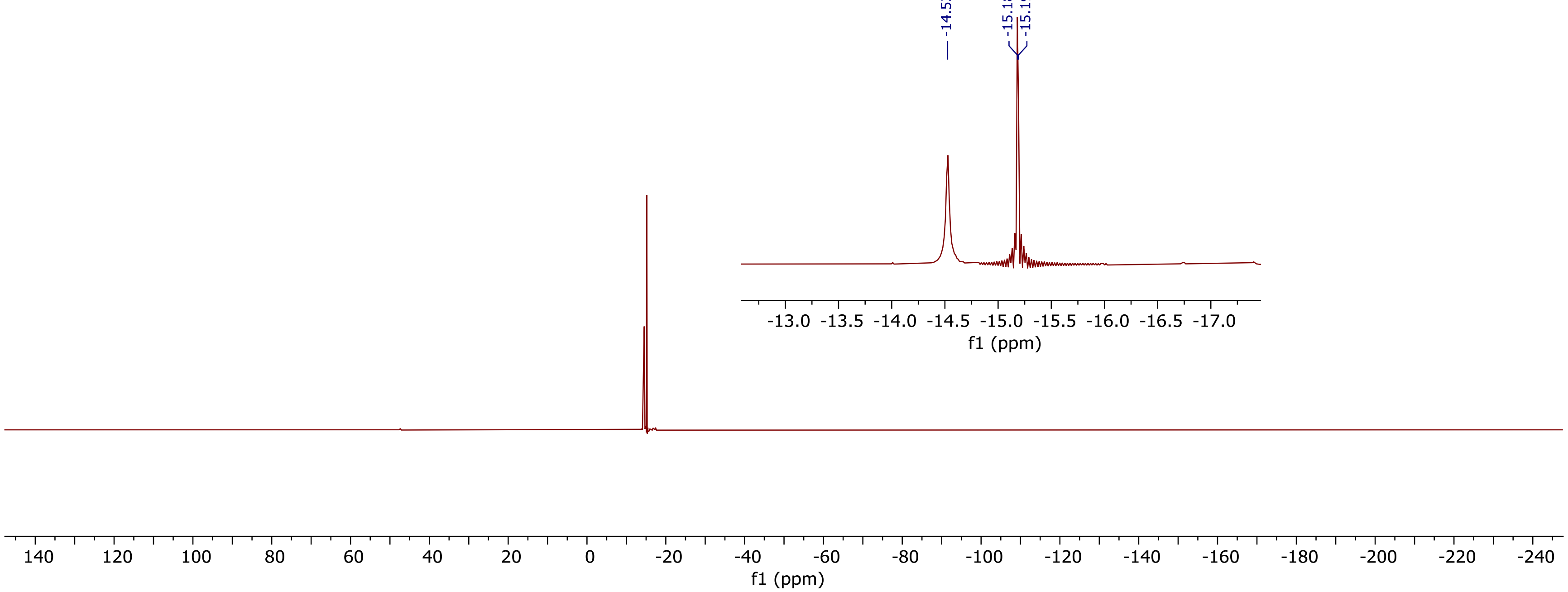

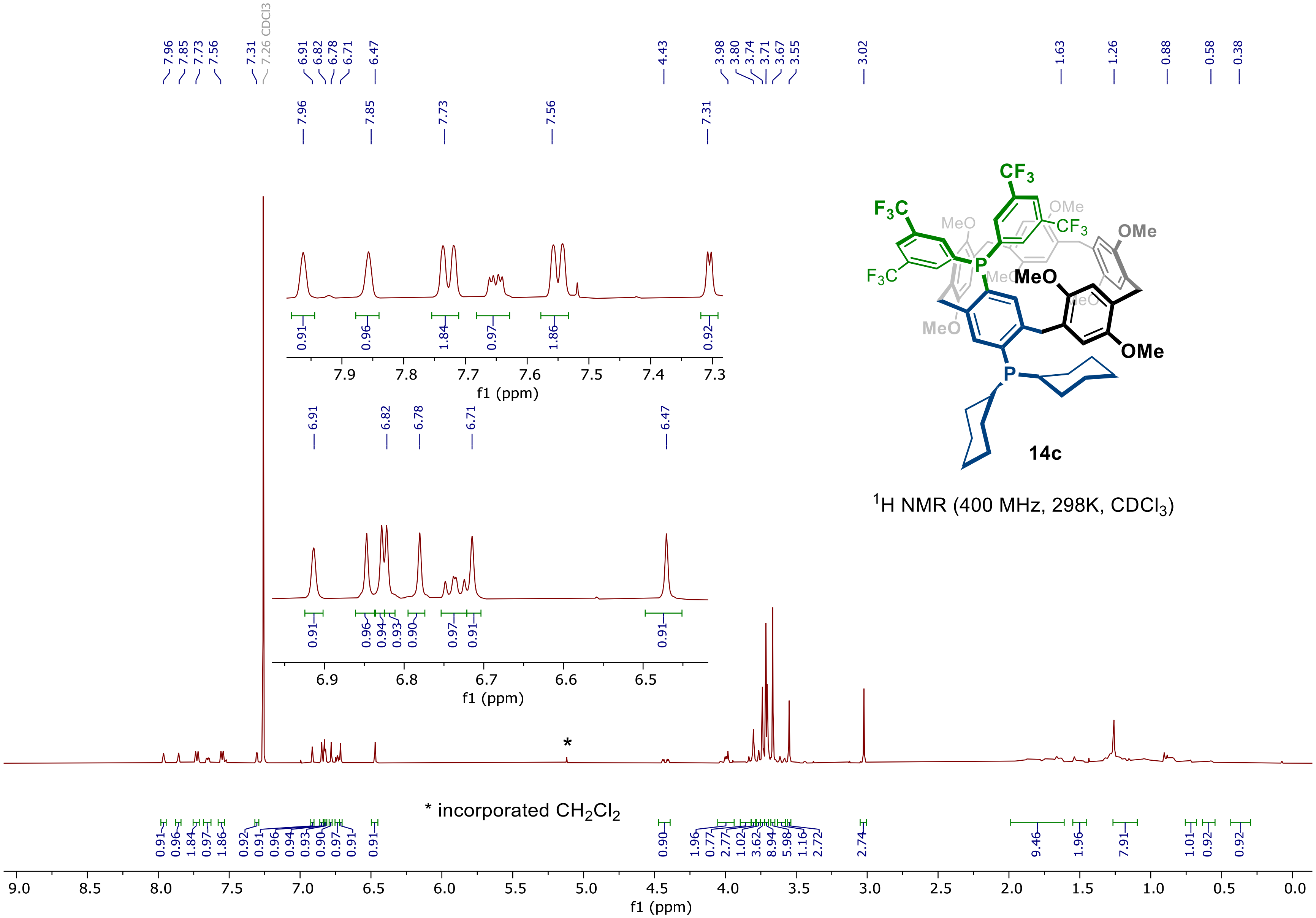

^{1}H NMR (400 MHz, 298K, $CDCl_3$)

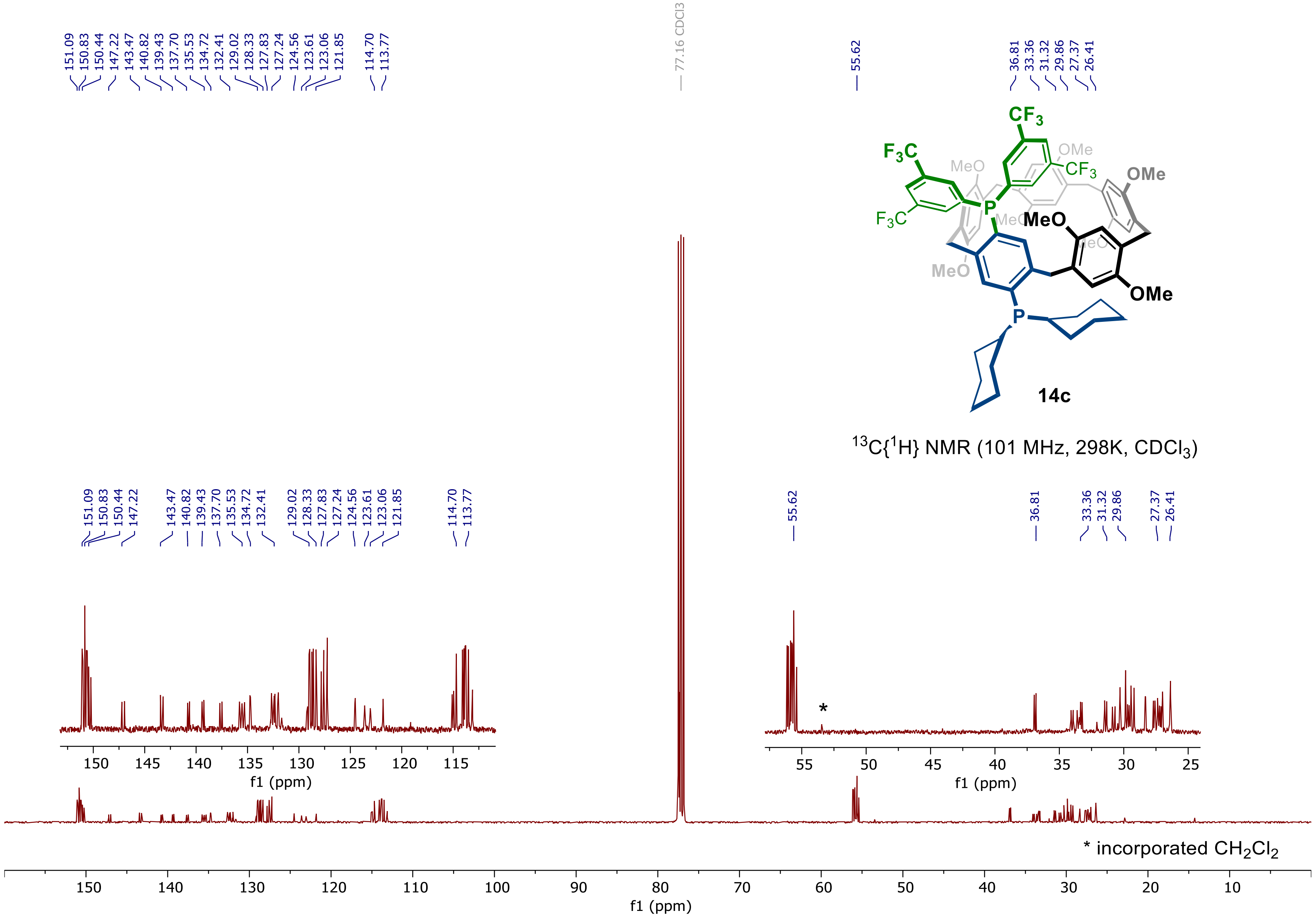
151.09
150.83
150.44
147.22
143.47
140.82
139.43
137.70
135.53
134.72
132.41
129.02
128.33
127.83
127.24
124.56
123.61
123.06
121.85
114.70
113.77
77.16 CDCl3
55.62
36.81
33.36
31.32
29.86
27.37
26.41
CF3
F3C
F3C
CF3
MeO
OMe
OMe
MeO
MeO
OMe
P
P
14c
13C{1H} NMR (101 MHz, 298K, CDCl3)
f1 (ppm)
* incorporated CH2Cl2

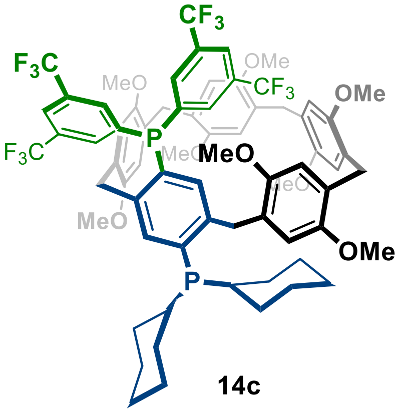

14c

$^{31}P\{^{1}H\}$ NMR (162 MHz, 298K, $CDCl_3$)

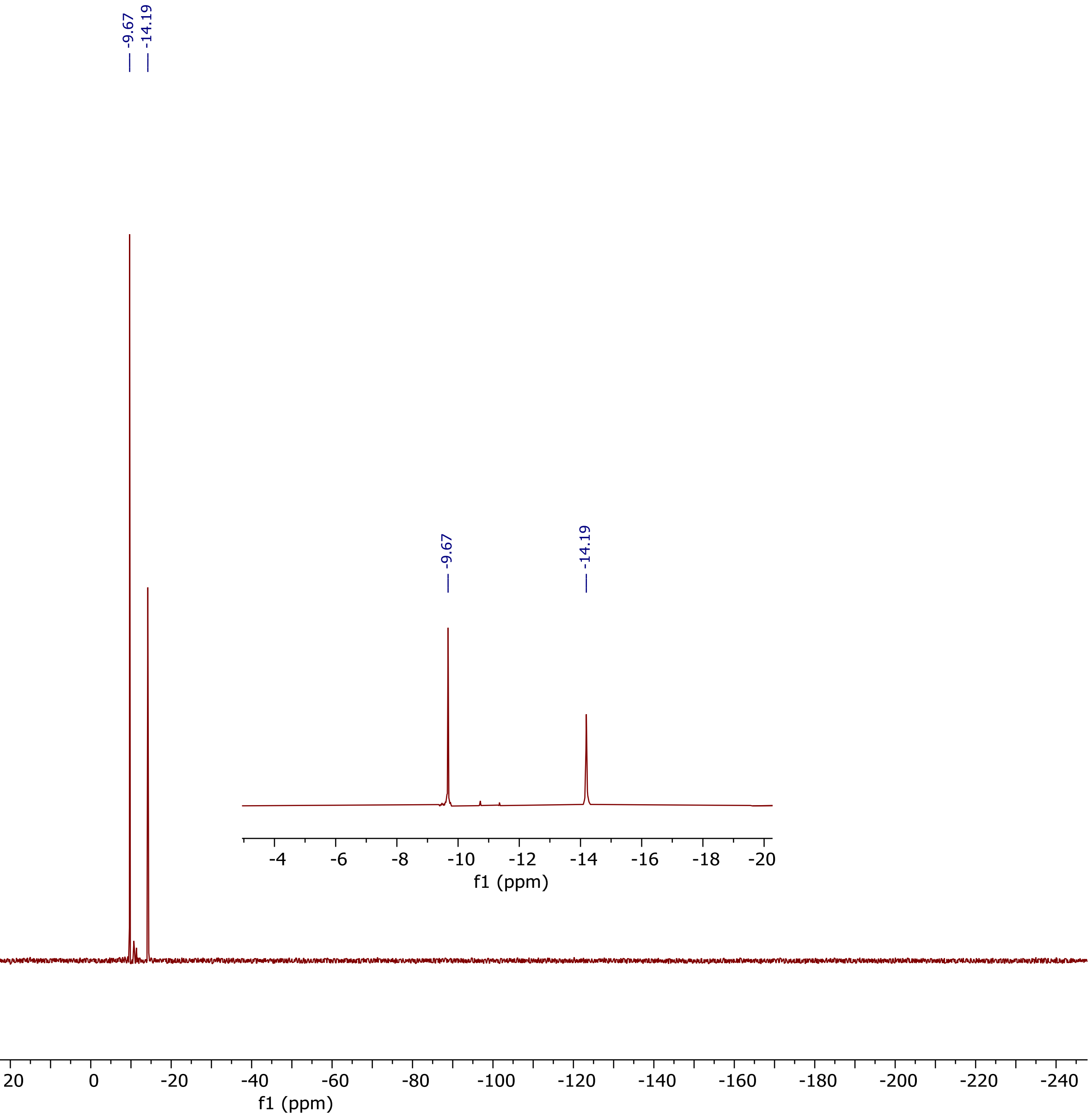

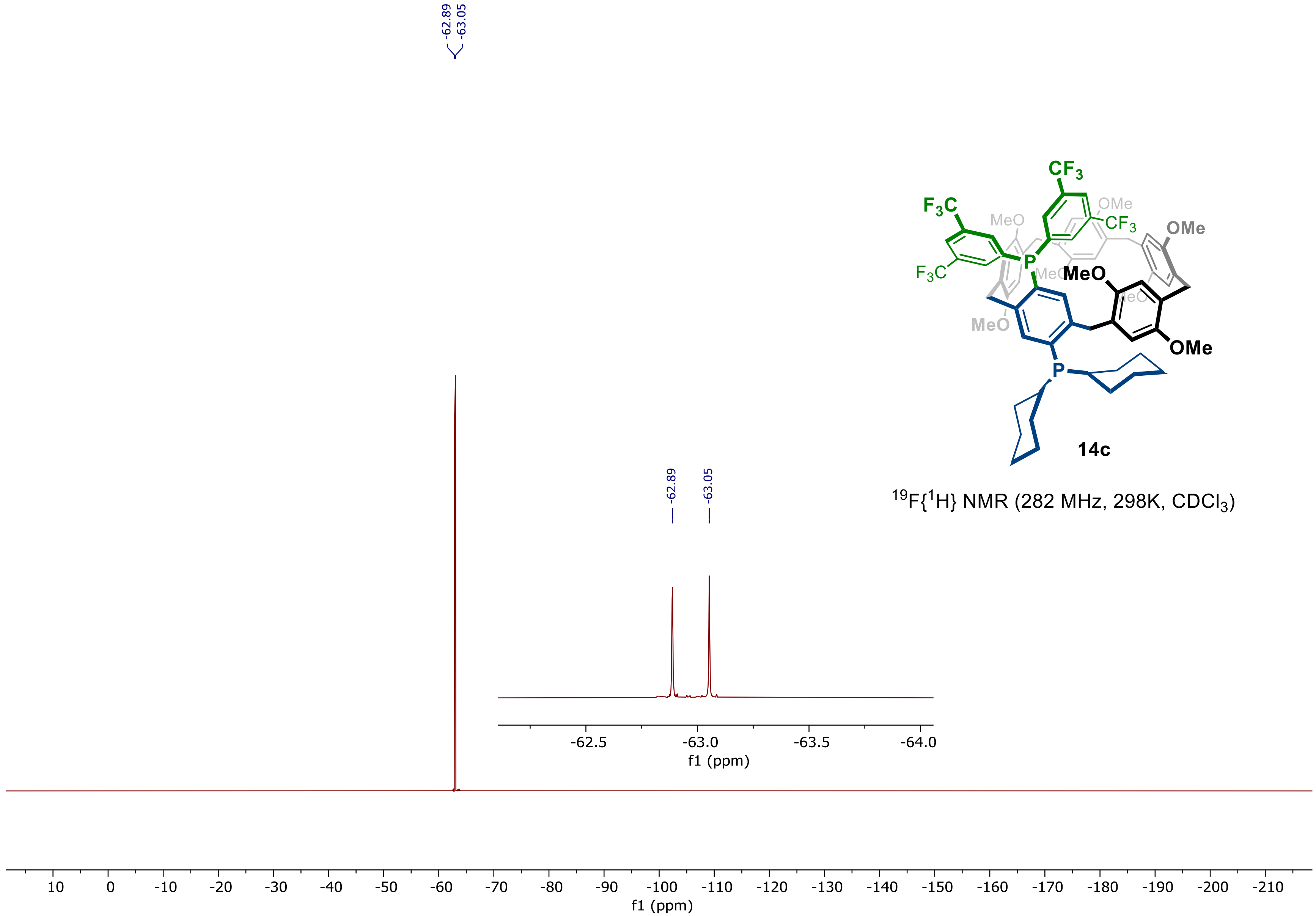

14c

$^{19}F\{^{1}H\}$ NMR (282 MHz, 298K, $CDCl_3$)

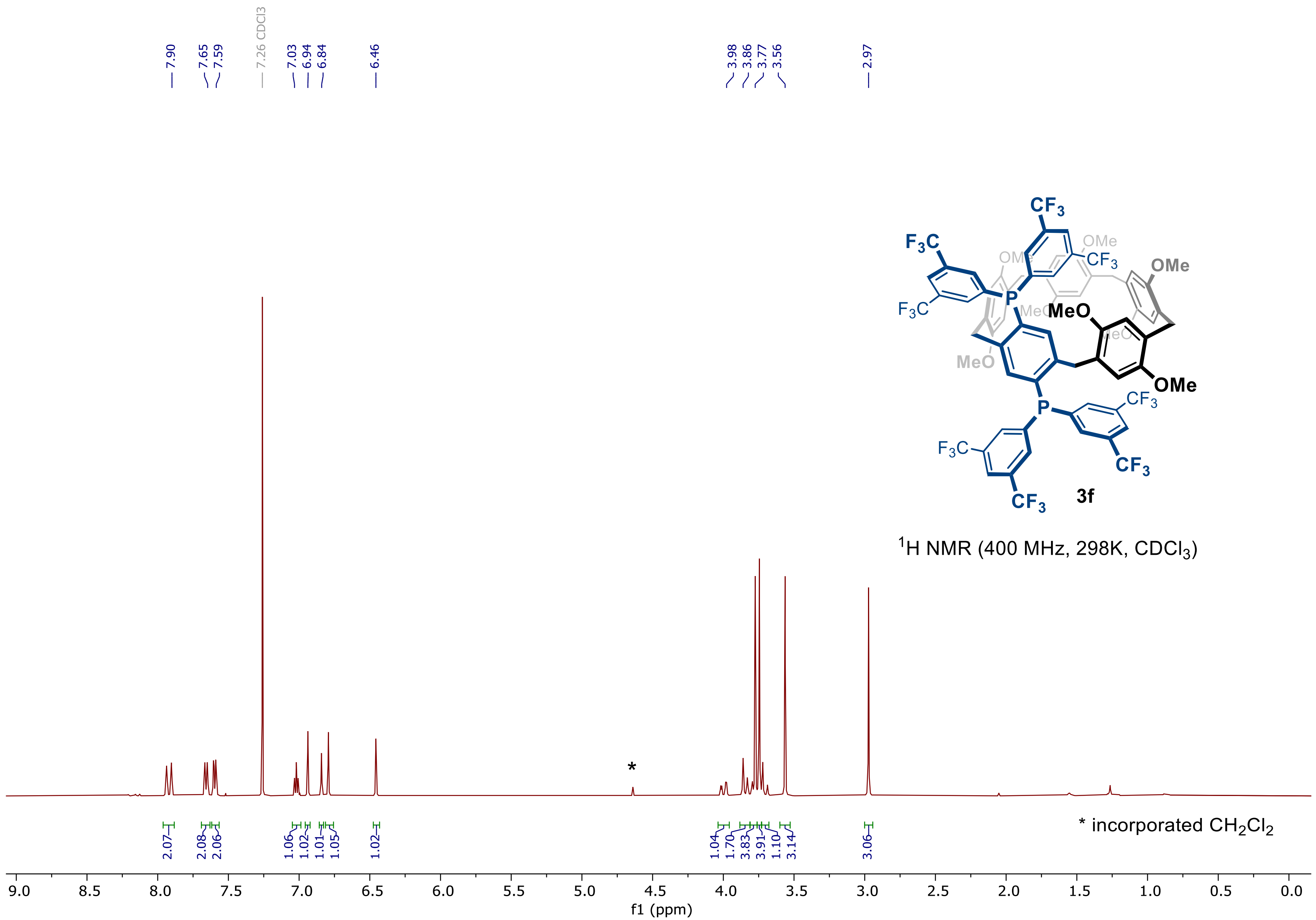
7.90
7.65
7.59
7.26 CDCl3
7.03
6.94
6.84
6.46
3.98
3.86
3.77
3.56
2.97
F3C
CF3
OMe
3f
^{1}H NMR (400 MHz, 298K, CDCl$_3$)
*
* incorporated CH$_2$Cl$_2$
2.07
2.08
2.06
1.06
1.02
1.01
1.05
1.02
1.04
1.70
3.83
3.91
1.10
3.14
3.06
9.0
8.5
8.0
7.5
7.0
6.5
6.0
5.5
5.0
4.5
4.0
3.5
3.0
2.5
2.0
1.5
1.0
0.5
0.0
f1 (ppm)

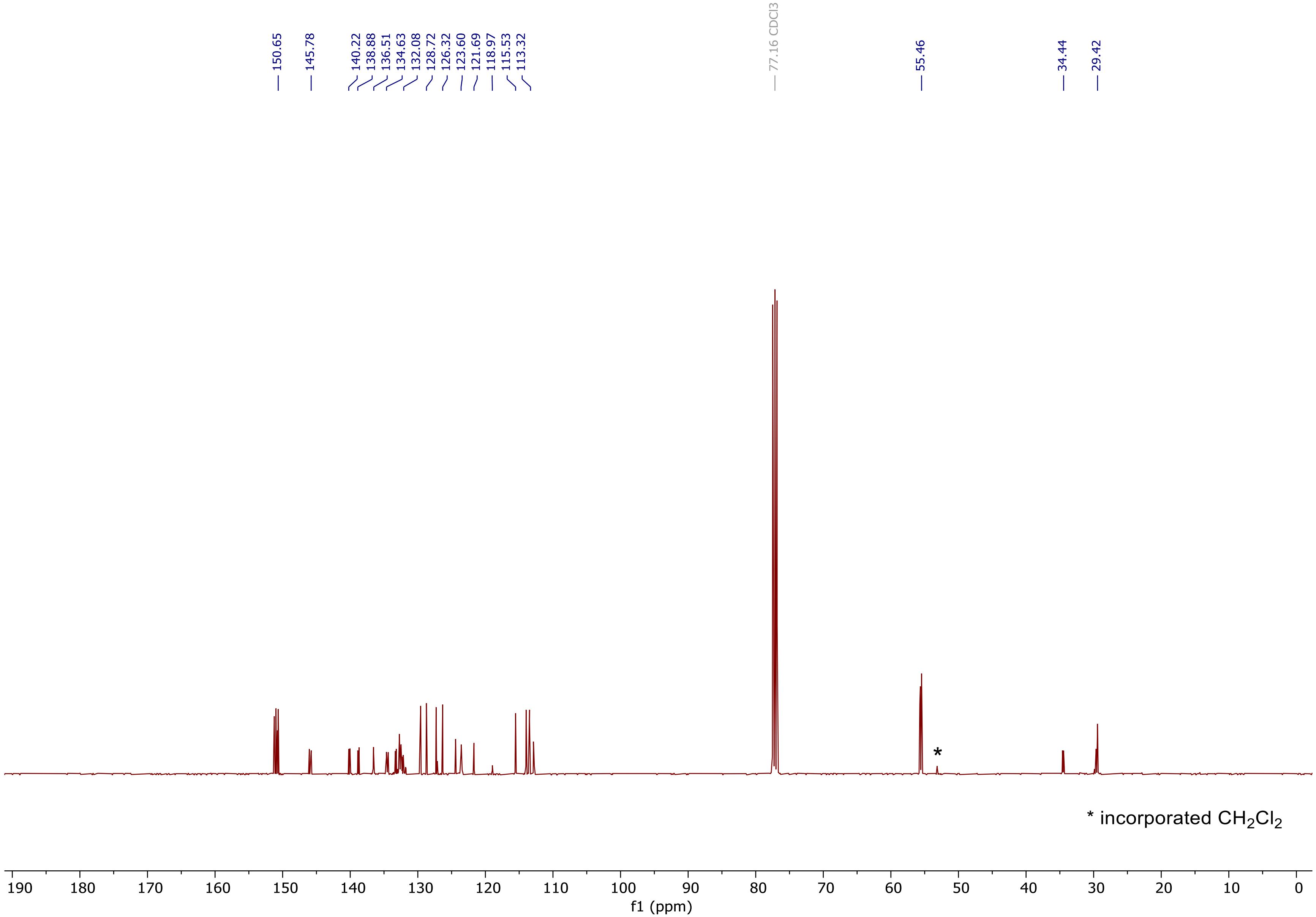
150.65
145.78
140.22
138.88
136.51
134.63
132.08
128.72
126.32
123.60
121.69
118.97
115.53
113.32
77.16 CDCl3
55.46
34.44
29.42
*
* incorporated CH_2Cl_2
190 180 170 160 150 140 130 120 110 100 90 80 70 60 50 40 30 20 10 0
f1 (ppm)

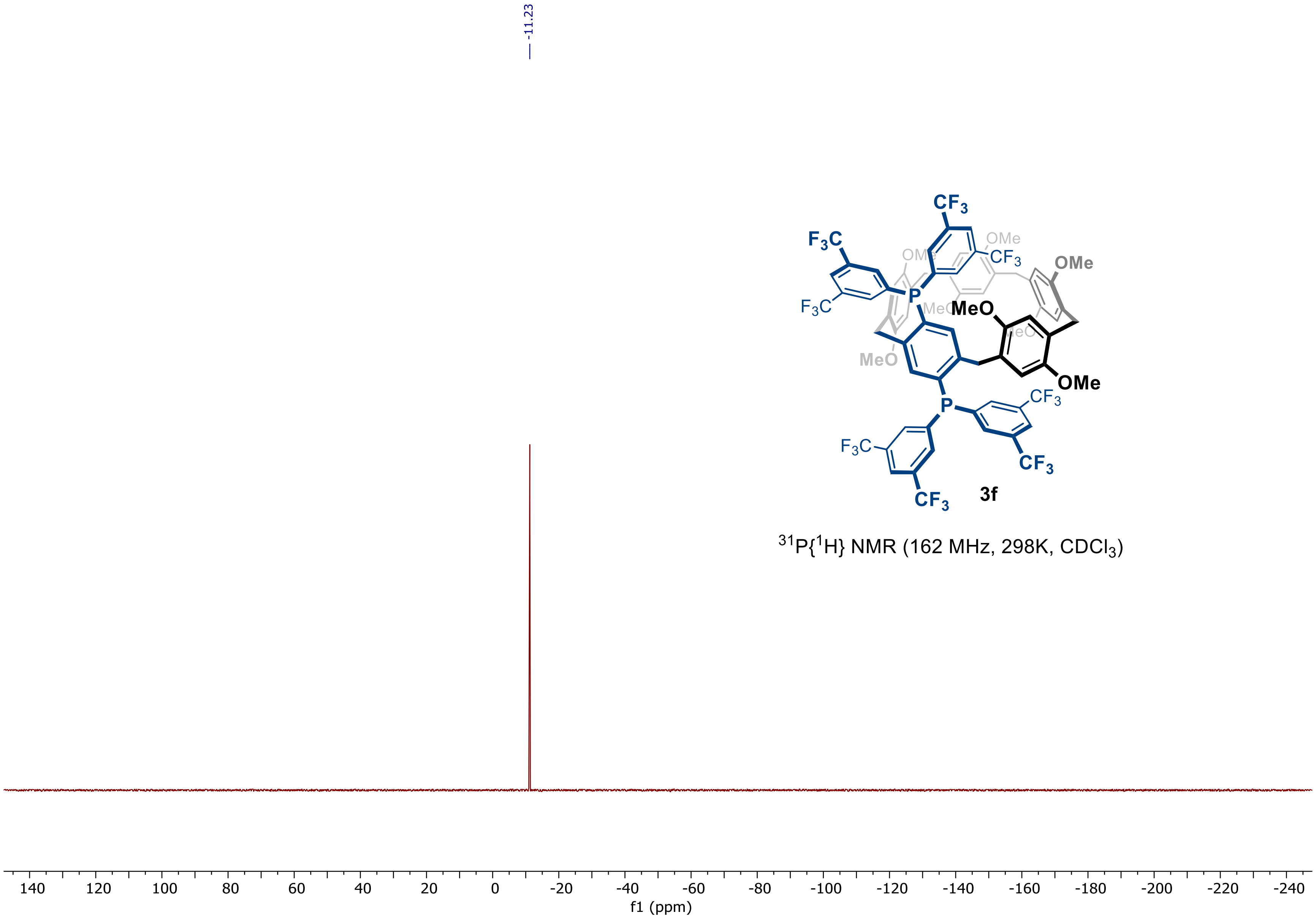

3f

$^{31}P\{^{1}H\}$ NMR (162 MHz, 298K, $CDCl_3$)

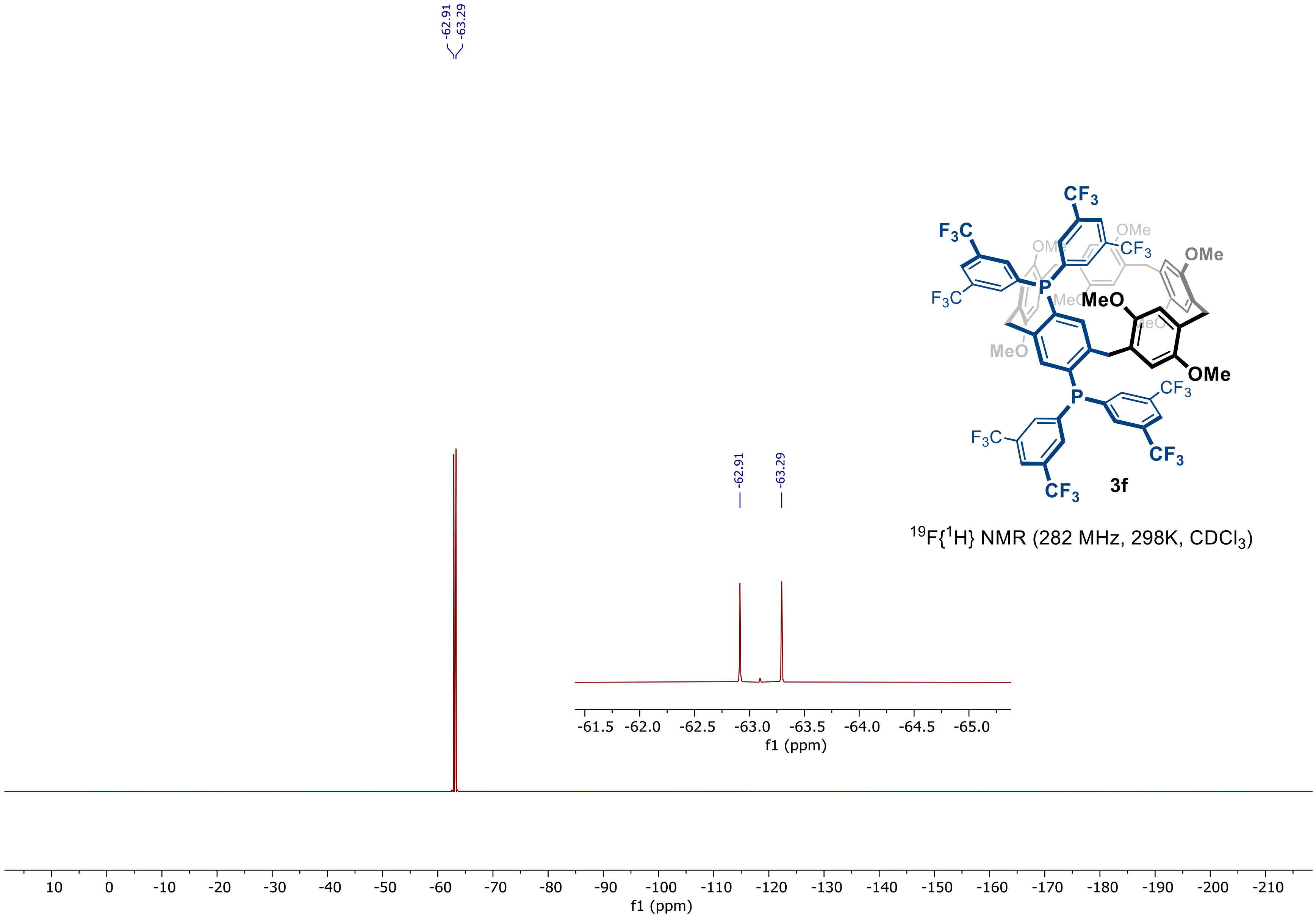

3f

$^{19}F\{^{1}H\}$ NMR (282 MHz, 298K, $CDCl_3$)

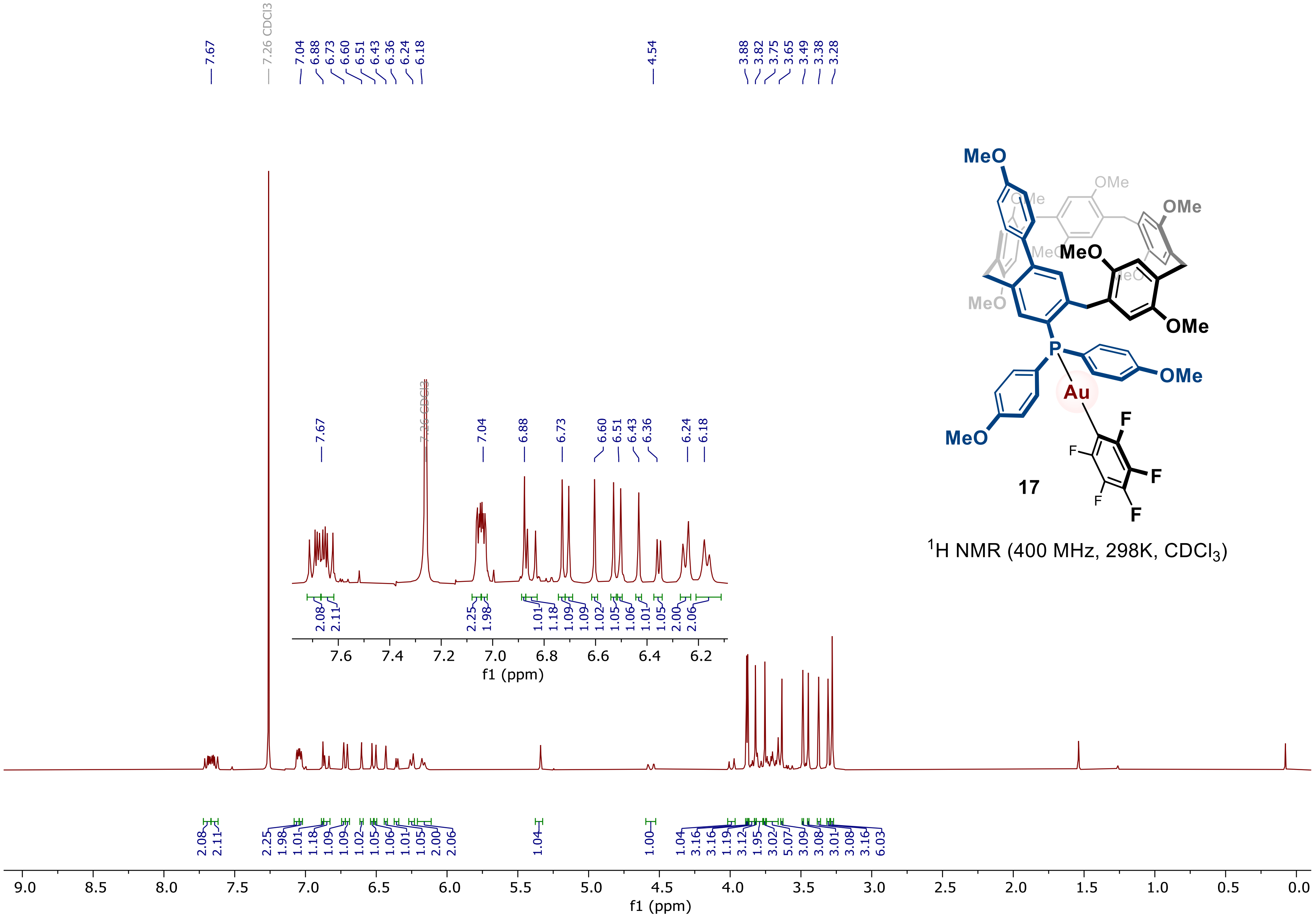
7.67
7.26 CDCl3
7.04
6.88
6.73
6.60
6.51
6.43
6.36
6.24
6.18
4.54
3.88
3.82
3.75
3.65
3.49
3.38
3.28
MeO
OMe
MeO
OMe
MeO
P
Au
OMe
MeO
F
F
F
F
F
17
1H NMR (400 MHz, 298K, CDCl3)
f1 (ppm)

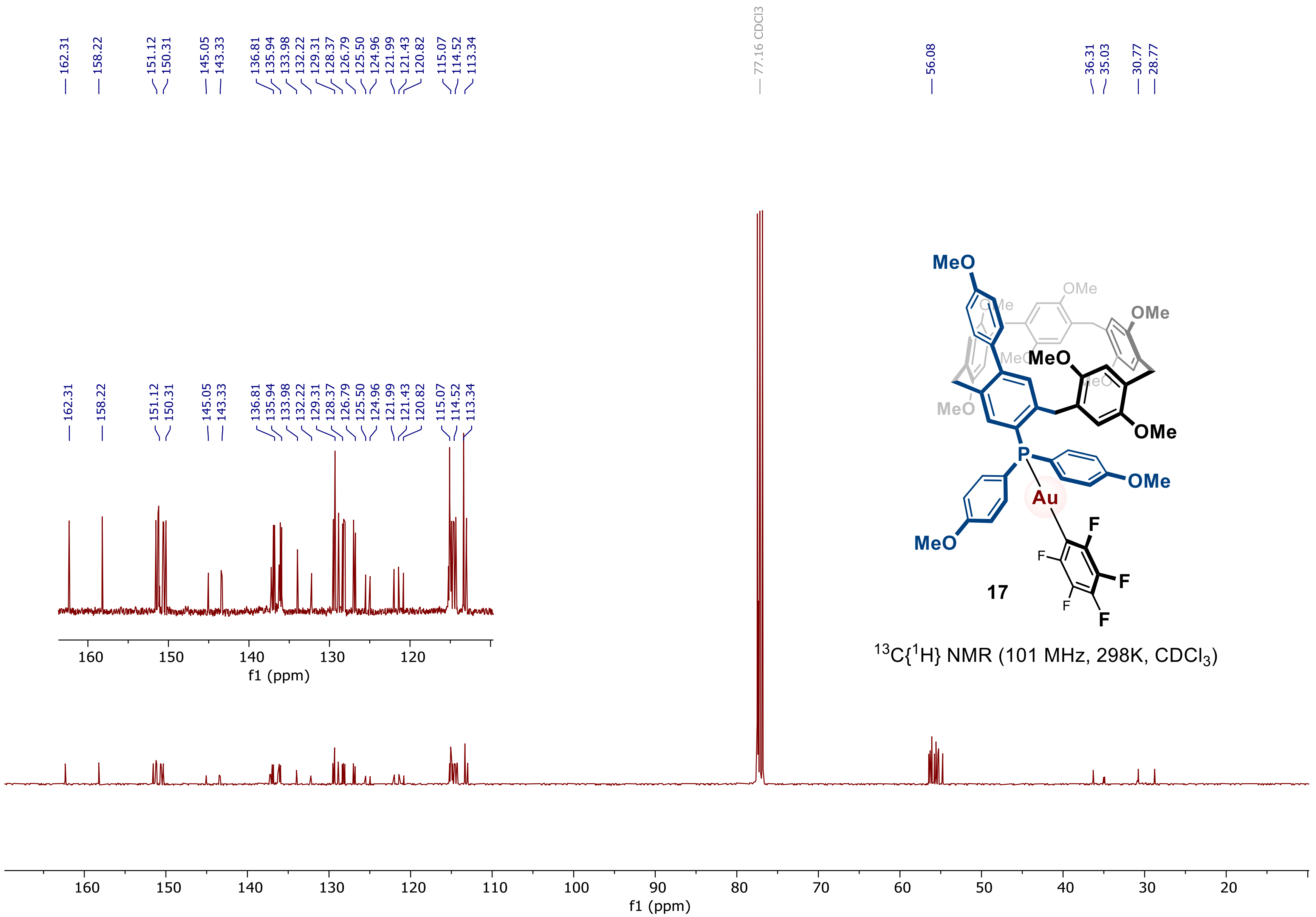

$^{13}C\{^{1}H\}$ NMR (101 MHz, 298K, $CDCl_3$)

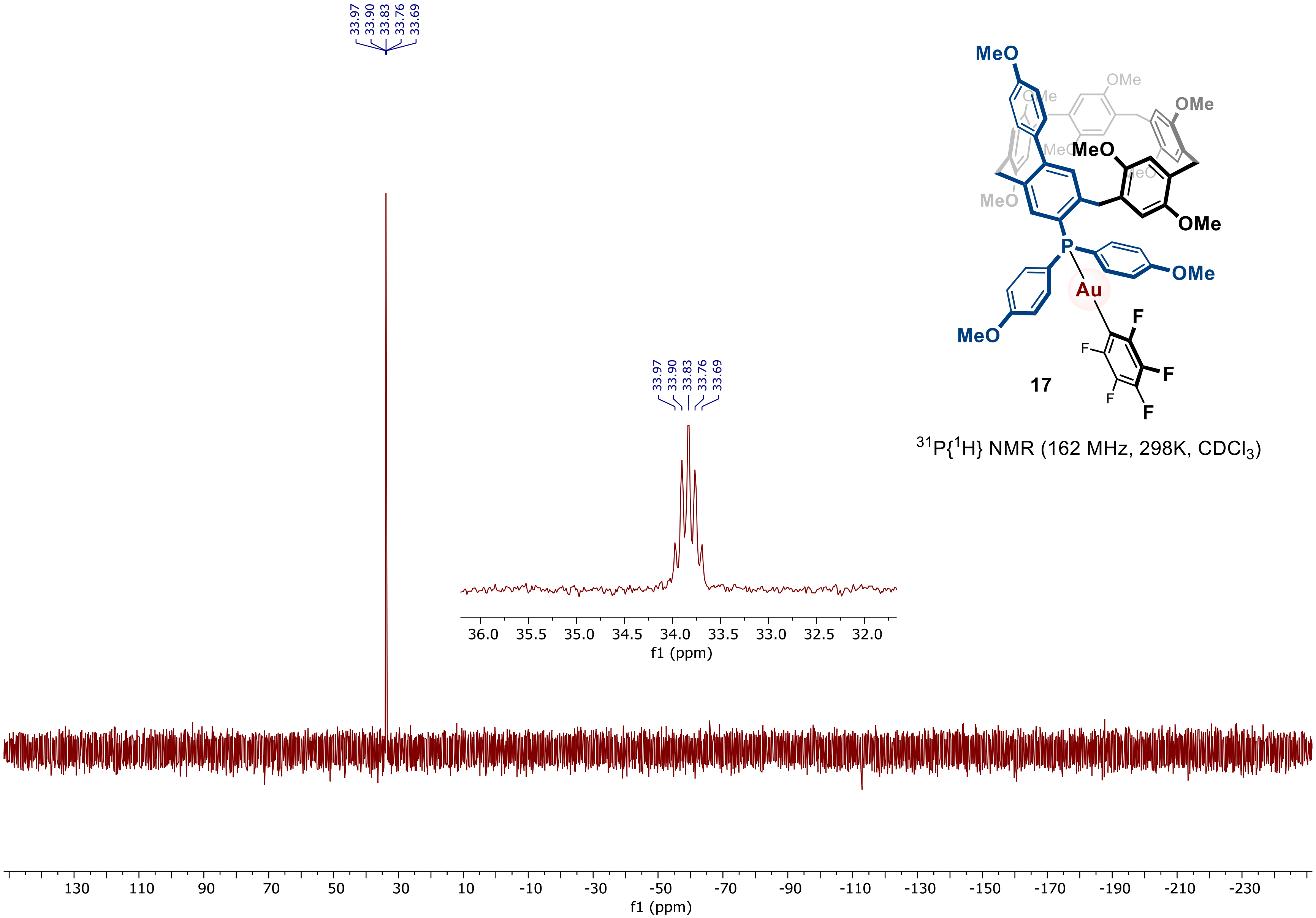

17

$^{31}P\{^{1}H\}$ NMR (162 MHz, 298K, $CDCl_3$)

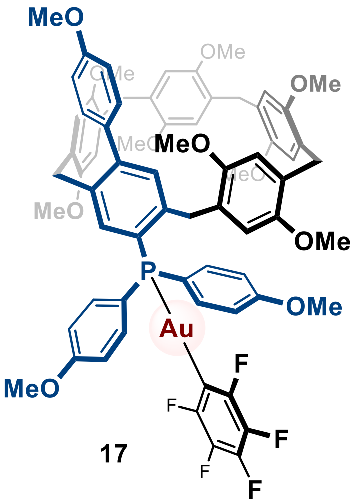

17

$^{19}F\{^{1}H\}$ NMR (282 MHz, 298K, $CDCl_3$)

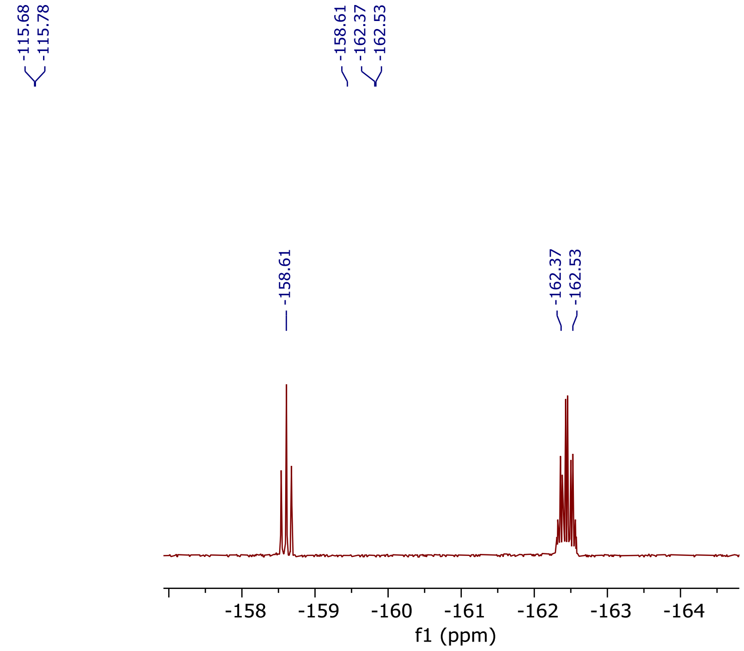

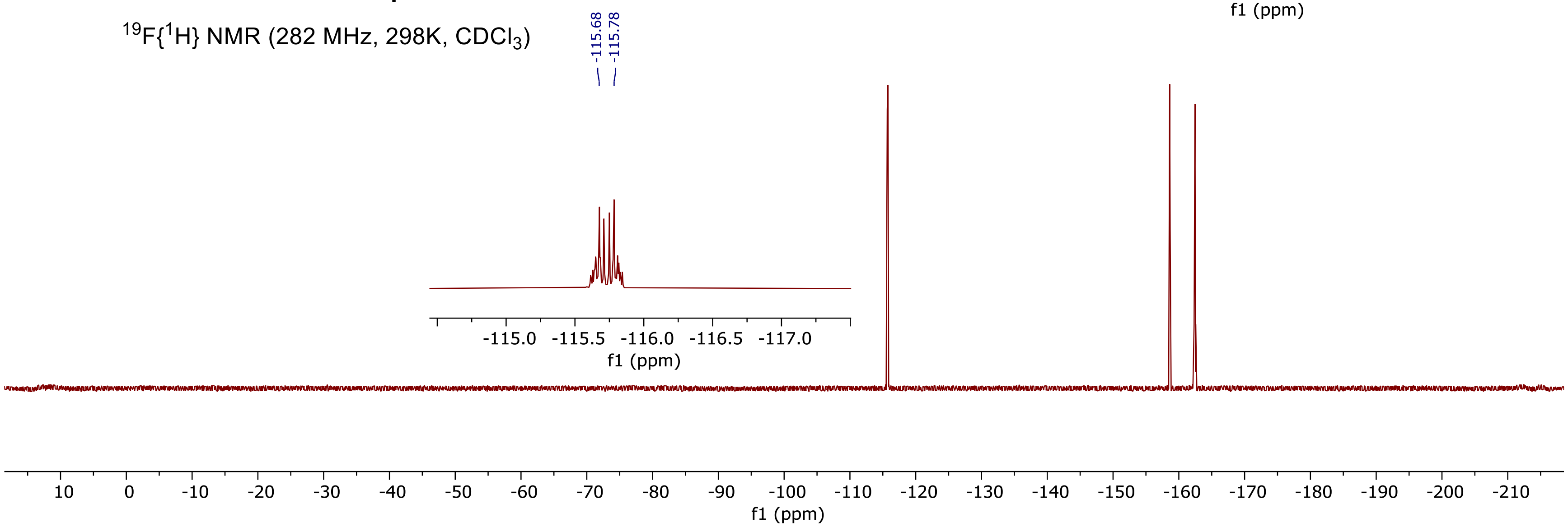

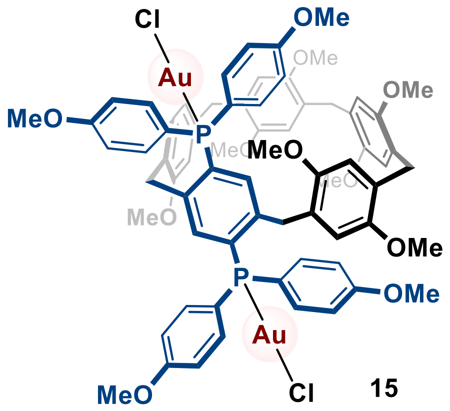

15

^{1}H NMR (400 MHz, 298K, CD_2Cl_2)

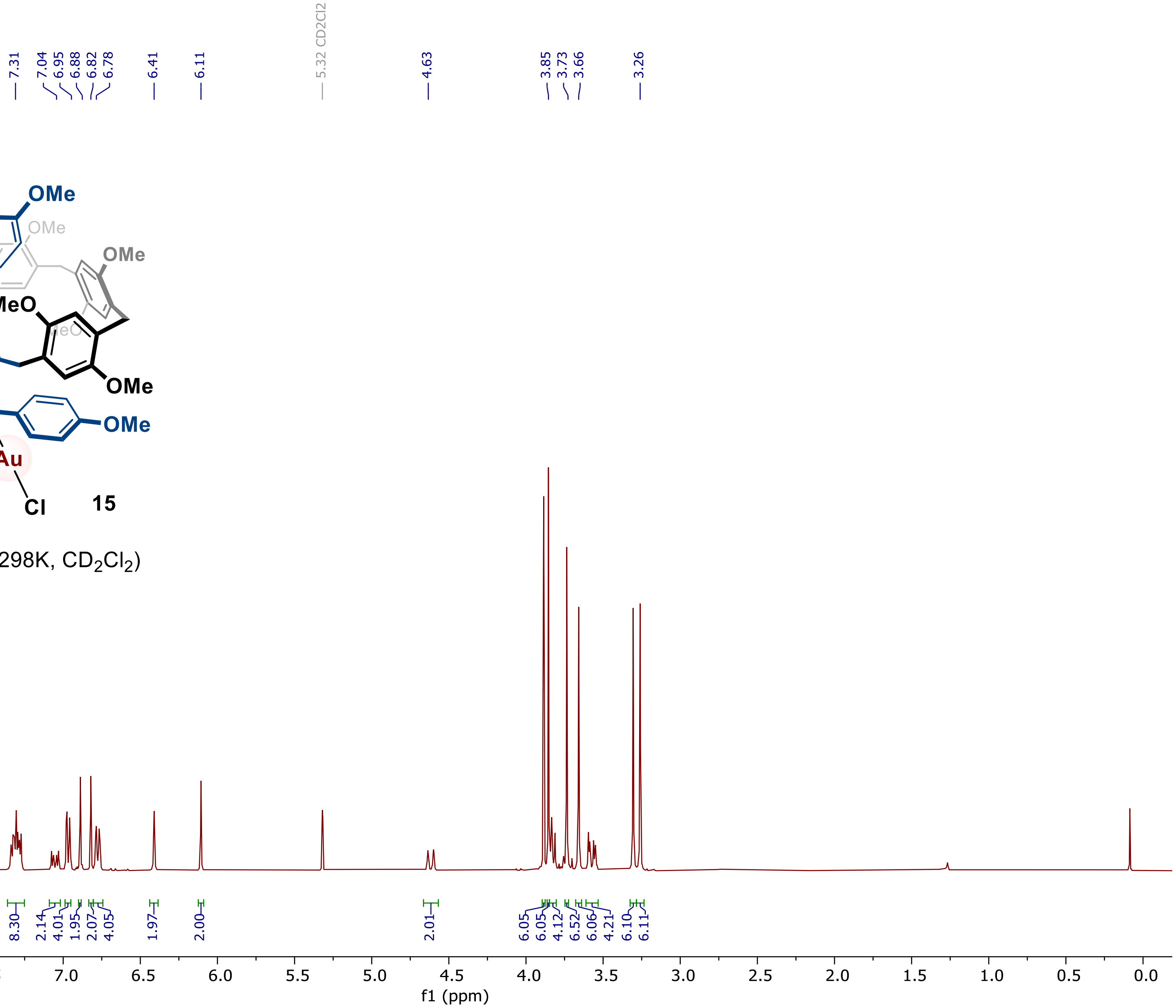

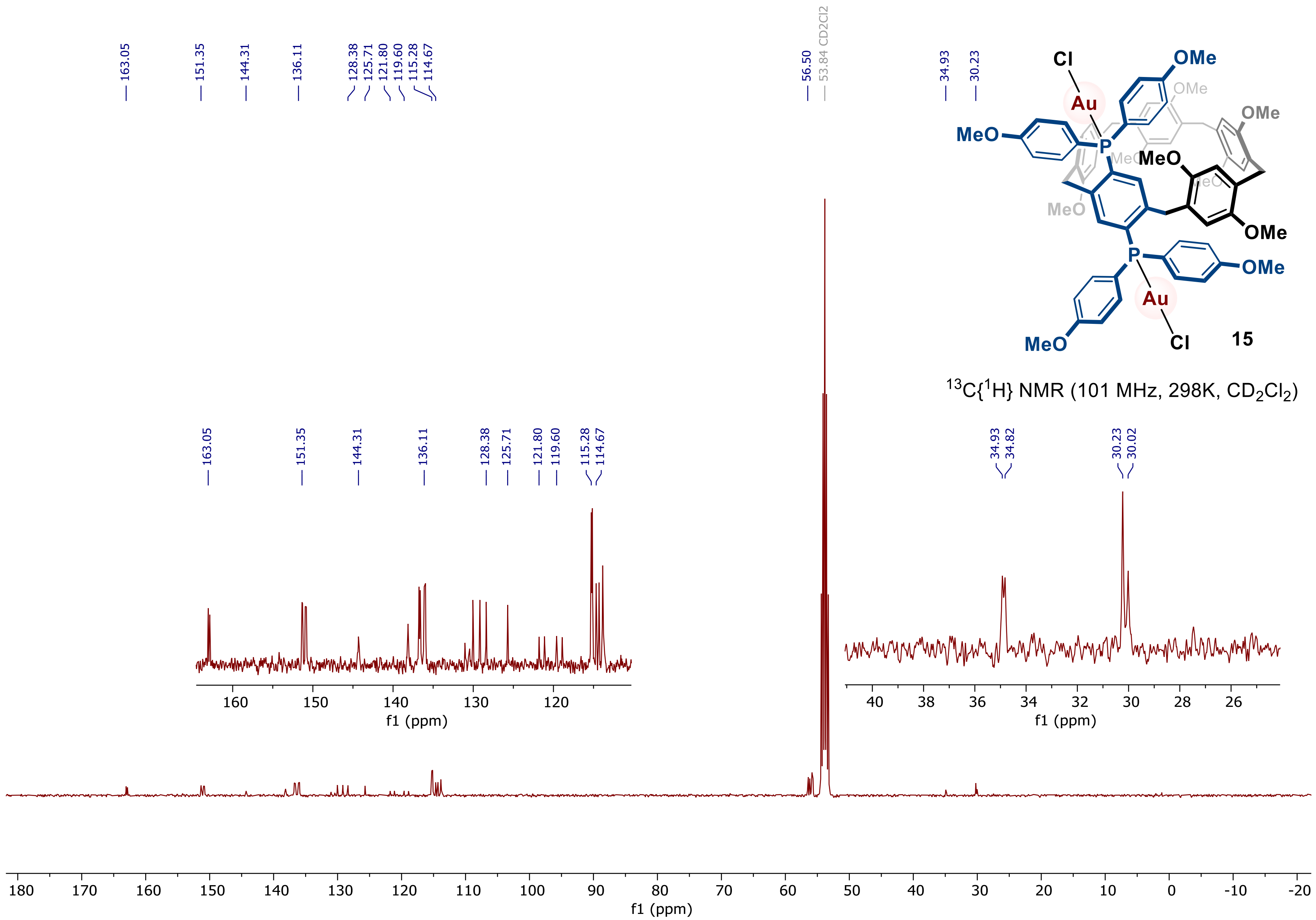

15

$^{13}C\{^{1}H\}$ NMR (101 MHz, 298K, CD_2Cl_2)

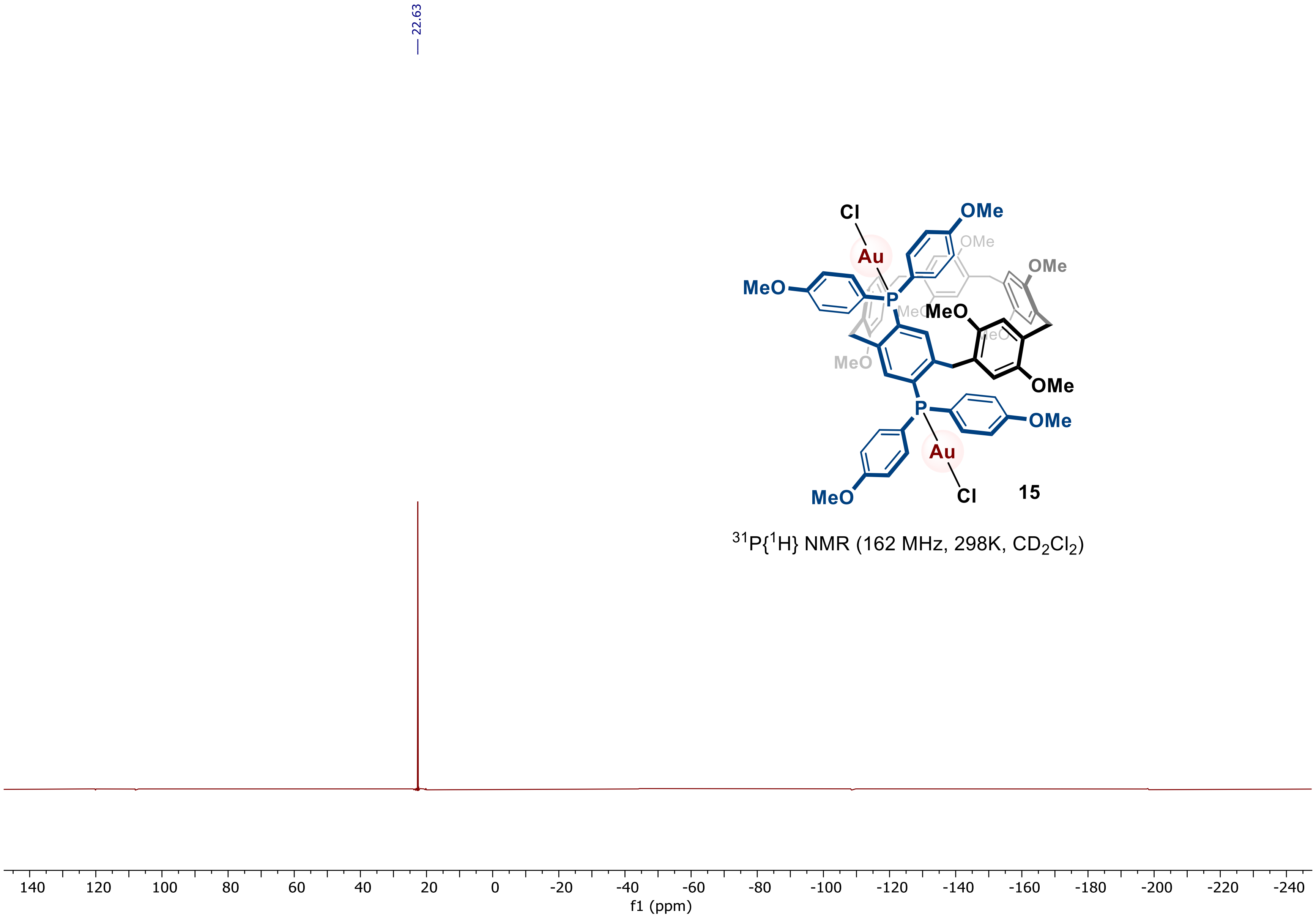

15

$^{31}P\{^{1}H\}$ NMR (162 MHz, 298K, CD_2Cl_2)

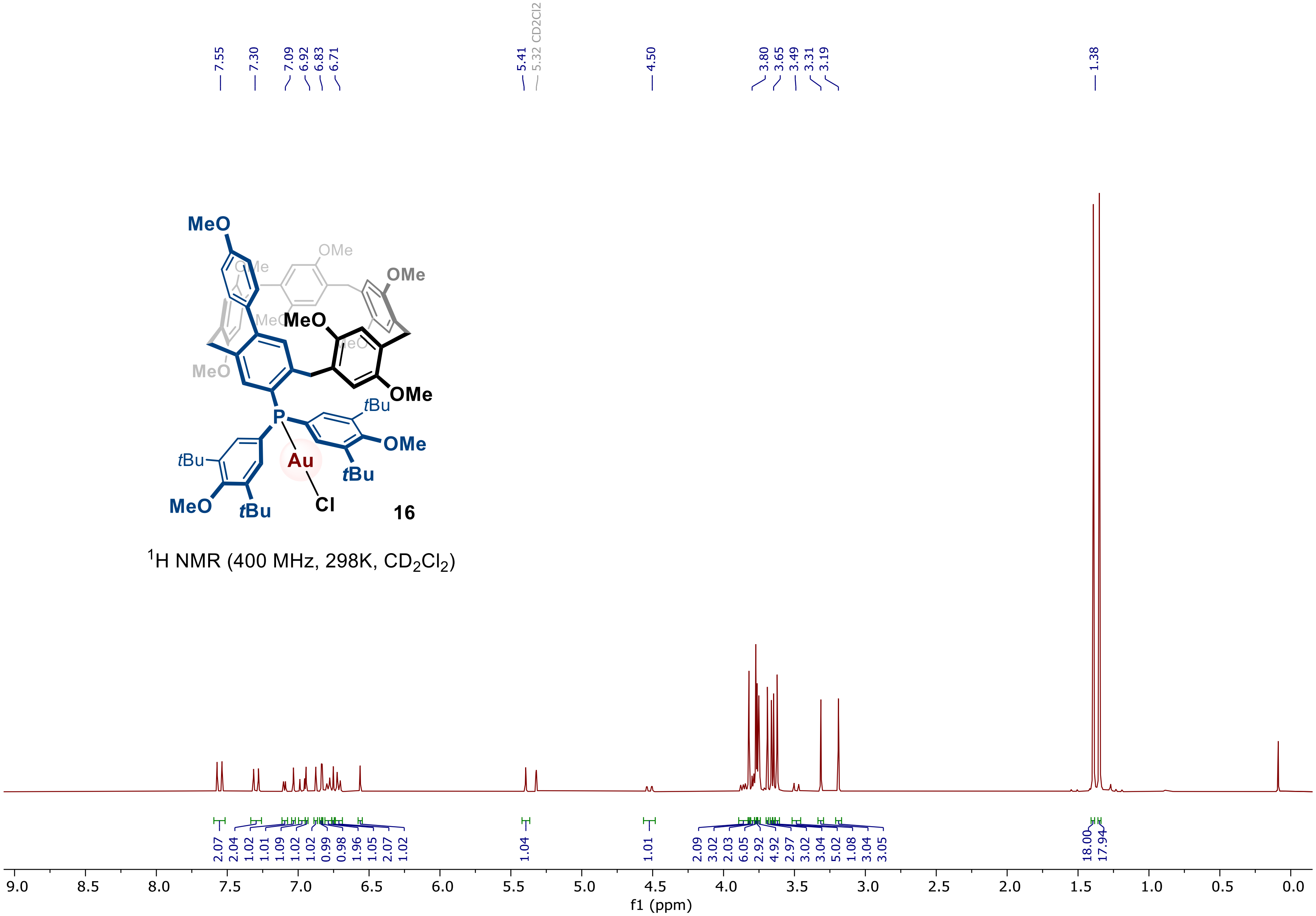

16

^{1}H NMR (400 MHz, 298K, CD_2Cl_2)

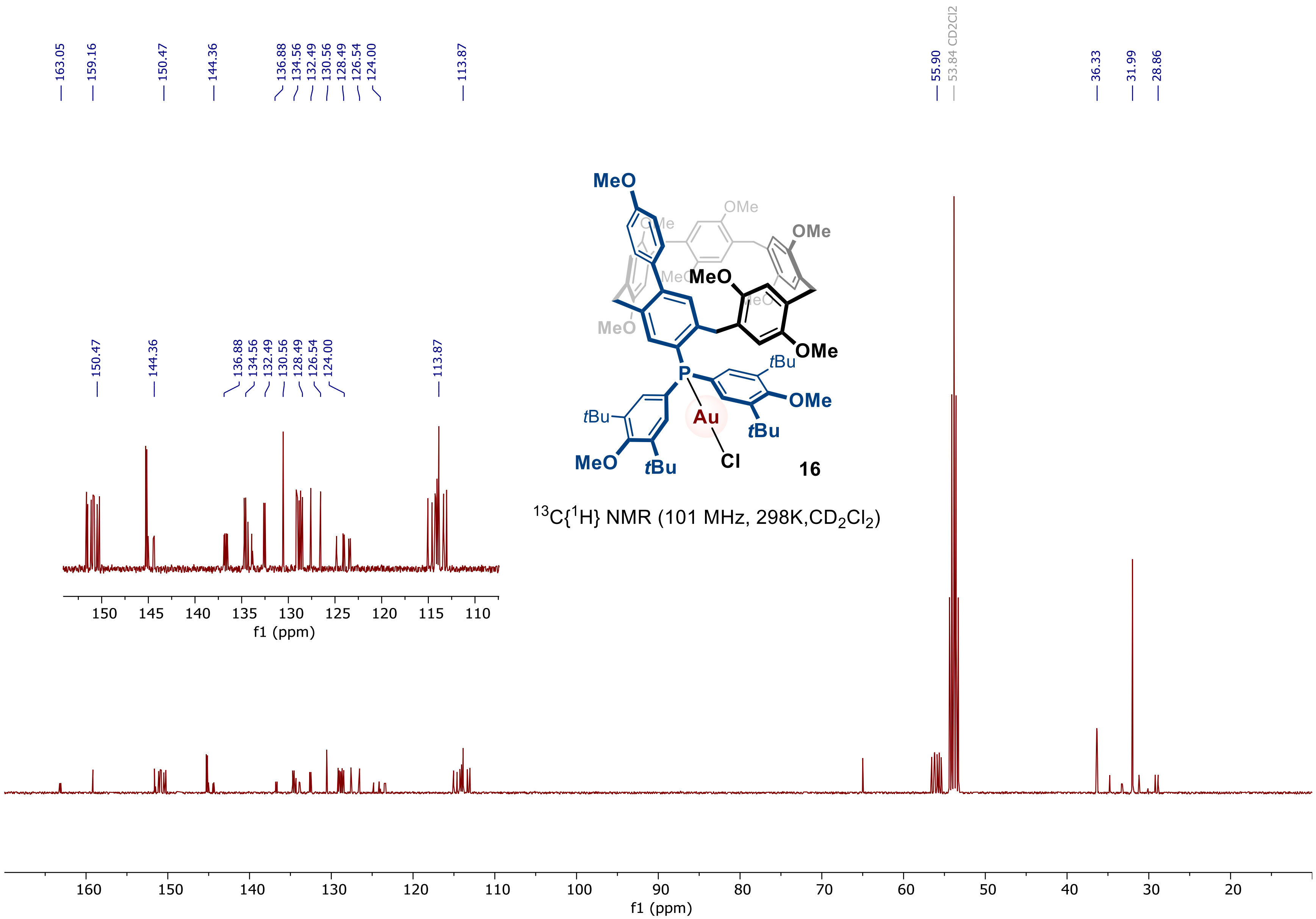

16

$^{13}C\{^{1}H\}$ NMR (101 MHz, 298K, CD_2Cl_2)

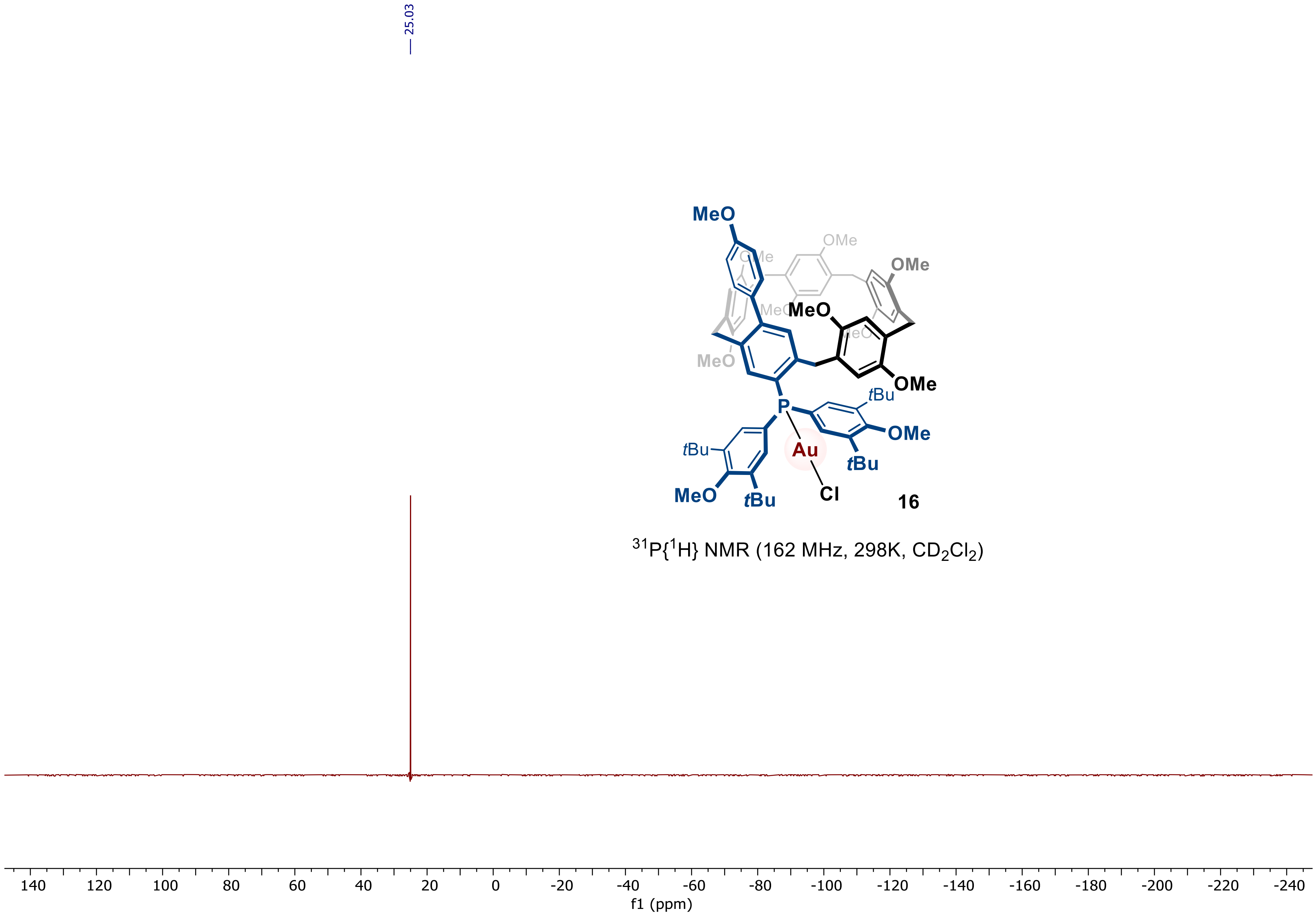

16

$^{31}P\{^1H\}$ NMR (162 MHz, 298K, CD_2Cl_2)

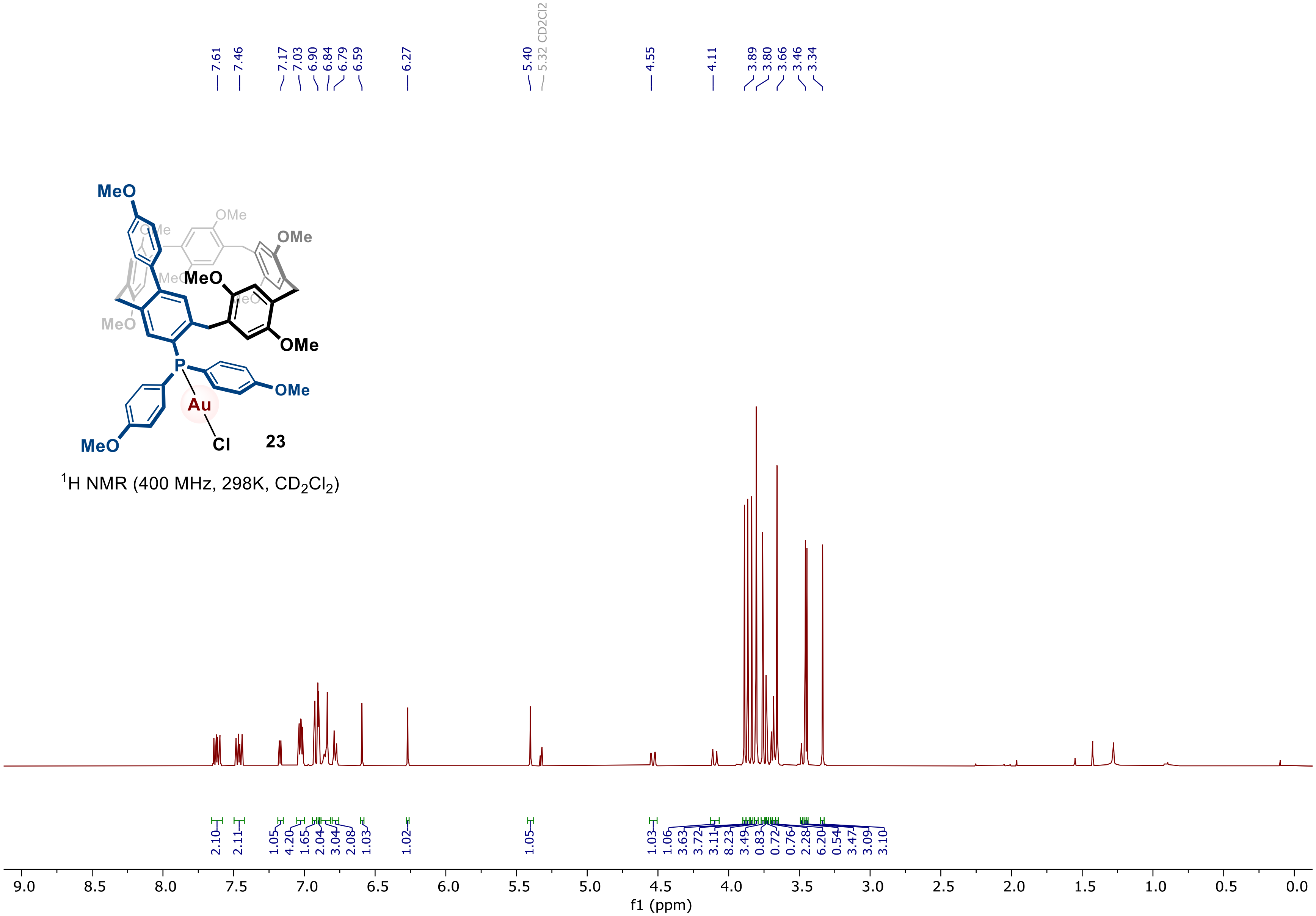

23

^{1}H NMR (400 MHz, 298K, CD_2Cl_2)

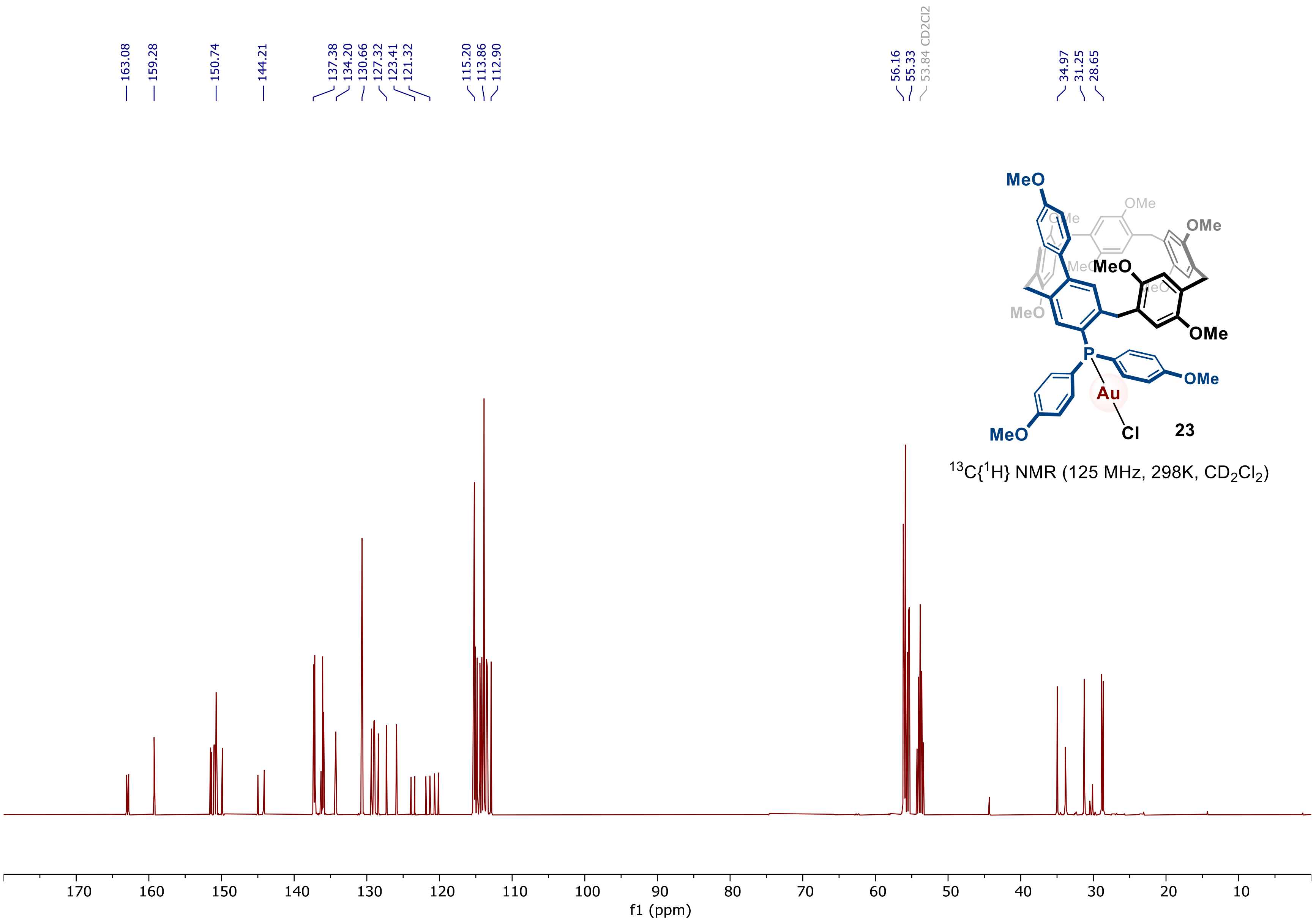

$^{13}C\{^1H\}$ NMR (125 MHz, 298K, CD_2Cl_2)

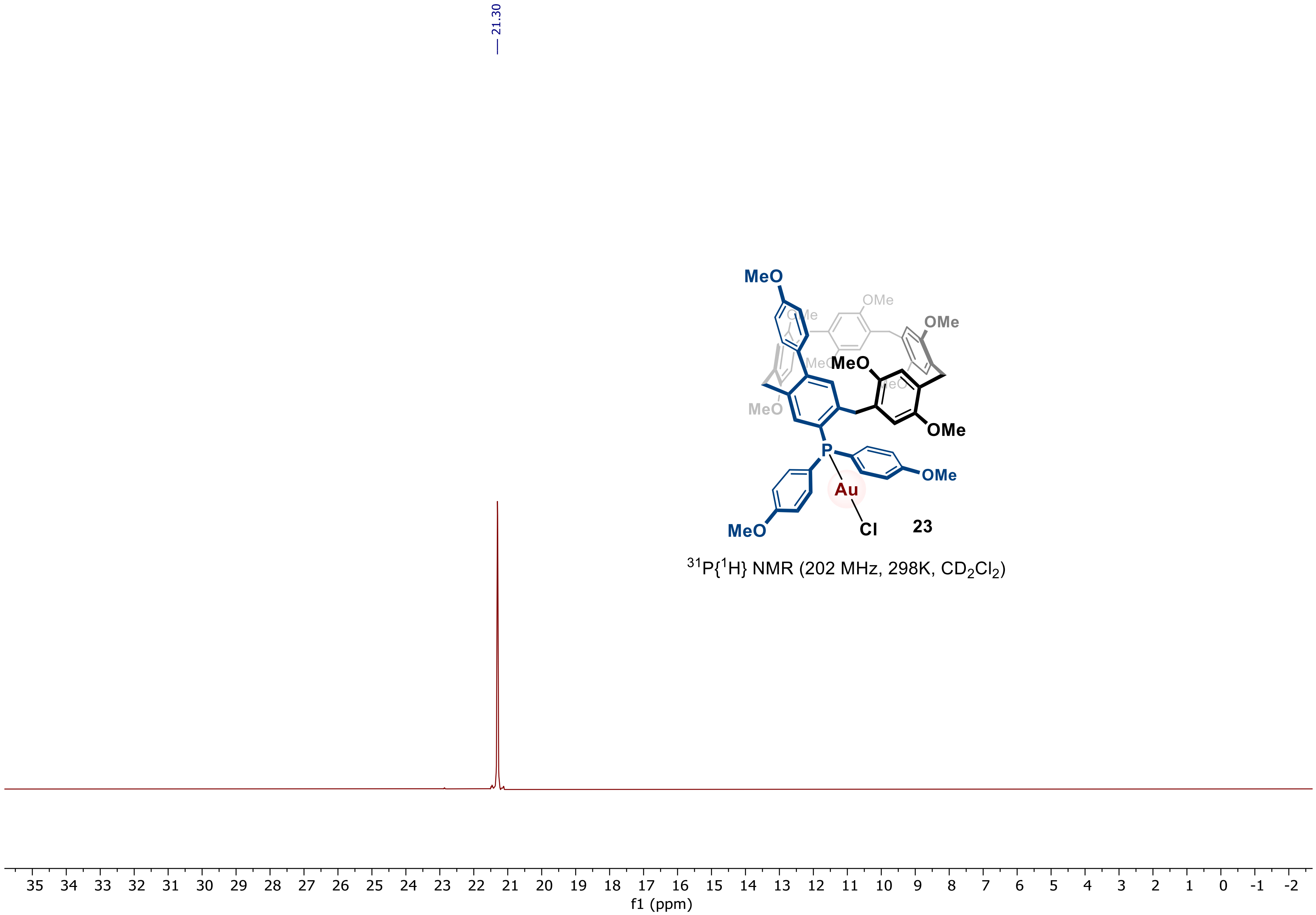

23

$^{31}P\{^{1}H\}$ NMR (202 MHz, 298K, CD_2Cl_2)

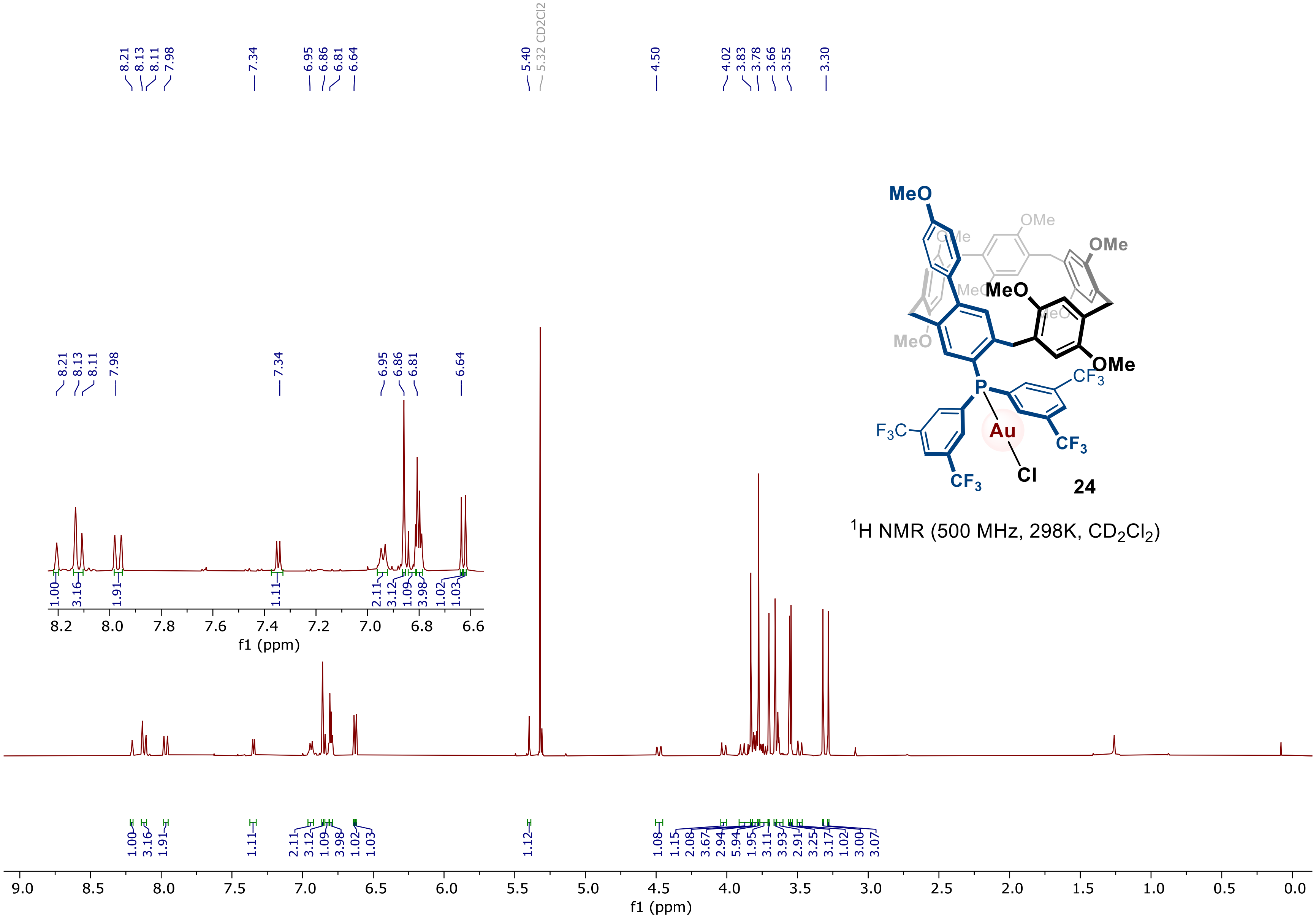

24

^{1}H NMR (500 MHz, 298K, CD_2Cl_2)

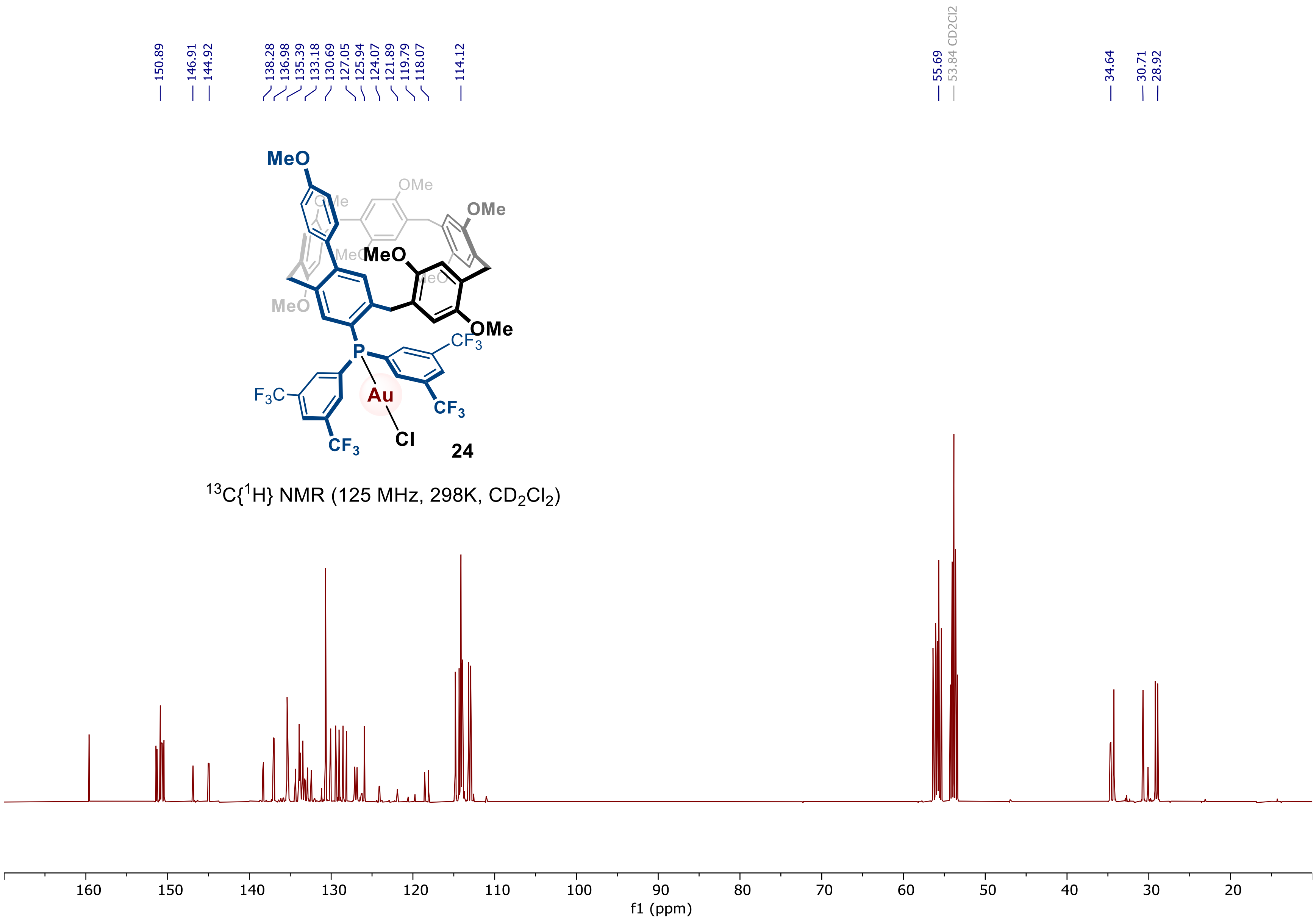

24

$^{13}C\{^1H\}$ NMR (125 MHz, 298K, CD_2Cl_2)

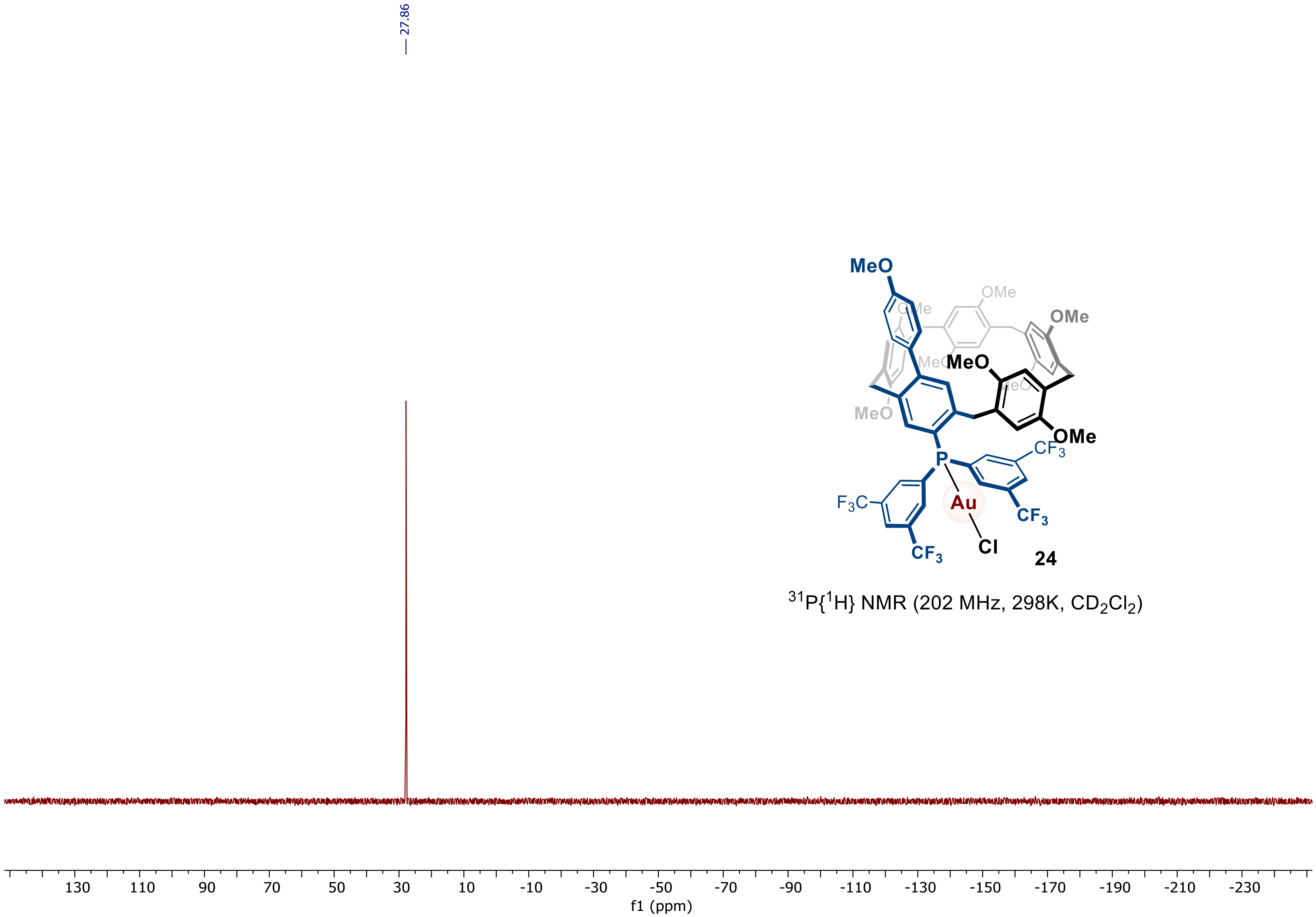

24

$^{31}P\{^1H\}$ NMR (202 MHz, 298K, CD_2Cl_2)

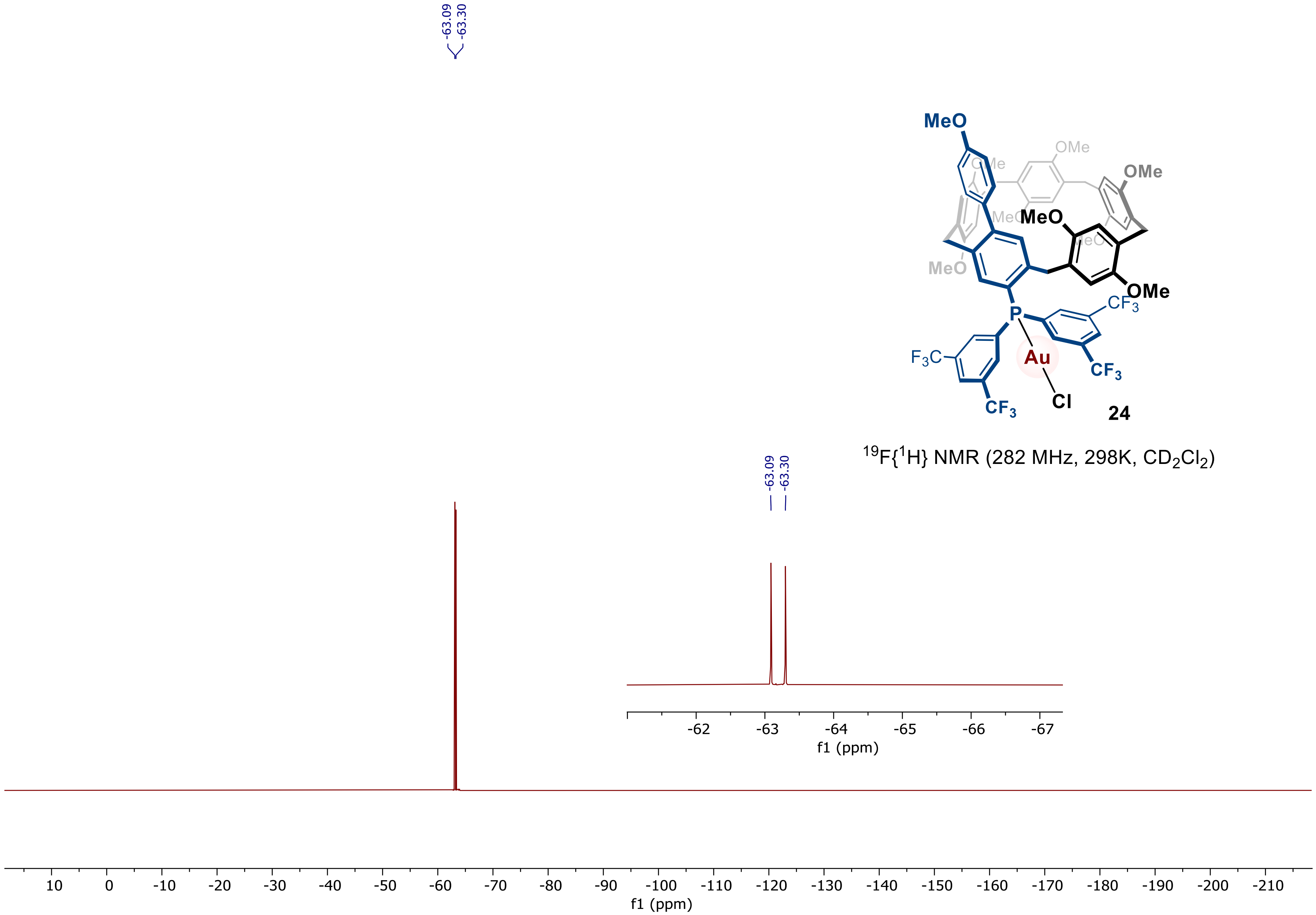

24

$^{19}F\{^{1}H\}$ NMR (282 MHz, 298K, CD_2Cl_2)

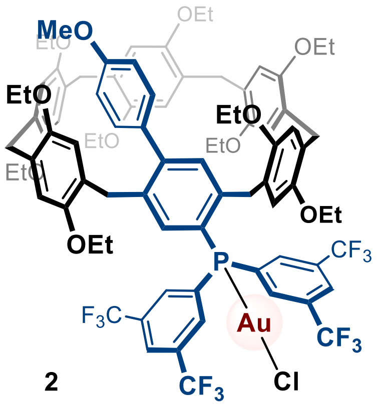

2

^1H NMR (500 MHz, 298K, CD_2Cl_2)

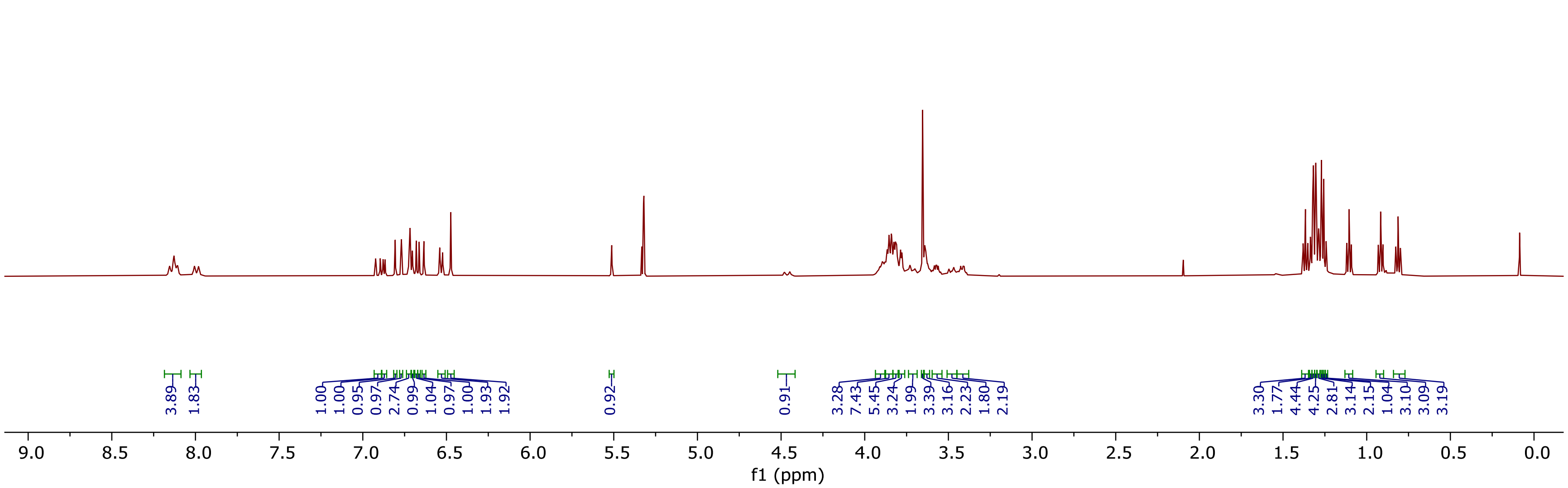

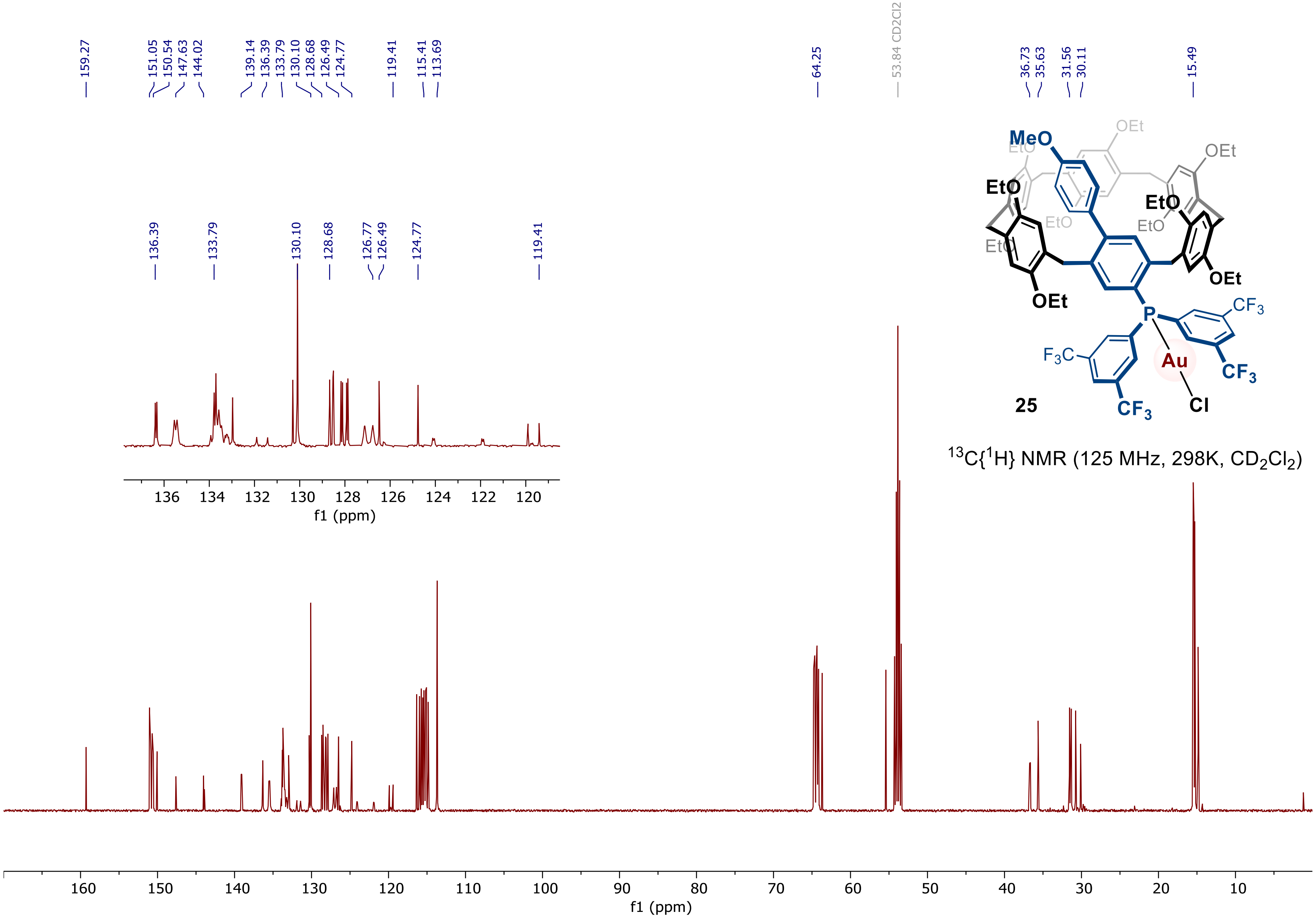

25

$^{13}C\{^{1}H\}$ NMR (125 MHz, 298K, CD_2Cl_2)

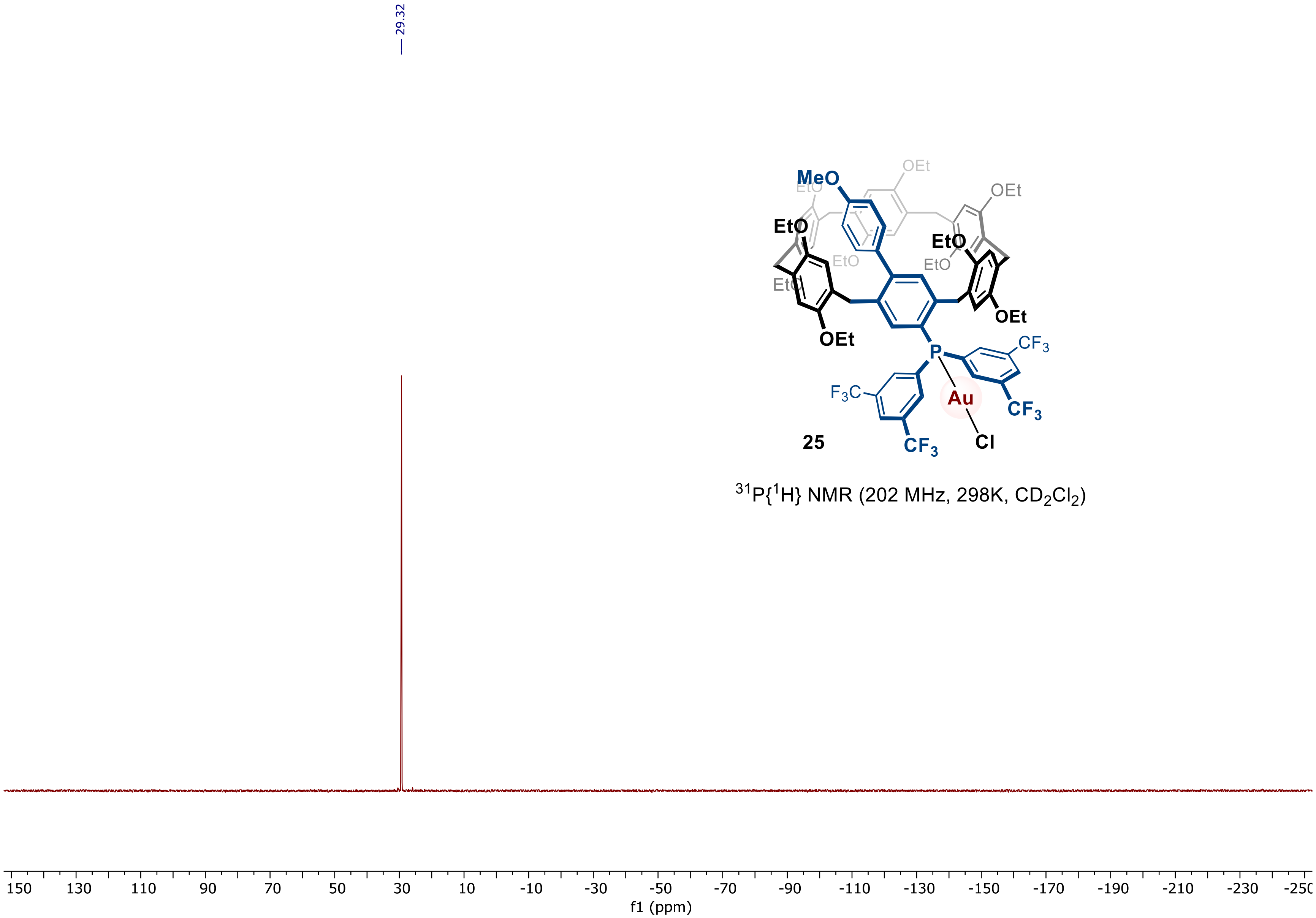

25

$^{31}P\{^{1}H\}$ NMR (202 MHz, 298K, CD_2Cl_2)

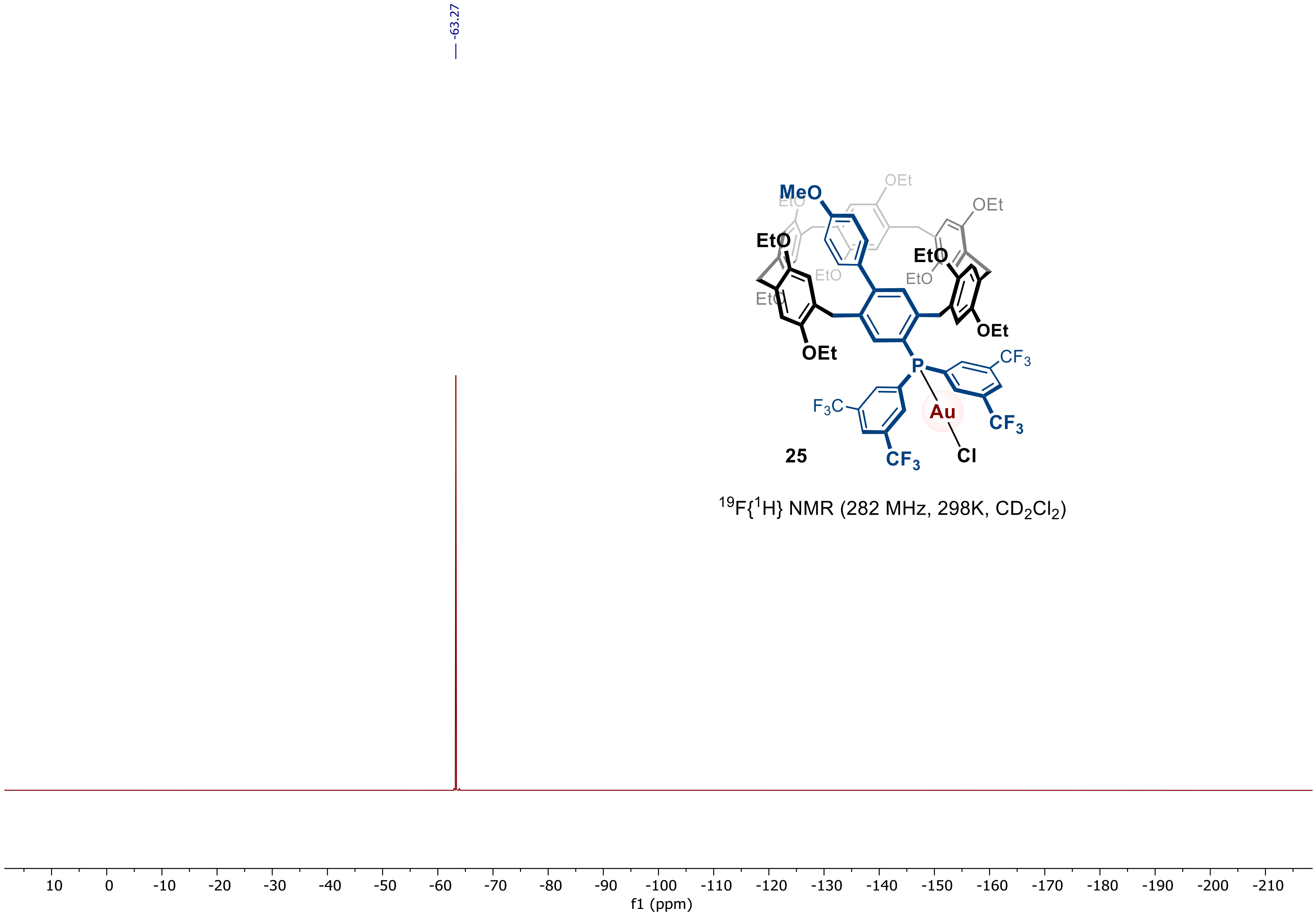

25

$^{19}F\{^1H\}$ NMR (282 MHz, 298K, CD_2Cl_2)
